# Synthesis and Antibacterial Analysis of Analogues of the Marine Alkaloid Pseudoceratidine

**DOI:** 10.3390/molecules25112713

**Published:** 2020-06-11

**Authors:** David Barker, Stephanie Lee, Kyriakos G. Varnava, Kevin Sparrow, Michelle van Rensburg, Rebecca C. Deed, Melissa M. Cadelis, Steven A. Li, Brent R. Copp, Vijayalekshmi Sarojini, Lisa I. Pilkington

**Affiliations:** 1 School of Chemical Sciences, University of Auckland, Auckland 1010, New Zealand; slee526@aucklanduni.ac.nz (S.L.); kvar749@aucklanduni.ac.nz (K.G.V.); kevin.sparrow@auckland.ac.nz (K.S.); mvan126@aucklanduni.ac.nz (M.v.R.); rebecca.deed@auckland.ac.nz (R.C.D.); m.cadelis@auckland.ac.nz (M.M.C.); sli359@aucklanduni.ac.nz (S.A.L.); b.copp@auckland.ac.nz (B.R.C.); v.sarojini@auckland.ac.nz (V.S.); lisa.pilkington@auckland.ac.nz (L.I.P.); 2 MacDiarmid Institute for Advanced Materials and Nanotechnology, Victoria University of Wellington, Wellington 6140, New Zealand; 3 School of Biological Sciences, University of Auckland, Auckland 1010, New Zealand

**Keywords:** marine natural product, analogue synthesis, SAR, pseudoceratidine, antibacterial activity

## Abstract

In an effort to gain more understanding on the structure activity relationship of pseudoceratidine **1**, a di-bromo pyrrole spermidine alkaloid derived from the marine sponge *Pseudoceratina purpurea* that has been shown to exhibit potent biofouling, anti-fungal, antibacterial, and anti-malarial activities, a large series of 65 compounds that incorporated several aspects of structural variation has been synthesised through an efficient, divergent method that allowed for a number of analogues to be generated from common precursors. Subsequently, all analogues were assessed for their antibacterial activity against both Gram-positive (*Staphylococcus aureus*) and Gram-negative (*Escherichia coli*) bacteria. Overall, several compounds exhibited comparable or better activity than that of pseudoceratidine **1**, and it was found that this class of compounds is generally more effective against Gram-positive than Gram-negative bacteria. Furthermore, altering several structural features allowed for the establishment of a comprehensive structure activity relationship (SAR), where it was concluded that several structural features are critical for potent anti-bacterial activity, including di-halogenation (preferable bromine, but chlorine is also effective) on the pyrrole ring, two pyrrolic units in the structure and with one or more secondary amines in the chain adjoining these units, with longer chains giving rise to better activities.

## 1. Introduction

Bacteria are ubiquitous, unicellular microorganisms that occupy critical roles in maintaining life on earth [1,2]. Bacteria are found in a range of diverse environments; from the deep sea to volcanic lakes and desert plains, caves to Antarctic regions and existing on and in other living organisms, including humans [3]. The relationship between humans and bacteria is complex and while most bacteria have a symbiotic relationship with humans, some are pathogenic and are the cause of diseases [4]. In recent history, combatting diseases caused by bacterial infections has been addressed through the extensive development and use of antibacterial agents [5].

Due to the overwhelmingly successful outcomes achieved by the discovery of early antibacterial drugs such as penicillin and sulfa drugs, the intensive research surrounding antibacterial agents conducted during the 1950s–70s led to the establishment of a wide range of antibacterial classes [6]. However, no new classes of antibacterial agents have been discovered since this era—this fact, coupled with the rise in pathogenic drug-resistant bacterial species predicates the need for new antibacterial classes and agents to be found [6,7].

In recent history, natural products have proved to be a great source of inspiration in the pursuit of drug therapies. This is exemplified by the fact that a range of therapeutics have been inspired by compounds of natural origin—a significant proportion (~40%) of existing drugs on the market are either natural products or derivatives thereof [8]. While almost all of these natural product-derived therapeutics are developed from natural products isolated from terrestrial organisms, there is an ever-increasing interest in the discovery and development of drugs from marine natural products [9,10,11]. This movement can be attributed to the diverse range of novel and unique chemical structural motifs that are found in marine organisms in conjunction with the fact that natural products of marine origins typically have greater biological activity than terrestrial natural products [9,11,12].

Marine sponges, in particular, have been found to be a rich marine-based source of bioactive molecules—40% of the total secondary metabolites discovered in marine organisms originate from marine sponges [13]. The rationale for this is that marine sponges are sessile organisms that rely on the production of secondary metabolites to act as a chemical defense mechanism against predators [14].

**Figure 1:**
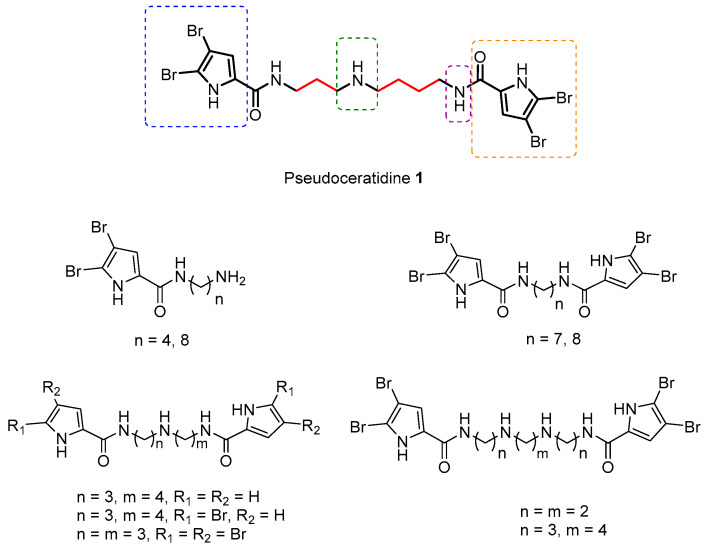
Molecular structure of pseudoceratidine **1** and previous analogues for which that antibacterial activity has been investigated. On psuedoceratidine **1**, the features investigated in this study are indicated by colour; the type and number of halogenation on the pyrrolic ring (blue), the length and symmetry of polyamine chain (red); the number of secondary amine groups—none, one or two (green)—the presence of a second pyrrolic unit (orange) and, if no pyrrolic unit, an alternative functionality (purple).

Pseudoceratidine **1** was first extracted from the marine sponge *Pseudoceratina purpurea* in 1996 in coastal regions around Japan and has instigated enthusiasm in further investigation of its analogues due its relatively simple molecular structure, yet potent biological activity [15,16,17,18,19]. Structural elucidation of pseudoceratidine **1** showed it to be an asymmetrical alkaloid with bromopyrrole units at each end of the triamine, spermidine, chain (Figure 1) [15]. Thought to function as a natural biofouling agent, pseudoceratidine **1** was initially tested for its antifouling activity against the marine larval organism *Balanus amphitrite* (EC_50_ 8 µg/mL) [15]. Subsequent to this, its anti-fungal (against *Candida albicans* minimal inhibitory concentration (MIC) 32 µg/mL) and antibacterial (against Gram-positive *Staphylococcus aureus* MIC 4 µg/mL, *Listeria monocytogenes* MIC 5 µg/mL and Gram-negative *Escherichia coli* MIC 32 µg/mL, *Pseudomonas aeruginosa* MIC 128 µg/mL) activities were investigated [17,18,19]. In addition to this, pseudoceratidine **1** was assessed for its anti-malarial activity against the malarial protist *Plasmodium falciparum* EC_50_ 1 µM) [16]. This testing verified pseudoceratidine **1** as a promising lead compound in the search for new antibacterials.

In addition to the pseudoceratidine 1, a series of closely related analogues have been previously prepared and assessed for their antibacterial activity (Figure 1) [17,19]. These various series have explored the effect of omitting one of the pyrrolic units (i.e., monoacyl derivatives) and bromination (or lack thereof) of the pyrrolic units and limited exploration of the length and symmetry of the amine chain. It was found that analogues with the most effective biological activities were those that possessed a di-brominated pyrrole in addition to a bis-amide structure with a polyamine chain with ≥1 positive charge at physiological pH. Despite these explorations and the development of an initial structure activity relationship (SAR) for this class of compounds, thus far there are no reported pseudoceratidine analogues that have better activity than pseudoceratidine **1** itself.

In an effort to extend the previous limited SAR and to investigate the effect of a variety of halogenations (degree and type of halogen) as well as a large variety of polyamine chains (both symmetric and asymmetric chains with no, one or two secondary amines), it was decided to synthesise a large and comprehensive library of analogues with these variations and assess their biological activity against representative Gram-positive (*S. aureus*) and Gram-negative (*E. coli*) bacteria.

## 2. Results and Discussion

**Scheme 1:**
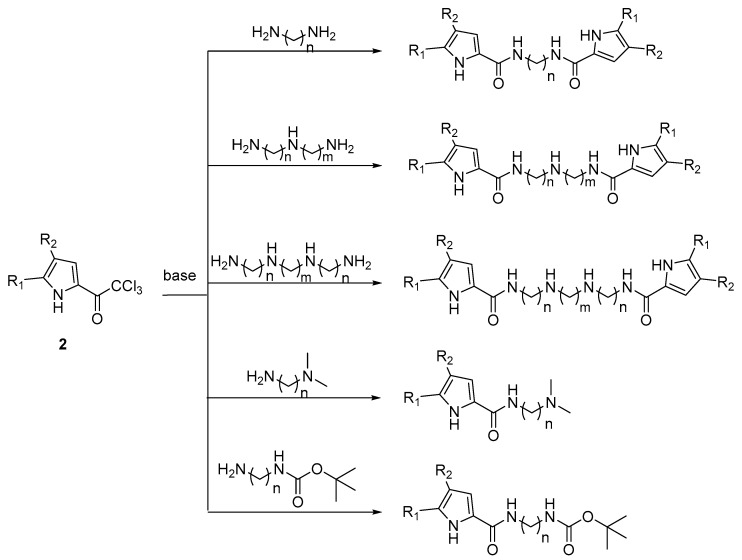
General strategy and the pseudoceratidine analogues targeted.

The general strategy for the synthesis of the proposed compounds was the base-mediated coupling of trichloroacetyl halogenated pyrroles **2** with a large variety of polyamines (Scheme 1).

**Scheme 2:**
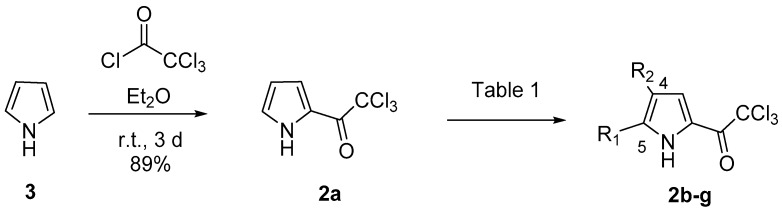
Synthesis of substituted pyrroles **2a**–**g**.

As such, the first major task was the generation and subsequent halogenation of 2-trichloroacetyl pyrrole **2a** to give the desired mono- and di-substituted pyrroles **2b**–**g**. The synthesis of 2-trichloroacetyl pyrrole **2a** was achieved through the selective electrophilic aromatic substitution of commercially available pyrrole **3** with trichloroacetyl chloride following the method reported by Dubis et al. (Scheme 2) [20]. Recrystallisation of the crude product with *n*-hexanes and a minimal amount of diethyl ether provided the desired product **2a** in a yield of 89%.

Following its synthesis, pyrrole **2a** was chlorinated with one equivalent of sulfuryl chloride to provide the C-4 mono-chloro pyrrole **2b** as the major product (Table 1) [21]. The C-4 position is the preferential site of reaction owing to the presence of the strongly electron-withdrawing properties of the trichloroacetyl group and longer and/or more forcing conditions were required for additional substitution at the C-5 position. As such, employing the same chlorinating reagent in a higher concentration and longer reaction time provided the di-chloro analogue **2c** in 85% yield. Following the reaction conditions reported by Behrens et al., the synthesis of 4-bromo-2-trichloroacetyl pyrrole **2d** was accomplished using a similar reaction procedure as that employed for mono-chloro pyrrole **2b**, but using bromine (1 equiv.) for 10 min [17]. Furthermore, doubling the amount of bromine and using acetic acid as a solvent to facilitate a higher reaction temperature allowed for the high-yielding production of the di-brominated pyrrole **2e** (91%) [22,23]. Finally, the selective iodination of 2-trichloroacetyl pyrrole **2a** with iodine monochloride provided 4-iodo-2-trichloroacetyl pyrrole **2f** [24], while diiodination was achieved using a combination of iodine and silver trifluoroacetate according to previously reported reaction conditions [22,25], giving **2g**.

**Scheme 3:**
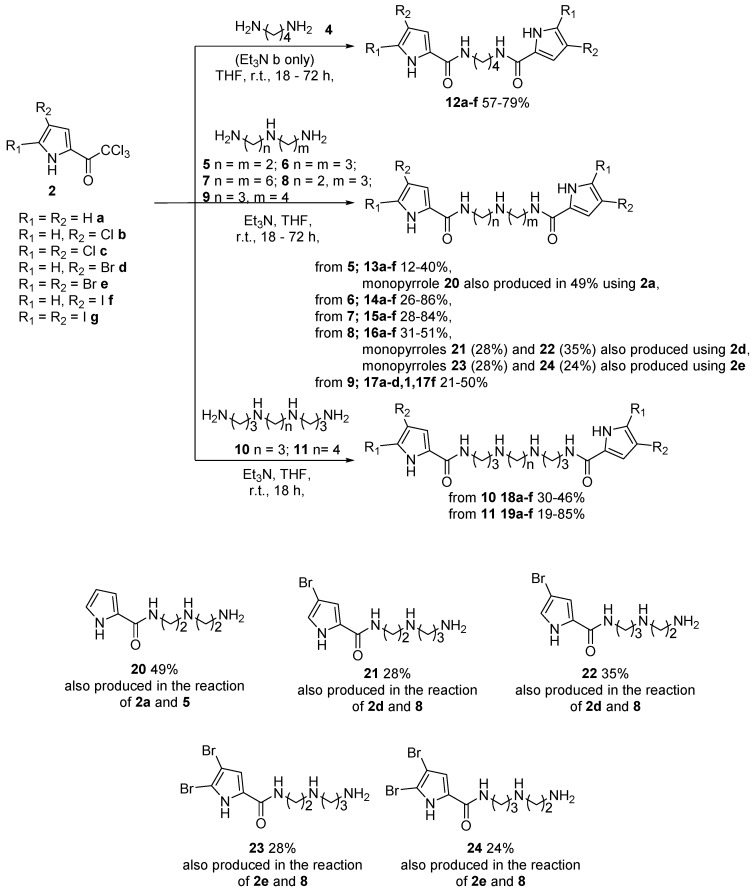
Synthesis of pseudoceratidine analogues **12**–**19a**–**f**, **20**–**24**. NB: no reactions involving diiodo pyrrole **2g** were successful.

Following the successful synthesis of the halogenated 2-trichloroacetylated pyrroles **2b**–**g**, all that was required for the synthesis of the targeted dipyrrole pseudoceratidine analogues was the coupling of these pyrroles **2a**–**g** with a range of polyamines, namely putrescine **4**, *N’*-(2-aminoethyl)ethane-1,2-diamine **5**, *N’*-(3-aminopropyl)propane-1,3-diamine **6**, *N’*-(6-aminohexyl)hexane-1,6-diamine **7**, *N’*-(2-aminoethyl)propane-1,3-diamine **8**, spermidine **9**, *N’*,*N’*-(propane-1,3-diyl)bis(propane-1,3-diamine) **10** and spermine **11**. The desired couplings were achieved using two equivalents of the trichloroacetylated pyrrole **2** and the polyamine in dry THF at room temperature for 18–72 h (Scheme 3) [17]. This reaction was generally carried out with the addition of Et_3_N, which was used to improve the reactivity of the pyrrole. While this addition was necessary for almost all amines, it was not generally required for reactions involving putrescine **4**, except for the generation of analogue **12b**, and the putrescine analogues **12a**–**f** being successfully produced in moderate to very good yields (57–79%). Reaction of amines **5**–**11** with pyrroles **2** resulted in the generation of a large library of di-pyrrole analogues **12**–**19** (including pseudoceratidine **1**) with a range of linear chains and halogenation patterns on the pyrrolic units. However, it should be noted that all of the reactions involving diiodo-substituted pyrrole **2g** gave none of the desired amides **12g**–**19g** possibly due to S_N_Ar of the labile iodine substituents.

**Scheme 4:**
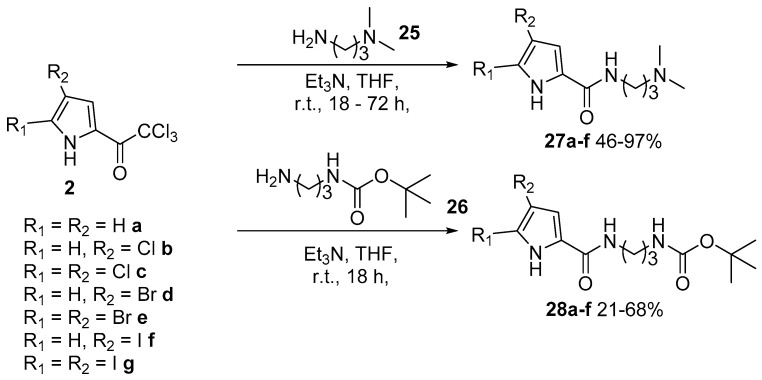
Synthesis of mono-pyrrole pseudoceratidine analogues **27a**–**f**, **28a**–**f**. NB: no reactions involving diiodo pyrrole **2g** were successful.

Mono-pyrrole products were seldom isolated as products of these reactions, with the exception of mono-pyrrole **20**, which was synthesised in addition to the desired dipyrrole **13a**, from pyrrole **2a** and *N’*-(2-aminoethyl)ethane-1,2-diamine **5**. Additionally, the reaction of brominated pyrroles **2d** and **2e** with the asymmetric triamine *N’*-(2-aminoethyl)propane-1,3-diamine **8**, formed appreciable amounts of mono-pyrroles **21**–**24** along with their respective di-pyrrole analogues **16d** and **16e** (Scheme 3). To generate further mono-pyrrole pseudoceratidine analogues, *N’*,*N’*-dimethylpropane-1,3-diamine **25** and *tert*-butyl (3-aminopropyl)carbamate **26** were reacted with pyrroles **2a**–**g**, using the aforementioned coupling procedure, providing mono-pyrroles **27a**–**f** and **28a**–**f**, respectively (Scheme 4). Once again, it was found that di-iodo pyrrole **2g** did not undergo the reaction. In total, 65 compounds were successfully synthesised, achieving the goal of generation of a large library of pseudoceratidine analogues which, together, it was hoped would allow for the desired SAR to be developed.

The antibacterial activity of all 65 synthesised compounds against both Gram-positive (*S. aureus*) and Gram-negative (*E. coli*) bacteria was assessed by measuring the MIC (minimal inhibitory concentration). The known antibiotics streptomycin and polymyxin B were used as positive controls against *S. aureus* and *E. coli*, respectively (Table 2). The measured MIC values of pseudoceratidine **1** were in accordance with those previously reported in the literature [17,18,19] and unless otherwise stated, the highest concentration of each compound that was tested was 32 µM for *S. aureus* and 128 µM for *coli*.

**Figure 2:**
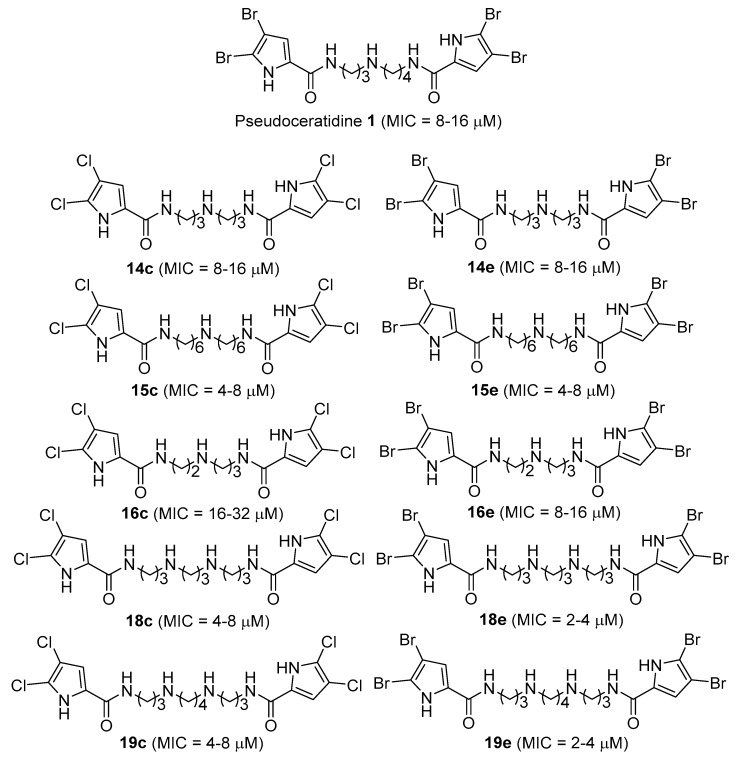
Pseudoceratidine **1** and the analogues shown to display comparative or more potent antibacterial activity than the natural product pseudoceratidine **1**. Gram-positive minimal inhibitory concentration (MIC) values are also given. N. B. **16c** showed equivalents activity to **1** against Gram-negative bacteria.

Pleasingly, ten synthetic analogues were equally or more potent than the natural product, pseudoceratidine **1**—an excellent result considering no previously synthesised pseudoceratidine analogue had been found to exhibit comparable activity to the lead compound (Figure 2). It was found that no mono-pyrrole analogues showed activity of note, while the most potent analogues were those that had di-bromo or di-chloro substitution on each of its two pyrrole moieties. This particularly highlights both the importance of the presence of both pyrrole units, as well as the positive effects of dihalogenation of each pyrrole. It can also be seen that brominated analogues display more potent activity than their chlorinated counterparts. The lack of activity exhibited by the putrescine analogues **12a**–**f** attest to the necessity of at least one secondary amine functionality in the linear chain, although it should be noted that the *N’*-(2-aminoethyl)ethane-1,2-diamine derived analogues **13a**–**f** also displayed no notable antibacterial activity despite possessing a secondary amine in its structure. While the presence of secondary amines in the chain was shown to be important, the symmetry of this chain did not seem to hold any significance on its activity, although it was observed that analogues with longer chains seems to be more active than those with shorter chains. The more potent activity in Gram-positive *S. aureus* appears to indicate that the antibacterial effects exerted by the compounds is dictated by the difference in the membrane composition of the bacterial species with these compounds [26]. Gram negative bacteria are known to have an additional outer-membrane barrier which is difficult to penetrate [27,28] so it appears these compounds are far more active against bacteria that possess a single lipid membrane. Analogues possessing a longer carbon chain are potentially able to penetrate the bacterial cell membrane more effectively.

Furthermore, the cytotoxicity of pseudoceratidine **1** and these ten analogues with comparable or better anti-bacterial activities was determined against HEK293 human embryonic kidney cells (Table 3). As can be seen, many of the most analogues with potent anti-bacterial activity were also shown to be cytotoxic against human cells at 16 µM. Of particular note, however, were two of the analogues, **18c** and **19e**, which showed almost no cytotoxic activity at concentrations 2–4 times the MIC. It is interesting that other analogues similar to these, including **19e** itself, have been tested for their cytotoxicity and in general were found to be far less toxic versus the HepG2 liver cell line [16]. This shows that these compounds not only exhibit different activities verses various bacteria but also against different mammalian cell lines. Whilst there is a clear SAR associated with antibacterial acitivty, stated above, there is no obvious SAR trends for cytotoxic activity.

## 3. Experimental Procedures

### 3.1. General Details

All reactions were carried out under a nitrogen atmosphere in dry, freshly distilled solvents unless otherwise noted. NMR spectra were recorded on a either a 300 or 400 MHz spectrometer. Chemical shifts are reported relative to the solvent peak of chloroform and/or CDCl_3_ (δ 7.26 for ^1^H and δ 77.16 for ^13^C, respectively) or *d*_6_-DMSO (δ 2.50 for ^1^H and δ 39.5 for ^13^C). ^1^H-NMR data is reported as position (δ), relative integral, multiplicity (s, singlet; d, doublet; t, triplet; q, quartet; m, multiplet; br, broad peak; qd, quartet of doublets), coupling constant (J, Hz), and the assignment of the atom. ^13^C-NMR data are reported as position (δ) and the assignment of the atom. NMR assignments were performed using HSQC and HMBC experiments. High-resolution mass spectroscopy (HRMS) was carried out by electrospray ionization (ESI) on a MicroTOF-Q mass spectrometer. Unless noted, chemical reagents were used as purchased.

### 3.2. General Procedures

General Procedure A: the coupling of substituted pyrroles with amine for synthesis of di-pyrrole analogues 12–19.

To a solution of pyrrole **2** (2 equiv.) in dry THF at r.t., under an atmosphere of nitrogen, amine (1 equiv.) and triethylamine (4 equiv.) were added dropwise. The mixture was stirred for 18–72 h and the solvent was removed in vacuo to give the crude product, which was purified as stated.

General Procedure B: the coupling of substituted pyrroles with amine for synthesis of mono-pyrrole analogues 27 and 28.

To a solution of pyrrole **2** (1 equiv.) in dry THF at r.t., under an atmosphere of nitrogen, amine (1 equiv.) and triethylamine (4 equiv.) was added dropwise. The mixture was stirred for 18–72 h and the solvent was removed in vacuo to give the crude product which was purified as stated.

### 3.3. Synthesis of Compounds

The experimental procedures and characterisation data for all synthesised compounds are provided in the supporting information file.

### 3.4. Antibacterial Assays

The MIC was determined using the broth microdilution method [29,30,31,32]. Mueller Hinton (MH) broth base was prepared in milli-Q water, then autoclaved at 120 °C for 1 h before allowing it to cool to room temperature. A single colony of each bacterial strain (*E. coli* DH5α and *S. aureus* 16207) was inoculated in MHB and incubated at 37 °C overnight. Overnight cultures were adjusted to 10^6^ CFU/mL in MHB according to the McFarland Standard,100 μL of which was further diluted to a total of 10 mL in the media. The stock solutions of the compounds were prepared at 12.8 mM in purified DMSO. The stock solutions were further diluted in milli-Q water to 64 μM in 0.5% DMSO for testing against *S. aureus*, and 256 μM in 2% DMSO for testing against *E. coli*. Compounds that were not soluble in these solvent mixtures were further diluted in half to get clear solutions for the bioassay. DMSO is known to have weak antibacterial activity [33]. To exclude the antibacterial effect of DMSO from the results, a solution of 2% DMSO in milli-Q water was used as an additional control in the experiments.

The compounds and three controls (the natural product pseudoceratidine **1** for both strains, streptomycin for *S. aureus*, and polymyxin B for *E. coli*) then underwent a dilution series. The assay was performed by adding 50 μL of each compound solution at the different concentrations and 50 μL of the diluted bacterial culture to the different wells of a 96-well microtiter plate. The experiments were performed at least thrice and in triplicates. MIC after 24 h of incubation was determined by both visual inspection and absorbance at 600 nm using an EnSpire Multimode plate reader.

### 3.5. Cytotoxicity Assay

As described previously [34], cell proliferation was measured by sulforhodamine B colorimetric assay in which 2000 HEK293 human embryonic kidney cells were seeded in 96-well plates in the presence of varying concentrations of drugs. The sulforhodamine B colorimetric assay [35], which is based on the measurement of cellular protein content, was used to measure cell density. After drug treatment for 3 days, cells were fixed with 10% (*w*/*v*) trichloroacetic acid and stained with 0.057% (*w*/*v*) sulforhodamine B for 30 min, and the excess dye was removed by washing repeatedly with 1% (*v*/*v*) acetic acid. The protein-bound dye was dissolved in Tris base solution (10 mM) for optical density determination at 510 nm using a microplate reader. All experiments were done in triplicate, and were repeated at least twice. The optimal cell densities were previously determined to select initial cell densities that ensured that the cells were in the logarithmic phase for the experiments.

## 4. Conclusions

**Figure 3:**
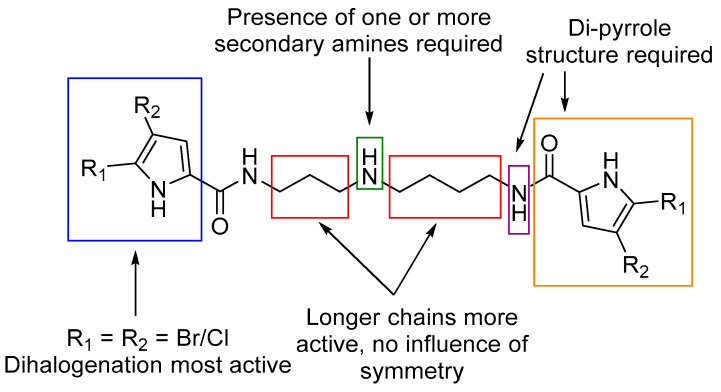
Summary of the SAR of pseudeoceratidine analogues revealed during this investigation.

Pseudoceratidine **1** and 64 closely-related analogues have been successfully synthesised, constituting a comprehensive series of compounds that explore structural motifs, most of which have not previously been investigated before. This synthesis utilised an efficient, divergent methodology that allowed for a large number of final compounds to be synthesised from common starting materials. Following the successful synthesis of this library of compounds, antibacterial testing against both Gram-positive and Gram-negative bacteria was performed, allowing for significant insight into the SAR of pseudoceratidine **1** and the establishment of essential structural features that are required for its antibacterial activity. It was able to be established that these analogues are most effective against Gram-positive bacteria and furthermore that features essential for good anti-bacterial activity include two pyrrole units and the presence of one or more secondary amines in the linear chain between units. It was also verified that the halogenation of the pyrrole units is essential for activity, with dichloro and dibromo pyrroles featuring in the most active analogues. It was also shown that while the symmetry of the chain was not important, longer chain compounds were more active. This work afforded analogues with more potent activity than the parent compound pseudoceratidine **1** and allowed for the establishment and verification of a detailed antibacterial SAR (Figure 3). This work has highlighted the ability to prepare compounds in this class with improved activity over the natural product and provides an excellent basis for further investigation into analogues with even greater potency. Whilst some of the most active compounds also showed mammalian cell cytotoxicity, the novel compound **18c** and **19e** were found to be potent antibacterial agents whilst having almost no cytotoxic activity.

## Figures and Tables

**Table 1:** Reaction conditions and outcomes for the synthesis of halogenated pyrroles **2b**–**g**.

Conditions	Product (Yield)
SOCl_2_ (1.2 equiv.), CH_2_Cl_2_, 0 °C to r.t., 18 h	R_1_ = H, R_2_ = Cl **2b** (63%)
SOCl_2_ (5 equiv.), CH_2_Cl_2_, r.t., 4.5 d	R_1_ = R_2_ = Cl **2c** (85%)
Br_2_ (1 equiv.), CH_2_Cl_2_, 0 °C to r.t., 10 min	R_1_ = H, R_2_ = Br **2d** (45%)
Br_2_ (2 equiv.), AcOH, r.t. to 60 °C, 2 h	R_1_ = R_2_ = Br **2e** (91%)
ICl (1 equiv.), CH_2_Cl_2_, r.t., 2 h	R_1_ = H, R_2_ = I **2f** (64%)
Silver trifluoro acetate (2 equiv.), I_2_ (2 equiv.), CH_2_Cl_2_, r.t., 36 h	R_1_ = R_2_ = Br **2g** (43%)

**Table 2:** Antibacterial activity of all synthesised analogues in comparison to standard antibiotics.

Compound	MIC (µM)	
	*S. Aureus*	*E. Coli*
Pseudoceratine **1**	8–16	32–64
Streptomycin	0.625–1.25	-
Polymyxin B	-	0.3–0.6
**12a**	>32	>128
**12b**	>32	>64 ^†^
**12c**	>32	>64 ^†^
**12d**	>32	>64 ^†^
**12e**	>32	>64 ^†^
**12f**	>32	>64 ^†^
**13a**	>32	>128
**13b**	>32	>128
**13c**	>32	>64 ^†^
**13d**	>32	>128
**13e**	>32	>64 ^†^
**13f**	>32	>64 ^†^
**14a**	>32	>128
**14b**	>32	>128
**14c**	8–16	64–128
**14d**	>32	>128
**14e**	8–16	32–64
**14f**	>32	>128
**15a**	>32	>128
**15b**	>32	>128
**15c**	4–8	32–64
**15d**	>32	>64
**15e**	4–8	16–32
**15f**	>32	>64
**16a**	>32	>128
**16b**	>32	>128
**16c**	16–32	32–64
**16d**	>32	>128
**16e**	8–16	64–128
**16f**	>32	>128
**17a**	>32	>128
**17b**	>32	>128
**17c**	>32	128–256
**17d**	>32	>128
**17f**	>32	>128
**18a**	>32	>128
**18b**	>32	64–128
**18c**	4–8	32–64
**18d**	16–32	64–128
**18e**	2–4	32–64
**18f**	16–32	64–128
**19a**	>32	>128
**19b**	>32	>128
**19c**	4–8	128–256
**19d**	16–32	64–128
**19e**	2–4	32–64
**19f**	16–32	>128
**27a**	>32	>128
**27b**	>32	>128
**27c**	>32	>128
**27d**	>32	>128
**27e**	>32	>128
**27f**	>32	>128
**28a**	>32	>128
**28b**	>32	>128
**28c**	>32	>128
**28d**	>32	>128
**28e**	>32	>128
**28f**	>32	>128
**20**	>32	>128
**21**	>32	>128
**22**	>32	>128
**23**	>32	>128
**24**	>32	>128

^†^ Starting concentration is lower due to the insolubility of compounds at higher concentrations.

**Table 3:** Cytotoxic activity of **1** and other compounds in Figure 2 against HEK293 human embryonic kidney cells when treated with 16 µM of the stated compound.

Compound	Relative Growth % *
Pseudoceratine **1**	0.7 ± 0.8
**14c**	0.5 ± 0.3
**14e**	1.1 ± 0.9
**15c**	1.8 ± 2.0
**15e**	1.3 ± 0.8
**16c**	2.5 ± 1.8
**16e**	2.6 ± 2.0
**18c**	98.0 ± 4.5
**18e**	2.1 ± 1.5
**19c**	1.6 ± 2.0
**19e**	94.5 ± 5.9

* relative compared to a positive control with no treatment. The values are the average of triplicate experiments ± standard deviation.

## Supplementary Materials

The following are available online. The experimental procedures and characterisation data for all synthesised compounds are provided in the supporting information file.
