# Supplementary material for: Synthesis and Antibacterial Analysis of Analogues of the Marine Alkaloid Pseudoceratidine
Source: Molecules. 2020 Jun 11;25(11):2713. doi: 10.3390/molecules25112713 (PMC7321382; doi:10.3390/molecules25112713)
Supplement: Supplementary file 1 [file molecules-25-02713-s001.pdf]

Supporting Information for:

Synthesis and antibacterial analysis of analogues of the marine alkaloid pseudoceratidine

David Barker,[1,2*] Stephanie Lee,[1] Kyriakos G. Varnava,[1] Kevin Sparrow,[1] Michelle van Rensburg,[1] Rebecca C. Deed,[1,3] Melissa M. Cadelis,[1] Steven A. Li,[1] Brent R. Copp,[1] Vijayalekshmi Sarojini,[1,2] Lisa I. Pilkington[1]

[1] School of Chemical Sciences, University of Auckland, Auckland 1010, New Zealand

[2] MacDiarmid Institute for Advanced Materials and Nanotechnology, Wellington, New Zealand

[3] School of Biological Sciences, University of Auckland, Auckland 1010, New Zealand

*To whom correspondence should be addressed: School of Chemical Sciences, The University of Auckland, Private Bag 92019, Auckland 1142, New Zealand.
E-mail: d.barker@auckland.ac.nz, Tel. 64-9-923-9703

Synthesis and characterisation data of all compounds:

2,2,2-Trichloro-1-(1*H*-pyrrol-2-yl)ethan-1-one 2a

To a stirring solution of trichloroacetyl chloride (21 mL, 0.188 mol) in dry ether (60 mL), under an atmosphere of nitrogen, was added dropwise a solution of pyrrole **3** (13.06 mL, 0.188 mol) in dry ether over 2 h. After 72 h, a solution of potassium carbonate (26 g, 0.188 mol) in water (50 mL) was added carefully. The organic layer was separated, dried ($MgSO_4$), filtered and then stirred with activated charcoal for 1 h. The organic layer was then filtered again and the solvent was removed *in vacuo* to give the crude product, which was recrystallised from *n*-hexanes to afford the *title compound* **2a** (36 g, 89%) as a purple-white metallic solid. m.p. 69–70 °C. (lit. m.p. 73.1–74.9 °C).[1] δ_H (400 MHz, $CDCl_3$) 9.52 (1H, br s, NH), 7.42–7.37 (1H, m, H-5), 7.19–7.15 (1H, m, H-3), 6.42–6.36 (1H, m, H-4). The spectroscopic data was in agreement with literature values.[1,2]

2,2,2-Trichloro-1-(4-chloro-1*H*-pyrrol-2-yl)ethan-1-one 2b

To a stirring solution of pyrrole **2a** (3.4 g, 16.2 mmol) in CH_2Cl_2 (80 mL) in 0 °C was added sulfuryl chloride (1.57 mL, 19.4 mmol) in CH_2Cl_2 (10 mL) dropwise. The reaction was protected by light and stirred at r.t. for 18 h. The reaction mixture was then diluted with CH_2Cl_2 (20 mL), poured onto ice, and quenched with sat. aq. sodium bicarbonate (10 mL). The reaction mixture was extracted with CH_2Cl_2 (3 x 20 mL) and the combined organic extracts washed with brine (50 mL) and dried ($MgSO_4$). The solvent was removed *in vacuo* to give the crude product, which was purified by flash chromatography (8:92 Et_2O:petroleum ether) to afford the *title compound* **2b** (2.5 g, 63%) as a pale cream solid. R_f = 0.17 (8:92 Et_2O:petroleum ether). m.p. 119–121 °C. (lit. m.p. 119–121 °C).[3] δ_H (400 MHz, $CDCl_3$) 9.50 (1H, br s, NH), 7.28 (1H, d, J = 1.5 Hz, H-5), 7.11 (1H, d, J = 1.5 Hz, H-3). The spectroscopic data was in agreement with literature values.[3,4]

2,2,2-Trichloro-1-(4,5-dichloro-1*H*-pyrrol-2-yl)ethan-1-one 2c

The reaction was carried out following a similar procedure to that used for pyrrole **2b** using pyrrole **2a** (2.3 g, 10.7 mmol) and sulfuryl chloride (1.73 mL, 21.4 mmol) in CH_2Cl_2 (6 mL). The reaction was warmed to r.t. and stirred for 18 h, then further sulfuryl chloride (1.73 mL, 21.4 mmol) was added at r.t. and stirred for 18 h. After stirring for a total of 36 h, a final addition of sulfuryl chloride (0.87 mL, 10.7 mmol) was added at r.t. and stirred for 72 hr, before being quenched with sat. aq. sodium bicarbonate (30 mL). The reaction mixture was extracted with CH_2Cl_2 (3 x 20 mL) and the combined extracts washed with brine (50 mL) and dried ($MgSO_4$). The solvent was removed *in vacuo* to give the crude product, which was recrystallised from petroleum ether to afford the *title compound* **2c** (2.55 g, 85%) as a pale pink solid. m.p. 134–136 °C. (lit. m.p. 128–130 °C).[5] δ_H (400 MHz, $CDCl_3$) 9.75 (1H, br s, NH), 7.30 (1H, s, H-3). The spectroscopic data was in agreement with literature values.[5]

1-(4-Bromo-1*H*-pyrrol-2-yl)-2,2,2-trichloroethan-1-one 2d

To a stirring solution of pyrrole **2a** (9.6 g, 0.045 mol) in CH_2Cl_2 (100 mL) at 0 °C, was added dropwise a solution of bromine (2.55 mL, 0.05 mol) in CH_2Cl_2 (20 mL). The mixture was allowed to warm to r.t. and stirred for 10 min before being poured onto water and the organic layer extracted from CH_2Cl_2 (3 x 40 mL). The combined organic extracts were washed with sat. aq. sodium bicarbonate (50 mL), water (50 mL), and dried ($MgSO_4$). The solvent was removed *in vacuo* to give the crude product which was purified by flash chromatography (1:19 EtOAc:petroleum ether) to afford the *title compound* **2d** (6 g, 45%) as a silvery, metallic solid. R_f = 0.17 (1:19 EtOAc:petroleum ether). m.p. 134–136 °C. (lit. m.p. 140–143 °C).[6] δ_H (400 MHz, $CDCl_3$) 9.60 (1H, br s, NH), 7.36 (1H, dd, J = 3.0 and 1.5 Hz, H-5), 7.16 (1H, dd, J = 3.0 and 1.5 Hz, H-3). The spectroscopic data was in agreement with literature values.[7]

2,2,2-Trichloro-1-(4,5-dibromo-1*H*-pyrrol-2-yl)ethan-1-one 2e

To a stirring solution of pyrrole **2a** (5.7 g, 0.027 mol) in glacial acetic acid (30 mL), under an atmosphere of nitrogen, was added a solution of bromine (2.77 mL, 0.054 mol) in acetic acid (30 mL) at such a rate as to maintain the reaction temperature at 18 °C. After the addition the reaction was heated to 60 °C for 2 h before being cooled to r.t and extracted with CH_2Cl_2 (3 x 50 mL). The combined organic extracts were washed with sat. aq. sodium bicarbonate (100 mL), brine (100 mL), and dried ($MgSO_4$). The solvent was removed *in vacuo* to afford the *title compound* **2e** (9.1 g, 91%) as a grey, metallic solid. m.p. 136–138 °C. (lit. m.p. 136–139 °C).[5] δ_H (400 MHz, $CDCl_3$) 9.94 (1H, br s, NH), 7.35 (1H, d, J = 3.0 Hz, H-3). The spectroscopic data was in agreement with literature values.[5]

2,2,2-Trichloro-1-(4-iodo-1*H*-pyrrol-2-yl)ethan-1-one 2f

To a stirring solution of pyrrole **2a** (4.4 g, 20.7 mmol) in CH_2Cl_2 (40 mL), under an atmosphere of nitrogen, was added dropwise a solution of iodine chloride (1.06 mL, 21.1 mmol) in CH_2Cl_2 (20 mL) at r.t. The reaction mixture was stirred for 2 h and 10% sodium carbonate was added to quench the reaction. The organic layer was separated and stirred with sat. aq. sodium thiosulfate (30 mL) for 10 min. The organic layer was separated again and washed with water (50 mL), brine (50 mL), and dried ($MgSO_4$). The solvent was removed *in vacuo* and purified by flash chromatography (8:92 Et_2O:petroleum ether) to afford the *title compound* **2f** (4.5 g, 64%) as a pale tan solid. R_f = 0.14 (8:92 Et_2O:petroleum ether). m.p. 134–136 °C. (lit. m.p. 129–130 °C).[8] δ_H (400 MHz, $CDCl_3$) 9.60 (1H, br s, NH), 7.46 (1H, dd, J = 3.0 and 1.5 Hz, H-5), 7.21 (1H, dd, J = 3.0 and 1.5 Hz, H-3). The spectroscopic data was in agreement with literature values.[8]

2,2,2-Trichloro-1-(4,5-diiodo-1*H*-pyrrol-2-yl)ethan-1-one 2g

To a stirring solution of pyrrole **2a** (0.74 g, 3.5 mmol) and silver trifluoroacetate (1.53 g, 6.9 mmol) in CH_2Cl_2 (6 mL) at 0 °C was added iodine (1.65 g, 6.9 mmol) portionwise. The reaction mixture was stirred at r.t. for 36 h before being filtered and the filtrate washed with sat. aq. sodium thiosulfate (20 mL), brine (20 mL), and dried ($MgSO_4$). The solvent was removed *in vacuo* to give the crude product which then underwent purification by flash chromatography (8:92 Et_2O:petroleum ether) to afford the *title compound* **2g** (0.70 g, 43%) as a pale tan solid. R_f = 0.24 (8:92 Et_2O:petroleum ether). m.p. 179–181 °C. (lit. m.p. 176–178).[5] δ_H (400 MHz, $CDCl_3$) 9.73 (1H, br s, NH), 7.36 (1H, d, *J* = 3.0 Hz, H-3). The spectroscopic data was in agreement with literature values.[5]

_N,N'_-(Butane-1',4'-diyl)bis(1_H_-pyrrole-2-carboxamide) 12a

The reaction was carried out following General Procedure **A** using pyrrole **2a** (319 mg, 1.5 mmol) and amine **4** (0.05 mL, 0.5 mmol) in THF (2 mL), stirring for 48 h to give the crude product. The crude product was then dissolved in ethyl acetate (10 mL) and washed with 2 M HCl (3 mL). The organic layer was separated and washed with 1 M NaOH (10 mL), dried ($MgSO_4$), and the solvent removed *in vacuo* to afford the *title compound* **12a** (77 mg, 57%) as a white, powdery solid. m.p. 215–216 °C. ν_{max} (ATR)/cm^{-1} 3217 (NH amine), 3088 (CH aromatic), 2947 (CH aliphatic), 1605 (C=O amide), 1559 (NH amide), 1525 (C=C aromatic), 1329 (CN aryl), 1093 (CN aliphatic). δ_H (400 MHz, $(CD_3)_2SO$) 11.35 (2H, br s, pyrrole NH), 7.95 (2H, t, *J* = 5.8 Hz, amide NH), 6.83–6.80 (2H, m, H-5), 6.75–6.71 (2H, m, H-3), 6.07–6.04 (2H, m, H-4), 3.25–3.19 (4H, m, H-1'), 1.54–1.49 (4H, m, H-2'). δ_C (100 MHz, $(CD_3)_2SO$) 160.6 (C=O), 126.4 (C-2), 121.0 (C-5), 109.6 (C-3), 108.4 (C-4), 38.2 (C-1'), 27.0 (C-2'). *m/z* (ESI^+): 571 ($[M+Na]^+$, 7%), 297 (100), 204 (7). HRMS (ESI^+): Found $[M+Na]^+$: 297.1324, $C_{14}H_{18}N_4NaO_2$ requires 297.1322.

_N_,_N'_-(Butane-1',4'-diyl)bis(4-chloro-1_H_-pyrrole-2- carboxamide) 12b

The reaction was carried out following General Procedure **A** using pyrrole **2b** (247 mg, 1 mmol) and amine **4** (0.05 mL, 0.5 mmol) in THF (2 mL), stirring for 72 h to give the crude product. The crude product was then dissolved in ethyl acetate (10 mL) and washed with 2 M HCl (3 mL). The organic layer was separated and washed with 1 M NaOH (10 mL), dried ($MgSO_4$), and the solvent removed *in vacuo* to afford the *title compound* **12b** (120 mg, 70%) as a white, powdery solid. m.p. > 230 °C. ν_{max} (ATR)/cm^{-1} 3240 (NH amine), 3120 (CH aromatic), 2948 (CH aliphatic), 1616 (C=O amide), 1562 (NH amide), 1520 (C=C aromatic), 1332 (CN aryl), 1129 (CN aliphatic), 777 (C-Cl). δ_H (400 MHz, $(CD_3)_2SO$) 11.76 (2H, br s, pyrrole NH), 8.11 (2H, t, *J* = 5.8 Hz, amide NH), 7.00–6.97 (2H, m, H-5), 6.84–6.81 (2H, m, H-3), 3.31–3.25 (4H, m, H-1'), 1.59–1.54 (4H, m, H-2'). δ_C (100 MHz, $(CD_3)_2SO$) 159.6 (C=O), 126.1 (C-2), 118.6 (C-5), 110.4 (C-4), 108.8 (C-3), 38.2 (C-1'), 26.8 (C-2'). *m/z* (ESI$^+$): 369 ([$^{37}Cl_2M$+Na]$^+$, 14%), 367 ([$^{37}Cl^{35}ClM$+Na]$^+$, 67), 365 ([$^{35}Cl_2M$+Na]$^+$, 100), 101 (16). HRMS (ESI$^+$): Found [$^{35}Cl_2M$+Na]$^+$: 365.0546, $C_{14}H_{16}{}^{35}Cl_2N_4NaO_2$ requires 365.0543.

_N_,_N'_-(Butane-1',4'-diyl)bis(4,5-dichloro-1_H_-pyrrole-2- carboxamide) 12c

The reaction was carried out following General Procedure **A** using pyrrole **2d** (281 mg, 1 mmol) and amine **4** (0.05 mL, 0.5 mmol) in THF (2 mL), stirring for 48 h to give the crude product. The crude product was then dissolved in ethyl acetate (10 mL) and washed with 2 M HCl (10 mL). A white solid precipitated out of the aqueous layer which was then filtered, dried and collected. The organic layer was also dried ($MgSO_4$), the solvent removed *in vacuo* and combined with the white solid to afford the *title compound* **12c** (162 mg, 79%) as an off-white, powdery solid. m.p. > 230 °C. ν_{max} (ATR)/cm^{-1} 3365 (NH amine), 3122 (CH aromatic), 2952 (CH aliphatic), 1615 (C=O amide), 1562 (NH amide), 1515 (C=C aromatic), 1315 (CN aryl), 1189 (CN aliphatic), 808 (C-Cl). δ_H (400 MHz, $(CD_3)_2SO$) 12.67 (2H, br s, pyrrole NH), 8.13

(2H, t, J = 5.6 Hz, amide NH), 6.87 (2H, d, J = 2.9 Hz, H-3), 3.26–3.17 (4H, m, H-1'), 1.53–1.46 (4H, m, H-2'). δ_C (100 MHz, $(CD_3)_2SO$) 158.9 (C=O), 125.0 (C-2), 114.6 (C-5), 109.4 (C-3), 107.9 (C-4), 38.3 (C-1'), 26.7 (C-2'). *m/z* (ESI^+): 439 ([$^{37}Cl_3{}^{35}Cl$M+Na] $^+$, 13%), 437 ([$^{37}Cl_2{}^{35}Cl_2$M+Na] $^+$, 50), 435 ([$^{37}Cl^{35}Cl_3$M+Na] $^+$, 100), 433 ([$^{35}Cl_4$M+Na] $^+$, 83), 358 (15), 314 (10), 227 (10), 159 (7), 101 (40). HRMS (ESI^+): Found [^{37}Cl2$^{35}Cl_2$M+Na] $^+$: 436.9704, $C_{14}H_{14}{}^{37}$Cl2$^{35}Cl_2N_4NaO_2$ requires 436.9707. Found [$^{37}Cl^{35}Cl_3$M+Na] $^+$: 434.9734, $C_{14}H_{14}{}^{37}Cl^{35}Cl_3N_4NaO_2$ requires 434.9735. Found [$^{35}Cl_4$M+Na^+]: 432.9761, $C_{14}H_{14}{}^{35}Cl_4N_4NaO_2$ requires 432.9763.

N,N'-(Butane-1',4'-diyl)bis(4-bromo-1H-pyrrole-2- carboxamide) 12d

The reaction was carried out following General Procedure **A** using pyrrole **2d** (291 mg, 1 mmol) and amine **4** (0.05 mL, 0.5 mmol) in THF (2 mL), stirring for 48 h to give the crude product. The crude product was then dissolved in ethyl acetate (10 mL) and washed with 2 M HCl (3 mL). The organic layer was separated and washed with 1 M NaOH (10 mL), dried ($MgSO_4$), and the solvent removed *in vacuo* to afford the *title compound* **12d** (125 mg, 58%) as an off-white, powdery solid. m.p. > 230 °C. ν_{max} (ATR)/cm^{-1} 3406 (NH amine), 3118 (CH aromatic), 2949 (CH aliphatic), 1611 (C=O amide), 1575 (NH amide), 1524 (C=C aromatic), 1337 (CN aryl), 1119 (CN aliphatic), 703 (C-Br). δ_H (400 MHz, $(CD_3)_2SO$) 11.72 (2H, br s, pyrrole NH), 8.05 (2H, t, J = 5.7 Hz, amide NH), 6.95 (2H, d, J = 1.5 Hz, H-5), 6.82 (2H, d, J = 1.5 Hz, H-3), 3.24–3.19 (4H, m, H-1'), 1.53–1.47 (4H, m, H-2'). δ_C (100 MHz, $(CD_3)_2SO$) 159.5 (C=O), 127.0 (C-2), 121.0 (C-5), 111.2 (C-3), 94.8 (C-4), 38.2 (C-1'), 26.8 (C-2'). *m/z* (ESI^+): 457 ([$^{81}Br_2$M+Na]$^+$, 52%), 455 ([$^{81}Br^{79}Br$M+Na]$^+$, 100), 453 ([$^{79}Br_2$M+Na]$^+$, 52), 381 (5), 288 (4), 227 (7), 159 (4), 101 (14). HRMS (ESI^+): Found [$^{81}Br_2$M+Na] $^+$: 456.9491, $C_{14}H_{16}{}^{81}Br_2N_4NaO_2$ requires 456.9494. Found [$^{81}Br^{79}Br$M+Na] $^+$: 454.9511,

$C_{14}H_{16}{}^{81}Br^{79}BrN_4NaO_2$ requires 454.9512. Found $[^{79}Br_2M+Na]^+$: 452.9529, $C_{14}H_{16}{}^{79}Br_2N_4NaO_2$ requires 452.9532.

***N*,*N'*-(Butane-1',4'-diyl)bis(4,5-dibromo-1*H*-pyrrole-2- carboxamide) 12e**

The reaction was carried out following General Procedure **A** using pyrrole **2e** (370 mg, 1 mmol) and amine **4** (0.05 mL, 0.5 mmol) in THF (2 mL), stirring for 72 h to give the crude product. The crude product was then dissolved in ethyl acetate (10 mL) and washed with 2 M HCl (10 mL). A white solid precipitated out of the aqueous layer which was then filtered, dried and collected. The organic layer was also dried ($MgSO_4$), the solvent removed *in vacuo* and combined with the white solid to afford the *title compound* **12e** (213 mg, 73%) as an off-white, powdery solid. m.p. > 230 °C. ν_{max} (ATR)/cm^{-1} 3427 (NH amine), 3114 (CH aromatic), 2939 (CH aliphatic), 1617 (C=O amide), 1559 (NH amide), 1507 (C=C aromatic), 1313 (CN aryl), 1062 (CN aliphatic). δ_H (400 MHz, $(CD_3)_2SO$) 12.63 (2H, br s, pyrrole NH), 8.09 (2H, t, *J* = 5.7 Hz, amide NH), 6.90 (2H, d, *J* = 2.5 Hz, H-3), 3.25–3.17 (4H, m, H-1'), 1.52–1.47 (4H, m, H-2'). δ_C (100 MHz, $(CD_3)_2SO$) 158.8 (C=O), 128.3 (C-2), 112.4 (C-3), 104.3 (C-5), 97.7 (C-4), 38.2 (C-1'), 26.7 (C-2'). *m/z* (ESI$^+$): 617 ($[^{81}Br_4M+Na]^+$, 20%), 615 ($[^{81}Br_3{}^{79}BrM+Na]^+$, 67), 613 ($[^{81}Br_2{}^{79}Br_2M+Na]^+$, 100), 611 ($[^{81}Br^{79}Br_3M+Na]^+$, 70), 609 ($[^{79}Br_4M+Na]^+$, 21), 381 (31), 101 (53). HRMS (ESI$^+$): Found $[^{81}Br_4M+Na]^+$: 616.7668, $C_{14}H_{14}{}^{81}Br_4N4NaO_2$ requires 616.7668. Found $[^{81}Br_2{}^{79}Br2M+Na]^+$: 612.7704, $C_{14}H_{14}{}^{81}Br_2{}^{79}Br_2N_4NaO_2$ requires 612.7702. Found $[^{81}Br^{79}Br_3M+Na]^+$: 610.7725, $C_{14}H_{14}{}^{81}Br^{79}Br_3N_4NaO_2$ requires 610.7722. Found $[^{79}Br_4M+Na]^+$: 608.7745, $C_{14}H_{14}{}^{79}Br_4N_4NaO_2$ requires 608.7742.

***N*,*N'*-(Butane-1',4'-diyl)bis(4-iodo-1*H*-pyrrole-2-carboxamide) 12f**

The reaction was carried out following General Procedure **A** using pyrrole **2f** (338 mg, 1 mmol) and amine **4** (0.05 mL, 0.5 mmol) in THF (2 mL), stirring for 48 h to give the crude product.

The crude product was then dissolved in ethyl acetate (10 mL) and washed with 2 M HCl (3 mL). The organic layer was separated and washed with 1 M NaOH (10 mL), dried ($MgSO_4$), and the solvent removed *in vacuo* to afford the *title compound* **2.10f** (188 mg, 72%) as an off-white, powdery solid. m.p. > 230 °C. ν_{max} (ATR)/cm^{-1} 3224 (NH amine), 3118 (CH aromatic), 2939 (CH aliphatic), 1612 (C=O amide), 1557 (NH amide), 1512 (C=C aromatic), 1325 (CN aryl), 1133 (CN aliphatic). δ_H (400 MHz, $(CD_3)_2SO$) 11.65 (2H, br s, pyrrole NH), 8.03 (2H, t, *J* = 5.7 Hz, amide NH), 6.95 (2H, d, *J* = 1.5 Hz, H-5), 6.88 (2H, d, *J* = 1.5 Hz, H-3), 3.25–3.16 (4H, m, H-1'), 1.52–1.46 (4H, m, H-2'). δ_C (100 MHz, $(CD_3)_2SO$) 159.3 (C=O), 128.5 (C-2), 125.9 (C-5), 116.0 (C-3), 60.3 (C-4), 38.2 (C-1'), 26.8 (C-2'). *m/z* (ESI$^+$): 549 ([M+Na]$^+$, 100%), 526 ([M+H]$^+$, 9), 423 (30), 381 (13), 101 (24). HRMS (ESI$^+$): Found [M+Na]$^+$: 548.9258, $C_{14}H_{16}I_2N_4NaO_2$ requires 548.9255. Found [M+H]$^+$: 526.9433, $C_{14}H_{17}I_2N_4O_2$ requires 526.9435.

_N,N'_-(Azanediylbis(ethane-2',1'-diyl))bis(1_H_-pyrrole-2- carboxamide) 13a and _N_-(2-((2-Aminoethyl)amino)ethyl)-1_H_-pyrrole- 2-carboxamide 20

The reaction was carried out following General Procedure **A** using pyrrole **2a** (212 mg, 1 mmol) and amine **14** (0.05 mL, 0.5 mmol) in THF (2 mL), stirring for 48 h to give the crude product, which was purified by flash chromatography (50:5:45 MeOH: NH_4OH, CH_2Cl_2) to afford the *title compound* **13a** (58 mg, 40%) as an off-white, powdery solid. R_f = 0.29 (50:5:45 MeOH: NH_4OH, CH_2Cl_2). m.p. 191–193 °C. ν_{max} (ATR)/cm^{-1} 3284 (NH amine), 3117 (CH aromatic), 2934 (CH aliphatic), 1612 (C=O amide), 1563 (NH amide), 1527 (C=C aromatic), 1330 (CN aryl), 1031 (CN aliphatic). δ_H (400 MHz, $(CD_3)_2SO$) 11.38 (2H, br s, pyrrole NH), 7.91 (2H, t, *J* = 5.7 Hz, amide NH), 6.85–6.80 (2H, m, H-5), 6.76–6.70 (2H, m, H-3), 6.09–6.03 (2H, m, H-4), 3.28 (4H, dt, *J* = 6.0 and 6.8 Hz, H-1'), 2.67 (4H, t, *J* = 6.8 Hz, H-2'). δ_C (100 MHz, $(CD_3)_2SO$) 160.7 (C=O), 126.4 (C-2), 121.1 (C-5), 109.7 (C-3), 108.4 (C-4), 48.8 (C-2'), 38.8

(C-1'). *m/z* (ESI⁻): 288 ([M−H]⁻, 100%), 221 (41). HRMS (ESI⁻): Found [M−H]⁻: 288.1462, $C_{14}H_{18}N_5O_2$ requires 288.1466.

In a separate fraction, *N-(2-((2-aminoethyl)amino)ethyl)-1H-pyrrole-2-carboxamide* **20** (48 mg, 49%) was also isolated, as a yellow oil. R_f = 0.06 (50:5:45 MeOH: NH_4OH: CH_2Cl_2). ν_{max} (ATR)/cm^{-1} 2778 (CH aromatic), 1542 (NH amide), 1403 (C=C aromatic), 1340 (CN aryl), 1013 (CN aliphatic). δ_H (400 MHz, $(CD_3)_2SO$) 11.82 (1H, br s, pyrrole NH), 8.34 (1H, t, *J* = 5.5 Hz, amide NH), 6.85–6.81 (1H, m, H-5), 6.76–6.73 (1H, m, H-3), 6.07–6.03 (1H, m, H-4), 3.29 (2H, dt, *J* = 6.0 and 6.3 Hz, H-1'), 2.83–2.76 (2H, m, H-2''), 2.76–2.69 (2H, m, H-1''), 2.66 (2H, t, *J* = 6.3 Hz, H-2'). δ_C (100 MHz, $(CD_3)_2SO$) 160.8 (C=O), 126.6 (C-2), 121.1 (C-5), 110.5 (C-3), 108.4 (C-4), 48.3 (C-2'), 47.0 (C-1''), 38.7 (C-2''), 38.4 (C-1'). *m/z* (ESI⁺): 219 ([M+Na]⁺, 7%), 101 (100). HRMS (ESI⁺): Found [M+Na]⁺: 219.1221, $C_9H_{16}N_4NaO$ requires 219.1216.

_N,N'_-(Azanediylbis(ethane-2',1'-diyl))bis(4-chloro-1_H_-pyrrole- 2-carboxamide) 13b

The reaction was carried out following General Procedure **A** using pyrrole **2b** (247 mg, 1 mmol) and amine **5** (0.05 mL, 0.5 mmol) in THF (2 mL), stirring for 18 h to give the crude product. The crude product was then dissolved in MeOH (10 mL). A white solid precipitated out which was then filtered, dried and collected to afford the *title compound* **13b** (62 mg, 35%) as a white, powdery solid. m.p. 199–201 °C. ν_{max} (ATR)/cm^{-1} 3287 (NH amine), 3112 (CH aromatic), 2935 (CH aliphatic), 1626 (C=O amide), 1568 (NH amide), 1532 (C=C aromatic), 1328 (CN aryl), 1046 (CN aliphatic), 663 (C-Cl). δ_H (400 MHz, $(CD_3)_2SO$) 11.68 (2H, br s, pyrrole NH), 8.01 (2H, t, *J* = 5.7 Hz, amide NH), 6.93 (2H, d, *J* = 1.5 Hz, H-3), 6.77 (2H, d, *J* = 1.5 Hz, H-5), 3.27 (4H, dt, *J* = 6.0 and 6.5 Hz, H-1'), 2.65 (4H, t, *J* = 6.5 Hz, H-2'). δ_C (100 MHz, $(CD_3)_2SO$) 159.8 (C=O), 126.1 (C-2), 118.6 (C-5), 110.5 (C-4), 108.9 (C-3), 48.6 (C-2'), 38.9 (C-1'). *m/z* (ESI⁻): 360 ([$^{37}Cl_2$M−H]⁻, 15%), 358 ([$^{37}Cl^{35}Cl$M−H]⁻, 67), 356

([$^{35}Cl_2M-H]^-$, 100), 311 (7), 255 (23), 113 (8). HRMS (ESI$^-$): Found [$^{37}Cl^{35}ClM-H]^-$: 358.0655, $C_{14}H_{16}{}^{37}Cl^{35}ClN_5O_2$ requires 358.0659.

_N_,_N'_-(Azanediylbis(ethane-2',1'-diyl))bis(4,5-dichloro-1_H_-pyrrole-2-carboxamide) 13c

The reaction was carried out following General Procedure **A** using pyrrole **2c** (281 mg, 1 mmol) and amine **5** (0.05 mL, 0.5 mmol) in THF (2 mL), stirring for 18 h to give the crude product. The crude product was then triturated in CH_2Cl_2 (20 mL), followed by recrystallisation in EtOH (3 mL) to afford the *title compound* **13c** (25 mg, 12%) as a white, powdery solid. m.p. > 230 °C. ν_{max} (ATR)/cm^{-1} 3282 (NH amine), 3116 (CH aromatic), 2996 (CH aliphatic), 1603 (C=O amide), 1576 (NH amide), 1540 (C=C aromatic), 1329 (CN aryl), 1048 (CN aliphatic), 684 (C-Cl). δ_H (400 MHz, $(CD_3)_2SO$) 8.02 (2H, t, *J* = 5.7 Hz, amide NH), 6.83 (2H, s, H-3), 3.28 (4H, dt, *J* = 6.0 and 6.5 Hz, H-2'), 2.68 (4H, t, *J* = 6.5 Hz, H-1'). δ_C (100 MHz, $(CD_3)_2SO$) 159.5 (C=O), 125.3 (C-2), 115.1 (C-5), 109.5 (C-3), 107.5 (C-4), 48.4 (C-2'), 38.6 (C-1'). *m/z* (ESI$^-$): 432 ([$^{37}Cl_4M-H]^-$, 1%), 430 ([$^{37}Cl_3{}^{35}ClM-H]^-$, 11), 428 ([$^{37}Cl_2{}^{35}Cl_2M-H]^-$, 50), 426 ([$^{37}Cl^{35}Cl_3M-H]^-$, 100), 424 ([$^{35}Cl_4M-H]^-$, 78), 356 (9), 289 (22), 263 (20). HRMS (ESI$^-$): Found [$^{37}Cl_4M-H]^-$: 431.9845, $C_{14}H_{14}{}^{37}Cl_4N_5O_2$ requires 431.9794. Found [$^{37}Cl_3{}^{35}ClM-H]^-$: 429.9825, $C_{14}H_{14}{}^{37}Cl_3{}^{35}ClN_5O_2$ requires 429.9825. Found [$^{37}Cl_2{}^{35}Cl_2M-H]^-$: 427.9847, $C_{14}H_{14}{}^{37}Cl_2{}^{35}Cl_2N_5O_2$ requires 427.9851. Found [$^{37}Cl^{35}Cl_3M-H]^-$: 425.9874, $C_{14}H_{14}{}^{37}Cl^{35}Cl_3N_5O_2$ requires 425.9879. Found [$^{35}Cl_4M-H]^-$: 423.9905, $C_{14}H_{14}{}^{35}Cl_4N_5O_2$ requires 423.9907.

_N_,_N'_-(Azanediylbis(ethane-2',1'-diyl))bis(4-bromo-1_H_-pyrrole- 2-carboxamide) 13d

The reaction was carried out following General Procedure **A** using pyrrole **2d** (291 mg, 1 mmol) and amine **5** (0.05 mL, 0.5 mmol) in THF (2 mL), stirring for 18 h to give the crude

product. The crude product was then triturated in CH_2Cl_2 (20 mL), followed by recrystallisation in EtOH (3 mL) to afford the *title compound* **13d** (43 mg, 19%) as a white, powdery solid. m.p. 201–203 °C. ν_{max} (ATR)/cm^{-1} 3288 (NH amine), 3110 (CH aromatic), 2937 (CH aliphatic), 1623 (C=O amide), 1564 (NH amide), 1521 (C=C aromatic), 1326 (CN aryl), 1108 (CN aliphatic), 660 (C-Br). δ_H (400 MHz, $(CD_3)_2SO$) 11.76 (2H, br s, pyrrole NH), 8.02 (2H, t, *J* = 5.8 Hz, amide NH), 6.95 (2H, d, *J* = 1.5 Hz, H-5), 6.82 (2H, d, *J* = 1.5 Hz, H-3), 3.27 (4H, dt, *J* = 6.0 and 6.5 Hz, H-2'), 2.65 (4H, t, *J* = 6.5 Hz, H-1'). δ_C (100 MHz, $(CD_3)_2SO$) 159.6 (C=O), 127.0 (C-2), 121.0 (C-5), 111.3 (C-3), 94.8 (C-4), 48.5 (C-2'), [obscured by solvent peak] (C-1'). *m/z* (ESI$^-$): 448 ([$^{81}Br_2$M−H]$^-$, 50%), 446 ([$^{81}Br^{79}Br$M−H]$^-$, 100), 444 ([$^{79}Br_2$M−H]$^-$, 50%). HRMS (ESI$^-$): Found [$^{81}Br_2$M−H]$^-$: 447.9638, $C_{14}H_{16}{}^{81}Br_2N_5O_2$ requires 447.9638. Found [$^{81}Br^{79}Br$M−H]$^-$: 445.9658, $C_{14}H_{16}{}^{81}Br^{79}BrN_5O_2$ requires 445.9656. Found [$^{79}Br_2$M−H]$^-$: 443.9675, $C_{14}H_{16}{}^{79}Br_2N_5O_2$ requires 443.9676.

***N*,*N'*-(Azanediylbis(ethane-2',1'-diyl))bis(4,5-dibromo-1*H*-pyrrole-2-carboxamide) 13e**

The reaction was carried out following General Procedure **A** using pyrrole **2e** (370 mg, 1 mmol) and amine **5** (0.05 mL, 0.5 mmol) in THF (2 mL), stirring for 18 h to give the crude product. The crude product was then triturated in CH_2Cl_2 (20 mL), followed by recrystallisation in EtOH (3 mL) to afford the *title compound* **13e** (38 mg, 13%) as a white, powdery solid. m.p. > 230 °C. ν_{max} (ATR)/cm^{-1} 3288 (NH amine), 3110 (CH aromatic), 2929 (CH aliphatic), 1624 (C=O amide), 1567 (NH amide), 1529 (C=C aromatic), 1326 (CN aryl), 1109 (CN aliphatic), 660 (C-Br). δ_H (400 MHz, $(CD_3)_2SO$) 8.00 (2H, t, *J* = 5.9, amide NH), 6.87 (2H, s, H-3), 3.28 (4H, dt, *J* = 6.0 and 6.5 Hz, H-2'), 2.67 (4H, t, *J* = 6.5 Hz, H-1'). δ_C (100 MHz, $(CD_3)_2SO$) 159.3 (C=O), 128.6 (C-2), 112.5 (C-3), 105.0 (C-5), 97.3 (C-4), 48.4 (C-2'), 38.7 (C-1'). *m/z* (ESI$^-$): 608 ([$^{81}Br_4$M−H]$^-$, 18%), 606 ([$^{81}Br_3{}^{79}Br$M−H]$^-$, 73), 604 ([$^{81}Br_2{}^{79}Br_2$M−H]$^-$, 100), 602 ([$^{81}Br^{79}Br_3$M−H]$^-$, 73), 600 ([$^{79}Br_4$M−H]$^-$, 18), 379 (6), 255 (8). HRMS (ESI$^-$): Found

[$^{81}Br_3{}^{79}BrM-H]^-$: 605.7832, $C_{14}H_{14}{}^{81}Br_3{}^{79}BrN_5O_2$ requires 605.7827. Found [$^{81}Br_2{}^{79}Br_2M-H]^-$: 603.7851, $C_{14}H_{14}{}^{81}Br_2{}^{79}Br_2N_5O_2$ requires 603.7846. Found [$^{81}Br^{79}Br_3M-H]^-$: 601.7867, $C_{14}H_{14}{}^{81}Br^{79}Br_3N_5O_2$ requires 601.7866. Found [$^{79}Br_4M-H]^-$: 599.7883, $C_{14}H_{14}{}^{79}Br_4N_5O_2$ requires 599.7886.

***N*,*N*'-(Azanediylbis(ethane-2',1'-diyl))bis(4-iodo-1*H*-pyrrole-2- carboxamide) 13f**

The reaction was carried out following General Procedure **A** using pyrrole **2f** (338 mg, 1 mmol) and amine **5** (0.05 mL, 0.5 mmol) in THF (2 mL), stirring for 72 h to give the crude product. The crude product was then triturated in CH_2Cl_2 (20 mL), followed by recrystallisation in EtOH (3 mL) to afford the *title compound* **13f** (82 mg, 30%) as a white, powdery solid. m.p. 193–195 °C. ν_{max} (ATR)/cm^{-1} 3276 (NH amine), 3107 (CH aromatic), 2936 (CH aliphatic), 1623 (C=O amide), 1566 (NH amide), 1544 (C=C aromatic), 1324 (CN aryl), 1104 (CN aliphatic), 664 (C-I). δ_H (400 MHz, $(CD_3)_2SO$) 11.74 (2H, br s, pyrrole NH), 7.99 (2H, t, *J* = 5.8 Hz, amide NH), 6.96 (2H, d, *J* = 1.5 Hz, H-5), 6.88 (2H, d, *J* = 1.5 Hz, H-3), 3.26 (4H, dt, *J* = 6.0 and 6.5 Hz, H-2'), 2.65 (4H, t, *J* = 6.5 Hz, H-1'). δ_C (100 MHz, $(CD_3)_2SO$) 159.4 (C=O), 128.4 (C-2), 125.9 (C-3), 116.1 (C-5), 60.3 (C-4), 48.6 (C-2'), [obscured by solvent peak] (C-1'). *m/z* (ESI$^-$): 540 ([M−H]$^-$, 100%), 347 (5). HRMS (ESI-): Found [M−H]$^-$: 539.9397, $C_{14}H_{16}I_2N_5O_2$ requires 539.9399.

***N*,*N*'-(Azanediylbis(propane-3',1'-diyl))bis(1*H*-pyrrole-2- carboxamide) 14a**

The reaction was carried out following General Procedure **A** using pyrrole **2a** (212 mg, 1 mmol) and amine **6** (0.07 mL, 0.5 mmol) in THF (2 mL), stirring for 72 h to give the crude product. The crude product was then dissolved in a minimum amount of MeOH and triturated with water (10 mL). A white solid precipitated out which was filtered, dried and collected to afford the *title compound* **14a** (61 mg, 38%) as a light cream solid. m.p. 165–166 °C. ν_{max} (ATR)/cm^{-1}

3291 (NH amine), 3069 (CH aromatic), 2934 (CH aliphatic), 1620 (C=O amide), 1564 (NH amide), 1526 (C=C aromatic), 1326 (CN aryl), 1102 (CN aliphatic). δ_H (400 MHz, $(CD_3)_2SO$) 11.38 (2H, br s, pyrrole NH), 8.05–7.95 (2H, m, amide NH), 6.85–6.78 (2H, m, H-5), 6.75–6.67 (2H, m, H-3), 6.07–6.03 (2H, m, H-4), 3.28–3.20 (4H, m, H-1'), 2.57–2.46 (4H, m, H-3'), 1.66–1.56 (4H, m, H-2'). δ_C (100 MHz, $(CD_3)_2SO$) 160.6 (C=O), 126.4 (C-2), 121.0 (C-5), 109.5 (C-3), 108.4 (C-4), 47.1 (C-3'), 36.8 (C-1'), 29.8 (C-2'). *m/z* (ESI^-): 316 ($[M-H]^-$, 100%), 249 (34) 165 (5). HRMS (ESI^-): Found $[M-H]^-$: 316.1779, $C_{16}H_{22}N_5O_2$ require 316.1779.

***N,N*'-(Azanediylbis(propane-3',1'-diyl))bis(4-chloro-1*H*-pyrrole-2-carboxamide) 14b**

The reaction was carried out following General Procedure **A** using pyrrole **2b** (247 mg, 1 mmol) and amine **6** (0.07 mL, 0.5 mmol) in THF (2 mL), stirring for 72 h to give the crude product, which was then purified by flash chromatography (20:5:75 MeOH: NH_4OH: CH_2Cl_2) to afford the *title compound* **14b** (85 mg, 44%) as a pale yellow solid. R_f = 0.64 (20:5:75 MeOH: NH_4OH: CH_2Cl_2). m.p. 78–80 °C. ν_{max} (ATR)/cm^{-1} 3130 (NH amine), 2937 (CH aliphatic), 1616 (C=O amide), 1566 (NH amide), 1518 (C=C aromatic), 1322 (CN aryl), 1117 (CN aliphatic), 824 (CH aromatic), 769 (C-Cl). δ_H (400 MHz, $(CD_3)_2SO$) 11.70 (2H, br s, pyrrole NH), 8.10 (2H, t, *J* = 5.6 Hz, amide NH), 6.92 (2H, d, *J* = 1.5 Hz, H-5), 6.75 (2H, d, *J* = 1.5 Hz, H-3), 3.24 (4H, dt, *J* = 6.0 and 7.0 Hz, H-1'), 2.53 (4H, t, *J* = 7.0 Hz, H-3'), 1.61 (4H, quint, *J* = 7.0 Hz, H-2'). δ_C (100 MHz, $(CD_3)_2SO$) 159.7 (C=O), 126.1 (C-2), 118.6 (C-5), 110.4 (C-4), 108.8 (C-3), 46.9 (C-3'), 36.8 (C-1'), 29.5 (C-2'). *m/z* (ESI^-): 388 ($[^{37}Cl_2M-H]^-$, 9%), 386 ($[^{37}Cl^{35}ClM-H]^-$, 59), 384 ($[^{35}Cl_2M-H]^-$, 100), 283 (7), 113 (13). HRMS (ESI^-): Found $[^{35}Cl_2M-H]^-$: 384.0999, $C_{16}H_{20}{}^{35}Cl_2N_5O_2$ requires 384.1000.

***N,N*'-(Azanediylbis(propane-3',1'-diyl))bis(4,5-dichloro-1*H*-pyrrole-2-carboxamide) 14c**

The reaction was carried out following General Procedure **A** using pyrrole **2c** (521 mg, 1.9 mmol) and amine **6** (0.07 mL, 0.5 mmol) in THF (2 mL), stirred at reflux at 50 °C for 18 h to

give the crude product, which was then purified by flash chromatography (20:5:75 MeOH: NH_4OH: CH_2Cl_2) to afford the *title compound* **14c** (66 mg, 29%) as a tan, powdery solid. R_f = 0.58 (20:5:75 MeOH: NH_4OH: CH_2Cl_2). m.p. 189–190 °C. ν_{max} (ATR)/cm^{-1} 3336 (NH amine), 3070 (CH aromatic), 2958 (CH aliphatic), 1617 (C=O amide), 1568 (NH amide), 1520 (C=C aromatic), 1367 (CN aryl), 1019 (CN aliphatic), 738 (C-Cl). δ_H (400 MHz, $(CD_3)_2SO$) 8.03 (2H, t, *J* = 5.4 Hz, amide NH), 6.75 (2H, s, H-3), 3.25 (4H, dt, *J* = 6.0 and 7.0 Hz, H-1'), 2.67 (4H, t, *J* = 7.0 Hz, H-3'), 1.67 (4H, quint, *J* = 7.0 Hz, H-2'). δ_C (100 MHz, $(CD_3)_2SO$) 160.3 (C=O), 125.8 (C-2), 116.1 (C-5), 109.4 (C-3), 106.8 (C-4), 45.9 (C-3'), 36.2 (C-1'), 28.3 (C-2'). *m/z* (ESI$^-$): 460 ([$^{37}Cl_4M-H]^-$, 3%), 458 ([$^{37}Cl_3{}^{35}ClM-H]^-$, 13), 456 ([$^{37}Cl_2{}^{35}Cl_2M-H]^-$, 50), 454 ([$^{37}Cl^{35}Cl_3M-H]^-$, 100), 452 ([$^{35}Cl_4M-H]^-$, 78), 317 (9). HRMS (ESI$^-$): Found [$^{37}Cl_4M-H]^-$: 460.0134, $C_{16}H_{18}{}^{37}Cl_4N_5O_2$ requires 460.0109. Found [$^{37}Cl_3{}^{35}ClM-H]^-$:458.0139, $C_{16}H_{18}{}^{37}Cl_3{}^{35}ClN_5O_2$ requires 458.0139.

***N*,*N'*-(Azanediylbis(propane-3',1'-diyl))bis(4-bromo-1*H*-pyrrole-2-carboxamide) 14d**

The reaction was carried out following General Procedure **A** using pyrrole **2d** (291 mg, 1 mmol) and amine **6** (0.07 mL, 0.5 mmol) in THF (2 mL), stirring for 72 h to give the crude product, which was then purified by flash chromatography (20:5:75 MeOH: NH_4OH: CH_2Cl_2) to afford the *title compound* **14d** (61 mg, 26%) as a light yellow solid. R_f = 0.66 (20:5:75 MeOH: NH_4OH: CH_2Cl_2). m.p. 73–75 °C. ν_{max} (ATR)/cm^{-1} 3124 (NH amine), 2928 (CH aliphatic), 1618 (C=O amide), 1562 (NH amide), 1516 (C=C aromatic), 1319 (CN aryl), 1125 (CN aliphatic), 769 (CH aromatic), 734 (C-Br). δ_H (400 MHz, $(CD_3)_2SO$) 11.75 (2H, br s, pyrrole NH), 8.09 (2H, t, *J* = 5.5 Hz, amide NH), 6.95 (2H, d, *J* = 1.5 Hz, H-5), 6.81 (2H, d, *J* = 1.5 Hz, H-3), 3.24 (4H, dt, *J* = 6.0 and 6.7 Hz, H-1'), 2.52 (4H, t, *J* = 6.7 Hz, H-3'), 1.61 (4H, quint, *J* = 6.7 Hz, H-2'). δ_C (100 MHz, $(CD_3)_2SO$) 159.5 (C=O), 127.0 (C-2), 120.9 (C-5), 111.1 (C-3), 94.8 (C-4), 46.9 (C-3'), 36.9 (C-1'), 29.6 (C-2'). *m/z* (ESI−): 476 ([$^{81}Br_2M-H]^-$, 52%),

474 ([^{81}Br^{79}BrM−H]$^-$, 100), 472 ([^{79}Br$_2$M−H]$^-$, 52), 255 (7). HRMS (ESI$^-$): Found [^{81}Br$_2$M−H]$^-$: 475.9956, $C_{16}H_{20}{}^{81}Br_2N_5O_2$ requires 475.9951.

_N_,_N'_-(Azanediylbis(propane-3',1'-diyl))bis(4,5-dibromo-1_H_-pyrrole-2-carboxamide) 14e

The reaction was carried out following General Procedure **A** using pyrrole **2e** (741 mg, 2 mmol) and amine **6** (0.07 mL, 0.5 mmol) in THF (2 mL), stirred at reflux at 50 °C for 18 h to give the crude product. The crude product was then dissolved in a minimum amount of MeOH and triturated with water (10 mL). The resulting precipitate was then further purified by flash chromatography (20:5:75 MeOH: NH_4OH: CH_2Cl_2), to afford the *title compound* **14e** (272 mg, 86%) as a tan, powdery solid. R_f = 0.74 (20:5:75 MeOH: NH_4OH: CH_2Cl_2). m.p. 135–137 °C. δ_H (400 MHz, $(CD_3)_2SO$) 8.06 (2H, t, *J* = 5.7 Hz, amide NH), 6.82 (2H, s, H-3), 3.25 (4H, dt, *J* = 6.0 and 6.8 Hz, H-1'), 2.68 (4H, t, *J* = 6.8 Hz, H-3'), 1.67 (4H, quint, *J* = 6.8 Hz, H-2'). The spectroscopic data was in agreement with literature values.[9]

_N_,_N'_-(Azanediylbis(propane-3',1'-diyl))bis(4-iodo-1_H_-pyrrole- 2-carboxamide) 14f

The reaction was carried out following General Procedure **A** using pyrrole **2f** (338 mg, 1 mmol) and amine **6** (0.07 mL, 0.5 mmol) in THF (2 mL), stirring for 18 h to give the crude product, which was then purified by flash chromatography (20:5:75 MeOH: NH_4OH: CH_2Cl_2) to afford the *title compound* **14f** (117 mg, 41%) as a pale yellow solid. R_f = 0.70 (20:5:75 MeOH: NH_4OH: CH_2Cl_2). m.p. 93–95 °C. ν_{max} (ATR)/cm^{-1} 3336 (NH amine), 3070 (CH aromatic), 2958 (CH aliphatic), 1617 (C=O amide), 1568 (NH amide), 1520 (C=C aromatic), 1367 (CN aryl), 1019 (CN aliphatic). δ_H (400 MHz, $(CD_3)_2SO$) 11.73 (2H, br s, pyrrole NH), 8.08 (2H, t, *J* = 5.8 Hz, amide NH), 6.96 (2H, d, *J* = 1.5 Hz, H-5), 6.87 (2H, d, *J* = 1.5 Hz, H-3), 3.24 (4H, dt, *J* = 6.0 and 7.0 Hz, H-1'), 2.53 (4H, t, *J* = 7.0 Hz, H-3'), 1.61 (4H, quint, *J* = 7.0 Hz, H-2'). δ_C (100 MHz, $(CD_3)_2SO$) 159.3 (C=O), 128.5 (C-2), 125.9 (C-5), 116.0 (C-3), 60.3 (C-4), 46.9

(C-3'), 36.8 (C-1'), 29.5 (C-2'). *m/z* (ESI⁻): 568 ([M−H]⁻, 100%). HRMS (ESI⁻): Found [M−H]⁻: 567.9709, $C_{16}H_{20}I_2N_5O_2$ requires 567.9712.

_N_,_N'_-(Azanediylbis(hexane-6',1'-diyl))bis(1_H_-pyrrole-2- carboxamide) 15a

The reaction was carried out following General Procedure **A** using pyrrole **2a** (212 mg, 1 mmol) and amine **7** (108 mg, 0.5 mmol) in THF (2 mL), stirring for 18 h to give the crude product. The crude product was then triturated with CH_2Cl_2 (20 mL), and the white solid was filtered, dried and collected to afford the *title compound* **15a** (56 mg, 28%) as a white, powdery solid. m.p. 129–131 °C. ν_{max} (ATR)/cm^{-1} 3248 (NH amine), 2929 (CH aliphatic), 1609 (C=O amide), 1562 (NH amide), 1522 (C=C aromatic), 1324 (CN aryl), 1114 (CN aliphatic), 821 (CH aromatic). δ_H (400 MHz, $(CD_3)_2SO$) 11.35 (2H, br s, pyrrole NH), 7.90 (2H, t, *J* = 5.6 Hz, amide NH), 6.83–6.79 (2H, m, H-5), 6.74–6.71 (2H, m, H-3), 6.07–6.03 (2H, m, H-4), 3.18 (4H, dt, *J* = 6.5 and 6.7 Hz, H-1'), 2.46 (4H, t, *J* = 7.0 Hz, H-6'), 1.51–1.43 (4H, m, H-2'), 1.42–1.33 (4H, m, H-5'), 1.33–1.23 (8H, m, H-3' and H-4'). δ_C (100 MHz, $(CD_3)_2SO$) 160.5 (C=O), 126.5 (C-2), 120.9 (C-5), 109.5 (C-3), 108.4 (C-4), 49.4 (C-6'), 38.4 (C-1'), 29.5 (C-2' and C-5'), 26.7 (C-3' or C-4'), 26.5 (C-3' or C-4'). *m/z* (ESI⁻): 400 ([M−H]⁻, 100%), 357 (19), 269 (8). HRMS (ESI⁻): Found [M−H]⁻: 400.2720, $C_{22}H_{34}N_5O_2$ requires 400.2718.

_N_,_N'_-(Azanediylbis(hexane-6',1'-diyl))bis(4-chloro-1_H_-pyrrole- 2-carboxamide) 15b

The reaction was carried out following General Procedure **A** using pyrrole **2b** (247 mg, 1 mmol) and amine **7** (108 mg, 0.5 mmol) in THF (2 mL), stirring for 18 h to give the crude product. The crude product was then triturated with CH_2Cl_2 (20 mL), and the white solid was filtered, dried and collected to afford the *title compound* **15b** (197 mg, 84%) as an off-white, powdery solid. m.p. 106–108 °C. ν_{max} (ATR)/cm^{-1} 3238 (NH amine), 2929 (CH aliphatic), 1626 (C=O amide), 1569 (NH amide), 1526 (C=C aromatic), 1329 (CN aryl), 1117 (CN aliphatic),

824 (CH aromatic), 606 (C-Cl). δ_H (400 MHz, $(CD_3)_2SO$) 8.03 (2H, t, *J* = 5.9 Hz, amide NH), 6.92 (2H, d, *J* = 1.5 Hz, H-5), 6.76 (2H, d, *J* = 1.5 Hz, H-3), 3.18 (4H, dt, *J* = 6.4 and 6.7 Hz, H-1'), 2.52–2.46 (4H, m, H-6'), 1.51–1.43 (4H, m, H-2'), 1.42–1.34 (4H, m, H-5'), 1.33–1.22 (8H, m, H-3' and H-4'). δ_C (100 MHz, $(CD_3)_2SO$) 159.6 (C=O), 126.1 (C-2), 118.5 (C-5), 110.4 (C-4), 108.8 (C-3), 49.1 (C-6'), 38.4 (C-1'), 29.3 (C-2' or C-5'), 29.1 (C-2' or C-5'), 26.5 (C-3' or C-4'), 26.4 (C-3' or C-4'). *m/z* (ESI−): 472 ($[^{37}Cl_2M-H]^-$, 7%), 470 ($[^{37}Cl^{35}ClM-H]^-$, 30), 468 ($[^{35}Cl_2M-H]^-$, 44), 387 (22), 313 (72), 269 (15), 239 (100), 165 (63) 91 (7). HRMS (ESI^-):. Found $[^{37}Cl^{35}ClM-H]^-$: 470.1918, $C_{22}H_{32}{}^{37}Cl^{35}ClN_5O_2$ requires 470.1914. Found $[^{35}Cl_2M-H]^-$: 468.1943, $C_{22}H_{32}{}^{35}Cl_2N_5O_2$ requires 468.1939.

***N,N'*-(Azanediylbis(hexane-6',1'-diyl))bis(4,5-dichloro-1*H*-pyrrole-2-carboxamide) 15c**

The reaction was carried out following General Procedure **A** using pyrrole **2c** (281 mg, 1 mmol) and amine **7** (108 mg, 0.5 mmol) in THF (2 mL), stirring for 72 h to give the crude product. The crude product was then triturated with CH_2Cl_2 (20 mL), then recrystallised with EtOH (5 mL), to afford the *title compound* **15c** (109 mg, 40%) as a pale tan, powdery solid. m.p. 189–190 °C. ν_{max} (ATR)/cm^{-1} 3101 (NH amine), 2931 (CH aliphatic), 1625 (C=O amide), 1571 (NH amide), 1525 (C=C aromatic), 1331 (CN aryl), 1118 (CN aliphatic), 823 (CH aromatic), 592 (C-Cl). δ_H (400 MHz, $(CD_3)_2SO$) 7.82 (2H, t, *J* = 5.7 Hz, amide NH), 6.72 (2H, s, H-3), 3.17 (4H, dt, *J* = 6.4 and 6.7 Hz, H-1'), 2.66 (4H, t, *J* = 7.5 Hz, H-6'), 1.52–1.38 (8H, m, H-2' and H-5'), 1.35–1.22 (8H, m, H-3' and H-4'). δ_C (100 MHz, $(CD_3)_2SO$) 160.2 (C=O), 126.3 (C-2), 116.2 (C-5), 109.2 (C-3), 106.4 (C-4), 47.9 (C-6'), 38.2 (C-1'), 29.3 (C-2'), 27.3 (C-5'), 26.1 (C-3' and C-4'). *m/z* (ESI^-): 544 ($[^{37}Cl_4M-H]^-$, 2%), 542 ($[^{37}Cl_3{}^{35}ClM-H]^-$, 15), 540 ($[^{37}Cl_2{}^{35}Cl_2M-H]^-$, 54), 538 ($[^{37}Cl^{35}Cl_3M-H]^-$, 100), 536 ($[^{35}Cl_4M-H]^-$, 77), 269 (15). HRMS (ESI−): Found $[^{37}Cl_4M-H]^-$: 544.1070, $C_{22}H_{30}{}^{37}Cl_4N_5O_2$ requires 544.1065. Found $[^{37}Cl_3{}^{35}ClM-H]^-$: 542.1080, $C_{22}H_{30}{}^{37}Cl_3{}^{35}ClN_5O_2$ requires 542.1083. Found

$[^{37}Cl_2{}^{35}Cl_2M-H]^-$: 540.1103, $C_{22}H_{30}{}^{37}Cl_2{}^{35}Cl_2N_5O_2$ requires 540.1106. Found $[^{37}Cl^{35}Cl_3M-H]^-$: 538.1127, $C_{22}H_{30}{}^{37}Cl^{35}Cl_3N_5O_2$ requires 538.1132. Found $[^{35}Cl_4M-H]^-$: 536.1155, $C_{22}H_{30}{}^{35}Cl_4N_5O_2$ requires 536.1159.

***N*,*N*'-(Azanediylbis(hexane-6',1'-diyl))bis(4-bromo-1*H*-pyrrole-2-carboxamide) 15d**

The reaction was carried out following General Procedure **A** using pyrrole **2d** (291 mg, 1 mmol) and amine **7** (108 mg, 0.5 mmol) in THF (2 mL), stirring for 72 h to give the crude product. The crude product was then triturated with CH_2Cl_2 (20 mL), and the solid was filtered, dried and collected to afford the *title compound* **15d** (78 mg, 28%) as a light grey, powdery solid. m.p. 178–180 °C. ν_{max} (ATR)/cm^{-1} 3236 (NH amine), 2929 (CH aliphatic), 1602 (C=O amide), 1564 (NH amide), 1522 (C=C aromatic), 1326 (CN aryl), 1117 (CN aliphatic), 822 (CH aromatic), 602 (C-Br). δ_H (400 MHz, $(CD_3)_2SO$) 8.01 (2H, t, *J* = 5.9 Hz, amide NH), 6.94 (2H, d, *J* = 1.5 Hz, H-5), 6.82 (2H, d, *J* = 1.5 Hz, H-3), 3.18 (4H, dt, *J* = 6.0 and 7.0 Hz, H-1'), 2.45 (4H, t, *J* = 7.0 Hz, H-6'), 1.51–1.42 (4H, m, H-2'), 1.41–1.33 (4H, m, H-5'), 1.32–1.22 (8H, m, H-3' and H-4'). δ_C (100 MHz, $(CD_3)_2SO$) 159.4 (C=O), 127.1 (C-2), 120.9 (C-5), 111.1 (C-3), 94.8 (C-4), 49.4 (C-6'). 38.5 (C-1'), 29.5 (C-2' or C-5'), 29.3 (C-2' or C-5'), 26.6 (C-3' or C-4'), 26.4 (C-3' or C-4'). *m/z* (ESI$^-$): 560 ($[^{81}Br_2M-H]^-$, 54%), 558 ($[^{81}Br^{79}BrM-H]^-$, 100), 556 ($[^{79}Br_2M-H]^-$, 54), 468 (35), 357 (31), 269 (12). HRMS (ESI$^-$): Found $[^{81}Br_2M-H]^-$: 560.08986, $C_{22}H_{32}{}^{81}Br_2N_5O_2$ requires 560.0893.

***N*,*N*'-(Azanediylbis(hexane-6',1'-diyl))bis(4,5-dibromo-1*H*-pyrrole-2-carboxamide) 15e**

The reaction was carried out following General Procedure **A** using pyrrole **2e** (370 mg, 1 mmol) and amine **7** (108 mg, 0.5 mmol) in THF (2 mL), stirring for 18 h to give the crude product.

The crude product was then triturated with CH_2Cl_2 (20 mL), and the solid was filtered, dried and collected to afford the *title compound* **15e** (281 mg, 78%) as an off-white, powdery solid. m.p. 196–198 °C. δ_H (400 MHz, $(CD_3)_2SO$) 7.86 (2H, t, *J* = 5.8 Hz, amide NH), 6.78 (2H, s, H-3), 3.17 (4H, dt, *J* = 6.5 and 6.8 Hz, H-1'), 2.65 (4H, t, *J* = 7.2 Hz, H-6'), 1.51–1.40 (8H, m, H-2' and H-5'), 1.34–1.22 (8H, m, H-3' and H-4'). The spectroscopic data was in agreement with literature values.[7]

N,N'-(Azanediylbis(hexane-6',1'-diyl))bis(4-iodo-1*H*-pyrrole-2- carboxamide) 15f

The reaction was carried out following General Procedure **A** using pyrrole **2f** (338 mg, 1 mmol) and amine **7** (108 mg, 0.5 mmol) in THF (2 mL), stirring for 18 h to give the crude product. The crude product was then triturated with CH_2Cl_2 (20 mL), and the solid was filtered, dried and collected, to afford the *title compound* **15f** (142 mg, 43%) as a light grey, powdery solid. m.p. 174–176 °C. ν_{max} (ATR)/cm^{-1} 3236 (NH amine), 3116 (CH aromatic), 2927 (CH aliphatic), 1599 (C=O amide), 1570 (NH amide), 1523 (C=C aromatic), 1328 (CN aryl), 1120 (CN aliphatic), 601 (C-I). δ_H (400 MHz, $(CD_3)_2SO$) 7.99 (2H, t, *J* = 5.6 Hz, amide NH), 6.95 (2H, d, *J* = 1.5 Hz, H-5), 6.88 (2H, d, *J* = 1.5 Hz, H-3), 3.17 (4H, dt, *J* = 6.0 and 7.0 Hz, H-1'), 2.44 (4H, t, *J* = 7.0 Hz, H-6'), 1.50–1.41 (4H, m, H- 2'), 1.41–1.32 (4H, m, H-5'), 1.31–1.23 (8H, m, H-3' and H-4'). δ_C (100 MHz, $(CD_3)_2SO$) 159.2 (C=O), 128.5 (C-2), 125.8 (C-5), 115.9 (C-3), 60.3 (C-4), 49.4 (C-1'), 38.4 (C-6'), 29.6 (C-2' or C-5'), 29.3 (C-2' or C-5'), 26.6 (C-3' or C-4'), 26.4 (C-3' or C-4'). *m/z* (ESI$^-$): 652 ([M−H]$^-$, 100%), 325 (12). HRMS (ESI$^-$): Found [M−H]$^-$: 652.0656, $C_{22}H_{32}I_2N_5O_2$ requires 652.0651.

***N*-(3''-((2'''-(1'*H*-pyrrole-2'- carboxamido)ethyl)amino)propyl)-1*H*-pyrrole-2-carboxamide 16a**

The reaction was carried out following General Procedure **A** using pyrrole **2a** (212 mg, 1 mmol) and amine **8** (0.06 mL, 0.5 mmol) in THF (2 mL), stirring for 72 h to give the crude product, which was then purified by flash chromatography (20:5:75 MeOH: NH_4OH: CH_2Cl_2) to afford the *title compound* **16a** (78 mg, 51%) as a pale yellow solid. R_f = 0.61 (20:5:75 MeOH: NH_4OH: CH_2Cl_2). m.p. 69–71 °C. ν_{max} (ATR)/cm^{-1} 3242 (NH amine), 2934 (CH aliphatic), 1613 (C=O amide), 1560 (NH amide), 1519 (C=C aromatic), 1319 (CN aryl), 1128 (CN aliphatic), 836 (CH aromatic). δ_H (400 MHz, $(CD_3)_2SO$) 11.45–11.34 (2H, m, pyrrole NH), 8.02 (1H, t, *J* = 5.7 Hz, amide NH-H-1''), 7.96 (1H, t, *J* = 5.7 Hz, amide NH-H-1'''), 6.85–6.80 (2H, m, H-5 and H-5'), 6.76–6.70 (2H, m, H-3 and H-3'), 6.08–6.04 (2H, m, H-4 and H-4'), 3.38–3.28 (2H, m, H- 1'''), 3.25 (2H, dt, *J* = 6.0 and 7.0 Hz, H-1''), 2.69 (2H, t, *J* = 6.5 Hz, H-2'''), 2.62 (2H, t, *J* = 7.0 Hz, H-3''), 1.65 (2H, quint, *J* = 7.0 Hz, H-2''). δ_C (100 MHz, $(CD_3)_2SO$) 160.8 (C-2-C=O or C-2'-C=O), 160.6 (C-2-C=O or C-2'-C=O), 126.4 (C-2 or C-2'), 126.3 (C-2 or C-2'), 121.1 (C-5 or C-5'), 121.0 (C-5 or C-5'), 109.8 (C-3 or C-3'), 109.6 (C-3 or C-3'), 108.4 (C-4 and C-4'), 48.8 (C-2'''), 46.6 (C-3''), 38.2 (C-1'''), 36.6 (C-1''), 29.3 (C-2''). *m/z* (ESI^-): 302 ($[M-H]^-$, 100%), 235 (18), 113 (9). HRMS (ESI^-): Found $[M-H]^-$: 302.1626, $C_{15}H_{20}N_5O_2$ requires 302.1622.

4-Chloro-*N*-(3''-((2'''-(4'-chloro-1'*H*-pyrrole-2'- carboxamido)ethyl)amino)propyl)-1*H*-pyrrole-2-carboxamide 16b

The reaction was carried out following General Procedure **A** using pyrrole **2b** (247 mg, 1 mmol) and amine **8** (0.06 mL, 0.5 mmol) in THF (2 mL), stirring for 18 h to give the crude product, which was then purified by flash chromatography (20:5:75 MeOH: NH_4OH: CH_2Cl_2) to afford the *title compound* **16b** (89 mg, 48%) as a pale cream solid. R_f = 0.68 (20:5:75 MeOH: NH_4OH: CH_2Cl_2). m.p. 167–169 °C. ν_{max} (ATR)/cm^{-1} 3283 (NH amine), 3116 (CH aromatic), 2934 (CH aliphatic), 1628 (C=O amide), 1567 (NH amide), 1528 (C=C aromatic), 1320 (CN

aryl), 1107 (CN aliphatic), 584 (C-Cl). δ_H (400 MHz, $(CD_3)_2SO$) 11.70 (2H, br s, pyrrole NH), 8.10 (1H, t, *J* = 5.7 Hz, amide NH-H-1''), 8.04 (1H, t, *J* = 5.7 Hz, amide NH-H-1'''), 6.94–6.91 (2H, m, H-5 and H-5'), 6.77 (2H, dd, *J* = 1.5 and 7.0 Hz, H-3 and H-3'), 3.29 (2H, dt, *J* = 6.0 and 7.0 Hz, H-1'''), 3.24 (2H, dt, *J* = 6.0 and 7.0 Hz, H-1''), 2.65 (2H, t, *J* = 6.5 Hz, H-2'''), 2.57 (2H, t, *J* = 7.0 Hz, H-3''), 1.62 (2H, quint, *J* = 7.0 Hz, H-2''). δ_C (100 MHz, $(CD_3)_2SO$) 159.8 (C-2-C=O or C-2'-C=O), 159.7 (C-2-C=O or C-2'-C=O), 126.11 (C-2 or C-2'), 126.05 (C-2 or C-2'), 118.63 (C-5 or C-5'), 118.58 (C-5 and C-5'), 110.4 (C-4 and C-4'), 109.0 (C-3 or C-3'), 108.8 (C-3 or C-3'), 48.7 (C-2'''), 46.6 (C-3''), 38.6 (C-1'''), 36.8 (C-1''), 29.5 (C-2''). *m/z* (ESI^-): 374 ($[^{37}Cl_2M-H]^-$, 8%), 372 ($[^{37}Cl^{35}ClM-H]^-$, 67), 370 ($[^{35}Cl_2M-H]^-$, 100%), 269 (8), 113 (6). HRMS (ESI^-): Found $[^{37}Cl^{35}ClM-H]^-$: 372.0815, $C_{15}H_{18}{}^{37}Cl^{35}ClN_5O_2$ requires 372.0816.

4,5-Dichloro-*N*-(3''-((2'''-(4',5'-dichloro-1'*H*-pyrrole-2'-carboxamido)ethyl)amino)propyl)-1*H*-pyrrole-2-carboxamide 16c

The reaction was carried out following General Procedure **A** using pyrrole **2c** (281 mg, 1 mmol) and amine **8** (0.06 mL, 0.5 mmol) in THF (2 mL), stirring for 72 h to give the crude product, which was then purified by flash chromatography (20:5:75 MeOH: NH_4OH: CH_2Cl_2) to afford the *title compound* **16c** (94 mg, 43%) as a pale cream solid. R_f = 0.66 (20:5:75 MeOH: NH_4OH: CH_2Cl_2). m.p. 133–135 °C. ν_{max} (ATR)/cm^{-1} 3318 (NH amine), 3115 (CH aromatic), 2935 (CH aliphatic), 1617 (C=O amide), 1569 (NH amide), 1525 (C=C aromatic), 1333 (CN aryl), 1017 (CN aliphatic), 590 (C-Cl). δ_H (400 MHz, $(CD_3)_2SO$) 8.11 (1H, t, *J* = 5.6 Hz, amide NH-H-1''), 8.06 (1H, t, *J* = 5.6 Hz, amide NH-H-1'''), 6.80 (2H, d, *J* = 4.0 Hz, H-3 and H-3'), 3.32 (2H, dt, *J* = 6.0 and 6.5 Hz, H-1'''), 3.25 (2H, dt, *J* = 6.0 and 6.5 Hz, H-1''), 2.72 (2H, t, *J* = 6.5 Hz, H-2'''), 2.64 (2H, t, *J* = 6.5 Hz, H-3''), 1.65 (2H, quint, *J* = 6.5 Hz, H-2''). δ_C (100 MHz, $(CD_3)_2SO$) 159.9 (C-2-C=O or C-2'-C=O), 159.7 (C-2-C=O or C-2'-C=O), 125.5 (C-2 or C-2'), 125.4 (C-2 or C-2'), 115.6 (C-5 or C-5'), 115.4 (C-5 or C-5'), 109.6 (C-3 or C-3'), 109.4 (C-3 or C-3'),

107.3 (C-4 or C-4'), 107.1 (C-4 or C-4'), 48.4 (C-2'''), 46.1 (C-3''), 37.9 (C-1'''), 36.5 (C-1''), 28.8 (C-2''). *m/z* (ESI^-): 446 ($[^{37}Cl_4M{-}H]^-$, 2%), 444 ($[^{37}Cl_3{}^{35}ClM{-}H]^-$, 10), 442 ($[^{37}Cl_2{}^{35}Cl_2M{-}H]^-$, 46), 440 ($[^{37}Cl^{35}Cl_3M{-}H]^-$, 100), 438 ($[^{35}Cl_4M{-}H]^-$, 75), 303 (8). HRMS ($ESI^-$): Found $[^{37}Cl_2{}^{35}Cl_2M{-}H]^-$: 442.0010, $C_{15}H_{16}{}^{37}Cl_2{}^{35}Cl_2N_5O_2$ requires 442.0008.

4-Bromo-*N*-(3''-((2'''-(4'-bromo-1'*H*-pyrrole-2'- carboxamido)ethyl)amino)propyl)-1*H*-pyrrole-2-carboxamide 16d and *N*-(2''-((3'-Aminopropyl)amino)ethyl)-4-bromo-1*H*-pyrrole-2- carboxamide 21 and *N*-(3'-((2''-Aminoethyl)amino)propyl)-4- bromo-1*H*-pyrrole-2-carboxamide 22

The reaction was carried out following General Procedure **A** using pyrrole **2d** (291 mg, 1 mmol) and amine **8** (0.06 mL, 0.5 mmol) in THF (2 mL), stirring for 18 h to give the crude product, which was then purified by flash chromatography (20:5:75 MeOH: NH_4OH: CH_2Cl_2) to afford the *title compound* **16d** (82 mg, 36%) as a pale yellow solid. R_f = 0.74 (20:5:75 MeOH: NH_4OH: CH_2Cl_2). m.p. 188–190 °C. ν_{max} (ATR)/cm^{-1} 3278 (NH amine), 3114 (CH aromatic), 2932 (CH aliphatic), 1624 (C=O amide), 1562 (NH amide), 1524 (C=C aromatic), 1318 (CN aryl), 1113 (CN aliphatic), 583 (C-Br). δ_H (400 MHz, $(CD_3)_2SO$) 11.75 (2H, br s, pyrrole NH), 8.10 (1H, t, *J* = 5.6 Hz, amide NH-H-1''), 8.04 (1H, t, *J* = 5.6 Hz, amide NH-H-1'''), 6.97–6.94 (2H, m, H-5 and H-5'), 6.83 (2H, dd, *J* = 6.9 and 1.7 Hz, H-3 and H-3'), 3.29 (2H, dt, *J* = 6.0 and 6.5 Hz, H-1'''), 3.24 (2H, dt, *J* = 6.0 and 6.5 Hz, H-1''), 2.64 (2H, t, *J* = 6.5 Hz, H-2'''), 2.56 (2H, t, *J* = 6.5 Hz, H-3''), 1.61 (2H, quint, *J* = 6.5 Hz, H-2''). δ_C (100 MHz, $(CD_3)_2SO$) 159.7 (C-2-C=O or C-2'-C=O), 159.5 (C-2-C=O or C-2'-C=O), 127.03 (C-2 or C-2'), 126.98 (C-2 or C-2'), 121.01 (C-5 or C-5'), 120.96 (C-5 or C-5'), 111.4 (C-3 or C-3'), 111.2 (C-3 or C-3'), 94.8 (C-4 and C-4'), 48.8 (C-2'''), 46.6 (C-3''), 38.7 (C-1'''), 36.8 (C-1''), 29.6 (C-2''). *m/z* (ESI^-): 462 ($[^{81}Br_2M{-}H]^-$, 55%), 460 ($[^{81}Br^{79}BrM{-}H]^-$, 100), 458 ($[^{79}Br_2M{-}H]^-$, 55), 370 (17), 127 (17). HRMS (ESI^-): Found $[^{81}Br_2M{-}H]^-$: 461.9794, $C_{15}H_{18}{}^{81}Br_2N_5O_2$ requires

461.9795. Found [^{81}Br^{79}BrM−H]$^-$: 459.9813, $C_{15}H_{18}{}^{81}Br^{79}BrN_5O_2$ requires 459.9813. Found [^{79}Br$_2$M−H]$^-$: 457.9828, $C_{15}H_{18}{}^{79}Br_2N_5O_2$ requires 457.9833.

In a separate fraction, *N-(2''-((3'-Aminopropyl)amino)ethyl)-4-bromo-1H-pyrrole-2-carboxamide* **21** (41 mg, 28%) was also isolated, as a pale yellow oil. R_f = 0.26 (20:5:75 MeOH: NH_4OH: CH_2Cl_2). ν_{max} (ATR)/cm^{-1} 3125 (NH amine), 2928 (CH aromatic), 2851 (CH aliphatic), 1623 (C=O amide), 1566 (NH amide), 1524 (C=C aromatic), 1323 (CN aryl), 1123 (CN aliphatic). δ_H (400 MHz, $(CD_3)_2SO$) 8.15–8.06 (1H, m, amide NH), 6.95 (1H, d, *J* = 1.5 Hz, H-5), 6.83 (1H, d, *J* = 1.5 Hz, H-3), 3.27 (2H, dt, *J* = 6.0 and 6.5 Hz, H-1''), 2.66–2.58 (4H, m, H-1' and H-2''), 2.55 (2H, t, *J* = 6.5 Hz, H-3'), 1.50 Hz (2H, quint, *J* = 6.5 Hz, H-2'). δ_C (100 MHz, $(CD_3)_2SO$) 159.6 (C=O), 127.1 (C-2), 121.0 (C-5), 111.4 (C-3), 94.8 (C-4), 48.9 (C-2''), 46.8 (C-3'), 38.8 (C-1''), [obscured by solvent peak] (C-1'), 32.0 (C-2'). *m/z* (ESI$^-$): 289 ([^{81}BrM−H]$^-$, 100%), 287 ([^{79}BrM−H]$^-$, 91), 283 (27), 281 (36), 255 (45). HRMS (ESI$^-$): Found [^{81}BrM−H]$^-$: 289.0493, $C_{10}H_{16}{}^{81}BrN_4O$ requires 289.0493. Found [^{79}BrM−H]$^-$: 287.0508, $C_{10}H_{16}{}^{79}BrN_4O$ requires 287.0513.

In a separate fraction, *N-(3'-((2''-Aminoethyl)amino)propyl)-4-bromo-1H-pyrrole-2-carboxamide* **22** (50 mg, 35%) was also isolated, as a pale yellow oil. R_f = 0.40 (20:5:75 MeOH: NH_4OH: CH_2Cl_2). ν_{max} (ATR)/cm^{-1} 3117 (NH amine), 2937 (CH aromatic), 2854 (CH aliphatic), 1619 (C=O amide), 1567 (NH amide), 1526 (C=C aromatic), 1324 (CN aryl), 1119 (CN aliphatic). δ_H (400 MHz, $(CD_3)_2SO$) 8.15 (1H, t, *J* = 5.7 Hz, amide NH), 6.94 (1H, d, *J* = 1.5 Hz, H-5), 6.81 (1H, d, *J* = 1.5 Hz, H-3), 3.24 (2H, dt, *J* = 6.0 and 6.5 Hz, H-1'), 2.65–2.58 (2H, m, H-1''), 2.56–2.47 (4H, m, H-2'' and H-3'), 1.60 (2H, quint, *J* = 6.5 Hz, H-2'). δ_C (100 MHz, $(CD_3)_2SO$) 159.5 (C=O), 127.1 (C-2), 120.9 (C-5), 111.2 (C-3), 94.8 (C-4), 51.4 (C-2''), 46.7 (C-3'), 40.9 (C-1''), 36.9 (C-1'), 29.6 (C-2'). *m/z* (ESI−): 289 ([^{81}BrM−H]$^-$, 100%), 287 ([^{79}BrM−H]$^-$, 90), 283 (30), 272 (10), 255 (30), 239 (30), 227 (30), 187 (10). HRMS (ESI$^-$): Found [^{81}BrM−H]$^-$: 289.0498, $C_{10}H_{16}{}^{81}BrN_4O$ requires 289.0493.

4,5-Dibromo-*N*-(3''-((2'''-(4',5'-dibromo-1'*H*-pyrrole-2'-carboxamido)ethyl)amino)propyl)-1*H*-pyrrole-2-carboxamide 16e and *N*-(2''-((3'-Aminopropyl)amino)ethyl)-4,5-dibromo-1*H*-pyrrole-2- carboxamide 23 and *N*-(3'-((2''-Aminoethyl)amino)propyl)-4,5- dibromo-1*H*-pyrrole-2-carboxamide 24

The reaction was carried out following General Procedure **A** using pyrrole **2e** (370 mg, 1 mmol) and amine **8** (0.06 mL, 0.5 mmol) in THF (2 mL), stirring for 18 h to give the crude product, which was then purified by flash chromatography (20:5:75 MeOH: NH_4OH: CH_2Cl_2) to afford the *title compound* **16e** (117 mg, 38%) as a pale orange solid. R_f = 0.75 (20:5:75 MeOH: NH_4OH: CH_2Cl_2). m.p. 206–208 °C. ν_{max} (ATR)/cm^{-1} 3274 (NH amine), 3109 (CH aromatic), 2926 (CH aliphatic), 1625 (C=O amide), 1569 (NH amide), 1525 (C=C aromatic), 1323 (CN aryl), 1081 (CN aliphatic), 564 (C-Br). δ_H (400 MHz, $(CD_3)_2SO$) 8.09 (1H, t, *J* = 5.8 Hz, amide NH-H-1''), 8.04 (1H, t, *J* = 5.8 Hz, amide NH-H-1'''), 6.85 (2H, s, H-3 and H-3'), 3.31 (2H, dt, *J* = 6.0 and 6.5 Hz, H-1'''), 3.24 (2H, dt, *J* = 6.0 and 6.5 Hz, H-1''), 2.70 (2H, t, *J* = 6.5 Hz, H-2'''), 2.63 (2H, t, *J* = 7.0 Hz, H-3''), 1.64 (2H, quint, *J* = 7.0 Hz, H-2''). δ_C (100 MHz, $(CD_3)_2SO$) 159.6 (C-2-C=O or C-2'-C=O), 159.4 (C-2-C=O or C-2'-C=O), 128.7 (C-2 or C-2'), 128.6 (C-2 or C-2'), 112.6 (C-3 or C-3'), 112.4 (C-3 or C-3'), 105.3 (C-5 or C-5'), 105.1 (C-5 or C-5'), 97.2 (C-4 or C-4'), 97.1 (C-4 or C-4'), 48.4 (C-2'''), 46.2 (C-3''), 38.0 (C-1'''), 36.5 (C-1''), 29.0 (C-2''). *m/z* (ESI$^-$): 622 ([$^{81}Br_4$M−H]$^-$, 17%), 620 ([$^{81}Br_3{}^{79}Br$M−H]$^-$, 67), 618 ([$^{81}Br_2{}^{79}Br_2$M−H]$^-$, 100), 616 ([$^{81}Br^{79}Br_3$M−H]$^-$, 71), 614 ([$^{79}Br_4$M−H]$^-$, 21), 554 (13), 440 (8), 370 (54). HRMS (ESI−): Found [$^{81}Br_2{}^{79}Br_2$M−H]$^-$: 617.7999, $C_{15}H_{16}{}^{81}Br_2{}^{79}Br_2N_5O_2$ requires 617.8003.

In a separate fraction, *N-(2''-((3'-Aminopropyl)amino)ethyl)-4,5-dibromo-1H-pyrrole-2-carboxamide* **23** (52 mg, 28%) was also isolated, as a pale yellow oil. R_f = 0.23 (20:5:75 MeOH: NH_4OH: CH_2Cl_2). ν_{max} (ATR)/cm^{-1} 3276 (NH amine), 2928 (CH aromatic), 2852 (CH

aliphatic), 1567 (NH amide), 1526 (C=O aromatic), 1345 (CN aryl), 1218 (CN aliphatic), 652 (C-Br). δ_H (400 MHz, $(CD_3)_2SO$) 7.66–7.55 (1H, m, amide NH), 6.57 (1H, s, H-3), 3.24 (2H, dt, J = 6.0 and 6.5 Hz, H-1''), 2.79 (2H, t, J = 6.8 Hz, H-1'), 2.65–2.57 (4H, m, H-2'', H-3'), 1.60 (2H, quint, J = 6.8 Hz, H-2'). δ_C (100 MHz, $(CD_3)_2SO$) 162.2 (C=O), 131.6 (C-2), 112.1 (C-3), 109.1 (C-5), 94.0 (C-4), 49.0 (C-2''), 46.4 (C-3'), 38.5 (C-1'), 38.1 (C-1''), 28.4 (C-2'). *m/z* (ESI^-): 369 ($[^{81}Br_2M{-}H]^-$, 50%), 367 ($[^{81}Br^{79}BrM{-}H]^-$, 100), 365 ($[^{79}Br_2M{-}H]^-$, 50), 289 (8), 113 (72). HRMS (ESI−): Found $[^{81}Br_2M{-}H]^-$: 368.9577, $C_{10}H_{15}{}^{81}Br_2N_4O$ requires 368.9578.

In a separate fraction, *N-(3'-((2''-Aminoethyl)amino)propyl)-4,5-dibromo-1H-pyrrole-2-carboxamide* **24** (44 mg, 24%) was also isolated, as a pale yellow oil. R_f = 0.35 (20:5:75 MeOH: NH_4OH: CH_2Cl_2). ν_{max} (ATR)/cm^{-1} 3282 (NH amine), 2934 (CH aliphatic), 1529 (NH amide), 1321 (CN aryl), 1216 (CN aliphatic), 812 (CH aromatic), 753 (C-Br). δ_H (400 MHz, $(CD_3)_2SO$) 7.97 (1H, t, J = 5.5 Hz, amide NH), 6.56 (1H, s, H-3), 3.24 (2H, dt, J = 6.0 and 6.5 Hz, H-1'), 2.81–2.75 (2H, m, H-1''), 2.69–2.63 (2H, m, H-2''), 2.58 (2H, t, J = 6.5 Hz, H-3'), 1.60 (2H, quint, J = 6.5 Hz, H-2'). δ_C (100 MHz, $(CD_3)_2SO$) 162.1 (C=O), 131.8 (C-2), 112.2 (C-3), 109.2 (C-5), 94.0 (C-4), 48.0 (C-2''), 46.8 (C-3'), [obscured by solvent peak] (C-1''), 37.0 (C-1'), 28.9 (C-2'). *m/z* (ESI^-): 369 ($[^{81}Br_2M{-}H]^-$, 50%), 367 ($[^{81}Br^{79}BrM{-}H]^-$, 100), 365 ($[^{79}Br_2M{-}H]^-$, 50), 287 (5), 113 (60). HRMS (ESI^-): Found $[^{81}Br^{79}BrM{-}H]^-$: 366.9594, $C_{10}H_{15}{}^{81}Br^{79}BrN_4O$ requires 366.9598.

4-Iodo-*N*-(3''-((2'''-(4'-iodo-1'*H*-pyrrole-2'-carboxamido)ethyl)amino)propyl)-1*H*-pyrrole-2-carboxamide 16f

The reaction was carried out following General Procedure **A** using pyrrole **2f** (338 mg, 1 mmol) and amine **8** (0.06 mL, 0.5 mmol) in THF (2 mL), stirring for 18 h to give the crude product,

which was then purified by flash chromatography (20:5:75 MeOH: NH_4OH: CH_2Cl_2) to afford the *title compound* **16f** (86 mg, 31%) as a pale yellow solid. R_f = 0.74 (20:5:75 MeOH: NH_4OH: CH_2Cl_2). m.p. 178–180 °C. ν_{max} (ATR)/cm^{-1} 3350 (NH amine), 3107 (CH aromatic), 2929 (CH aliphatic), 1626 (C=O amide), 1559 (NH amide), 1521 (C=C aromatic), 1312 (CN aryl), 1107 (CN aliphatic), 592 (C-I). δ_H (400 MHz, $(CD_3)_2SO$) 11.70 (2H, br s, pyrrole NH), 8.07 (1H, t, *J* = 5.7 Hz, amide NH-H-1''), 8.01 (1H, t, *J* = 5.7 Hz, amide NH-H-1'''), 6.98–6.94 (2H, m, H-5 and H-5'), 6.88 (2H, dd, *J* = 6.5 and 1.5 Hz, H-3 and H-3'), 3.27 (2H, dt, *J* = 6.0 and 6.5 Hz, H-1'''), 3.24 (2H, dt, *J* = 6.0 and 6.5 Hz, H-1''), 2.63 (2H, t, *J* = 6.5 Hz, H-2'''), 2.55 (2H, t, *J* = 6.5 Hz, H-3''), 1.61 (2H, quint, *J* = 6.5 Hz, H-2''). δ_C (100 MHz, $(CD_3)_2SO$) 159.5 (C-2-C=O or C-2'-C=O), 159.3 (C-2-C=O or C-2'-C=O), 128.5 (C-2 or C-2'), 128.4 (C-2 or C-2'), 125.9 (C-5 and C-5'), 116.2 (C-3 or C-3'), 116.0 (C-3 or C-3'), 60.3 (C-4 and C-4'), 48.8 (C-2'''), 46.6 (C-3''), 38.7 (C-1'''), 36.8 (C-1''), 29.6 (C-2''). *m/z* (ESI^-): 554 ($[M-H]^-$, 100%), 497 (13), 421 (6), 385 (8), 325 (9), 283 (15), 255 (6). HRMS (ESI^-): Found $[M-H]^-$: 553.9556, $C_{15}H_{18}I_2N_5O_2$ requires 553.9555.

N-(3'''-((4''-(1'H-pyrrole-2'-carboxamido)butyl)amino)propyl)-1H-pyrrole-2-carboxamide 17a

The reaction was carried out following General Procedure **A** using pyrrole **2a** (212 mg, 1 mmol) and amine **9** (0.08 mL, 0.5 mmol) in THF (2 mL), stirring for 72 h to give the crude product, which was then purified by flash chromatography (20:5:75 MeOH: NH_4OH: CH_2Cl_2) to afford the *title compound* **17a** (83 mg, 50%) as a light yellow solid. R_f = 0.62 (20:5:75 MeOH: NH_4OH: CH_2Cl_2). m.p. 69–70 °C. δ_H (400 MHz, $(CD_3)_2SO$) 11.36 (2H, br s, pyrrole NH), 8.00 (1H, t, *J* = 5.7 Hz, amide NH-H-1'''), 7.94 (1H, t, *J* = 5.7 Hz, amide NH-H-1''), 6.84–6.79 (2H, m, H-5, H-5'), 6.75–6.69 (2H, m, H-3, H-3'), 6.07–6.03 (2H, m, H-4, H-4'), 3.24 (2H, dt, *J* = 6.0 and 7.0 Hz, H-1'''), 3.20 (2H, dt, *J* = 6.0 and 7.0 Hz, H-1''), 2.57–2.47 (4H, m, H-3''' and H-

4''), 1.61 (2H, quint, J = 7.0 Hz, H-2'''), 1.56–1.38 (4H, m, H-2'' and H-3''). The spectroscopic data was in agreement with literature values.[7]

4-Chloro-*N*-(3'''-((4''-(4'-chloro-1'*H*-pyrrole-2'- carboxamido)butyl)amino)propyl)-1*H*-pyrrole-2-carboxamide 17b

The reaction was carried out following General Procedure **A** using pyrrole **2b** (247 mg, 1 mmol) and amine **9** (0.08 mL, 0.5 mmol) in THF (2 mL), stirring for 72 h to give the crude product, which was then purified by flash chromatography (20:5:75 MeOH: NH_4OH: CH_2Cl_2) to afford the *title compound* **17b** (76 mg, 38%) as a pale cream solid. R_f = 0.67 (20:5:75 MeOH: NH_4OH: CH_2Cl_2). m.p. 80–81 °C. ν_{max} (ATR)/cm^{-1} 3226 (NH amine), 2933 (CH aliphatic), 1612 (C=O amide), 1568 (NH amide), 1524 (C=C aromatic), 1324 (CN aryl), 1123 (CN aliphatic), 821 (CH aromatic), 604 (C-Cl). δ_H (400 MHz, $(CD_3)_2SO$) 8.09 (1H, t, J = 5.8 Hz, amide NH-H-1'''), 8.05 (1H, t, J = 5.8 Hz, amide NH-H-1''), 6.92 (2H, d, J = 2.0 Hz, H-5 and H-5'), 6.75 (2H, dd, J = 7.5 and 2.0 Hz, H-3 and H-3'), 3.26–3.16 (4H, m, H-1'' and H-1'''), 2.54–2.47 (4H, m, H-3''' and H-4''), 1.60 (2H, quint, J = 6.9 Hz, H-2'''), 1.55–1.37 (4H, m, H-2'' and H-3''. δ_C (100 MHz, $(CD_3)_2SO$) 159.65 (C-2-C=O or C-2'-C=O), 159.59 (C-2-C=O or C-2'-C=O), 126.2 (C-2 or C-2'), 126.1 (C-2 or C-2'), 118.6 (C-5 and C-5'), 110.4 (C-4 and C-4'), 108.77 (C-3 or C-3'), 108.74 (C-3 or C-3'), 49.0 (C-4''), 47.0 (C-3'''), 38.5 (C-1''), 36.9 (C-1'''), 29.5 (C-2'''), 27.2 (C-2'' or C-3''), 27.0 (C-2'' or C-3''). *m/z* (ESI$^-$): 402 ([$^{37}Cl_2M-H]^-$, 12%), 400 ([$^{37}Cl^{35}ClM-H]^-$, 62), 398 ([$^{35}Cl_2M-H]^-$, 100), 297 (11). HRMS (ESI$^-$): Found [$^{37}Cl_2M-H]^-$: 402.1106, $C_{17}H_{22}{}^{37}Cl_2N_5O_2$ requires 402.1108. Found [$^{37}Cl^{35}ClM-H]^-$:400.1127, $C_{17}H_{22}{}^{37}Cl^{35}ClN_5O_2$ requires 400.1129. Found [$^{35}Cl_2M-H]^-$: 398.1155, $C_{17}H_{22}{}^{35}Cl_2N_5O_2$ requires 398.1156.

4,5-Dichloro-*N*-(3'''-((4''-(4',5'-dichloro-1'*H*-pyrrole-2'-carboxamido)butyl)amino)propyl)-1*H*-pyrrole-2-carboxamide 17c

The reaction was carried out following General Procedure **A** using pyrrole **2c** (281 mg, 1 mmol) and amine **9** (0.08 mL, 0.5 mmol) in THF (2 mL), stirring for 72 h to give the crude product, which was then purified by flash chromatography (20:5:75 MeOH: NH_4OH: CH_2Cl_2) to afford the *title compound* **17c** (84 mg, 36%) as a pale cream solid. R_f = 0.62 (20:5:75 MeOH: NH_4OH: CH_2Cl_2). m.p. 109–110 °C. δ_H (400 MHz, $(CD_3)_2SO$) 8.04–7.94 (2H, m, amide NH-H-1'' and amide NH-H-1'''), 6.77 (1H, s, H-3 or H-3'), 6.71 (1H, s, H-3 or H-3'), 3.27–3.17 (4H, m, H-1'' and H-1'''), 2.72–2.65 (4H, m, H-3''' and H-4''), 1.67 (2H, quint, *J* = 7.0 Hz, H-2'''), 1.55–1.45 (4H, m, H-2'' and H-3''). The spectroscopic data was in agreement with literature values.[7]

4-Bromo-*N*-(3'''-((4''-(4'-bromo-1'*H*-pyrrole-2'- carboxamido)butyl)amino)propyl)-1*H*-pyrrole-2-carboxamide 17d

The reaction was carried out following General Procedure **A** using pyrrole **2d** (291 mg, 1 mmol) and amine **9** (0.08 mL, 0.5 mmol) in THF (2 mL), stirring for 48 h to give the crude product, which was then purified by flash chromatography (20:5:75 MeOH: NH_4OH: CH_2Cl_2) to afford the *title compound* **17d** (51 mg, 21%) as a pale cream solid. R_f = 0.65 (20:5:75 MeOH: NH_4OH: CH_2Cl_2). m.p. 85–87 °C δ_H (400 MHz, $(CD_3)_2SO$) 8.11 (1H, t, *J* = 5.9 Hz, amide NH-H-1'''), 8.06 (1H, t, *J* = 5.9 Hz, amide NH-H-1''), 6.95 (2H, d, *J* = 1.5 Hz, H-5 and H-5'), 6.82 (2H, dd, *J* = 7.3 and 1.5 Hz, H-3 and H-3'), 3.23 (2H, dt, *J* = 6.0 and 7.0 Hz, H-1'''), 3.19 (2H, dt, *J* = 6.0 and 7.0 Hz, H-1''), 2.56–2.48 (4H, m, H-3''' and H-4''), 1.61 (2H, quint, *J* = 6.8 Hz, H-2'''), 1.55–1.37 (4H, m, H-2'' and H-3''). The spectroscopic data was in agreement with literature values.[9]

Psuedoceratidine 1

The reaction was carried out following General Procedure **A** using pyrrole **2e** (370 mg, 1 mmol) and amine **9** (0.08 mL, 0.5 mmol) in THF (2 mL), stirring for 48 h to give the crude product, which was then purified by flash chromatography (20:5:75 MeOH: NH_4OH: CH_2Cl_2) to afford the *title compound* **1** (135 mg, 42%) as a tan solid. R_f = 0.73 (20:5:75 MeOH: NH_4OH: CH_2Cl_2). m.p. 130–132 °C. (lit. m.p. 62–65 °C).[10] δ_H (400 MHz, $(CD_3)_2SO$) 8.02 (1H, t, J = 5.6 Hz, amide NH-H-1'''), 7.97 (1H, t, J = 5.6 Hz, amide NH-H-1''), 6.82 (1H, s, H-3 or H-3'), 6.77 (1H, s, H-3 or H-3'), 3.28–3.14 (4H, m, H-1'' and H-1'''), 2.73–2.62 (4H, m, H-3''' and H-4''), 1.67 (2H, quint, J = 6.8 Hz, H-2'''), 1.55–1.44 (4H, m, H-2'' and H-3''). The spectroscopic data was in agreement with literature values.[9,10]

4-Iodo-*N*-(3'''-((4''-(4'-iodo-1'*H*-pyrrole-2'-carboxamido)butyl)amino)propyl)-1*H*-pyrrole-2-carboxamide 17f

The reaction was carried out following General Procedure **A** using pyrrole **2f** (338 mg, 1 mmol) and amine **9** (0.08 mL, 0.5 mmol) in THF (2 mL), stirring for 18 h to give the crude product, which was then purified by flash chromatography (20:5:75 MeOH: NH_4OH: CH_2Cl_2) to afford the *title compound* **17f** (99 mg, 34%) as a tan solid. R_f = 0.56 (20:5:75 MeOH: NH_4OH: CH_2Cl_2). m.p. 89–90 °C. ν_{max} (ATR)/cm^{-1} 3190 (NH amine), 2931 (CH aliphatic), 1612 (C=O amide), 1560 (NH amide), 1516 (C=C aromatic), 1315 (CN aryl), 1129 (CN aliphatic), 824 (CH aromatic). δ_H (400 MHz, $(CD_3)_2SO$) 8.08 (1H, t, J = 5.7 Hz, amide NH-H-1'''), 8.03 (1H, t, J = 5.7 Hz, amide NH-H-1''), 6.95 (2H, d, J = 1.5 Hz, H-5 and H-5'), 6.87 (2H, dd, J = 8.0 and 1.5 Hz, H-3 and H-3'), 3.23 (2H, dt, J = 6.0 and 7.0 Hz, H-1'''), 3.19 (2H, dt, J = 6.0 and 7.0 Hz, H-1''), 2.55–2.47 (4H, m, H-3''' and H-4''), 1.60 (2H, quint, J = 7.0 Hz, H-2'''), 1.54–1.37 (4H, m, H-2'' and H-3''). δ_C (100 MHz, $(CD_3)_2SO$) 159.31 (C-2-C=O or C-2'-C=O), 159.25 (C-2-C=O or C-2'-C=O), 128.51 (C-2 or C-2'), 128.48 (C-2 or C-2'), 125.9 (C-5 and C-5'), 116.0 (C-3 and C-3'), 60.3 (C-4 and C-4'), 49.0 (C-4''), 46.9 (C-3'''), 38.4 (C-1''), 36.8 (C-1'''),

29.4 (C-2'''), 27.2 (C-2'' or C-3''), 26.9 (C-2'' or C-3''). *m/z* (ESI⁻): 582 ([M−H]⁻, 100%), 465 (10), 325 (40), 281 (30), 255 (55), 113 (15). HRMS (ESI⁻): Found [M−H]⁻: 581.9868, $C_{17}H_{22}I_2N_5O_2$ requires 581.9868.

_N_,_N'_-((Propane-1'',3''-diylbis(azanediyl))bis(propane-3',1'-diyl))bis(1_H_-pyrrole-2-carboxamide) 18a

The reaction was carried out following General Procedure **A** using pyrrole **2a** (212 mg, 1 mmol) and amine **10** (0.1 mL, 0.5 mmol) in THF (2 mL), stirring for 18 h to give the crude product, which was then purified by flash chromatography (20:5:75 MeOH: NH_4OH: CH_2Cl_2) to afford the *title compound* **18a** (62 mg, 33%) as a pale tan solid. R_f = 0.31 (20:5:75 MeOH: NH_4OH: CH_2Cl_2). m.p. 58–59 °C. ν_{max} (ATR)/cm^{-1} 3236 (NH amine), 2936 (CH aliphatic), 1615 (C=O amide), 1562 (NH amide), 1525 (C=C aromatic), 1323 (CN aryl), 1115 (CN aliphatic), 841 (CH aromatic). δ_H (400 MHz, $(CD_3)_2SO$) 11.37 (2H, br s, pyrrole NH), 8.06–7.96 (2H, m, amide NH), 6.80 (2H, s, H-5), 6.70 (2H, d, *J* = 2.5 Hz, H-3), 6.04 (2H, t, *J* = 2.5 Hz, H-4), 3.23 (4H, dt, *J* = 6.0 and 6.5 Hz, H-1'), 2.57–2.45 (8H, m, H-1'' and H-3'), 1.59 (4H, quint, *J* = 6.5 Hz, H-2'), 1.55–1.43 (2H, m, H-2''). δ_C (100 MHz, $(CD_3)_2SO$) 160.6 (C=O), 126.5 (C-2), 121.0 (C-5), 109.5 (C-3), 108.4 (C-4), 47.8 (C-1'' or C-3'), 47.2 (C-1'' or C-3'), 36.9 (C-1'), 29.9 (C-2''), 29.6 (C-2'). *m/z* (ESI⁻): 385 ([M−H]⁻, 100%), 339 (43), 325 (51), 311 (53), 283 (73), 255 (47), 147 (28), 120 (23), 93 (18). HRMS (ESI⁻): Found [M−H]⁻: 385.2352, $C_{20}H_{29}N_6O_2$ requires 385.2357.

_N_,_N'_-((Propane-1'',3''-diylbis(azanediyl))bis(propane-3',1'-diyl))bis(4-chloro-1_H_-pyrrole-2-carboxamide) 18b

The reaction was carried out following General Procedure **A** using pyrrole **2b** (247 mg, 1 mmol) and amine **10** (0.1 mL, 0.5 mmol) in THF (2 mL), stirring for 18 h to give the crude

product, which was then purified by flash chromatography (20:5:75 MeOH: NH_4OH: CH_2Cl_2) to afford the *title compound* **18b** (103 mg, 46%) as an off-white solid. R_f = 0.61 (20:5:75 MeOH: NH_4OH: CH_2Cl_2). m.p. 79–81 °C. ν_{max} (ATR)/cm^{-1} 3208 (NH amine), 2929 (CH aliphatic), 1619 (C=O amide), 1568 (NH amide), 1523 (C=C aromatic), 1324 (CN aryl), 1119 (CN aliphatic), 821 (CH aromatic), 604 (C-Cl). δ_H (400 MHz, $(CD_3)_2SO$) 8.18–8.07 (2H, m, amide NH), 6.92 (2H, d, *J* = 1.6 Hz, H-5), 6.75 (2H, d, *J* = 1.6 Hz, H-3), 3.23 (4H, dt, *J* = 6.0 and 6.7 Hz, H-1'), 2.57–2.48 (8H, m, H-1" and H-3'), 1.60 (4H, quint, *J* = 6.7 Hz, H-2'), 1.54 (2H, quint, *J* = 6.7 Hz, H-2"). δ_C (100 MHz, $(CD_3)_2SO$) 159.6 (C=O), 126.1 (C-2), 118.6 (C-5), 110.5 (C-4), 108.8 (C-3), 47.7 (C-1" or C-3'), 46.9 (C-1" or C-3'), 36.9 (C-1'), 29.5 (C-2"), 29.4 (C-2'). *m/z* (ESI$^-$): 457 ([$^{37}Cl_2$M−H]$^-$, 20%), 455 ([$^{37}Cl^{35}Cl$M−H]$^-$, 76), 453 ([$^{35}Cl_2$M−H]$^-$, 100), 339 (24), 325 (30), 311 (28), 283 (22), 249 (20), 147 (80), 130 (52), 113 (32). HRMS (ESI−): Found [$^{37}Cl_2$M−H]$^-$: 457.1530, $C_{20}H_{27}{}^{37}Cl_2N_6O_2$ requires 457.1533.

***N*,*N'*-((Propane-1",3"-diylbis(azanediyl))bis(propane-3',1'- diyl))bis(4,5-dichloro-1*H*-pyrrole-2-carboxamide) 18c**

The reaction was carried out following General Procedure **A** using pyrrole **2c** (281 mg, 1 mmol) and amine **10** (0.1 mL, 0.5 mmol) in THF (2 mL), stirring for 72 h to give the crude product, which was then purified by flash chromatography (20:5:75 MeOH: NH_4OH: CH_2Cl_2) to afford the *title compound* **18c** (83 mg, 32%) as a pale cream solid. R_f = 0.51 (20:5:75 MeOH: NH_4OH: CH_2Cl_2). m.p. 109–111 °C. ν_{max} (ATR)/cm^{-1} 3112 (NH amine), 2931 (CH aliphatic), 1615 (C=O amide), 1571 (NH amide), 1525 (C=C aromatic), 1325 (CN aryl), 1016 (CN aliphatic), 826 (CH aromatic), 585 (C-Cl). δ_H (400 MHz, $(CD_3)_2SO$) 8.00–7.90 (2H, m, amide NH), 6.69 (2H, s, H-3), 3.23 (4H, dt, *J* = 6.0 and 6.5 Hz, H-1'), 2.71 (4H, t, *J* = 6.8 Hz, H-1" or H-3'), 2.65 (4H, t, *J* = 6.8 Hz, H-1" or H-3'), 1.70–1.58 (6H, m, H-2' and H-2"). δ_C (100 MHz, $(CD_3)_2SO$) 161.0 (C=O), 126.4 (C-2), 117.0 (C-5), 109.4 (C-3), 106.1 (C-4), 46.7 (C-1" or C-3"), 45.8 (C-1" or

C-3'), 36.1 (C-1'), 28.4 (C-2'), 26.4 (C-2"). *m/z* (ESI$^-$): 529 ([$^{37}Cl_4M-H]^-$, 3%), 527 ([$^{37}Cl_3{}^{35}ClM-H]^-$, 15), 525 ([$^{37}Cl_2{}^{35}Cl_2M-H]^-$, 50), 523 ([$^{37}Cl^{35}Cl_3M-H]^-$, 100), 521 ([$^{35}Cl_4M-H]^-$, 79), 325 (18), 311 (18), 281 (16), 255 (17), 249 (21), 113 (28). HRMS (ESI$^-$): Found [$^{37}Cl^{35}Cl_3M-H]^-$: 523.0766, $C_{20}H_{25}{}^{37}Cl^{35}Cl_3N_6O_2$ requires 523.0771.

N,N'-((Propane-1",3"-diylbis(azanediyl))bis(propane-3',1'-diyl))bis(4-bromo-1*H*-pyrrole-2-carboxamide) 18d

The reaction was carried out following General Procedure **A** using pyrrole **2d** (291 mg, 1 mmol) and amine **10** (0.1 mL, 0.5 mmol) in THF (2 mL), stirring for 18 h to give the crude product, which was then purified by flash chromatography (20:5:75 MeOH: NH_4OH: CH_2Cl_2) to afford the *title compound* **18d** (80 mg, 30%) as an off-white solid. R_f = 0.41 (20:5:75 MeOH: NH_4OH: CH_2Cl_2). m.p. 80–82 °C. ν_{max} (ATR)/cm^{-1} 3127 (NH amine), 2929 (CH aliphatic), 1620 (C=O amide), 1564 (NH amide), 1521 (C=C aromatic), 1320 (CN aryl), 1124 (CN aliphatic), 824 (CH aromatic), 601 (C-Br). δ_H (400 MHz, $(CD_3)_2SO$) 8.19–8.11 (2H, m, amide NH), 6.95 (2H, d, J = 1.5 Hz, H-5), 6.81 (2H, d, J = 1.5 Hz, H-3), 3.24 (4H, dt, J = 6.0 and 7.0 Hz, H-1'), 2.59–2.49 (8H, m, H-1", H-3'), 1.62 (4H, quint, J = 7.0 Hz, H-2'), 1.56 (2H, quint, J = 7.0 Hz, H-2"). δ_C (100 MHz, $(CD_3)_2SO$) 159.5 (C=O), 127.0 (C-2), 121.0 (C-5), 111.2 (C-3), 94.8 (C-4), 47.5 (C-1" or C-3'), 46.7 (C-1" or C-3'), 36.8 (C-1'), 29.2 (C-2'), 28.9 (C-2"). *m/z* (ESI$^-$): 545 ([$^{81}Br_2M-H]^-$, 12%), 543 ([$^{81}Br^{79}BrM-H]^-$, 25), 541 ([$^{79}Br_2M-H]^-$, 13), 339 (21), 325 (32), 311 (23), 283 (21), 261 (13), 147 (100), 130 (13), 97 (13). HRMS (ESI$^-$): Found [$^{81}Br_2M-H]^-$: 545.0536, $C_{20}H_{27}{}^{81}Br_2N_6O_2$ requires 545.0531. Found [$^{79}Br_2M-H]^-$: 541.0569, $C_{20}H_{27}{}^{79}Br_2N_6O_2$ requires 541.0568.

N,N'-((Propane-1",3"-diylbis(azanediyl))bis(propane-3',1'-diyl))bis(4,5-dibromo-1*H*-pyrrole-2-carboxamide) 18e

The reaction was carried out following General Procedure **A** using pyrrole **2e** (370 mg, 1 mmol) and amine **10** (0.1 mL, 0.5 mmol) in THF (2 mL), stirring for 72 h to give the crude product, which was then purified by flash chromatography (20:5:75 MeOH: NH_4OH: CH_2Cl_2) to afford the *title compound* **18e** (137 mg, 41%) as a pale yellow solid. R_f = 0.54 (20:5:75 MeOH: NH_4OH: CH_2Cl_2). m.p. 79–81 °C. ν_{max} (ATR)/cm^{-1} 3107 (NH amine), 2925 (CH aliphatic), 1615 (C=O amide), 1563 (NH amide), 1520 (C=C aromatic), 1319 (CN aryl), 1112 (CN aliphatic), 826 (CH aromatic), 613 (C-Br). δ_H (400 MHz, $(CD_3)_2SO$) 8.02–7.92 (2H, m, amide NH), 6.75 (2H, s, H-3), 3.23 (4H, dt, *J* = 6.0 and 6.5 Hz, H-1'), 2.70 (4H, t, *J* = 7.0 Hz, H-1'' or H-3'), 2.64 (4H, t, *J* = 7.0 Hz, H-1'' or H-3'), 1.70–1.58 (6H, m, H-2', H-2''). δ_C (100 MHz, $(CD_3)_2SO$) 160.7 (C=O), 129.7 (C-2), 112.4 (C-3), 106.9 (C-5), 95.9 (C-4), 46.8 (C-1'' or C-3'), 45.9 (C-1'' or C-3'), 36.1 (C-1'), 28.5 (C-2'), 26.6 (C-2''). *m/z* (ESI^-): 705 ($[^{81}Br_4M{-}H]^-$, 19%), 703 ($[^{81}Br_3{}^{79}BrM{-}H]^-$, 67), 701 ($[^{81}Br_2{}^{79}Br_2M{-}H]^-$, 100), 699 ($[^{81}Br^{79}Br_3M{-}H]^-$, 67), 697 ($[^{79}Br_4M{-}H]^-$, 19), 283 (34), 255 (14). HRMS (ESI^-): Found $[^{81}Br^{79}Br_3M{-}H]^-$: 698.8764, $C_{20}H_{25}{}^{81}Br^{79}Br_3N_6O$ requires 698.8758. Found $[^{79}Br_4M{-}H]^-$: 696.8776, $C_{20}H_{25}{}^{79}Br_4N_6O$ requires 696.8778.

_N_,_N'_-((Propane-1'',3''-diylbis(azanediyl))bis(propane-3',1'- diyl))bis(4-iodo-1_H_-pyrrole-2-carboxamide) 18f

The reaction was carried out following General Procedure **A** using pyrrole **2f** (338 mg, 1 mmol) and amine **10** (0.1 mL, 0.5 mmol) in THF (2 mL), stirring for 18 h to give the crude product, which was then purified by flash chromatography (20:5:75 MeOH: NH_4OH: CH_2Cl_2) to afford the *title compound* **18f** (139 mg, 44%) as a pale cream solid. R_f = 0.49 (20:5:75 MeOH: NH_4OH: CH_2Cl_2). m.p. 78–80 °C. ν_{max} (ATR)/cm^{-1} 3118 (NH amine), 2930 (CH aliphatic), 1618 (C=O amide), 1561 (NH amide), 1520 (C=C aromatic), 1318 (CN aryl), 1112 (CN aliphatic), 829 (CH aromatic), 600 (C-I). δ_H (400 MHz, $(CD_3)_2SO$) 8.18–8.08 (2H, m, amide

NH), 6.96 (2H, d, *J* = 1.5 Hz, H-5), 6.87 (2H, d, *J* = 1.5 Hz, H-3), 3.23 (4H, dt, *J* = 6.0 and 6.8 Hz, H-1'), 2.59–2.48 (8H, m, H-1'', H-3'), 1.61 (4H, quint, *J* = 6.8 Hz, H-2'), 1.55 (2H, quint, *J* = 6.8 Hz, H-2''). δ_C (100 MHz, $(CD_3)_2SO$) 159.3 (C=O), 128.5 (C-2), 125.9 (C-5), 116.0 (C-3), 60.3 (C-4), 47.6 (C-1'' or C-3'), 46.8 (C-1'' or C-3'), 36.8 (C-1'), 29.2 (C-2'), 29.1 (C-2''). *m/z* (ESI^-): 637 ($[M-H]^-$, 100%), 339 (46), 325 (52), 311 (34), 113 (24). HRMS (ESI^-): Found $[M-H]^-$: 637.0291, $C_{20}H_{27}I_2N_6O_2$ requires 637.0290.

_N_,_N'_-((Butane-1'',4''-diylbis(azanediyl))bis(propane-3',1'- diyl))bis(1_H_-pyrrole-2-carboxamide) 19a

The reaction was carried out following General Procedure **A** using pyrrole **2a** (212 mg, 1 mmol) and amine **11** (0.11 mL, 0.5 mmol) in THF (2 mL), stirring for 18 h to give the crude product. The crude product was then triturated in MeOH (20 mL), and the precipitated white solid was filtered, dried and collected to afford the *title compound* **19a** (140 mg, 72%) as an off-white, powdery solid. m.p. 175–176 °C. ν_{max} (ATR)/cm^{-1} 3279 (NH amine), 3065 (CH aromatic), 2919 (CH aliphatic), 1621 (C=O amide), 1565 (NH amide), 1530 (C=C aromatic), 1333 (CN aryl), 1146 (CN aliphatic). δ_H (400 MHz, $(CD_3)_2SO$) 11.38 (2H, br s, pyrrole NH), 8.00 (2H, t, *J* = 5.4 Hz, amide NH), 6.81 (2H, s, H-5), 6.70 (2H, d, *J* = 3.0 Hz, H-3), 6.05 (2H, t, *J* = 3.0 Hz, H-4), 3.24 (4H, dt, *J* = 6.0 and 6.5 Hz, H-1'), 2.55–2.44 (8H, m, H-1'', H-3'), 1.60 (4H, quint, *J* = 6.5 Hz, H-2'), 1.45–1.37 (4H, m, H-2''). δ_C (100 MHz, $(CD_3)_2SO$) 160.6 (C=O), 126.4 (C-2), 121.0 (C-5), 109.5 (C-3), 108.4 (C-4), 49.4 (C-1''), 47.1 (C-3'), 36.9 (C-1'), 29.6 (C-2'), 27.5 (C-2''). *m/z* (ESI^-): 387 ($[M-H]^-$, 100%), 113 (10). HRMS (ESI^-): Found $[M-H]^-$: 387.2516, $C_{20}H_{31}N_6O_2$ requires 387.2514.

_N_,_N'_-((Butane-1'',4''-diylbis(azanediyl))bis(propane-3',1'- diyl))bis(4-chloro-1_H_-pyrrole-2-carboxamide) 19b

The reaction was carried out following General Procedure **A** using pyrrole **2b** (247 mg, 1 mmol) and amine **11** (0.11 mL, 0.5 mmol) in THF (2 mL), stirring for 18 h to give the crude product. The crude product was then triturated in 1:1 MeOH/EtOH (20 mL), and the precipitated white solid was filtered, dried and collected to afford the *title compound* **19b** (139 mg, 61%) as a white, powdery solid. m.p. 194–195 °C. ν_{max} (ATR)/cm^{-1} 3308 (NH amine), 3120 (CH aromatic), 2928 (CH aliphatic), 1624 (C=O amide), 1569 (NH amide), 1531 (C=C aromatic), 1334 (CN aryl), 1105 (CN aliphatic), 776 (C-Cl). δ_H (400 MHz, $(CD_3)_2SO$) 8.10 (2H, t, *J* = 5.4 Hz, amide NH), 6.92 (2H, d, *J* = 1.5 Hz, H-3), 6.74 (2H, d, *J* = 1.5 Hz, H-5), 3.23 (4H, dt, *J* = 6.0 and 6.7 Hz, H-1'), 2.54–2.43 (8H, m, H-1'', H-3'), 1.59 (4H, quint, *J* = 6.7 Hz, H-2'), 1.44–1.37 (4H, m, H-2''). δ_C (100 MHz, $(CD_3)_2SO$) 159.6 (C=O), 126.2 (C-2), 118.6 (C-5), 110.4 (C-4), 108.7 (C-3), 49.4 (C-1''), 47.0 (C-3'), 37.0 (C-1'), 29.6 (C-2'), 27.6 (C-2''). *m/z* (ESI$^-$): 459 ([$^{37}Cl_2M-H]^-$, 14%), 457 ([$^{37}Cl^{35}ClM-H]^-$, 64), 455 ([$^{35}Cl_2M-H]^-$, 100). HRMS (ESI$^-$): Found [$^{37}Cl_2M-H]^-$: 459.1691, $C_{20}H_{29}{}^{37}Cl_2N_6O_2$ requires 459.1689.

_N_,_N'_-((Butane-1'',4''-diylbis(azanediyl))bis(propane-3',1'- diyl))bis(4,5-dichloro-1_H_-pyrrole-2-carboxamide) 19c

The reaction was carried out following General Procedure **A** using pyrrole **2c** (281 mg, 1 mmol) and amine **11** (0.11 mL, 0.5 mmol) in THF (2 mL), stirring for 18 h to give the crude product. The crude product was then triturated in CH_2Cl_2 (20 mL), and the cream solid was filtered, dried and collected to afford the *title compound* **19c** (49 mg, 19%) as a tan solid. m.p. 110–112 °C. ν_{max} (ATR)/cm^{-1} 3066 (NH amine), 2946 (CH aliphatic), 1606 (C=O amide), 1576 (NH amide), 1533 (C=C aromatic), 1330 (CN aryl), 1052 (CN aliphatic), 829 (CH aromatic), 752 (C-Cl). δ_H (400 MHz, $(CD_3)_2SO$) 7.97 (2H, t, *J* = 5.9 Hz, amide NH), 6.67 (2H, s, H-3), 3.23 (4H, dt, *J* = 6.0 and 6.7 Hz, H-1'), 2.69–2.60 (8H, m, H-1'' and H-3'), 1.67 (4H, quint, *J* = 6.7 Hz, H-2'), 1.59–1.48 (4H, m, H-2''). δ_C (100 MHz, $(CD_3)_2SO$) 161.4 (C=O), 126.7 (C-2), 117.4

(C-5), 109.4 (C-3), 105.8 (C-4), 47.8 (C-1''), 45.5 (C-3'), 36.1 (C-1'), 28.3 (C-2'), 25.9 (C-2''). *m/z* (ESI⁻): 531 ([$^{37}Cl_4M-H]^-$, 2%), 529 ([$^{37}Cl_3{}^{35}ClM-H]^-$, 12), 527 ([$^{37}Cl_2{}^{35}Cl_2M-H]^-$, 50), 525 ([$^{37}Cl^{35}Cl_3M-H]^-$, 100), 523 ([$^{35}Cl_4M-H]^-$, 80), 488 (8), 388 (4), 330 (4), 262 (8), 113 (4). HRMS (ESI⁻): Found [$^{37}Cl_2{}^{35}Cl_2M-H]^-$: 527.0896, $C_{20}H_{27}{}^{37}Cl_2{}^{35}Cl_2N_6O_2$ requires 527.0901.

N,N'-((Butane-1'',4''-diylbis(azanediyl))bis(propane-3',1'-diyl))bis(4-bromo-1*H*-pyrrole-2-carboxamide) 19d

The reaction was carried out following General Procedure **A** using pyrrole **2d** (291 mg, 1 mmol) and amine **11** (0.11 mL, 0.5 mmol) in THF (2 mL), stirring for 18 h to give the crude product. The crude product was then triturated with CH_2Cl_2 (20 mL), and the precipitated white solid was filtered, dried and collected to afford the *title compound* **19d** (210 mg, 77%) as a white solid. m.p. 173–175 °C. ν_{max} (ATR)/cm^{-1} 3273 (NH amine), 3068 (CH aromatic), 2919 (CH aliphatic), 1621 (C=O amide), 1566 (NH amide), 1529 (C=C aromatic), 1334 (CN aryl), 1115 (CN aliphatic), 676 (C-Br). δ_H (400 MHz, $(CD_3)_2SO$) 8.11 (2H, t, *J* = 5.4 Hz, amide NH), 6.94 (2H, d, *J* = 1.5 Hz, H-5), 6.79 (2H, d, *J* = 1.5 Hz, H-3), 3.23 (4H, dt, *J* = 6.0 and 7.0 Hz, H-1'), 2.53–2.43 (8H, m, H-1'' and H-3'), 1.59 (4H, quint, *J* = 7.0 Hz, H-2'), 1.44–1.37 (4H, m, H-2''). δ_C (100 MHz, $(CD_3)_2SO$) 159.5 (C=O), 127.1 (C-2), 120.9 (C-5), 111.1 (C-3), 94.8 (C-4), 49.4 (C-1''), 47.0 (C-3'), 37.0 (C-1'), 29.5 (C-2'), 27.6 (C-2''). *m/z* (ESI⁻): 547 ([$^{81}Br_2M-H]^-$, 56%), 545 ([$^{81}Br^{79}BrM-H]^-$, 100), 543 ([$^{79}Br_2M-H]^-$, 56), 455 (18), 113 (13). HRMS (ESI⁻): Found [$^{79}Br_2M-H]^-$: 543.0720, $C_{20}H_{29}{}^{79}Br_2N_6O_2$ requires 543.0724.

N,N'-((Butane-1'',4''-diylbis(azanediyl))bis(propane-3',1'-diyl))bis(4,5-dibromo-1*H*-pyrrole-2-carboxamide) 19e

The reaction was carried out following General Procedure **A** using pyrrole **2e** (370 mg, 1 mmol) and amine **11** (0.11 mL, 0.5 mmol) in THF (2 mL), stirring for 18 h to give the crude product. The crude product was then triturated in CH_2Cl_2 (20 mL), and the precipitated creamy yellow

solid was filtered, dried and collected to afford the *title compound* **19e** (257 mg, 73%) as a pale tan solid. m.p. 201–203 °C δ_H (400 MHz, $(CD_3)_2SO$) 8.01 (2H, t, *J* = 5.7 Hz, amide NH), 6.74 (2H, s, H-3), 3.23 (4H, dt, *J* = 6.0 and 6.8 Hz, H-1'), 2.69–2.60 (8H, m, H-1" and H-3'), 1.67 (4H, quint, *J* = 6.8 Hz, H-2'), 1.58–1.49 (4H, m, H-2"). The spectroscopic data was in agreement with literature values.[11]

***N*,*N*'-((Butane-1",4"-diylbis(azanediyl))bis(propane-3',1'- diyl))bis(4-iodo-1*H*-pyrrole-2-carboxamide) 19f**

The reaction was carried out following General Procedure **A** using pyrrole **2f** (338 mg, 1 mmol) and amine **11** (0.11 mL, 0.5 mmol) in THF (2 mL), stirring for 18 h to give the crude product. The crude product was then triturated with CH_2Cl_2 (20 mL), and the precipitated white solid was filtered, dried and collected to afford the *title compound* **19f** (271 mg, 85%) as a white solid. m.p. 173–175 °C. ν_{max} (ATR)/cm^{-1} 3066 (NH amine), 2945 (CH aliphatic), 1606 (C=O amide), 1575 (NH amide), 1533 (C=C aromatic), 1329 (CN aryl), 1051 (CN aliphatic), 830 (CH aromatic), 590 (C-I). δ_H (400 MHz, $(CD_3)_2SO$) 8.09 (2H, t, *J* = 5.6 Hz, amide NH), 6.95 (2H, d, *J* = 1.5 Hz, H-5), 6.85 (2H, d, *J* = 1.5 Hz, H-3), 3.23 (4H, dt, *J* = 6.8 Hz, 6.0 and 7.0 Hz, H-1'), 2.54–2.43 (8H, m, H-1" and H-3'), 1.59 (4H, quint, *J* = 7.0 Hz, H-2'), 1.44–1.36 (4H, m, H-2"). δ_C (100 MHz, $(CD_3)_2SO$) 159.3 (C=O), 128.5 (C-2), 125.9 (C-5), 115.9 (C-3), 60.3 (C-4), 49.4 (C-1"), 47.1 (C-3'), 37.0 (C-1'), 29.5 (C-2'), 27.6 (C-2"). *m/z* (ESI$^-$): 639 ([M−H]$^-$, 100%), 488 (55), 330 (36), 113 (15). HRMS (ESI$^-$): Found [M−H]$^-$: 639.0451, $C_{20}H_{29}I_2N_6O_2$ requires 639.0447.

***N*-(3'-(Dimethylamino)propyl)-1*H*-pyrrole-2-carboxamide 27a**

The reaction was carried out following General Procedure **B** using pyrrole **2a** (212 mg, 1 mmol) and amine **25** (0.13 mL, 1 mmol) in THF (2 mL), stirring for 18 h to give the crude product.

The solvent was removed *in vacuo*, to afford the *title compound* **27a** (90 mg, 46%) as a pale tan, powdery solid. m.p. 84–85 °C. ν_{max} (ATR)/cm^{-1} 3297 (NH amine), 3050 (CH aromatic), 2953 (CH aliphatic), 1614 (C=O amide), 1559 (NH amide), 1524 (C=C aromatic), 1390 (CH aliphatic), 1325 (CN aryl), 1135 (CN aliphatic). δ_H (400 MHz, $(CD_3)_2SO$) 11.36 (1H, br s, NH), 7.97 (1H, t, *J* = 5.7 Hz, amide NH), 6.83–6.80 (1H, m, H-3), 6.72–6.68 (1H, m, H-5), 6.07–6.04 (1H, m, H-4), 3.21 (2H, dt, *J* = 6.0 and 7.0 Hz, H-1'), 2.23 (2H, t, *J* = 7.0 Hz, H-3'), 2.12 (6H, s, $N(CH_3)_2$), 1.61 (2H, quint, *J* = 7.0 Hz, H-2'). δ_C (100 MHz, $(CD_3)_2SO$) 160.6 (C=O), 126.4 (C-2), 121.0 (C-5), 109.4 (C-3), 108.4 (C-4), 57.0 (C-3'), 45.2 ($N(CH_3)_2$), 37.0 (C-1'), 27.4 (C-2'). *m/z* (ESI$^-$): 194 ([M−H]$^-$, 100%), 144 (10), 97 (10). HRMS (ESI$^-$): Found [M−H]$^-$: 194.1295, $C_{10}H_{16}N_3O$ requires 194.1299.

4-Chloro-*N*-(3'-(dimethylamino)propyl)-1*H*-pyrrole-2- carboxamide 27b

The reaction was carried out following General Procedure **B** using pyrrole **2b** (247 mg, 1 mmol) and amine **25** (0.13 mL, 1 mmol) in THF (2 mL), stirring for 18 h to give the crude product. The solvent was removed *in vacuo*, to afford the *title compound* **27b** (223 mg, 97%) as an off-white solid. m.p. 169–171 °C. ν_{max} (ATR)/cm^{-1} 3311 (NH amine), 3102 (CH aromatic), 2938 (CH aliphatic), 1628 (C=O amide), 1572 (NH amide), 1533 (C=C aromatic), 1393 (CH aliphatic), 1331 (CN aryl), 1052 (CN aliphatic), 613 (C-Cl). δ_H (400 MHz, $(CD_3)_2SO$) 11.69 (1H, br s, pyrrole NH), 8.07 (1H, t, *J* = 5.7 Hz, amide NH), 6.92 (1H, d, *J* = 1.5 Hz, H-5), 6.74 (1H, d, *J* = 1.5 Hz, H-3), 3.21 (2H, dt, *J* = 6.0 and 7.0 Hz, H-1'), 2.22 (2H, t, *J* = 7.0 Hz, H-3'), 2.12 (6H, s, $N(CH_3)_2$), 1.60 (2H, quint, *J* = 7.0 Hz, H-2'). δ_C (100 MHz, $(CD_3)_2SO$) 159.6 (C=O), 126.1 (C-2), 118.6 (C-5), 110.4 (C-4), 108.7 (C-3), 56.8 (C-3'), 45.2 ($N(CH_3)_2$), 37.0 (C-1'), 27.3 (C-2'). *m/z* (ESI$^-$): 230 ([^{37}ClM−H]$^-$, 38%), 228 ([^{35}ClM−H]$^-$, 100), 100 (4). HRMS (ESI$^-$): Found [^{37}ClM−H]$^-$: 230.0886, $C_{10}H_{15}{}^{37}ClN_3O$ requires 230.0882. Found [^{35}ClM−H]$^-$: 228.0912, $C_{10}H_{15}{}^{35}ClN_3O$ requires 228.0909.

4,5-Dichloro-*N*-(3'-(dimethylamino)propyl)-1*H*-pyrrole-2- carboxamide 27c

The reaction was carried out following General Procedure **B** using pyrrole **2c** (281 mg, 1 mmol) and amine **25** (0.13 mL, 1 mmol) in THF (2 mL), stirring for 18 h to give the crude product. The crude product was then triturated with CH_2Cl_2 (20 mL), and recrystallised from EtOH (5 mL), to afford the *title compound* **27c** (139 mg, 53%) as a pale cream solid. m.p. 175–177 °C. ν_{max} (ATR)/cm^{-1} 3291 (NH amine), 3106 (CH aromatic), 2946 (CH aliphatic), 1628 (C=O amide), 1575 (NH amide), 1537 (C=C aromatic), 1352 (CH aliphatic), 1334 (CN aryl), 1028 (CN aliphatic), 690 (C-Cl). δ_H (400 MHz, $(CD_3)_2SO$) 8.09 (1H, t, *J* = 5.7 Hz, amide NH), 6.83 (1H, s, H-3), 3.21 (2H, dt, *J* = 6.0 and 7.0 Hz, H-1'), 2.25 (2H, t, *J* = 7.0 Hz, H-3'), 2.14 (6H, s, $N(CH_3)_2$), 1.61 (2H, quint, *J* = 7.0 Hz, H-2'). δ_C (100 MHz, $(CD_3)_2SO$) 159.1 (C=O), 125.2 (C-2), 114.8 (C-5), 109.3 (C-3), 107.6 (C-4), 56.7 (C-3'), 45.0 ($N(CH_3)_2$), 36.9 (C-1'), 27.2 (C-2'). *m/z* (ESI$^-$): 266 ([$^{37}Cl_2M-H]^-$, 14%), 264 ([$^{37}Cl^{35}ClM-H]^-$, 71), 262 ([$^{35}Cl_2M-H]^-$, 100), 134 (7). HRMS (ESI$^-$): Found [$^{35}Cl_2M-H]^-$: 262.0514, $C_{10}H_{14}{}^{35}Cl_2N_3O$ requires 262.0519.

4-Bromo-*N*-(3'-(dimethylamino)propyl)-1*H*-pyrrole-2- carboxamide 27d

The reaction was carried out following General Procedure **B** using pyrrole **2d** (291 mg, 1 mmol) and amine **25** (0.13 mL, 1 mmol) in THF (2 mL), stirring for 18 h to give the crude product. The solvent was removed *in vacuo*, to afford the *title compound* **2.25d** (265 mg, 97%) as a pale cream solid. m.p. 174–176 °C. ν_{max} (ATR)/cm^{-1} 3301 (NH amine), 3102 (CH aromatic), 2955 (CH aliphatic), 1627 (C=O amide), 1568 (NH amide), 1530 (C=C aromatic), 1389 (CH aliphatic), 1330 (CN aryl), 1143 (CN aliphatic), 614 (C-Br). δ_H (400 MHz, $(CD_3)_2SO$) 11.76 (1H, br s, pyrrole NH), 8.07 (1H, t, *J* = 5.7 Hz, amide NH), 6.95 (1H, d, *J* = 1.5 Hz, H-5), 6.80 (1H, d, *J* = 1.5 Hz, H-3), 3.21 (2H, dt, *J* = 6.0 and 7.0 Hz, H-1'), 2.22 (2H, t, *J* = 7.0 Hz, H-3'), 2.12 (6H, s, $N(CH_3)_2$), 1.60 (2H, quint, *J* = 7.0 Hz, H-2'). δ_C (100 MHz, $(CD_3)_2SO$) 159.5

(C=O), 127.0 (C-2), 120.9 (C-5), 111.1 (C-3), 94.8 (C-4), 56.8 (C-3'), 45.2 ($N(CH_3)_2$), 36.9 (C-1'), 27.3 (C-2'). *m/z* (ESI^-): 274 ($[^{81}BrM-H]^-$, 100%), 272 ($[^{79}BrM-H]^-$, 96), 144 (8). HRMS (ESI^-): Found $[^{79}BrM-H]^-$: 272.0410, $C_{10}H_{15}{}^{79}BrN_3O$ requires 272.0404.

4,5-Dibromo-*N*-(3'-(dimethylamino)propyl)-1*H*-pyrrole-2- carboxamide 27e

The reaction was carried out following General Procedure **B** using pyrrole **2e** (370 mg, 1 mmol) and amine **25** (0.13 mL, 1 mmol) in THF (2 mL), stirring for 72 h to give the crude product. The solvent was removed *in vacuo*, to afford the *title compound* **27e**(192 mg, 54%) as a white, powdery solid. m.p. 189–191 °C. ν_{max} (ATR)/cm^{-1} 3289 (NH amine), 3105 (CH aromatic), 2945 (CH aliphatic), 1626 (C=O amide), 1570 (NH amide), 1533 (C=C aromatic), 1351 (CH aliphatic), 1331 (CN aryl), 1082 (CN aliphatic), 694 (C-Br). δ_H (400 MHz, $(CD_3)_2SO$) 8.06 (1H, t, *J* = 5.7 Hz, amide NH), 6.86 (1H, s, H-3), 3.20 (2H, dt, *J* = 6.0 and 7.0 Hz, H-1'), 2.24 (2H, t, *J* = 7.0 Hz, H-3'), 2.13 (6H, s, $N(CH_3)_2$), 1.60 (2H, quint, *J* = 7.0 Hz, H-2'). δ_C (100 MHz, $(CD_3)_2SO$) 159.0 (C=O), 128.5 (C-2), 112.3 (C-3), 104.6 (C-5), 97.5 (C-4), 56.7 (C-3'), 45.1 ($N(CH_3)_2$), 36.9 (C-1'), 27.2 (C-2'). *m/z* (ESI^-): 354 ($[^{81}Br_2M-H]^-$, 50%), 352 ($[^{81}Br^{79}BrM-H]^-$, 100), 350 ($[^{79}Br_2M-H]^-$, 53). HRMS (ESI^-): Found $[^{81}Br_2M-H]^-$: 353.9468, $C_{10}H_{14}{}^{81}Br_2N_3O$ requires 353.9469.

***N*-(3'-(Dimethylamino)propyl)-4-iodo-1*H*-pyrrole-2- carboxamide 27f**

The reaction was carried out following General Procedure **B** using pyrrole **2f** (338 mg, 1 mmol) and amine **25** (0.13 mL, 1 mmol) in THF (2 mL), stirring for 18 h to give the crude product. The crude product was then triturated in CH_2Cl_2 (20 mL), followed by recrystallisation in EtOH (5 mL) to afford the *title compound* **27f** (213 mg, 66%) as a white solid. m.p. 169–171 °C. ν_{max} (ATR)/cm^{-1} 3288 (NH amine), 3103 (CH aromatic), 2953 (CH aliphatic), 1628 (C=O amide),

1565 (NH amide), 1528 (C=C aromatic), 1377 (CH aliphatic), 1327 (CN aryl), 1144 (CN aliphatic), 614 (C-I). δ_H (400 MHz, $(CD_3)_2SO$) 11.73 (1H, br s, pyrrole NH), 8.04 (1H, t, J = 5.7 Hz, amide NH), 6.96 (1H, s, H-5), 6.86 (1H, s, H-3), 3.20 (2H, dt, J = 6.0 and 7.0 Hz, H-1'), 2.22 (2H, t, J = 7.0 Hz, H-3'), 2.11 (6H, s, $N(CH_3)_2$), 1.59 (2H, quint, J = 7.0 Hz, H-2'). δ_C (100 MHz, $(CD_3)_2SO$) 159.3 (C=O), 128.5 (C-2), 125.9 (C-5), 115.9 (C-3), 60.3 (C-4), 56.8 (C-3'), 45.2 ($N(CH_3)_2$), 36.9 (C-1'), 27.3 (C-2'). *m/z* (ESI^-): 320 ($[M-H]^-$, 100%), 192 (7). HRMS (ESI^-): Found $[M-H]^-$: 320.0260, $C_{10}H_{15}IN_3O$ requires 320.0265.

***tert*-Butyl (3'-(1*H*-pyrrole-2-carboxamido)propyl)carbamate 28a**

The reaction was carried out following General Procedure **B** using pyrrole **2a** (212 mg, 1 mmol) and amine **26** (0.17 mL, 1 mmol) in THF (2 mL), stirring for 18 h to give the crude product. The crude product was then dissolved in CH_2Cl_2 (1 mL), followed by addition of petroleum ether until a white solid precipitated. The white solid was filtered, dried and collected to afford the *title compound* **28a** (138 mg, 52%) as a white, powdery solid. m.p. 90–91 °C. ν_{max} (ATR)/cm^{-1} 3362 (NH amine), 2935 (CH aliphatic), 1678 (C=O amide), 1611 (NH amide), 1520 (C=C aromatic), 1367 (CH aliphatic), 1342 (CN aryl), 1165 (C-O ester), 1128 (CN aliphatic), 815 (CH aromatic). δ_H (400 MHz, $(CD_3)_2SO$) 11.38 (1H, br s, pyrrole NH), 7.92 (1H, t, J = 5.7 Hz, amide NH-pyrrole), 6.84–6.81 (1H, m, H-5), 6.79 (1H, t, J = 5.7 Hz, amide NH-Boc), 6.73–6.70 (1H, m, H-3), 6.08–6.04 (1H, m, H-4), 3.19 (2H, dt, J = 6.0 and 7.0 Hz, H-1'), 2.95 (2H, dt, J = 6.0 and 7.0 Hz, H-3'), 1.58 (2H, quint, J = 7.0 Hz, H-2'), 1.37 (9H, s, $C(CH_3)_3$). δ_C (100 MHz, $(CD_3)_2SO$) 160.6 (C-2-C=O), 155.6 (O=C-O), 126.3 (C-2), 121.1 (C-5), 109.5 (C-3), 108.4 (C-4), 77.5 ($C(CH_3)_3$), 37.6 (C-3'), 36.1 (C-1'), 29.9 (C-2'), 28.2 ($C(CH_3)_3$). *m/z* (ESI^+): 290 ($[M+Na]^+$, 100%), 234 (7), 190 (50), 151 (8). HRMS (ESI^+): Found $[M+Na]^+$: 290.1477, $C_{13}H_{21}N_3NaO_3$ requires 290.1475.

tert-Butyl (3'-(4-chloro-1_H_-pyrrole-2- carboxamido)propyl)carbamate 28b

The reaction was carried out following General Procedure **B** using pyrrole **2b** (247 mg, 1 mmol) and amine **26** (0.17 mL, 1 mmol) in THF (2 mL), stirring for 18 h to give the crude product. The crude product was then purified by flash chromatography (1→5% MeOH/ CH_2Cl_2) to afford the *title compound* **28 b** (179 mg, 59%) as a pale cream solid. m.p. 127–129 °C. R_f = 0.34. ν_{max} (ATR)/cm^{-1} 3210 (NH amine), 3124 (CH aromatic), 2975 (CH aliphatic), 1683 (C=O amide), 1625 (NH amide), 1532 (C=C aromatic), 1366 (CH aliphatic), 1333 (CN aryl), 1161 (C-O ester), 1148 (CN aliphatic), 607 (C-Cl). δ_H (400 MHz, $(CD_3)_2SO$) 11.72 (1H, br s, pyrrole NH), 8.03 (1H, t, *J* = 5.7 Hz, amide NH-pyrrole), 6.95–6.91 (1H, m, H-5), 6.79 (1H, t, *J* = 5.7 Hz, amide NH-Boc), 6.76–6.73 (1H, m, H-3), 3.19 (2H, dt, *J* = 6.0 and 7.0 Hz, H-1'), 2.95 (2H, dt, *J* = 6.0 and 7.0 Hz, H-3'), 1.58 (2H, quint, *J* = 7.0 Hz, H-2'), 1.37 (9H, s, $C(CH_3)_3$). δ_C (100 MHz, $(CD_3)_2SO$) 159.7 (C-2-C=O), 155.6 (O=C-O), 126.0 (C-2), 118.6 (C-5), 110.4 (C-4), 108.8 (C-3), 77.5 ($C(CH_3)3$), 37.7 (C-3'), 36.3 (C-1'), 29.7 (C-2'), 28.2 ($C(CH_3)_3$). *m/z* (ESI$^-$): 302 ([^{37}ClM−H]$^-$, 8%), 300 ([^{35}ClM−H]$^-$, 23), 226 (100). HRMS (ESI$^-$): Found [^{37}ClM−H]$^-$: 302.1090 $C_{13}H_{19}{}^{37}ClN_3O_3$ requires 302.1095.

tert-Butyl (3'-(4,5-dichloro-1_H_-pyrrole-2- carboxamido)propyl)carbamate 28c

The reaction was carried out following General Procedure **B** using pyrrole **2c** (281 mg, 1 mmol) and amine **26** (0.17 mL, 1 mmol) in THF (2 mL), stirring for 18 h to give the crude product. The crude product was then triturated with CH_2Cl_2 (10 mL) to afford the *title compound* **28c** (145 mg, 43%) as a pale cream solid. m.p. 158–160 °C. ν_{max} (ATR)/cm^{-1} 3339 (NH amine), 3127 (CH aromatic), 2975 (CH aliphatic), 1639 (C=O amide), 1571 (NH amide), 1530 (C=C aromatic), 1365 (CH aliphatic), 1326 (CN aryl), 1160 (C-O ester), 1091 (CN aliphatic), 648 (C-Cl). δ_H (400 MHz, $(CD_3)_2SO$) 12.67 (1H, br s, pyrrole NH), 8.08 (1H, t, *J* = 5.7 Hz, amide NH-pyrrole), 6.85 (1H, s, H-3), 6.79 (1H, t, *J* = 5.7 Hz, amide NH-Boc), 3.18 (2H, dt, *J* = 6.0

and 7.0 Hz, H-1'), 2.94 (2H, dt, J = 6.0 and 7.0 Hz, H-3'), 1.58 (2H, quint, J = 7.0 Hz, H-2'), 1.37 (9H, s, $C(CH_3)_3$). δ_C (100 MHz, $(CD_3)_2SO$) 159.0 (C-2-C=O), 155.6 (O=C-O), 125.0 (C-2), 114.7 (C-5), 109.4 (C-3), 107.8 (C-4), 77.5 ($C(CH_3)_3$), 37.7 (C-3'), 36.4 (C-1'), 29.7 (C-2'), 28.2 ($C(CH_3)_3$). *m/z* (ESI^-): 338 ($[^{37}Cl_2M{-}H]^-$, 13%), 336 ($[^{37}Cl^{35}ClM{-}H]^-$, 65), 334 ($[^{35}Cl_2M{-}H]^-$, 100), 260 (53), 234 (15). HRMS (ESI^-): Found $[^{37}Cl_2M{-}H]^-$: 338.0677, $C_{13}H_{18}{}^{37}Cl_2N_3O_3$ requires 338.0679. Found $[^{37}Cl^{35}Cl\ M{-}H]^-$: 336.0698, $C_{13}H_{18}{}^{37}Cl^{35}ClN_3O_3$ requires 336.0703.

***tert*-Butyl (3'-(4-bromo-1*H*-pyrrole-2-carboxamido)propyl)carbamate 28d**

The reaction was carried out following General Procedure **B** using pyrrole **2d** (291 mg, 1 mmol) and amine **26** (0.17 mL, 1 mmol) in THF (2 mL), stirring for 18 h to give the crude product. The crude product was then triturated in CH_2Cl_2 (10 mL) to afford the *title compound* **28d** (228 mg, 68%) as a pale yellow solid. m.p. 132–134 °C. ν_{max} (ATR)/cm- 1 3348 (NH amine), 3222 (CH aromatic), 2977 (CH aliphatic), 1695 (C=O amide), 1617 (NH amide), 1518 (C=C aromatic), 1365 (CH aliphatic), 1336 (CN aryl), 1160 (C-O ester), 1135 (CN aliphatic), 597 (C-Br). δ_H (400 MHz, $(CD_3)_2SO$) 11.38 (1H, br s, pyrrole NH), 8.03 (1H, t, J = 5.7 Hz, amide NH-pyrrole), 6.95 (1H, d, J = 1.5 Hz, H-5), 6.81 (1H, d, J = 1.5 Hz, H-3), 6.78 (1H, t, J = 5.7 Hz, amide NH-Boc), 3.19 (2H, dt, J = 6.0 and 7.0 Hz, H-1'), 2.95 (2H, dt, J = 6.0 and 7.0 Hz, H-3'), 1.58 (2H, quint, J = 7.0 Hz, H-2'), 1.37 (9H, s, $C(CH_3)_3$). δ_C (100 MHz, $(CD_3)_2SO$) 159.6 (C-2-C=O), 155.6 (O=C-O), 126.9 (C-2), 121.0 (C-5), 111.2 (C-3), 94.8 (C-4), 77.5 ($C(CH_3)_3$), 37.7 (C-3'), 36.3 (C-1'), 29.7 (C-2'), 28.2 ($C(CH_3)_3$). *m/z* (ESI^-): 346 ($[^{81}BrM{-}H]^-$, 34%), 344 ($[^{79}BrM{-}H]^-$, 34), 270 (100). HRMS (ESI^-): Found $[^{79}BrM{-}H]^-$: 344.0610, $C_{13}H_{19}{}^{79}BrN_3O_3$ requires 344.0615.

***tert*-Butyl (3'-(4,5-dibromo-1*H*-pyrrole-2- carboxamido)propyl)carbamate 28e**

The reaction was carried out following General Procedure **B** using pyrrole **2e** (370 mg, 1 mmol) and amine **26** (0.17 mL, 1 mmol) in THF (2 mL), stirring for 18 h to give the crude product. The crude product was then triturated in CH_2Cl_2 (10 mL) to afford the *title compound* **28e** (258 mg, 61%) as a pale cream solid. m.p. 159–161 °C. ν_{max} (ATR)/cm^{-1} 3348 (NH amine), 3125 (CH aromatic), 2973 (CH aliphatic), 1632 (C=O amide), 1566 (NH amide), 1526 (C=C aromatic), 1365 (CH aliphatic), 1276 (CN aryl), 1159 (C-O ester), 1091 (CN aliphatic), 612 (C-Br). δ_H (400 MHz, $(CD_3)_2SO$) 12.47 (1H, br s, pyrrole NH), 8.04 (1H, t, *J* = 5.7 Hz, amide NH-pyrrole), 6.87 (1H, s, H-3), 6.78 (1H, t, *J* = 5.7 Hz, amide NH-Boc), 3.18 (2H, dt, *J* = 6.0 and 7.0 Hz, H-1'), 2.94 (2H, dt, *J* = 6.0 and 7.0 Hz, H-3'), 1.57 (2H, quint, *J* = 7.0 Hz, H-2'), 1.37 (9H, s, $C(CH_3)3$). δ_C (100 MHz, $(CD_3)_2SO$) 159.0 (C-2-C=O), 155.6 (O=C-O), 128.3 (C-2), 112.3 (C-3), 97.6 (C-4 and C-5), 77.5 ($C(CH_3)_3$), 37.7 (C-3'), 36.3 (C-1'), 29.7 (C-2'), 28.2 ($C(CH_3)_3$). *m/z* (ESI$^-$): 426 ([$^{81}Br_2$M−H]$^-$, 53%), 424 ([$^{81}Br^{79}Br$M−H]$^-$, 100), 422 ([$^{79}Br_2$M−H]$^-$, 56), 350 (18). HRMS (ESI$^-$): Found [$^{81}Br_2$M−H]$^-$: 425.9682, $C_{13}H_{18}{}^{81}Br_2N_3O_3$ requires 425.9682. Found [$^{81}Br^{79}Br$M−H]$^-$: 423.9700, $C_{13}H_{18}{}^{81}Br^{79}BrN_3O_3$ requires 423.9701. Found [$^{79}Br_2$M−H]$^-$: 421.9717, $C_{13}H_{18}{}^{79}Br_2N_3O_3$ requires 421.9720.

***tert*-Butyl (3'-(4-iodo-1*H*-pyrrole-2- carboxamido)propyl)carbamate 28f**

The reaction was carried out following General Procedure **B** using pyrrole **2f** (338 mg, 1 mmol) and amine **26** (0.17 mL, 1 mmol) in THF (2 mL), stirring for 18 h to give the crude product. The crude product was then triturated with CH_2Cl_2 (10 mL) to afford the *title compound* **28f** (82 mg, 21%) as an off-white solid. m.p. 128–130 °C. ν_{max} (ATR)/cm^{-1} 3333 (NH amine), 3198 (CH aromatic), 2975 (CH aliphatic), 1623 (C=O amide), 1567 (NH amide), 1514 (C=C aromatic), 1366 (CH aliphatic), 1252 (CN aryl), 1162 (C-O ester), 1042 (CN aliphatic), 603 (C-I). δ_H (400 MHz, $(CD_3)_2SO$) 11.75 (1H, br s, pyrrole NH), 8.01 (1H, t, *J* = 5.7 Hz, amide NH-pyrrole), 6.98–6.95 (1H, m, H-5), 6.88–6.85 (1H, m, H-3), 6.78 (1H, t, *J* = 5.7 Hz, amide

NH-Boc), 3.18 (2H, dt, *J* = 6.0 and 7.0 Hz, H-1'), 2.94 (2H, dt, *J* = 6.0 and 7.0 Hz, H-3'), 1.57 (2H, quint, *J* = 7.0 Hz, H-2'), 1.37 (9H, s, $C(CH_3)_3$). δ_C (100 MHz, $(CD_3)_2SO$) 159.4 (C-2-C=O), 155.5 (O=C-O), 128.4 (C-2), 126.0 (C-5), 116.0 (C-3), 77.5 ($C(CH_3)_3$), 60.3 (C-4), 37.7 (C-3'), 36.2 (C-1'), 29.7 (C-2'), 28.2 ($C(CH_3)_3$). *m/z* (ESI^-): 392 ($[M-H]^-$, 51%), 318 (100). HRMS (ESI^-): Found $[M-H]^-$: 392.0475, $C_{13}H_{19}IN_3O_3$ requires 392.0477.

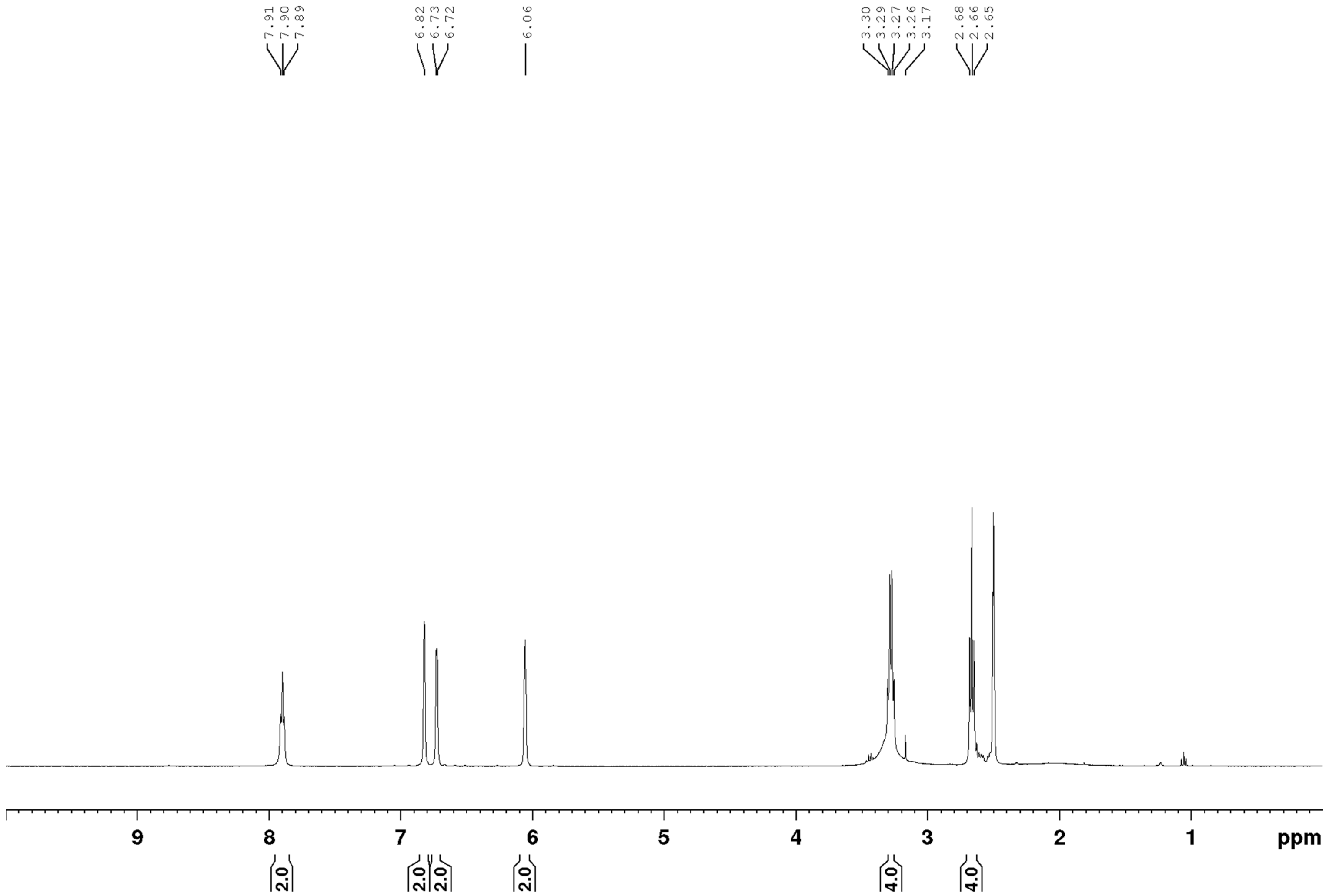

Figure x: ^{1}H NMR spectra of **13a** (400 MHz; DMSO-d_6).

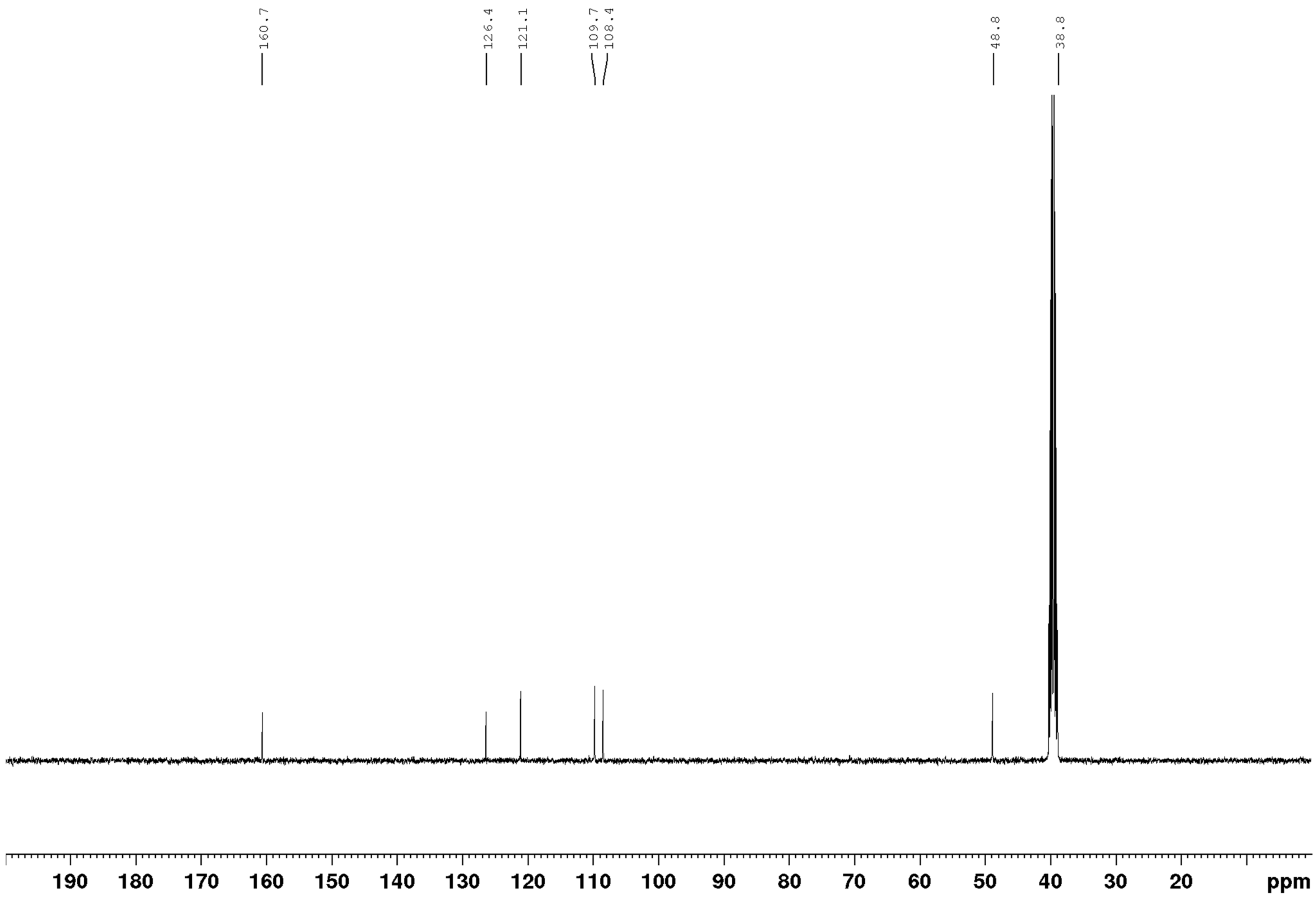

Figure x: ^{13}C NMR spectra of **13a** (100 MHz; DMSO-d_6).

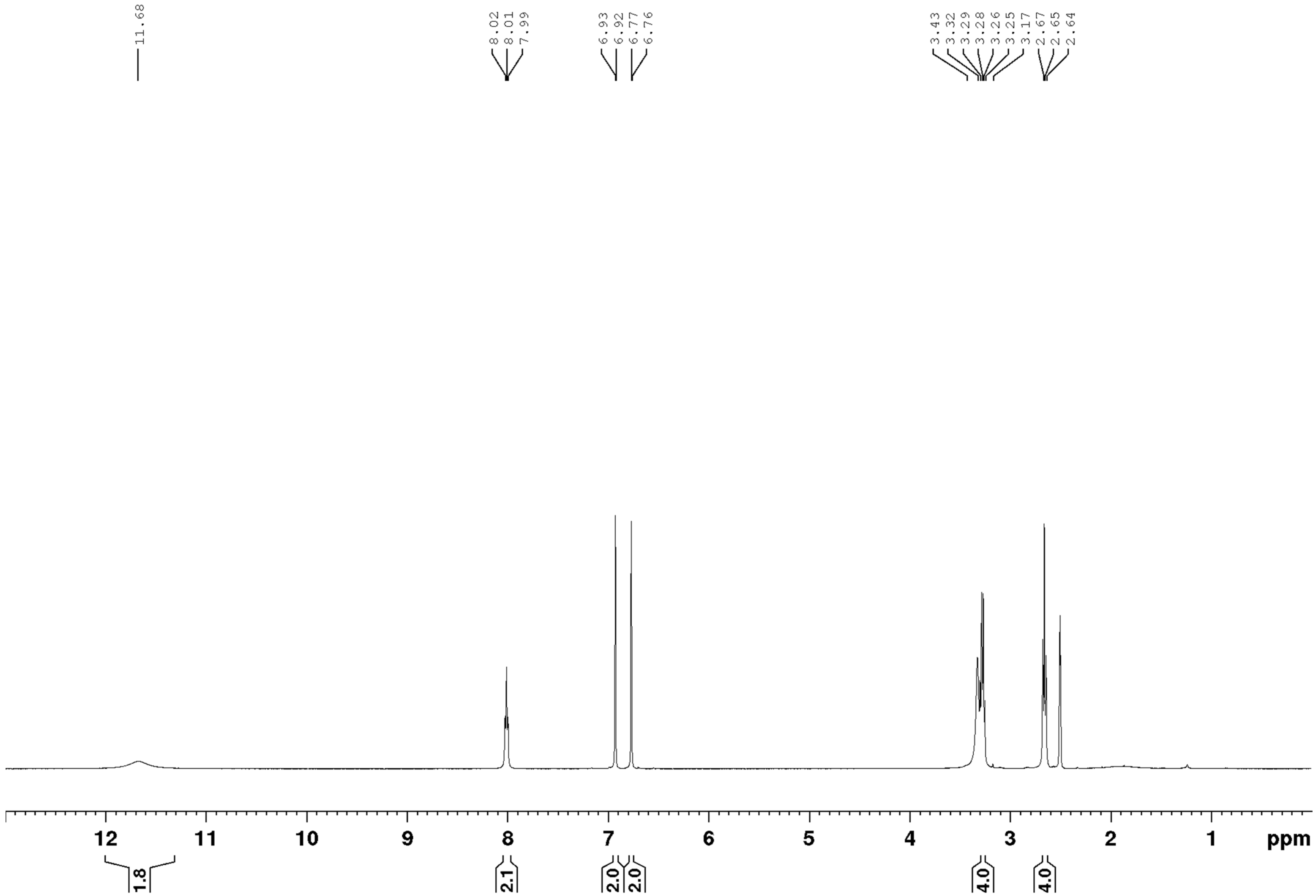

Figure x: ^{1}H NMR spectra of **13b** (400 MHz; DMSO-d_6).

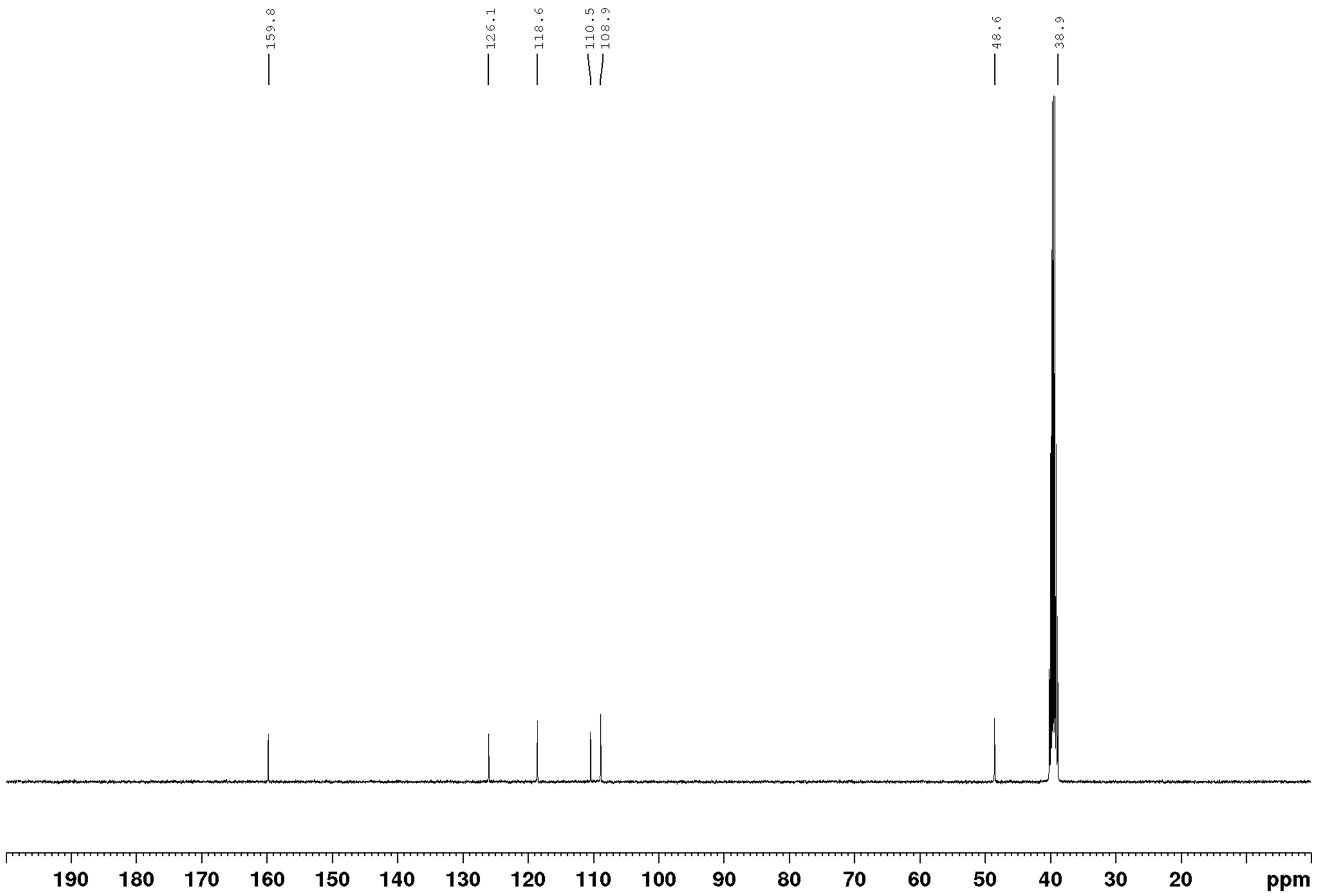

Figure x: ^{13}C NMR spectra of **13b** (100 MHz; DMSO-d_6).

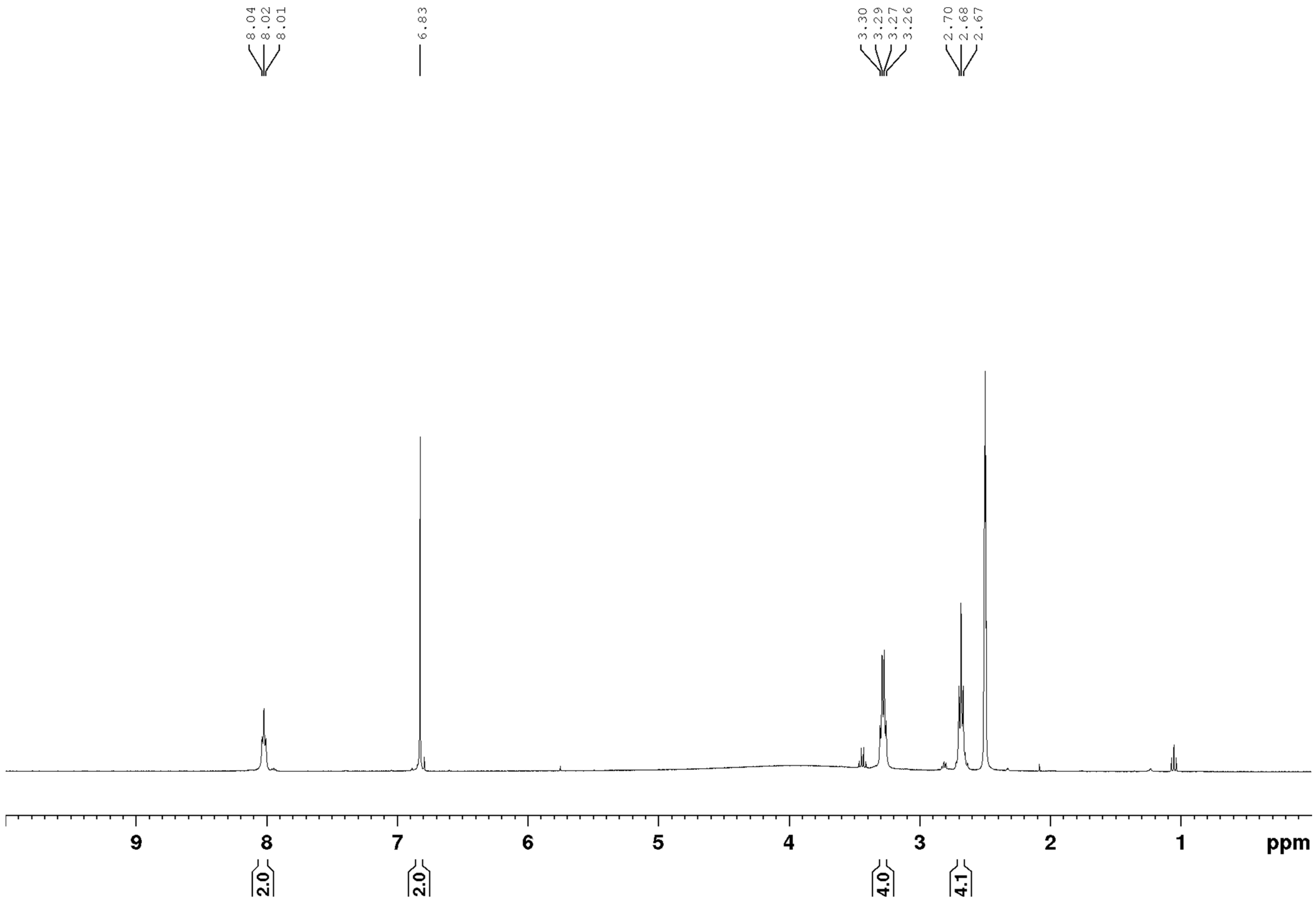

Figure x: ^{1}H NMR spectra of **13c** (400 MHz; DMSO-d_6).

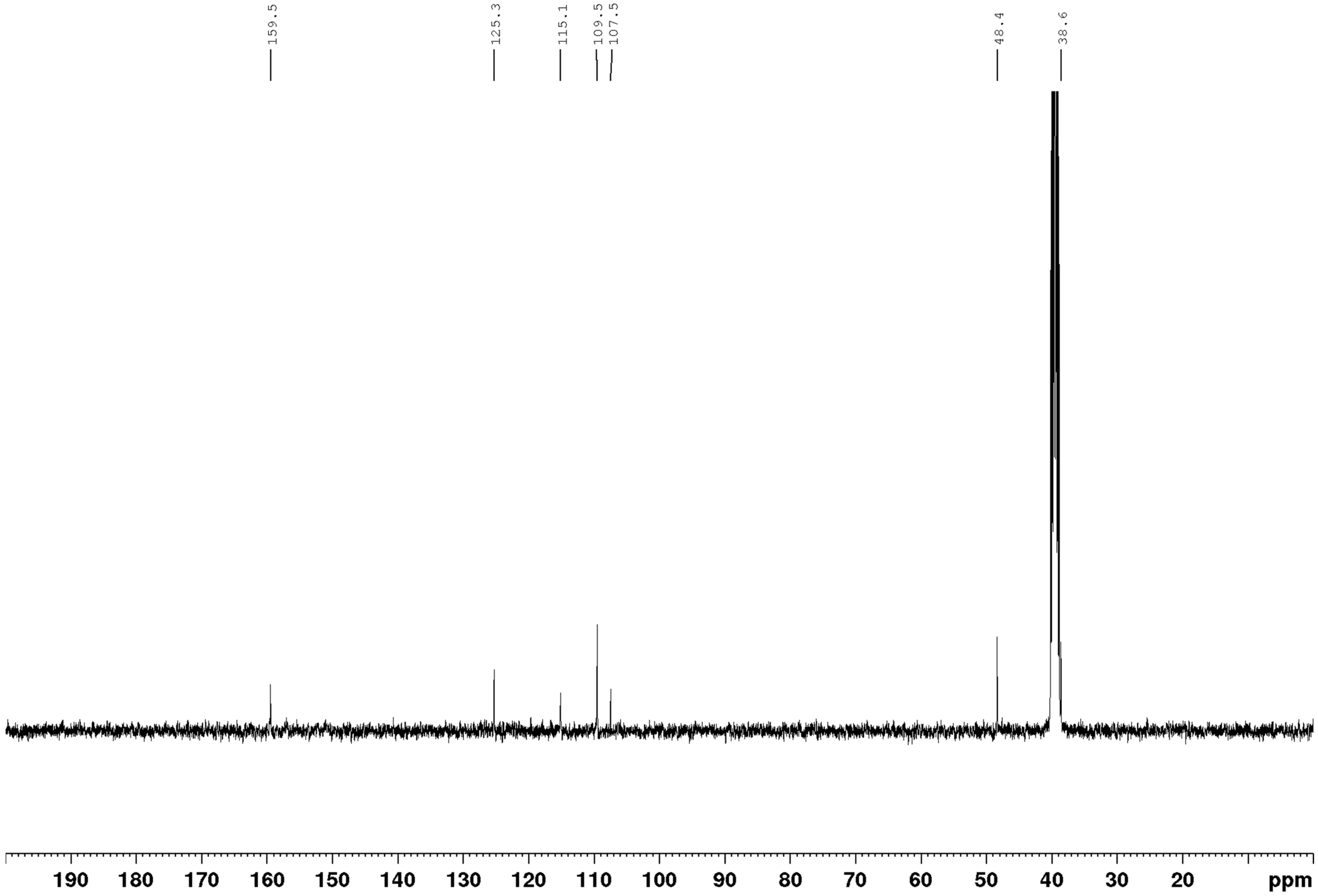

Figure x: ^{13}C NMR spectra of **13c** (100 MHz; DMSO-d_6).

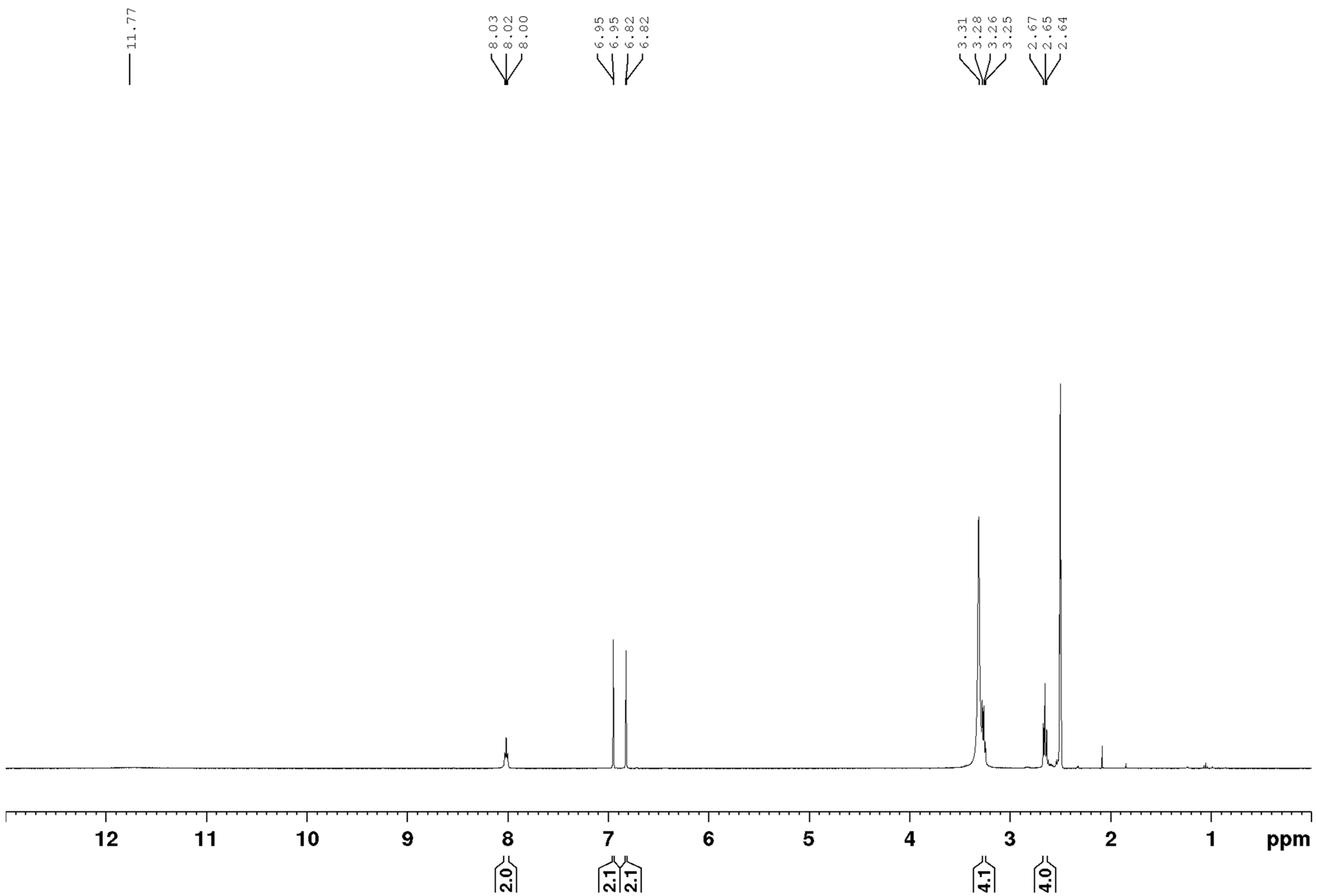

Figure x: ^{1}H NMR spectra of **13d** (400 MHz; DMSO-d_6).

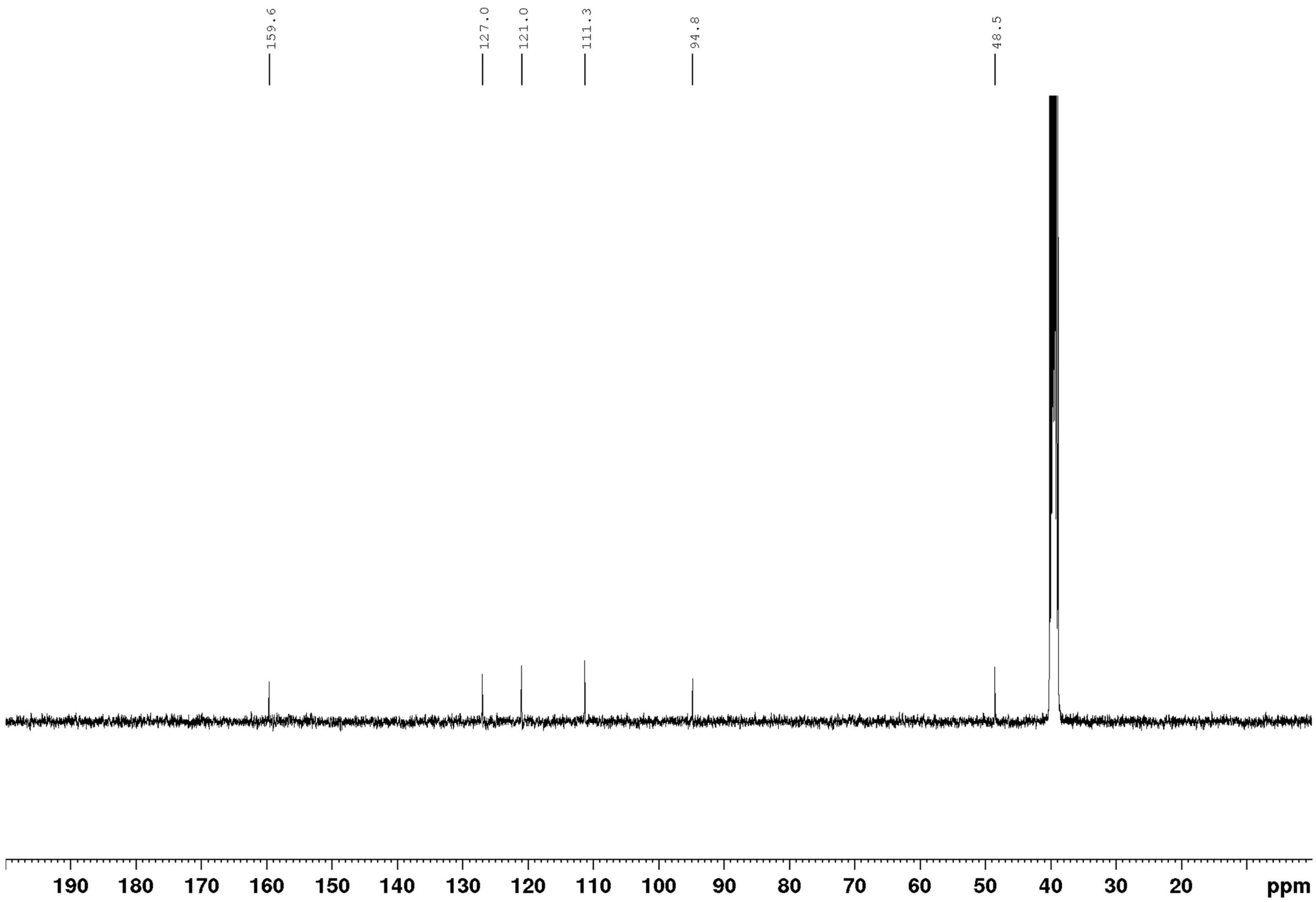

Figure x: ^{13}C NMR spectra of **13d** (100 MHz; DMSO-d_6).

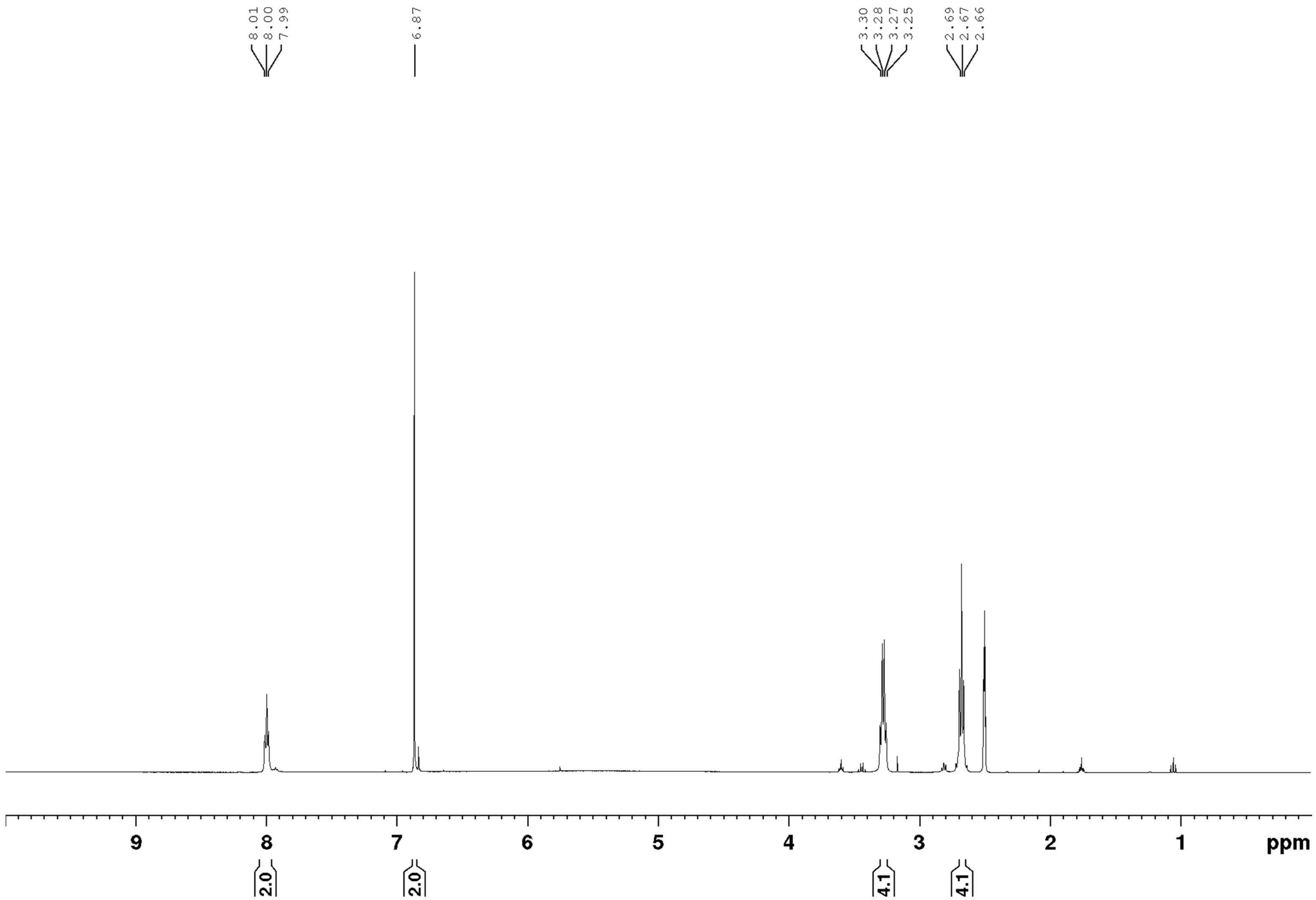

Figure x: ^{1}H NMR spectra of **13e** (400 MHz; DMSO-d_6).

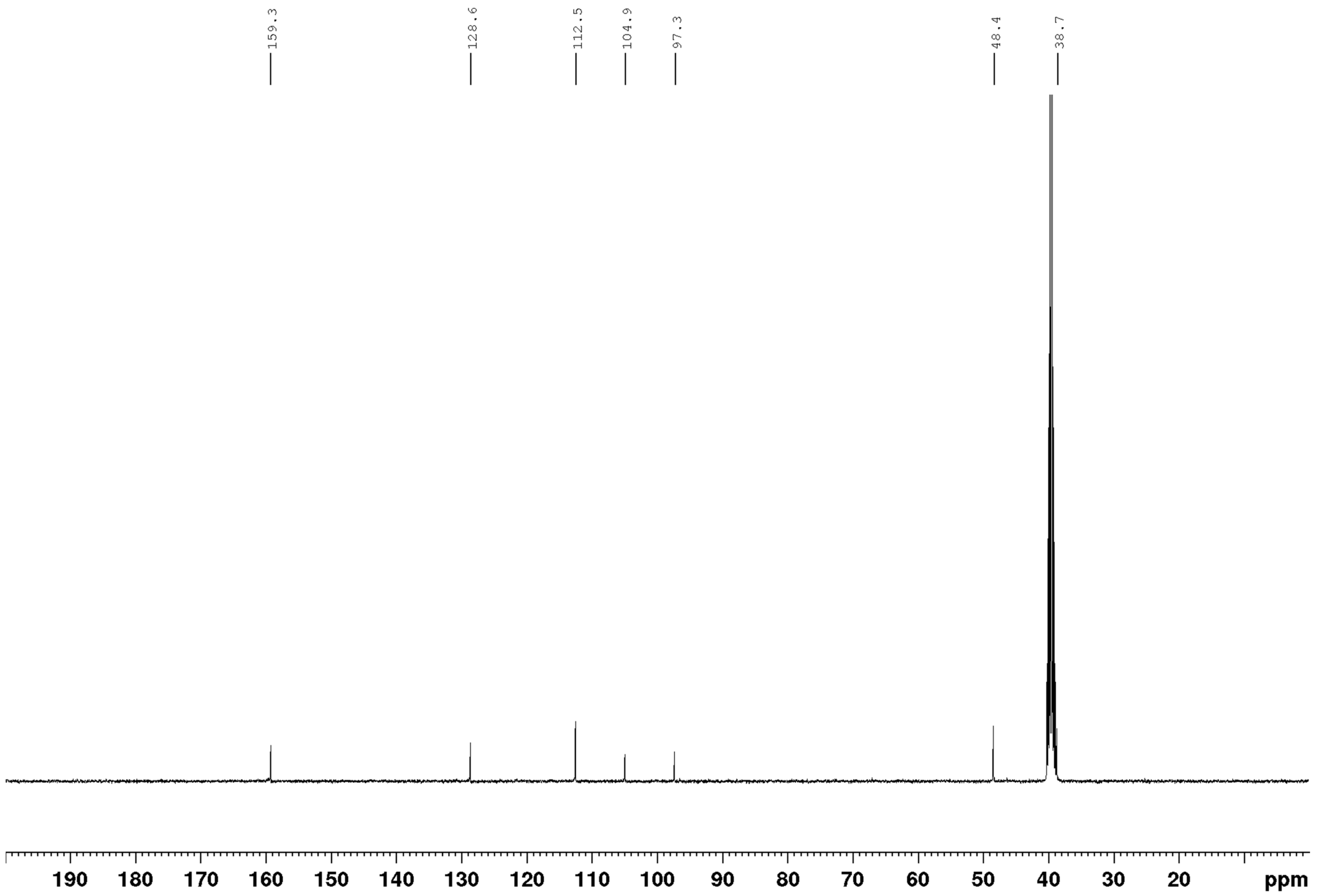

Figure x: ^{13}C NMR spectra of **13e** (100 MHz; DMSO-*d*$_6$).

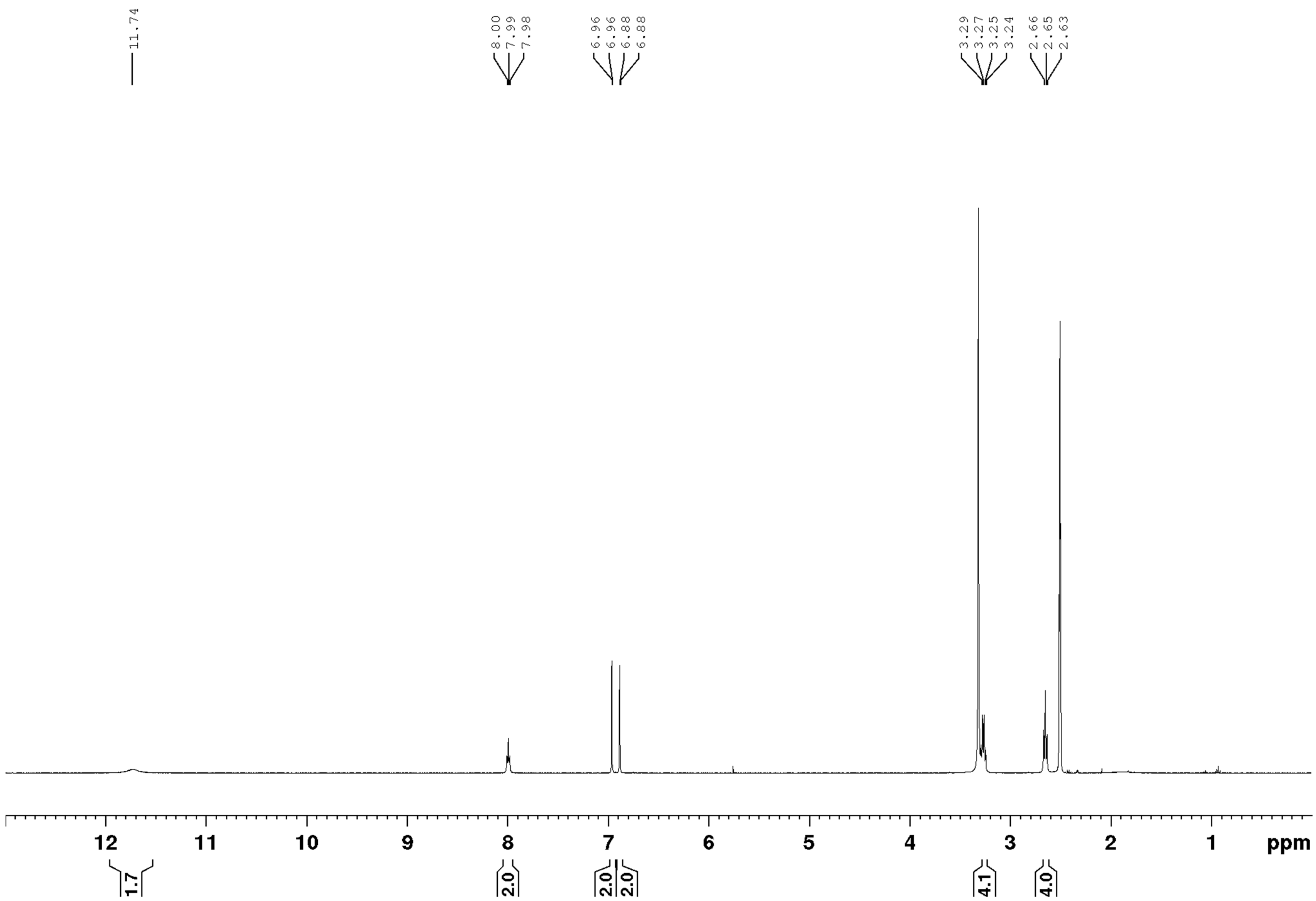

Figure x: ^{1}H NMR spectra of **13f** (400 MHz; DMSO-d_6).

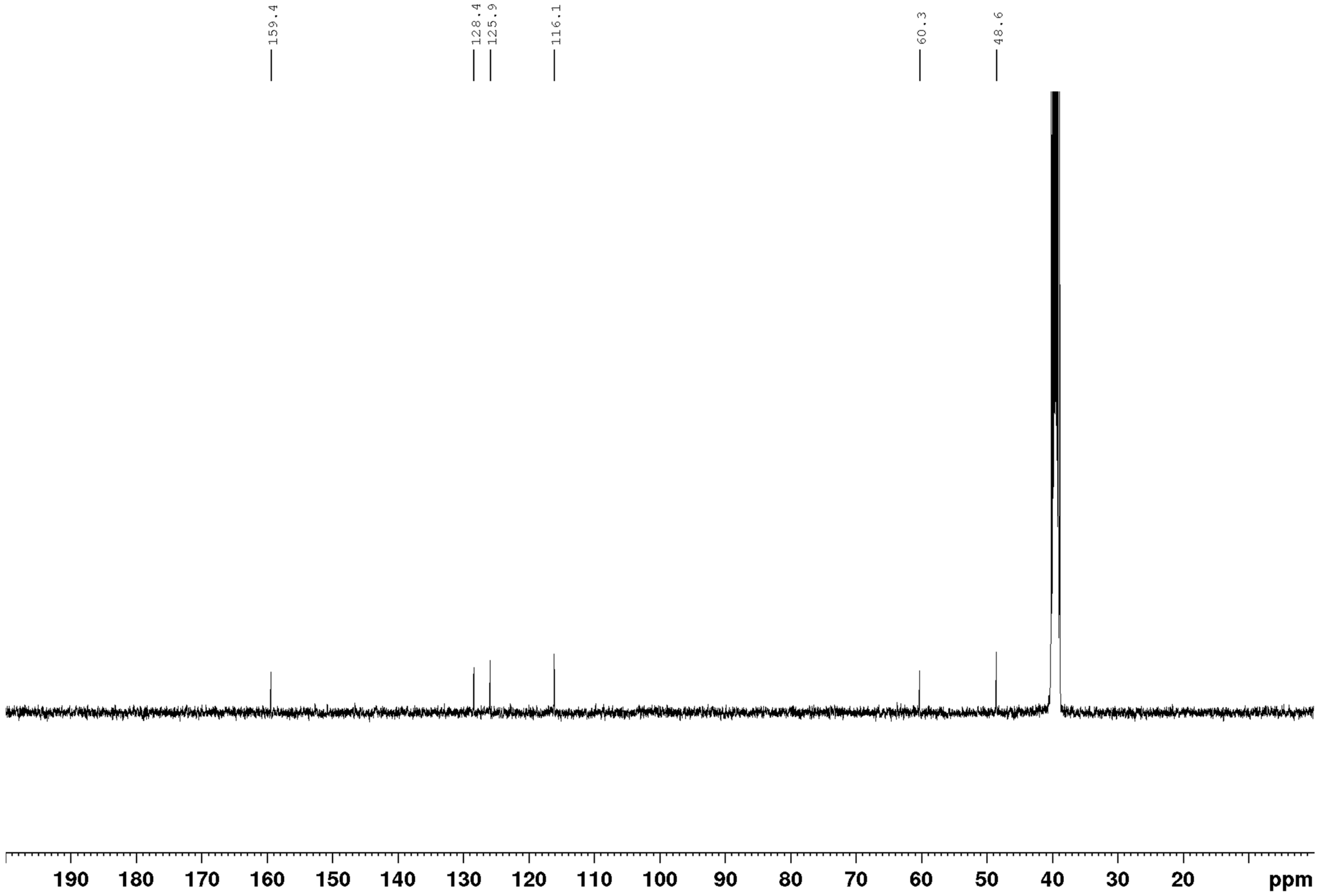

Figure x: ^{13}C NMR spectra of **13f** (100 MHz; DMSO-d_6).

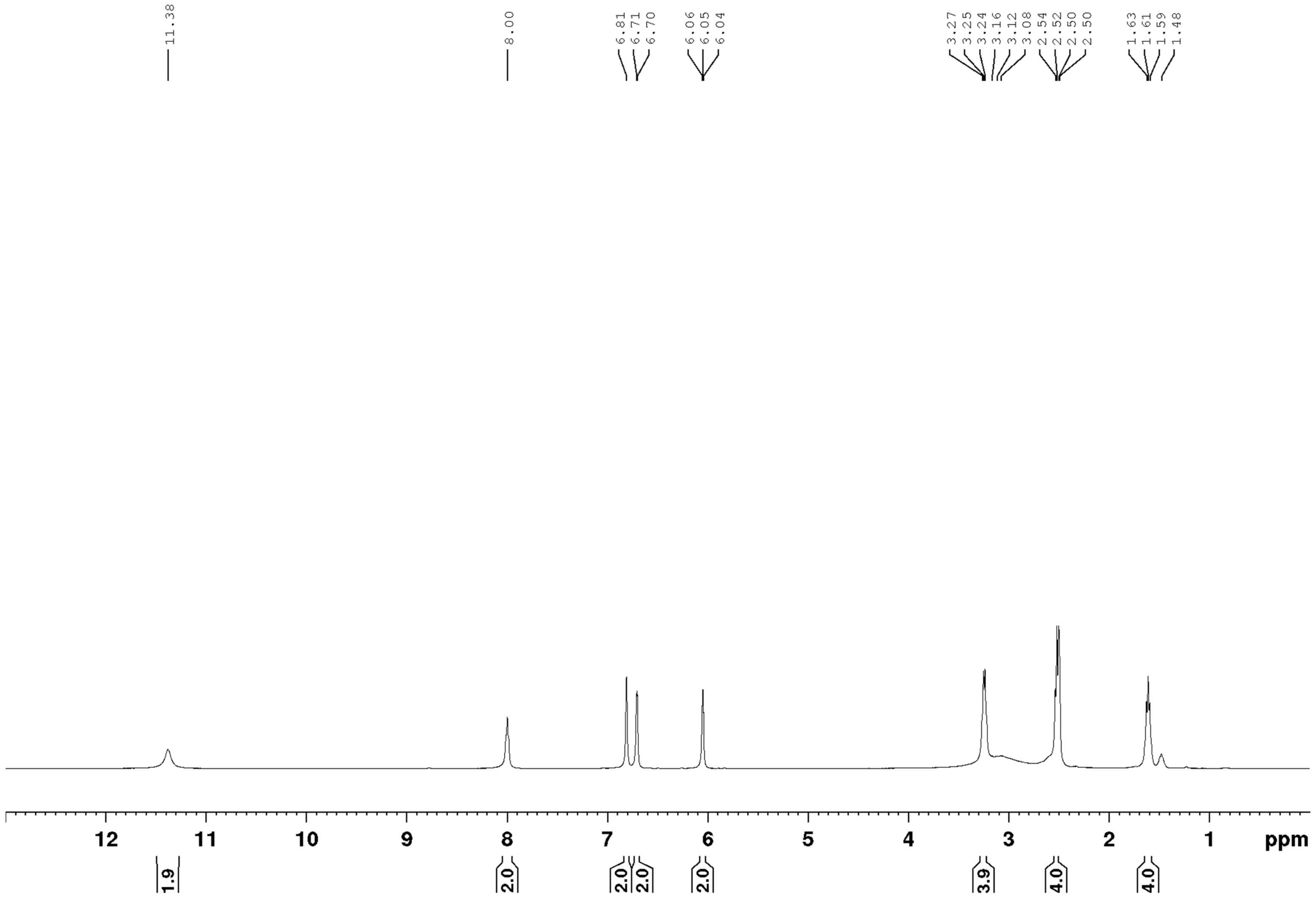

Figure x: ^{1}H NMR spectra of **14a** (400 MHz; DMSO-d_6).

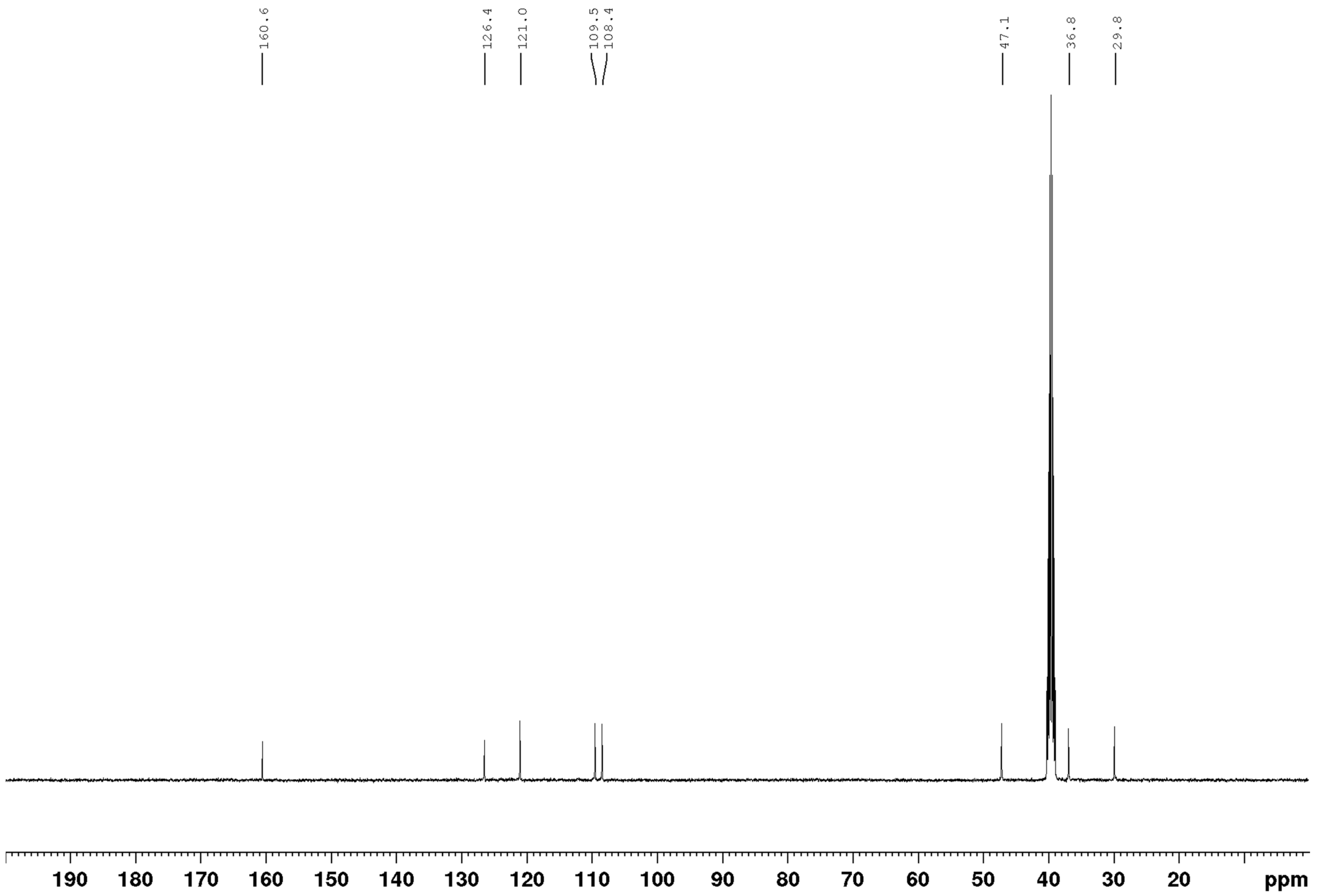

Figure x: ^{13}C NMR spectra of **14a** (100 MHz; DMSO-d_6).

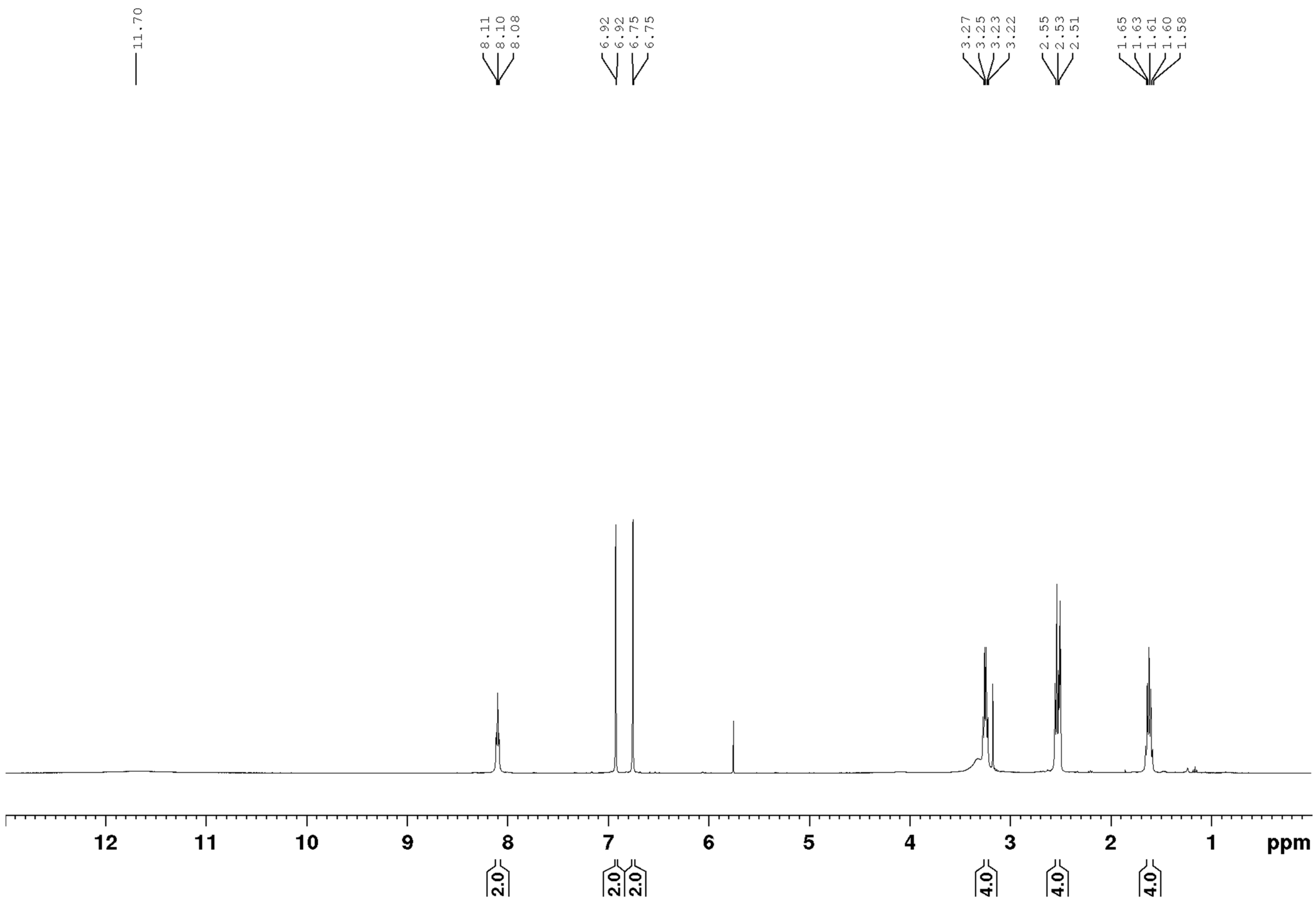

Figure x: ^{1}H NMR spectra of **14b** (400 MHz; DMSO-d_6).

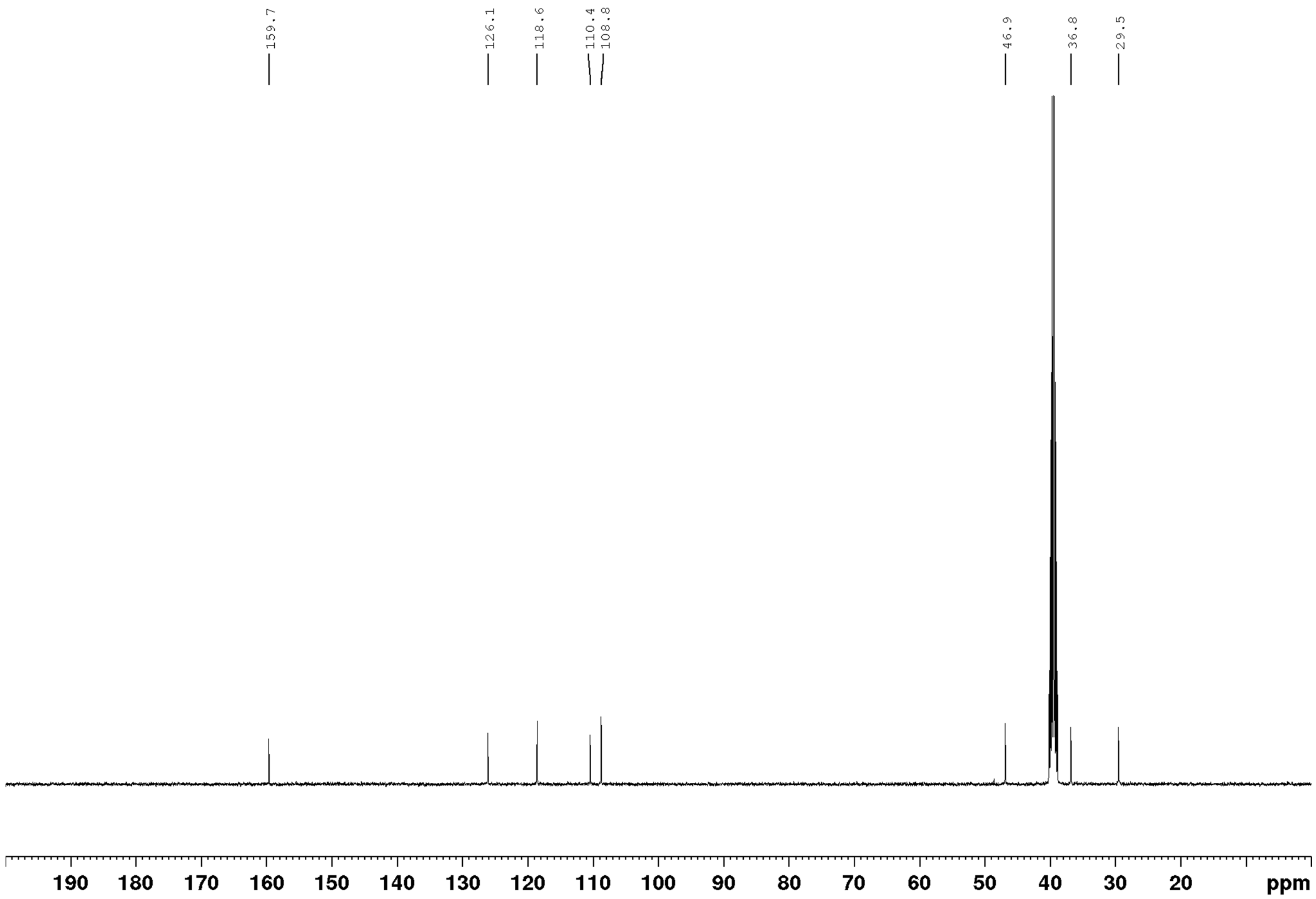

Figure x: ^{13}C NMR spectra of **14b** (100 MHz; DMSO-d_6).

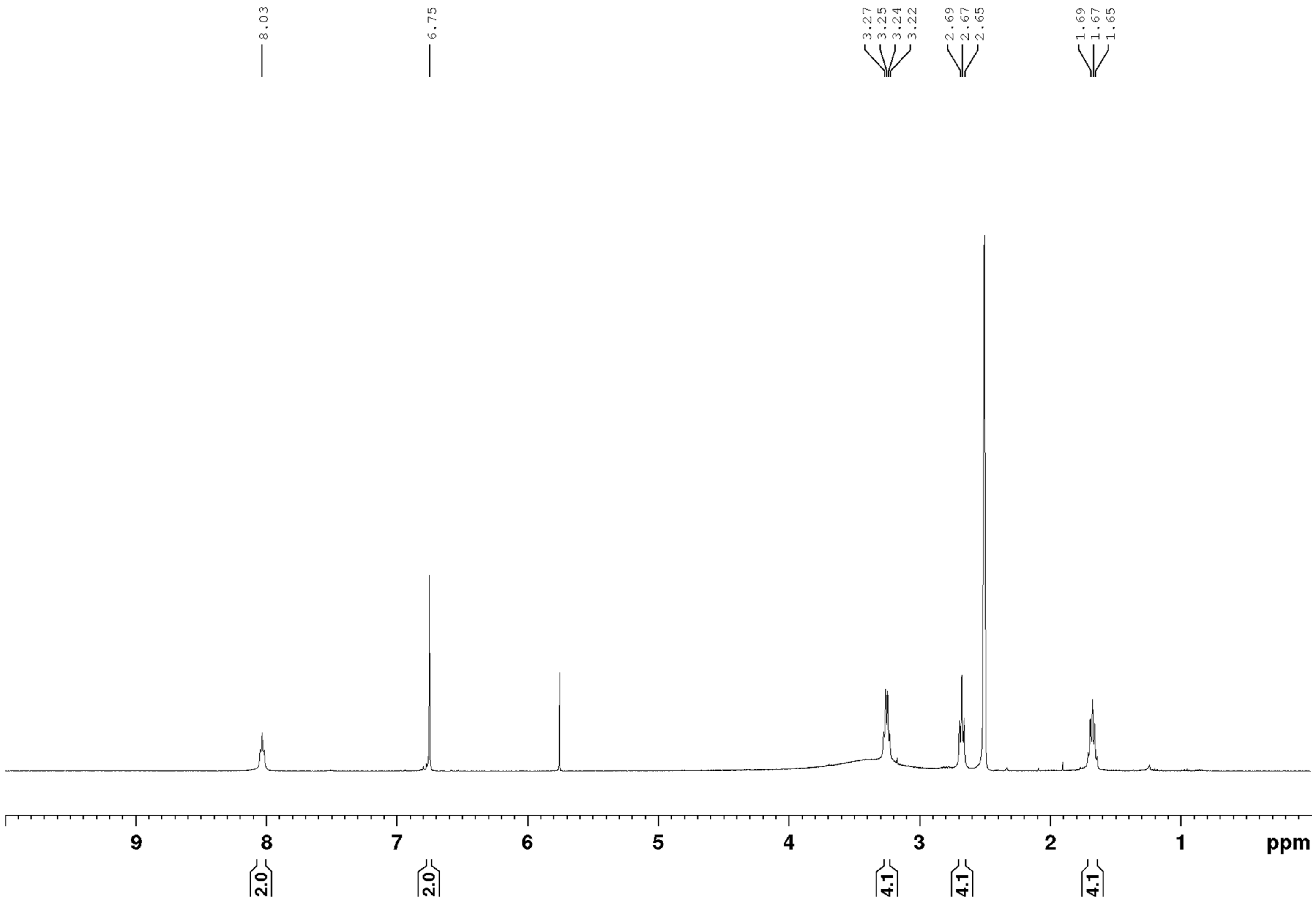

Figure x: ^{1}H NMR spectra of **14c** (400 MHz; DMSO-d_6).

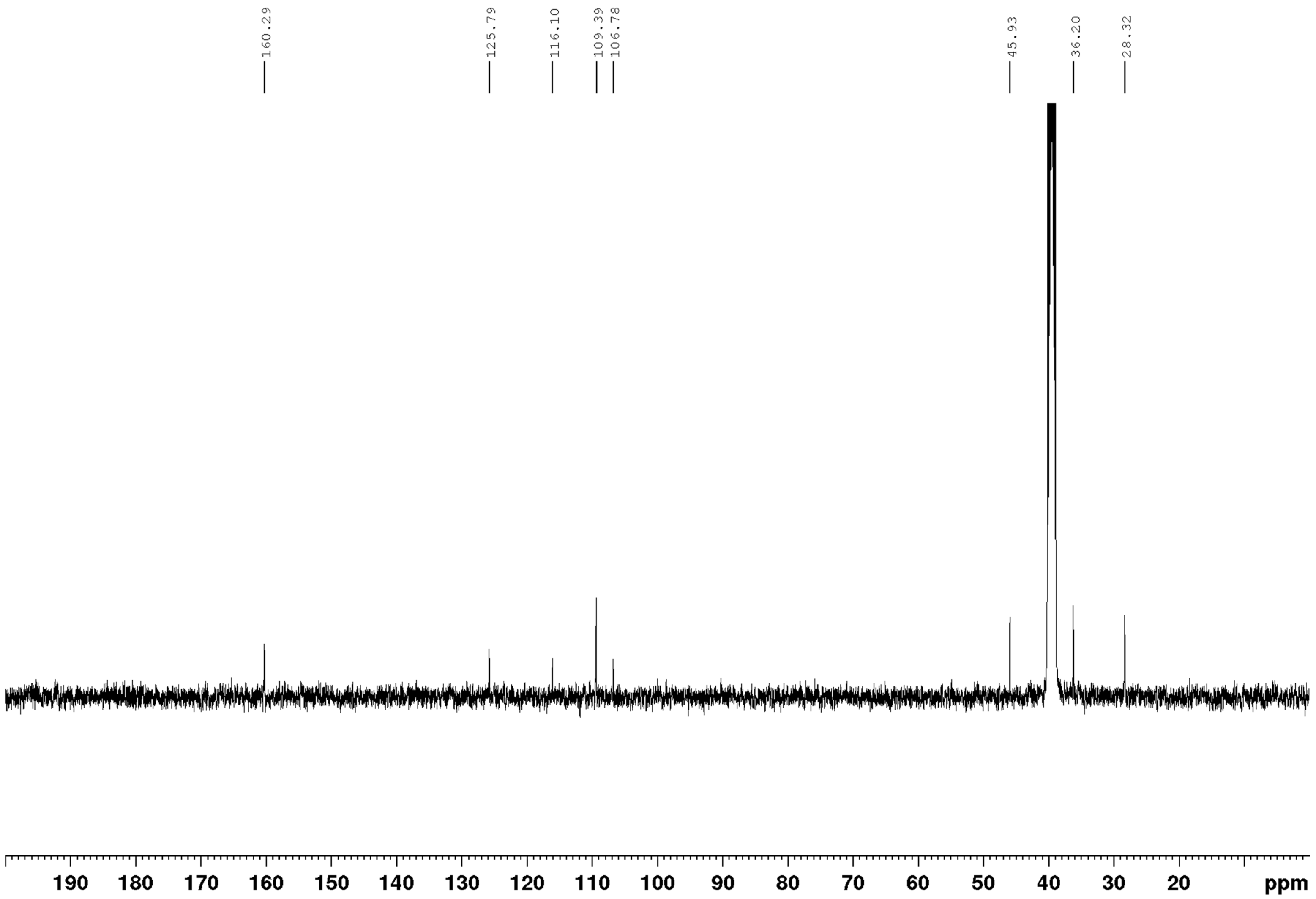

Figure x: ^{13}C NMR spectra of **14c** (100 MHz; DMSO-d_6).

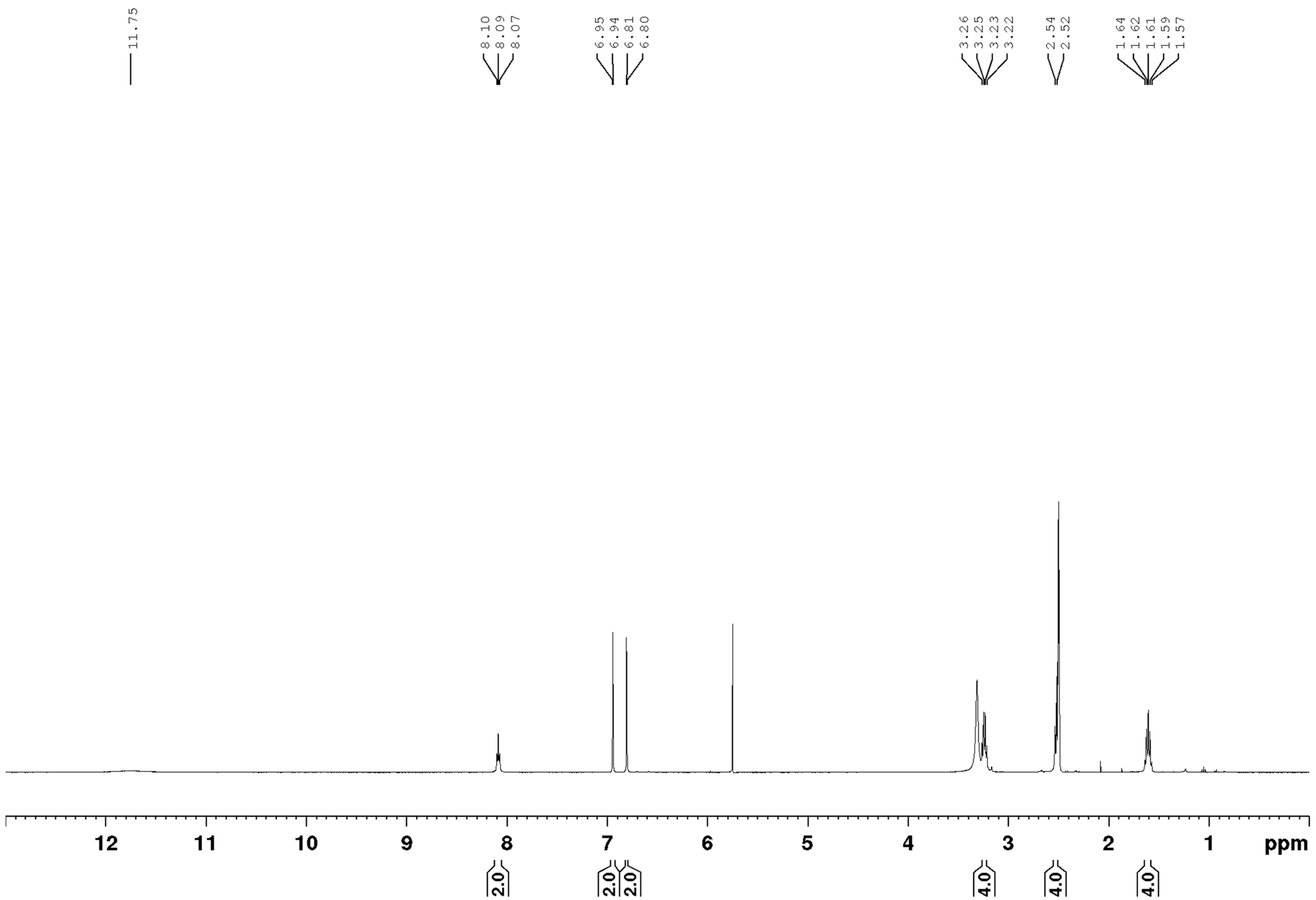

Figure x: ^{1}H NMR spectra of **14d** (400 MHz; DMSO-d_6).

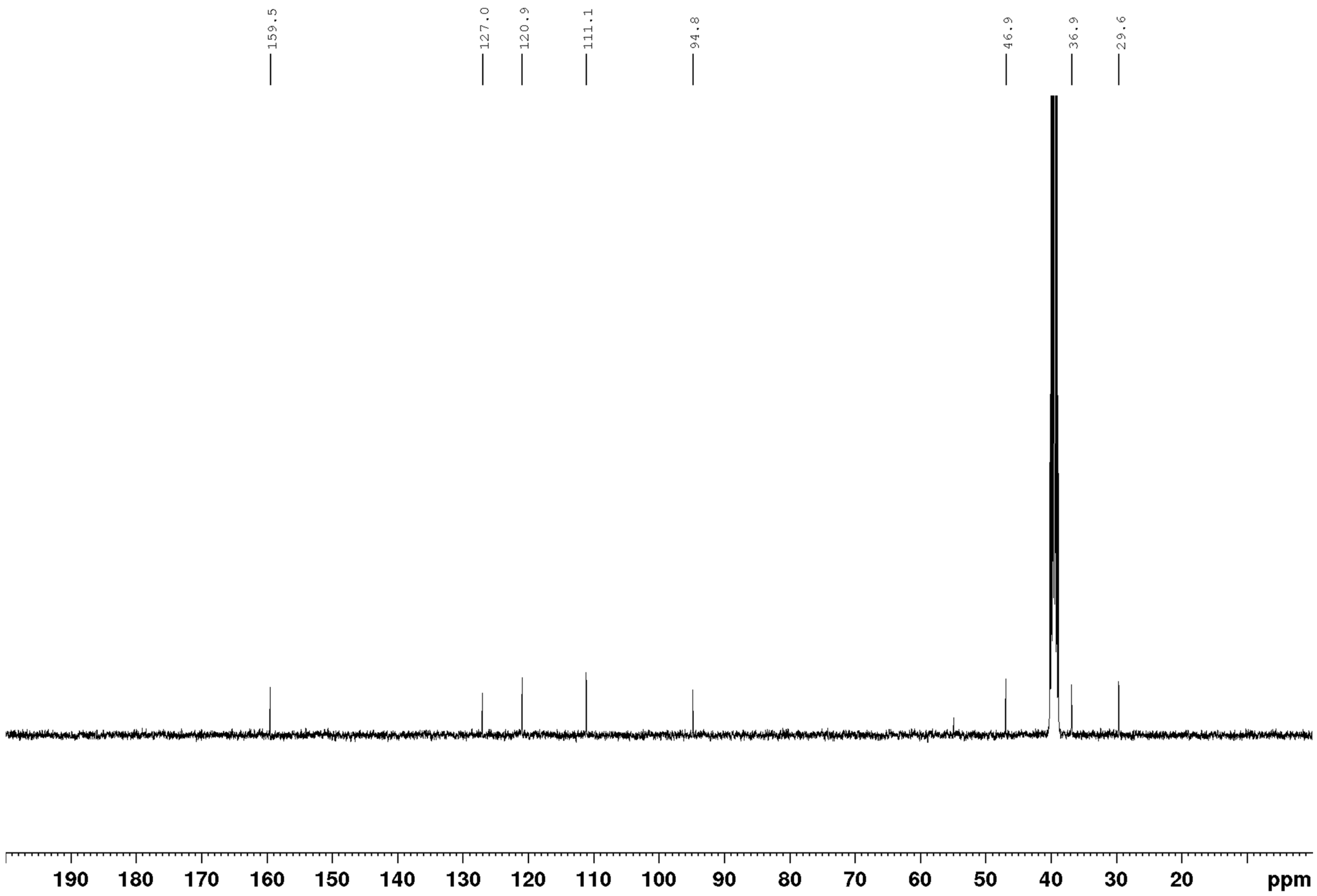

Figure x: ^{13}C NMR spectra of **14d** (100 MHz; DMSO-d_6).

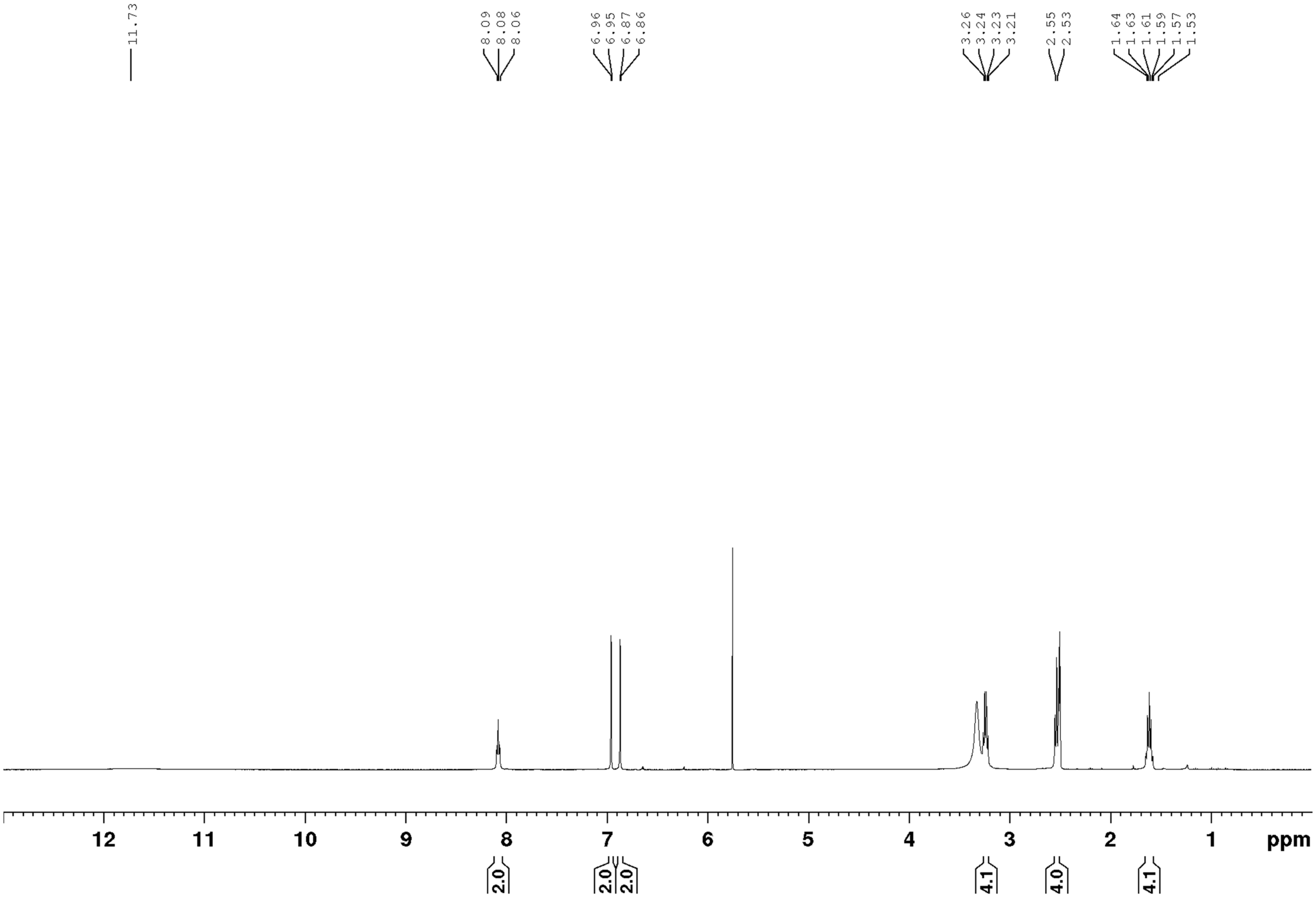

Figure x: ^{1}H NMR spectra of **14f** (400 MHz; DMSO-d_6).

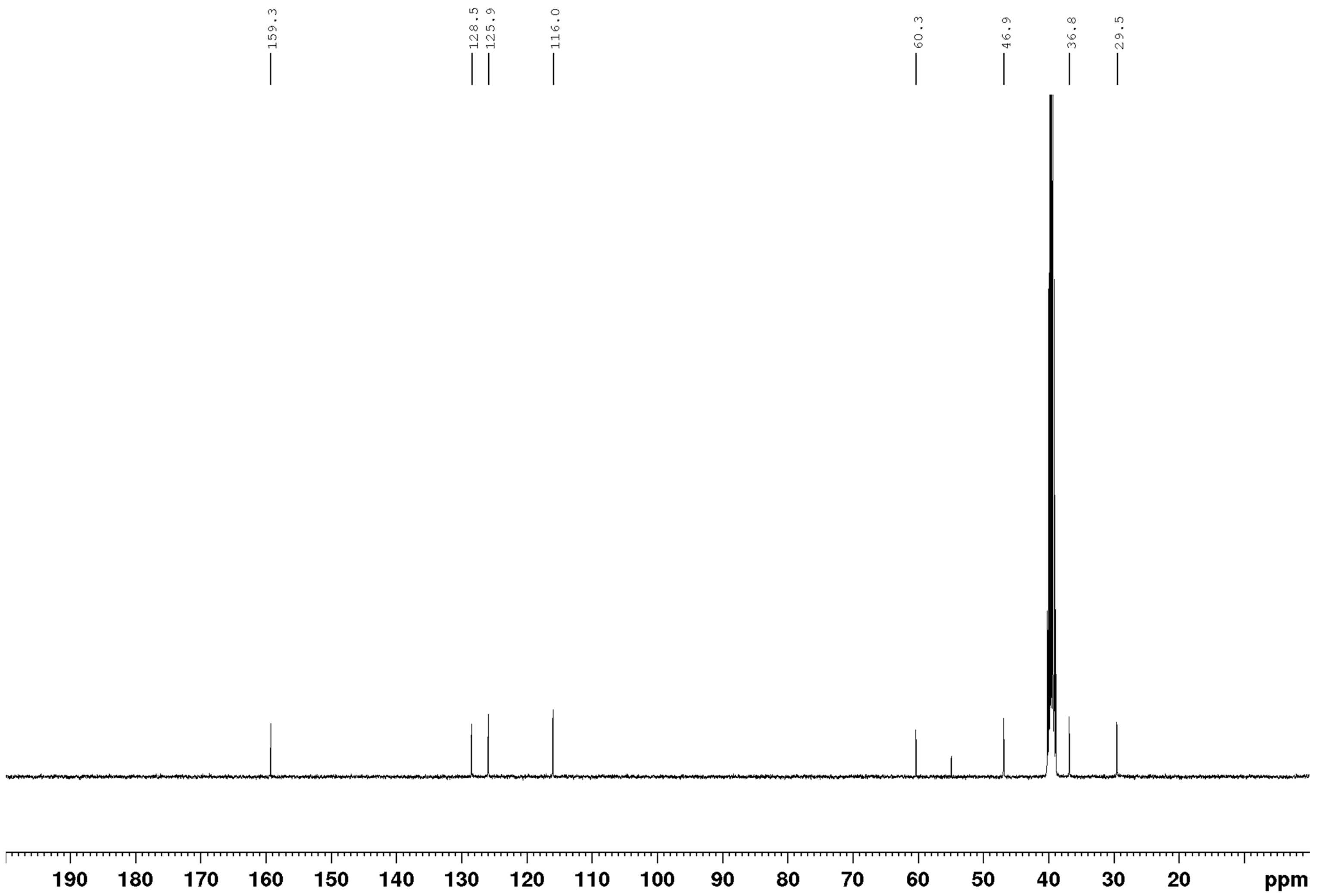

Figure x: ^{13}C NMR spectra of **14f** (100 MHz; DMSO-d_6).

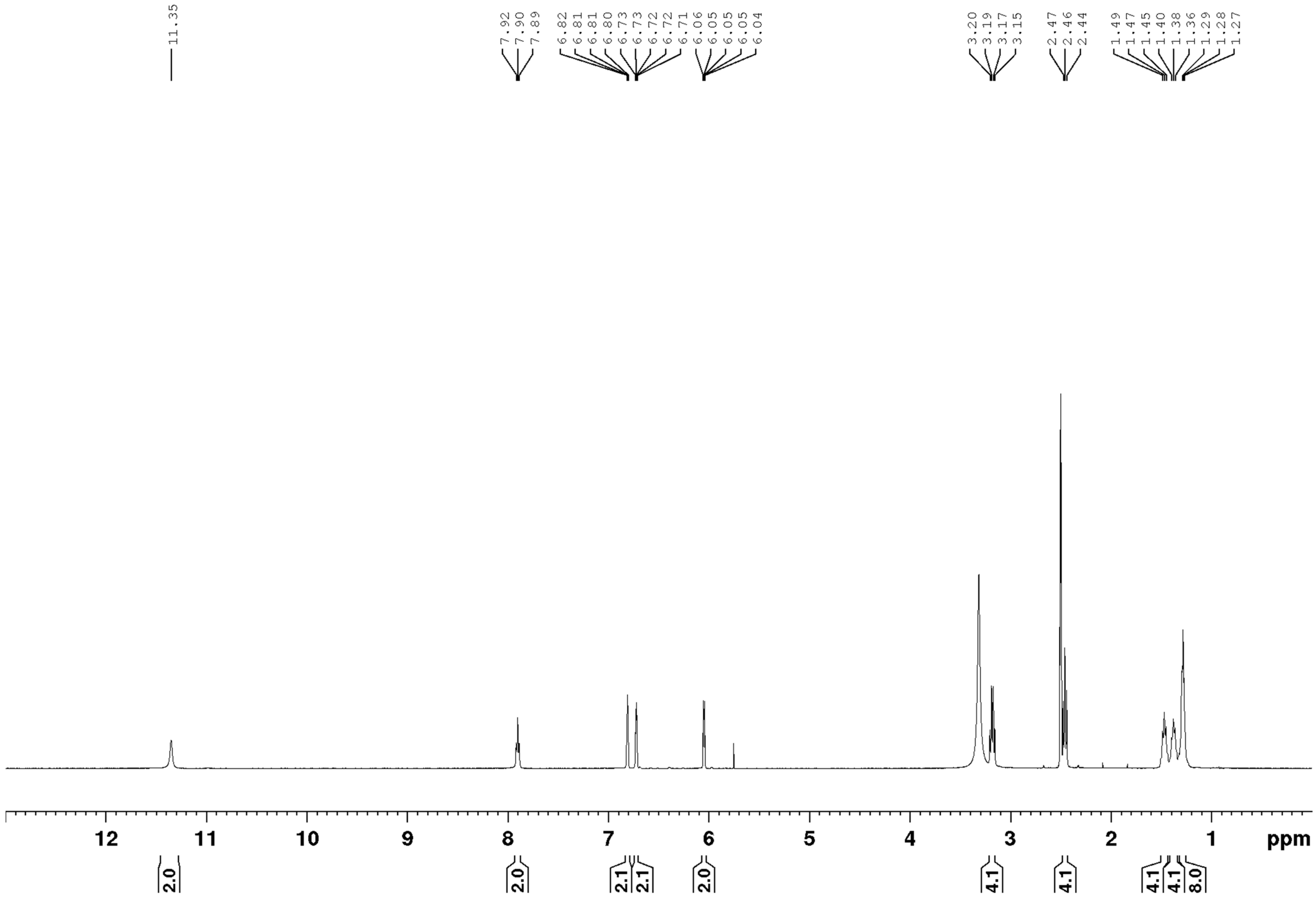

Figure x: ^{1}H NMR spectra of **15a** (400 MHz; DMSO-d_6).

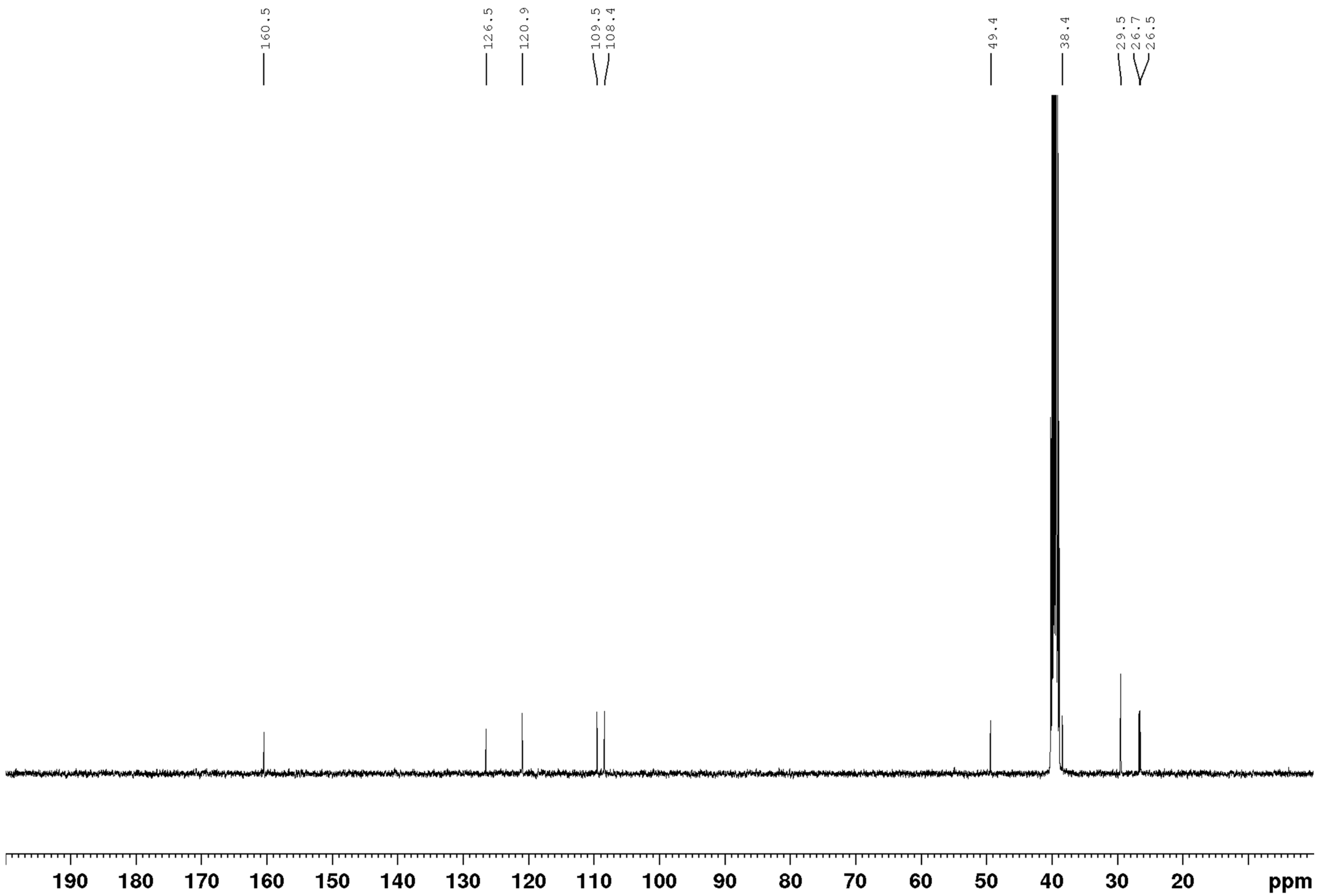

Figure x: ^{13}C NMR spectra of **15a** (100 MHz; DMSO-d_6).

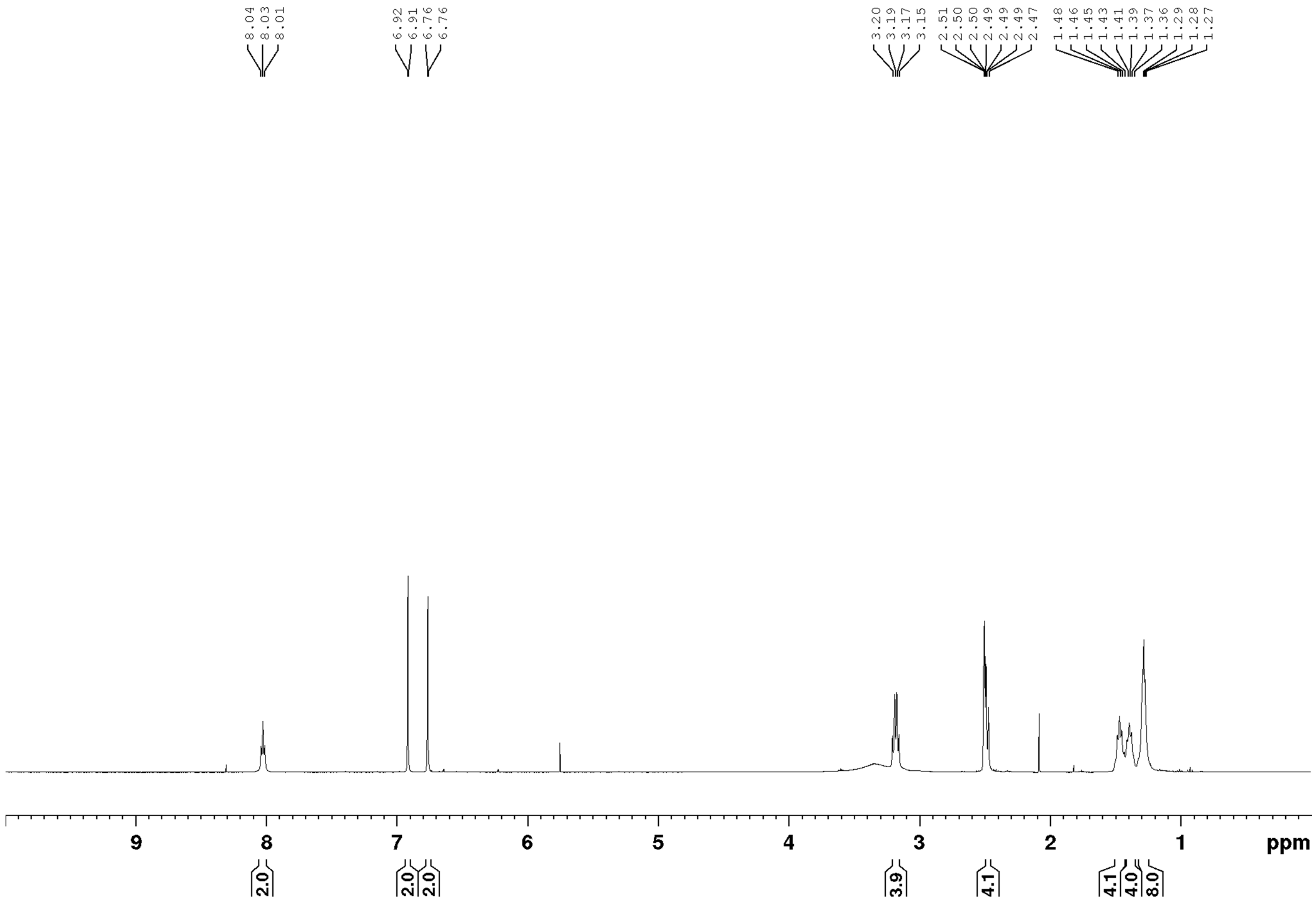

Figure x: ^{1}H NMR spectra of **15b** (400 MHz; DMSO-d_6).

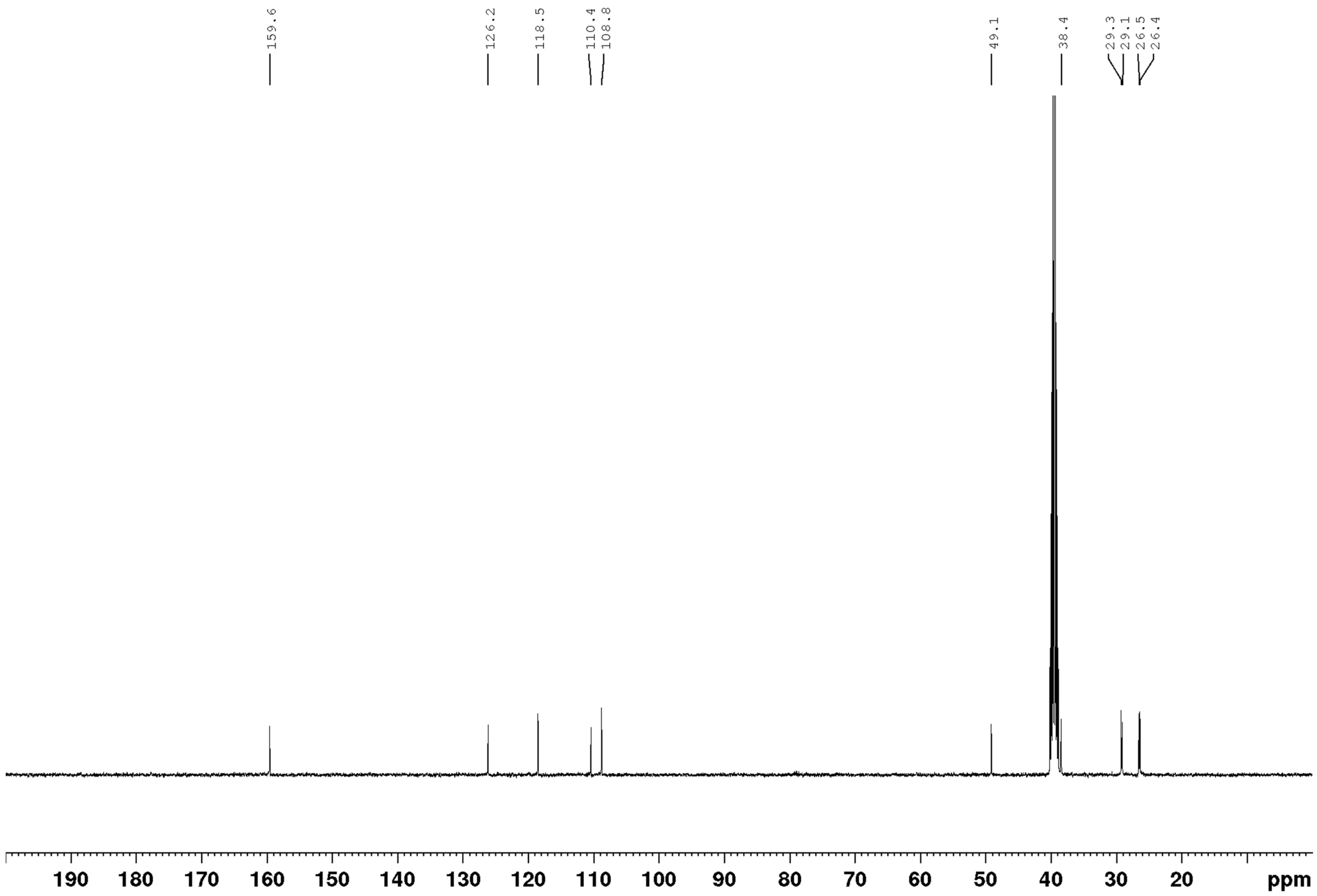

Figure x: ^{13}C NMR spectra of **15b** (100 MHz; DMSO-d_6).

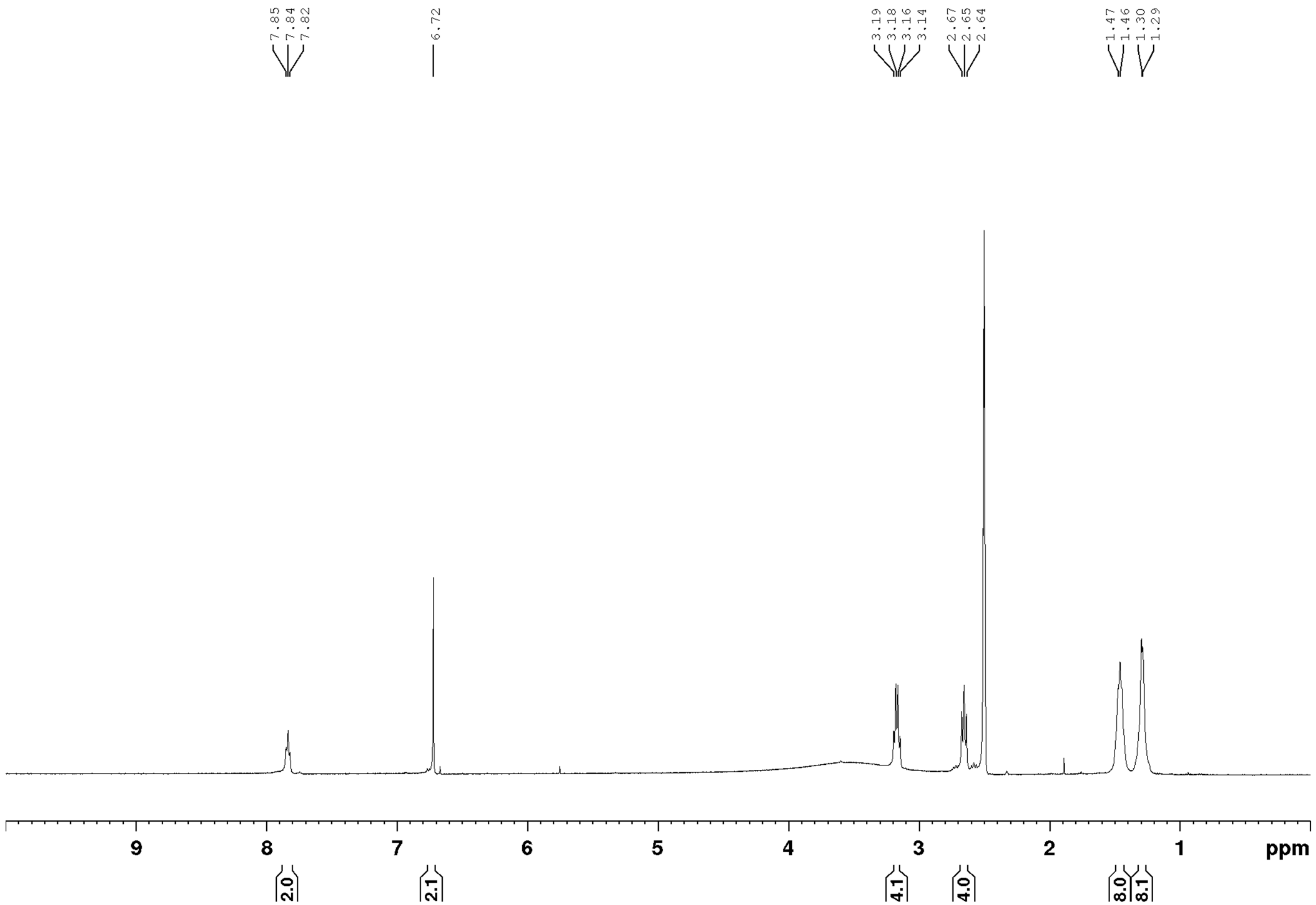

Figure x: ^{1}H NMR spectra of **15c** (400 MHz; DMSO-d_6).

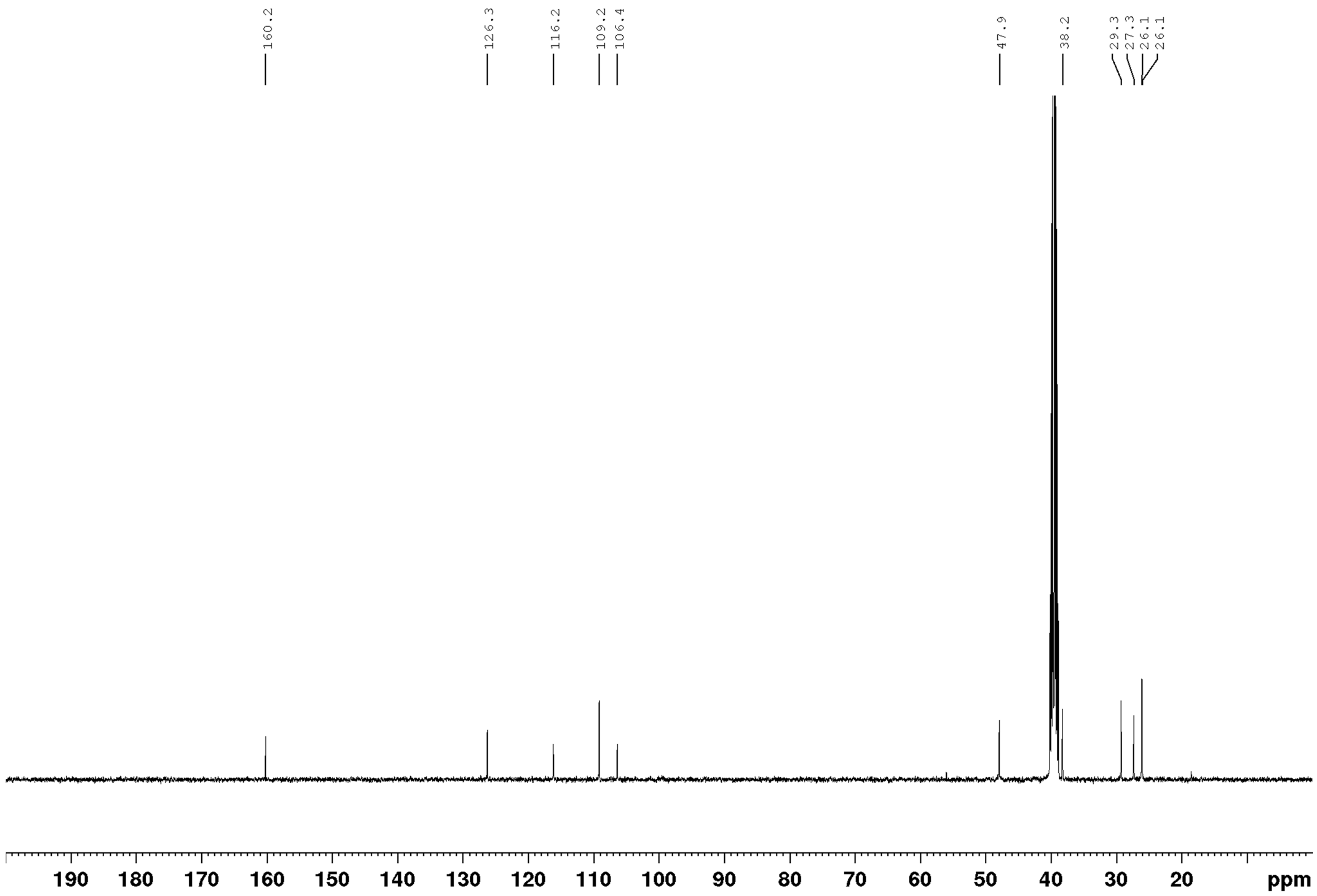

Figure x: ^{13}C NMR spectra of **15c** (100 MHz; DMSO-d_6).

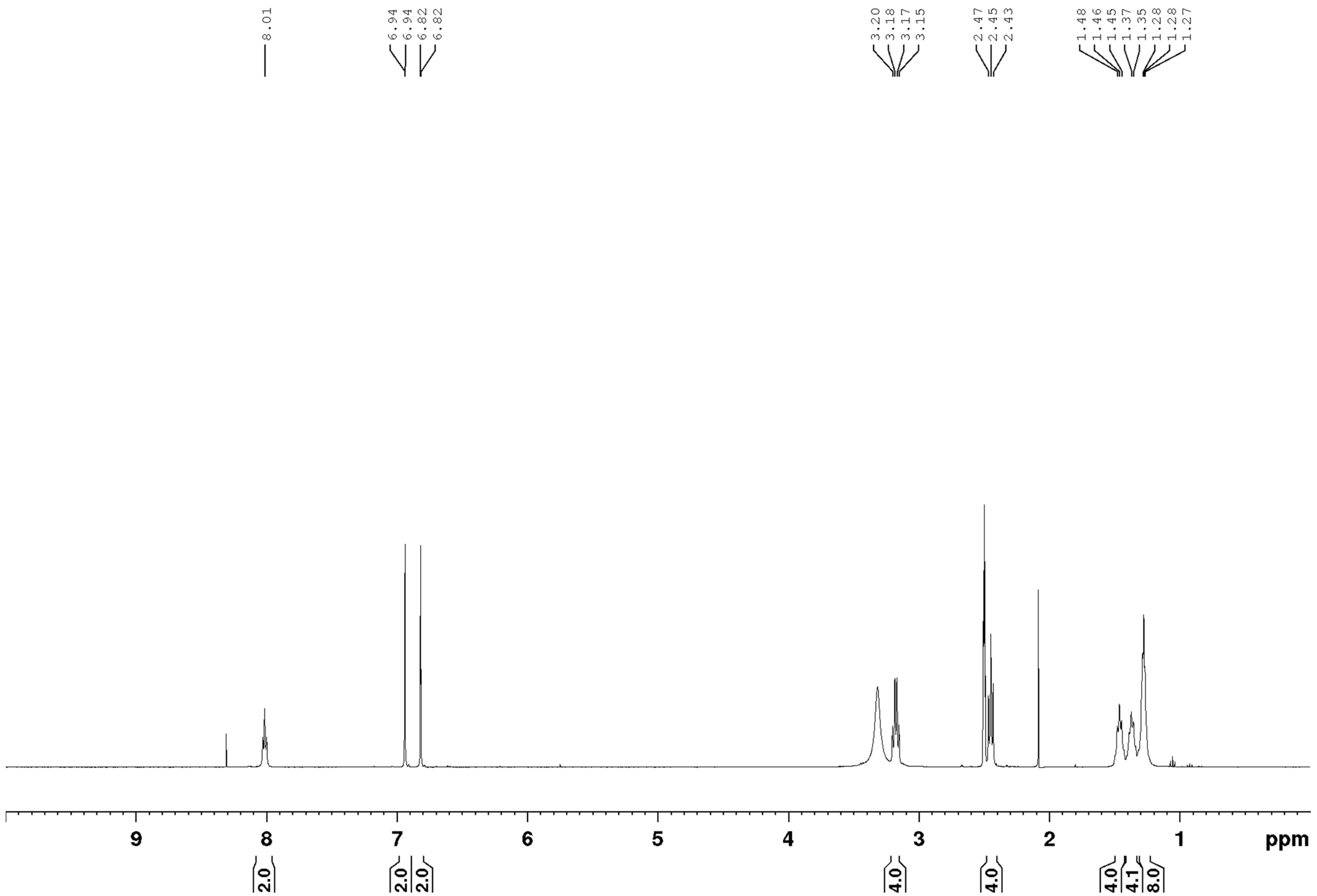

Figure x: ^{1}H NMR spectra of **15d** (400 MHz; DMSO-d_6).

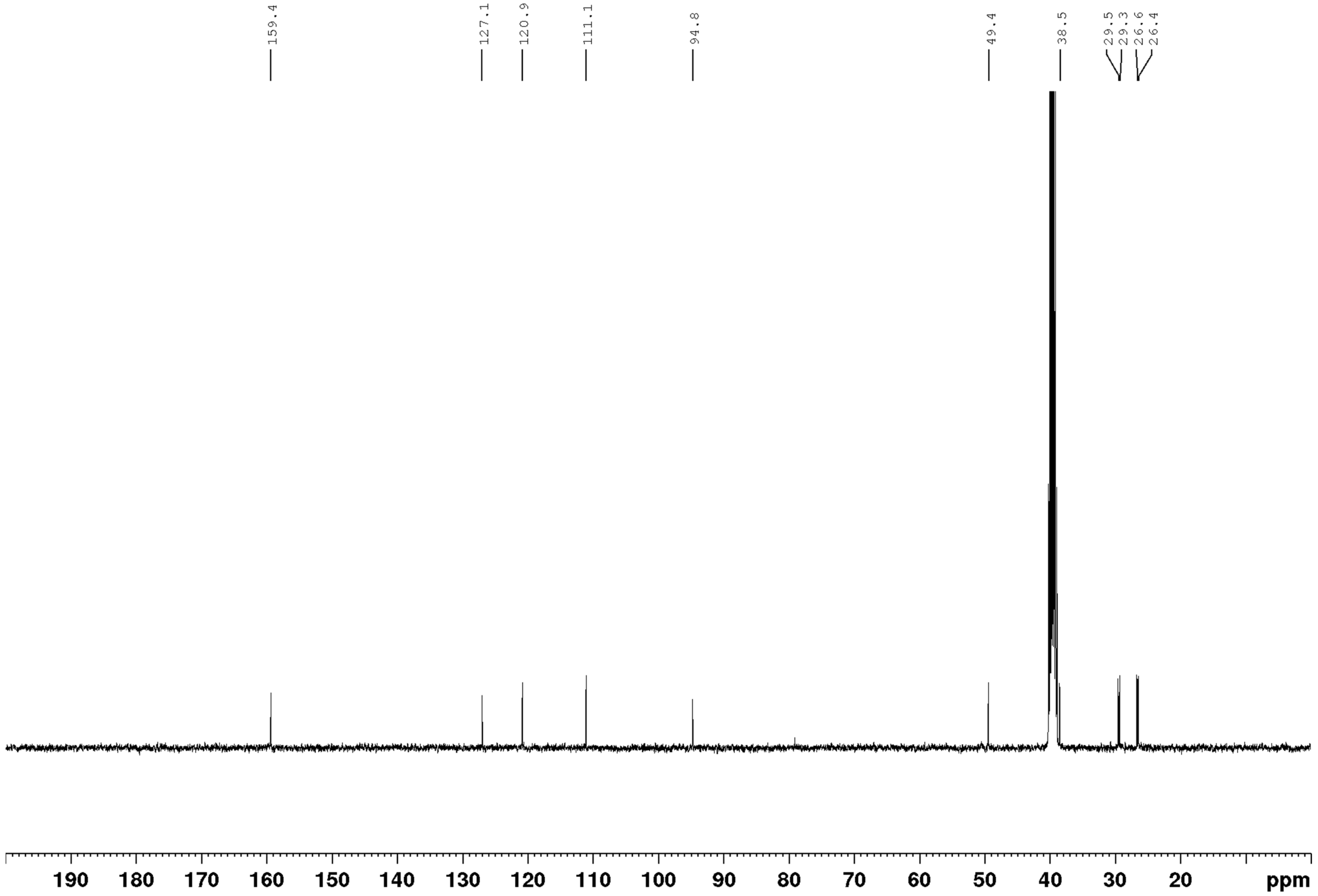

Figure x: ^{13}C NMR spectra of **15d** (100 MHz; DMSO-d_6).

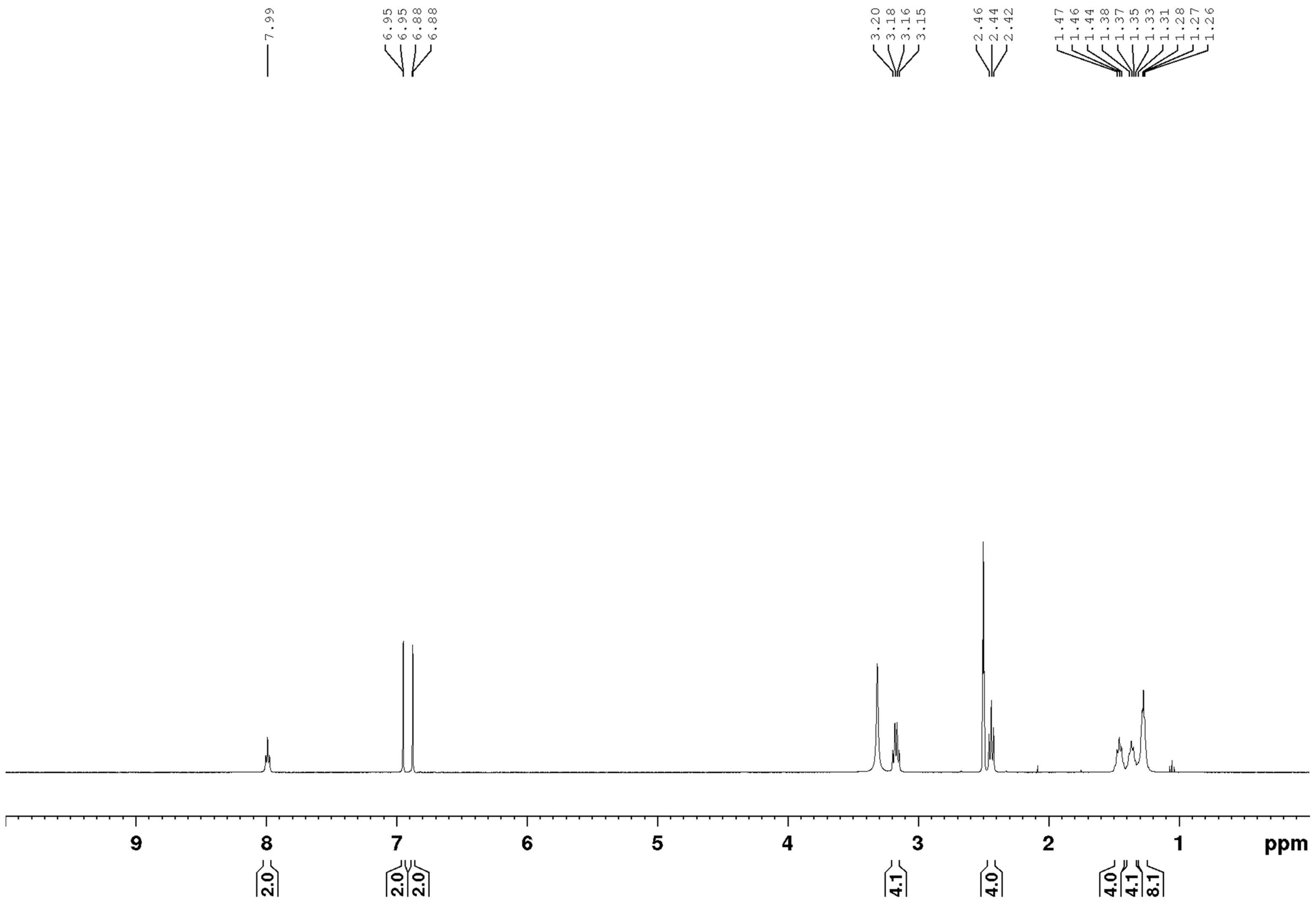

Figure x: ^{1}H NMR spectra of **15f** (400 MHz; DMSO-d_6).

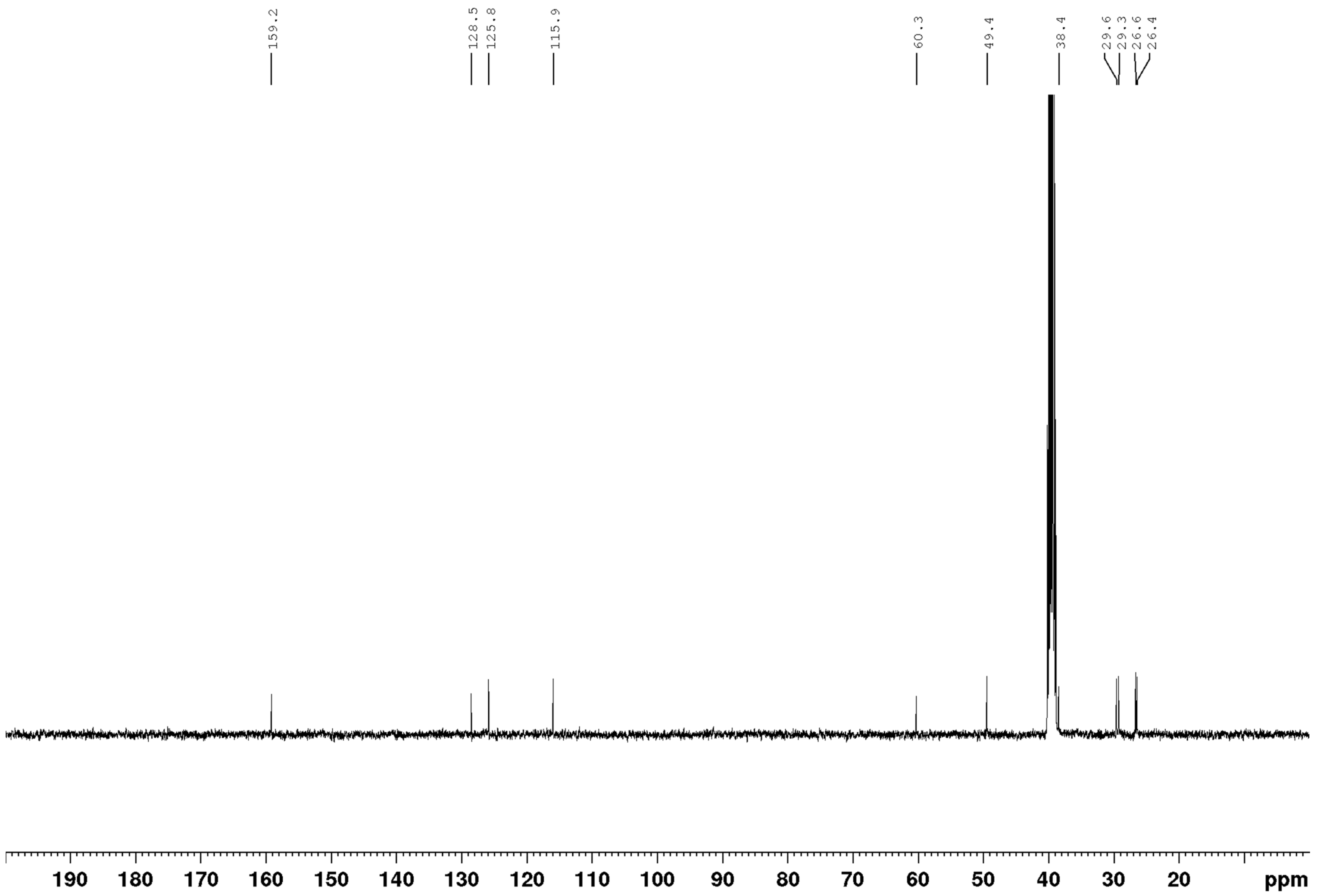

Figure x: ^{13}C NMR spectra of **15f** (100 MHz; DMSO-d_6).

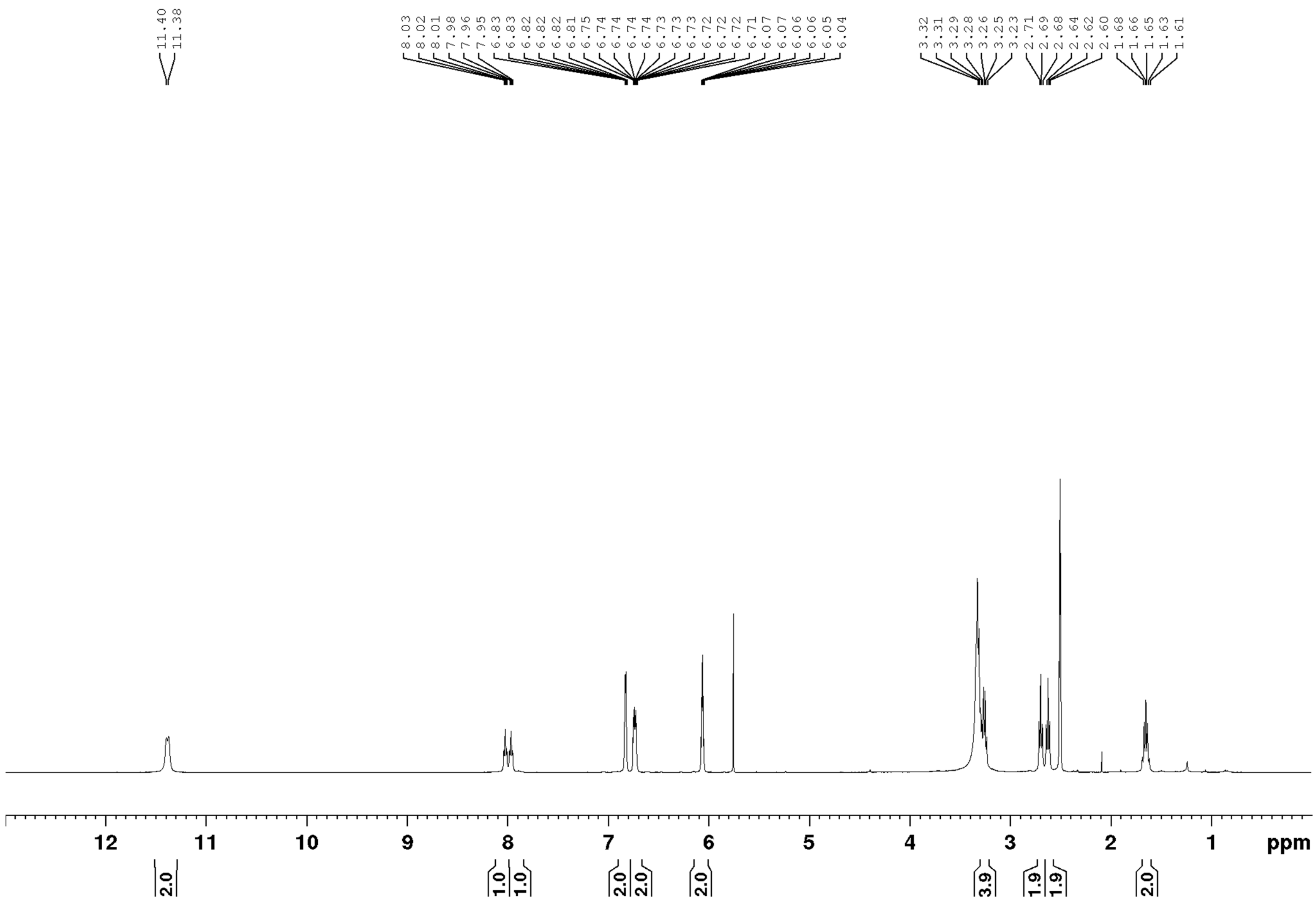

Figure x: ^{1}H NMR spectra of **16a** (400 MHz; DMSO-d_6).

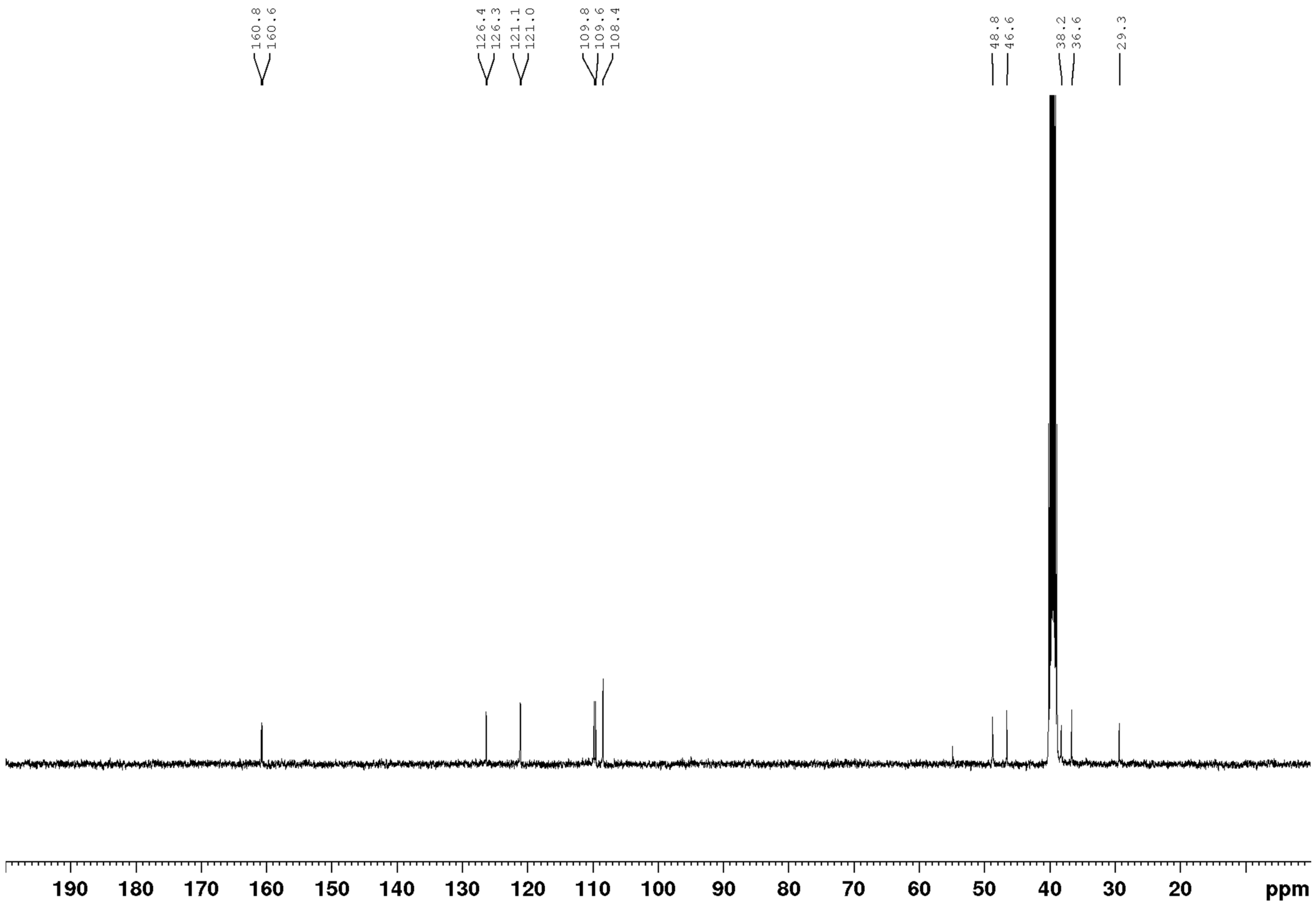

Figure x: ^{13}C NMR spectra of **16a** (100 MHz; DMSO-d_6).

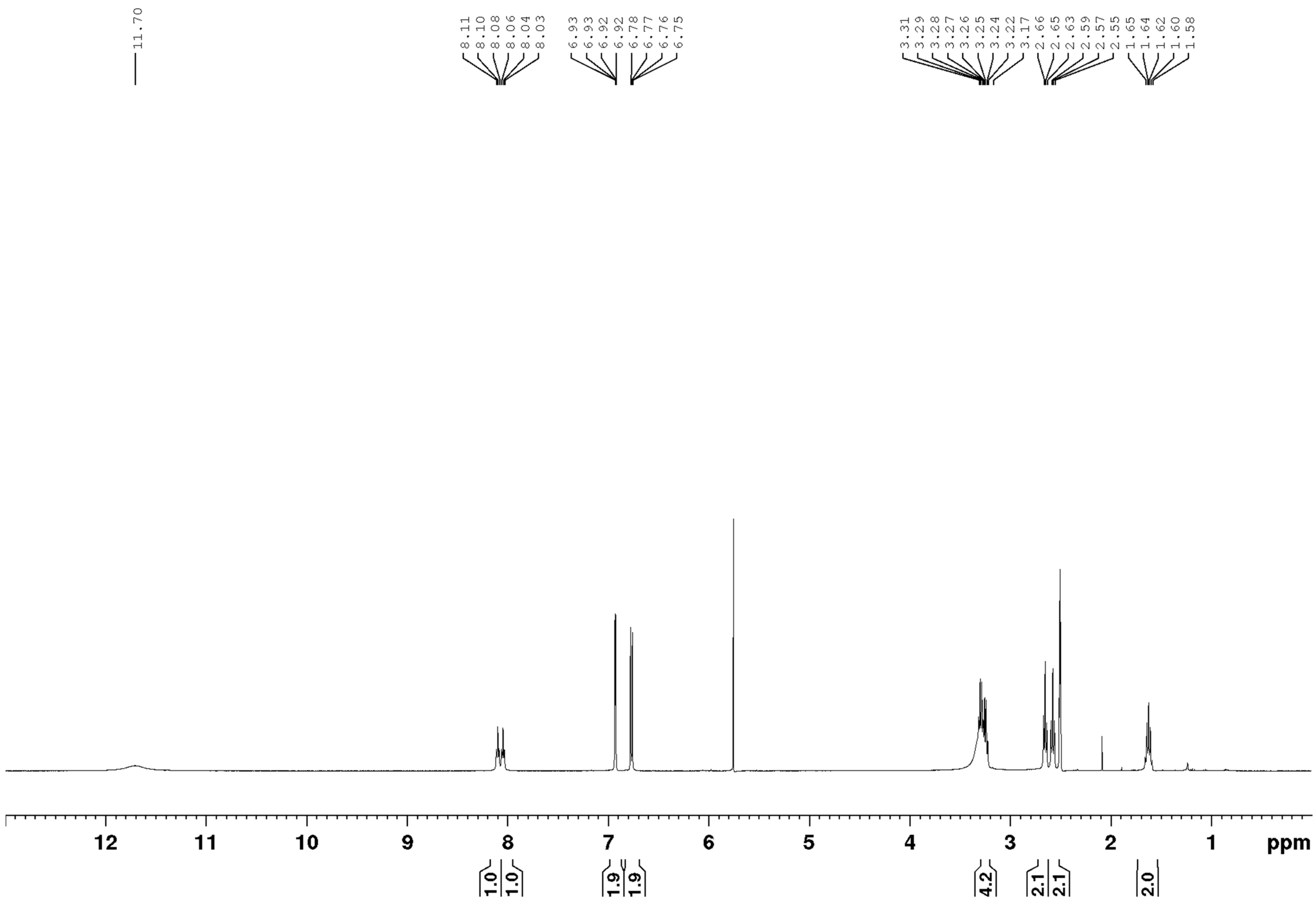

Figure x: ^{1}H NMR spectra of **16b** (400 MHz; DMSO-d_6).

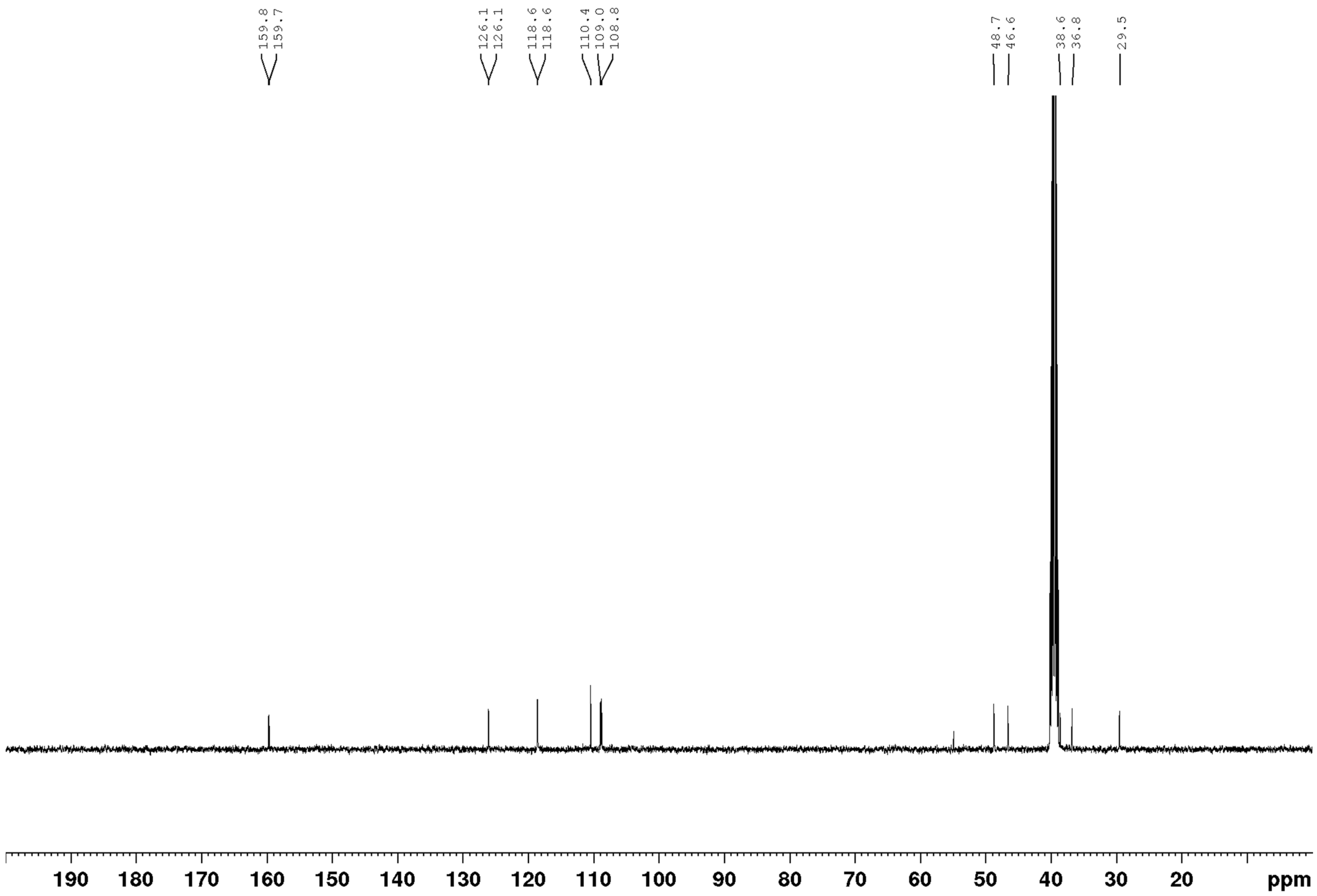

Figure x: ^{13}C NMR spectra of **16b** (100 MHz; DMSO-d_6).

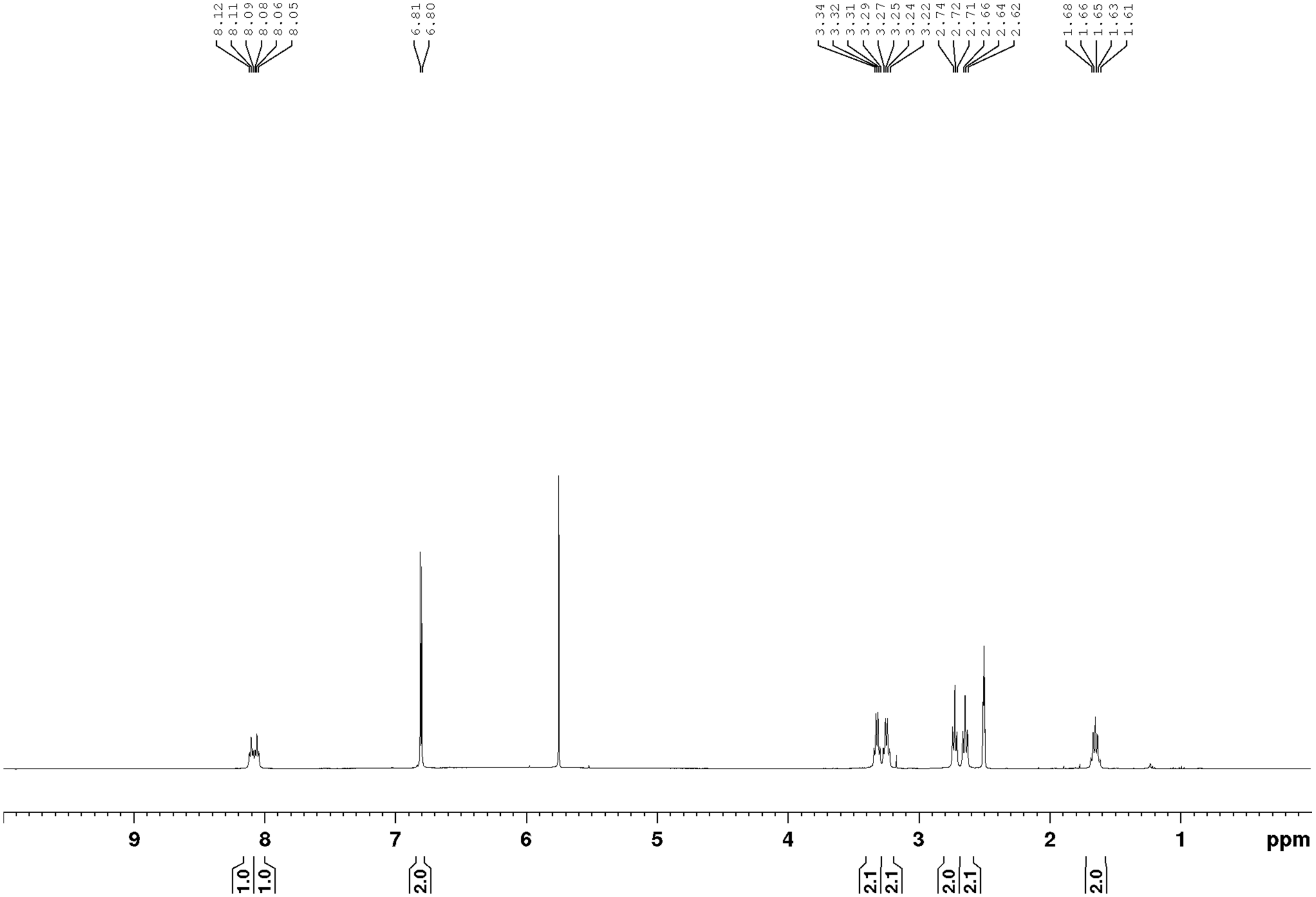

Figure x: ^{1}H NMR spectra of **16c** (400 MHz; DMSO-d_6).

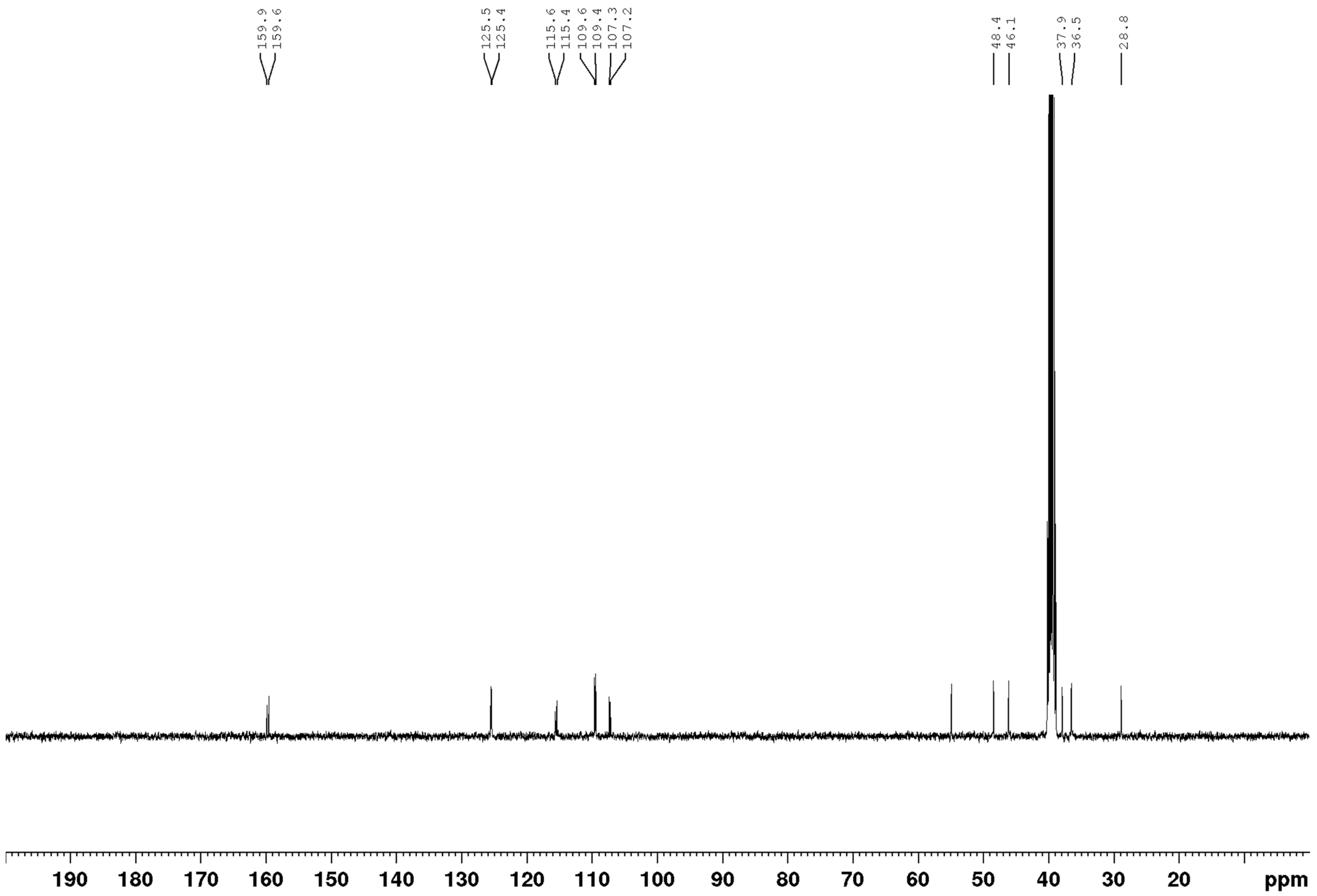

Figure x: ^{13}C NMR spectra of **16c** (100 MHz; DMSO-d_6).

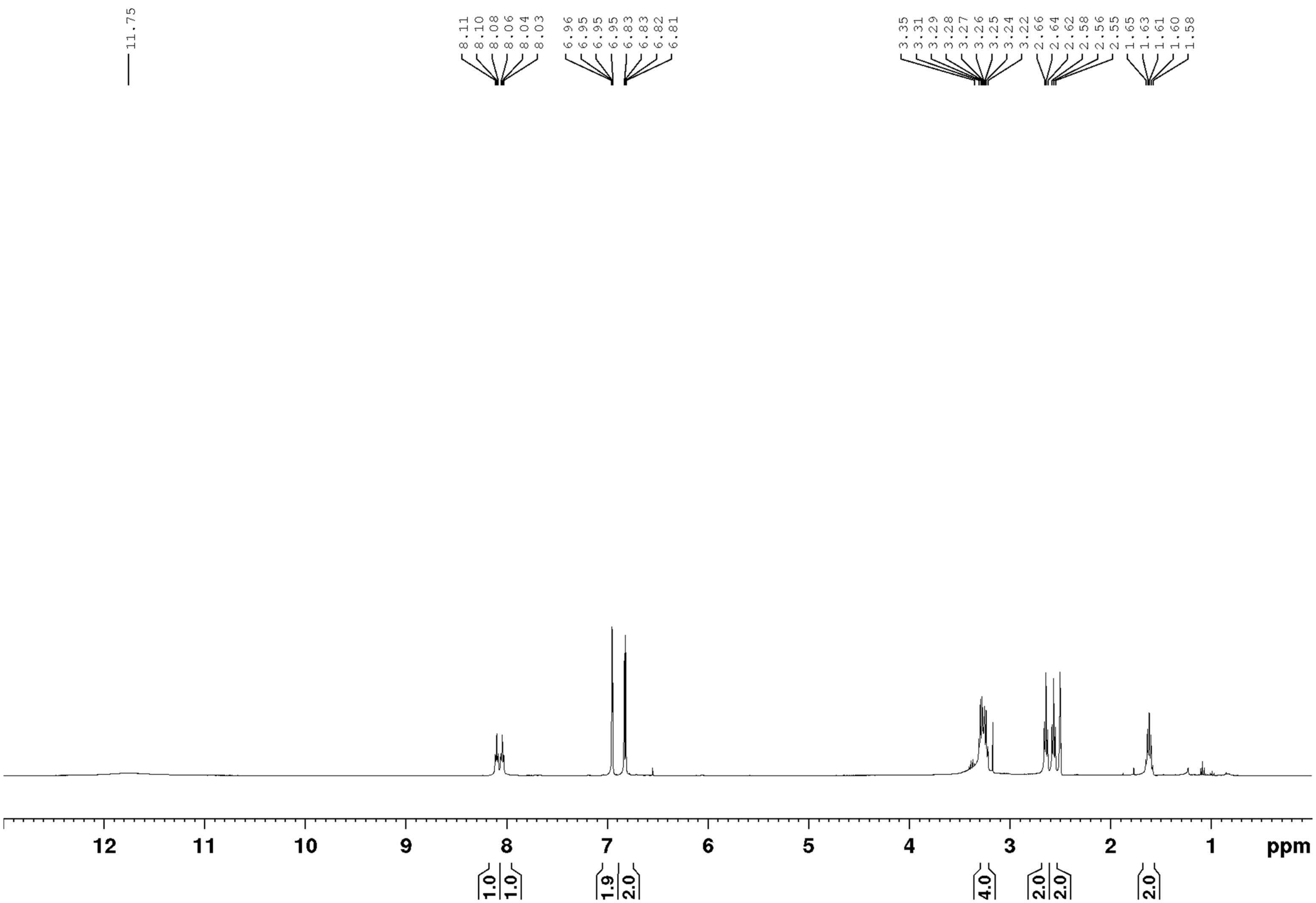

Figure x: ^{1}H NMR spectra of **16d** (400 MHz; DMSO-d_6).

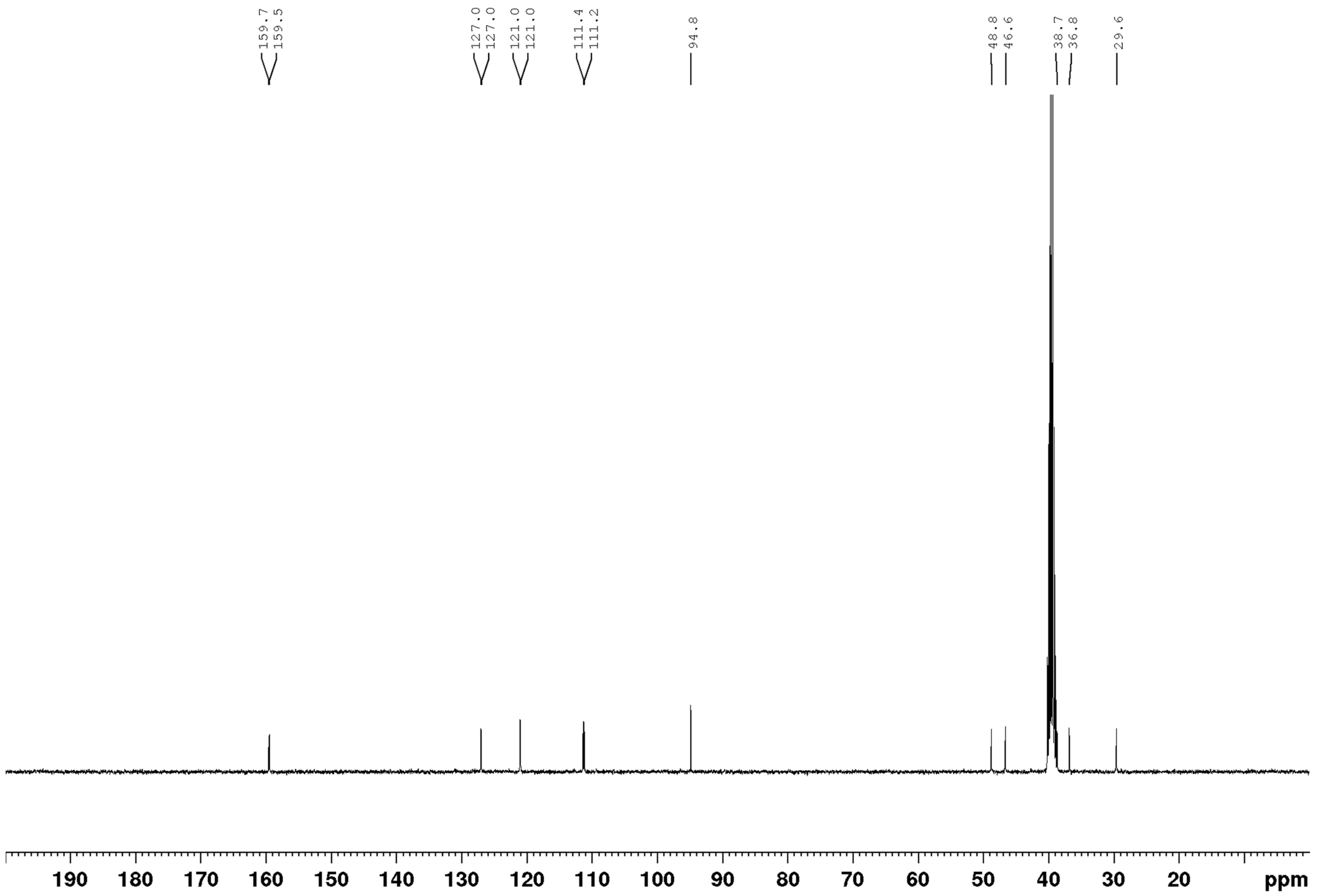

Figure x: ^{13}C NMR spectra of **16d** (100 MHz; DMSO-d_6).

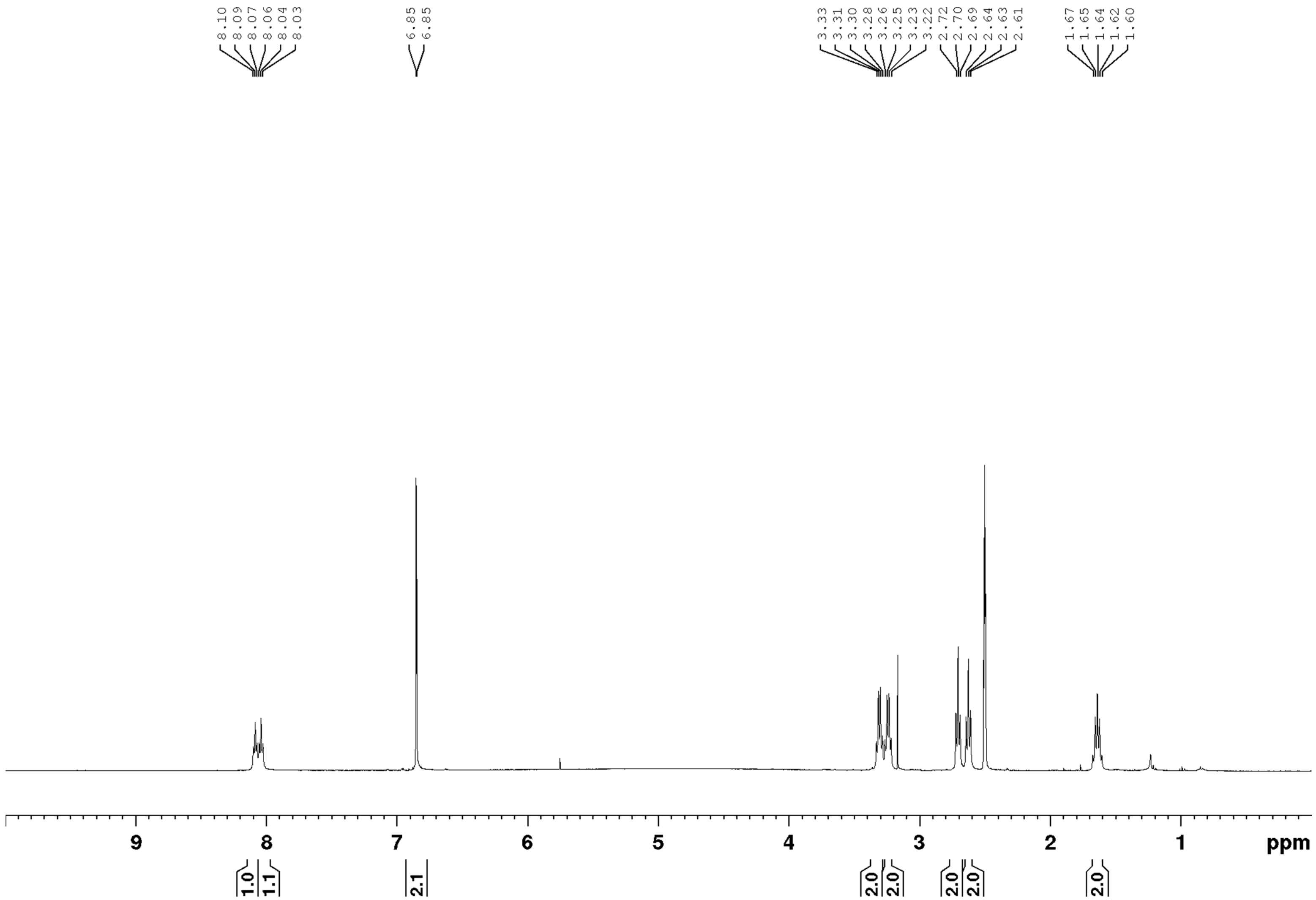

Figure x: ^{1}H NMR spectra of **16e** (400 MHz; DMSO-d_6).

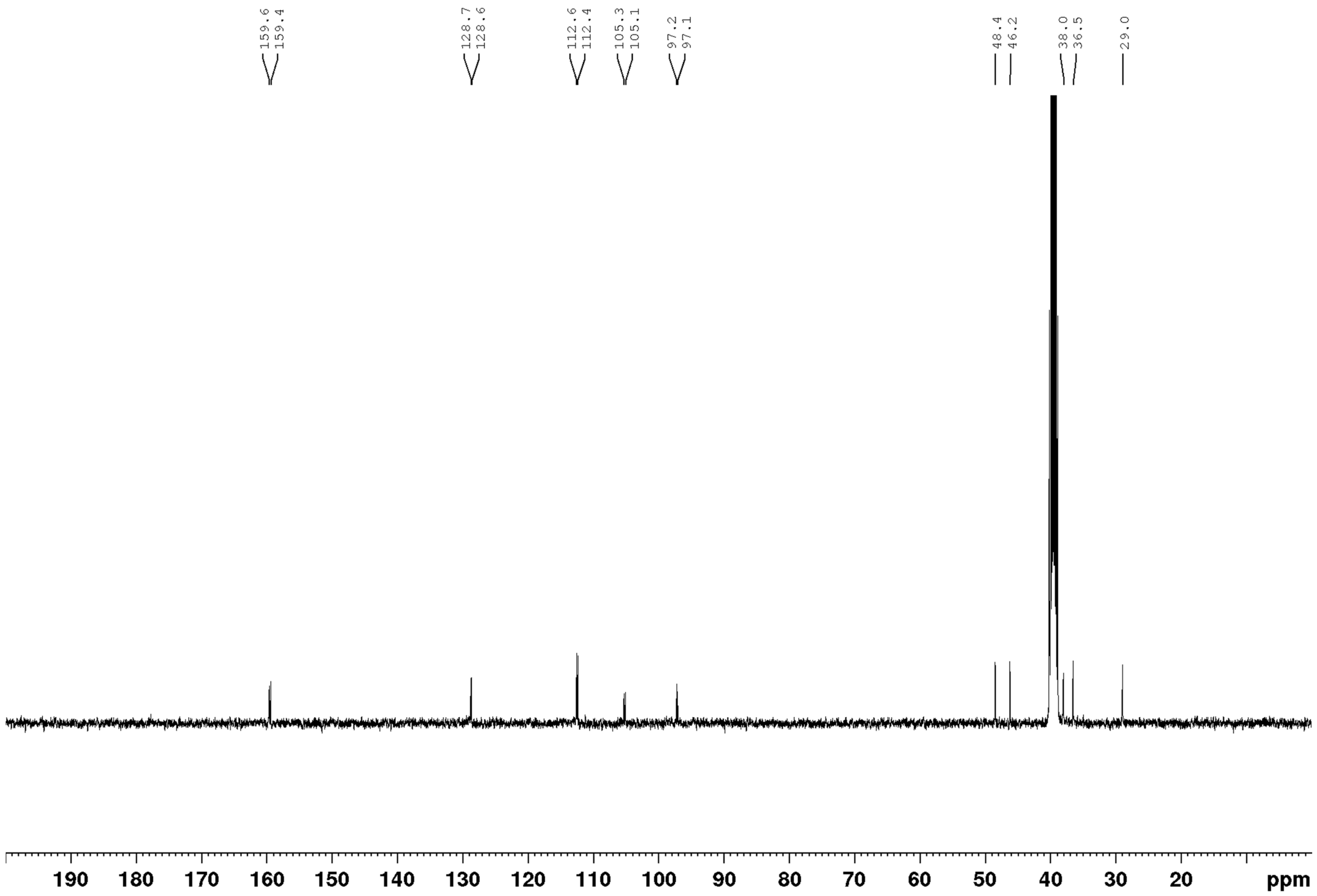

Figure x: ^{13}C NMR spectra of **16e** (100 MHz; DMSO-d_6).

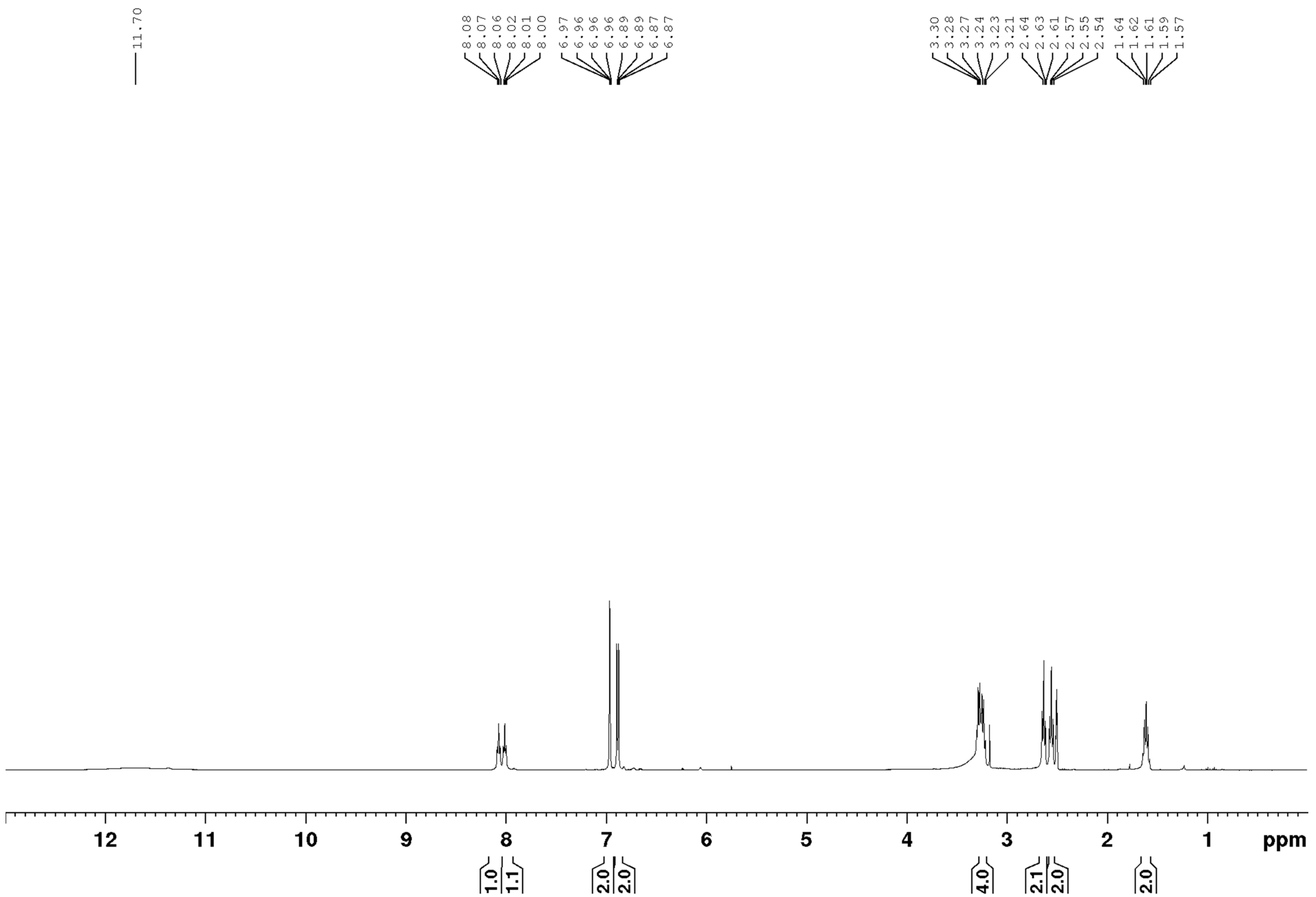

Figure x: ^{1}H NMR spectra of **16f** (400 MHz; DMSO-d_6).

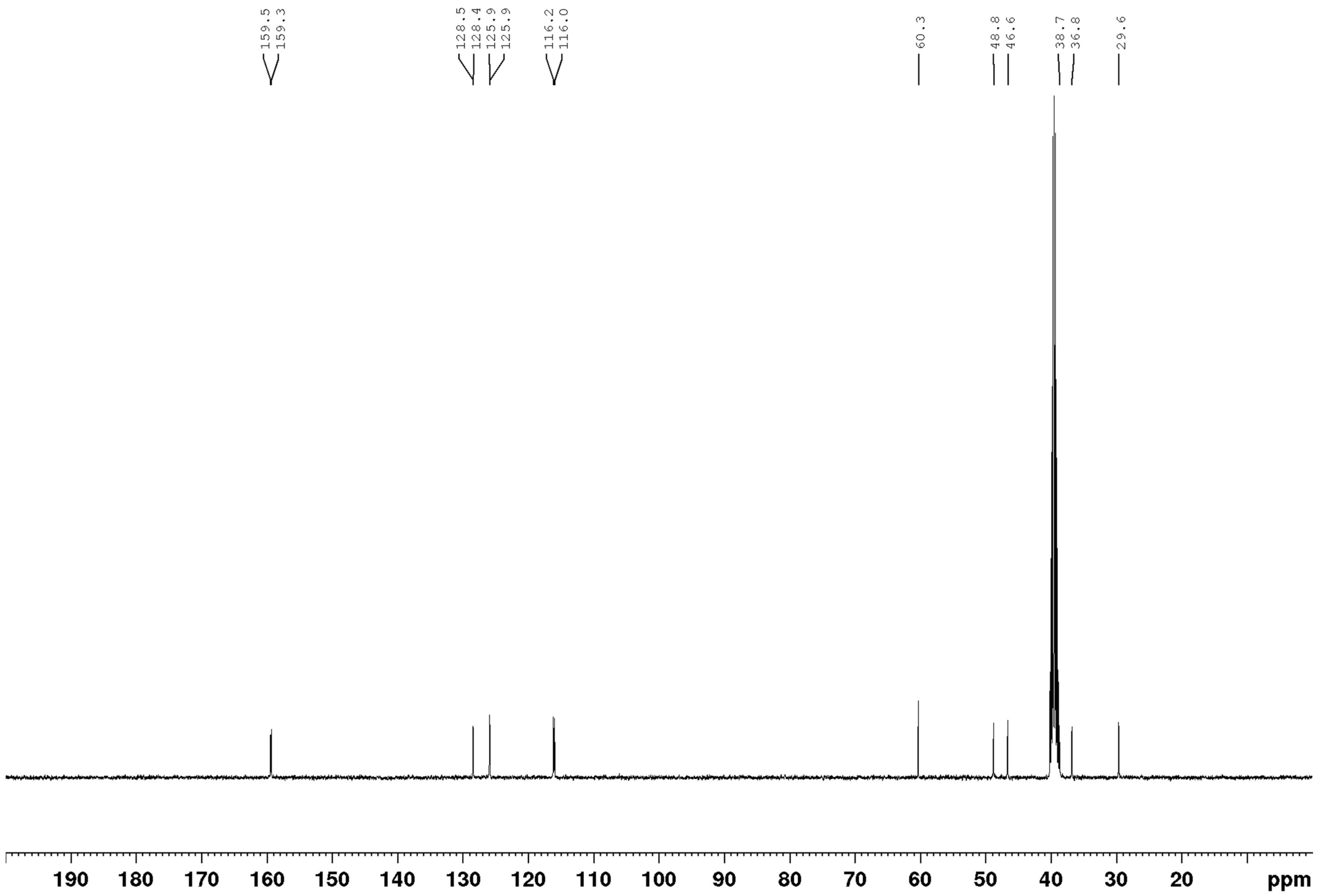

Figure x: ^{13}C NMR spectra of **16f** (100 MHz; DMSO-d_6).

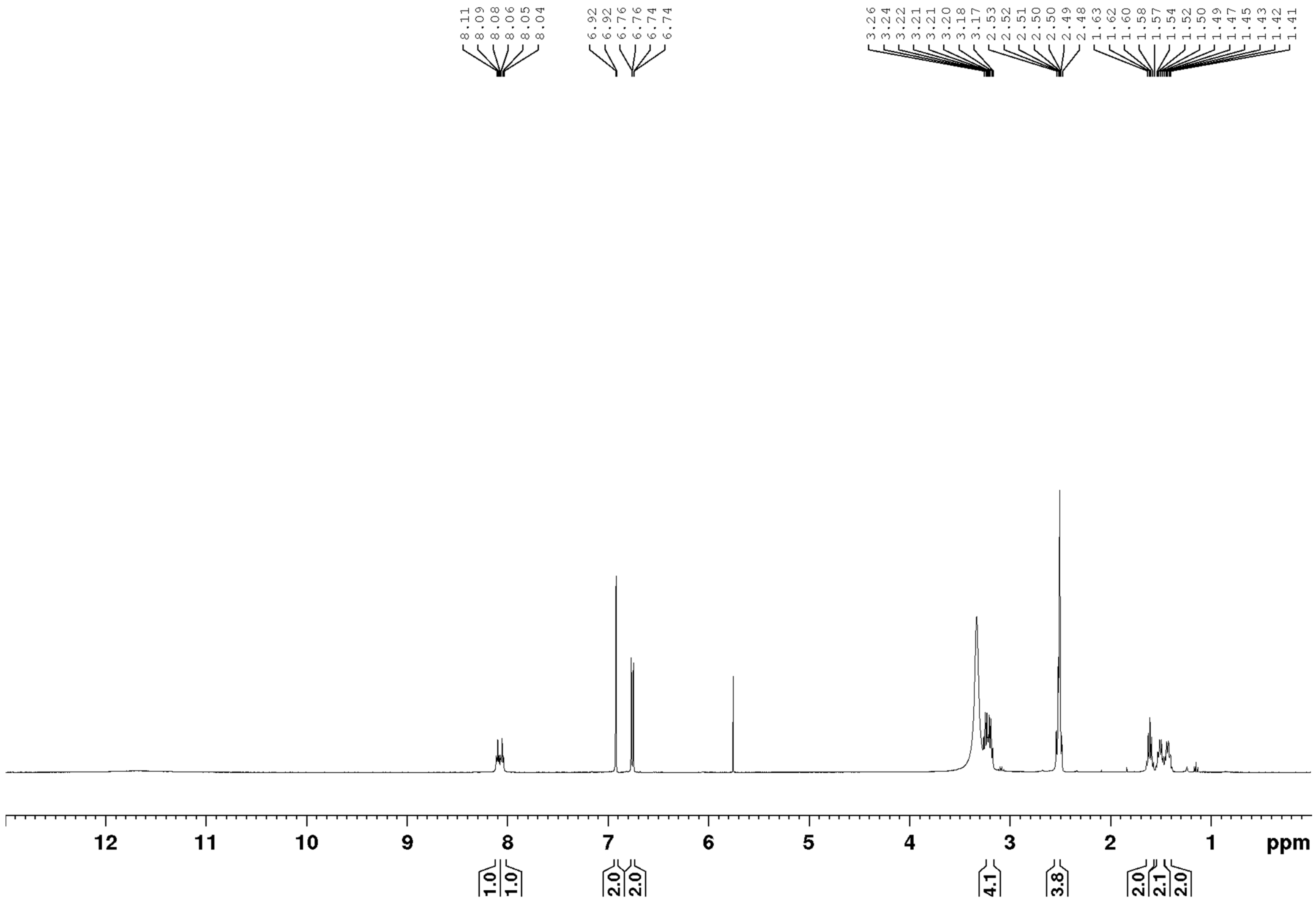

Figure x: ^{1}H NMR spectra of **17b** (400 MHz; DMSO-d_6).

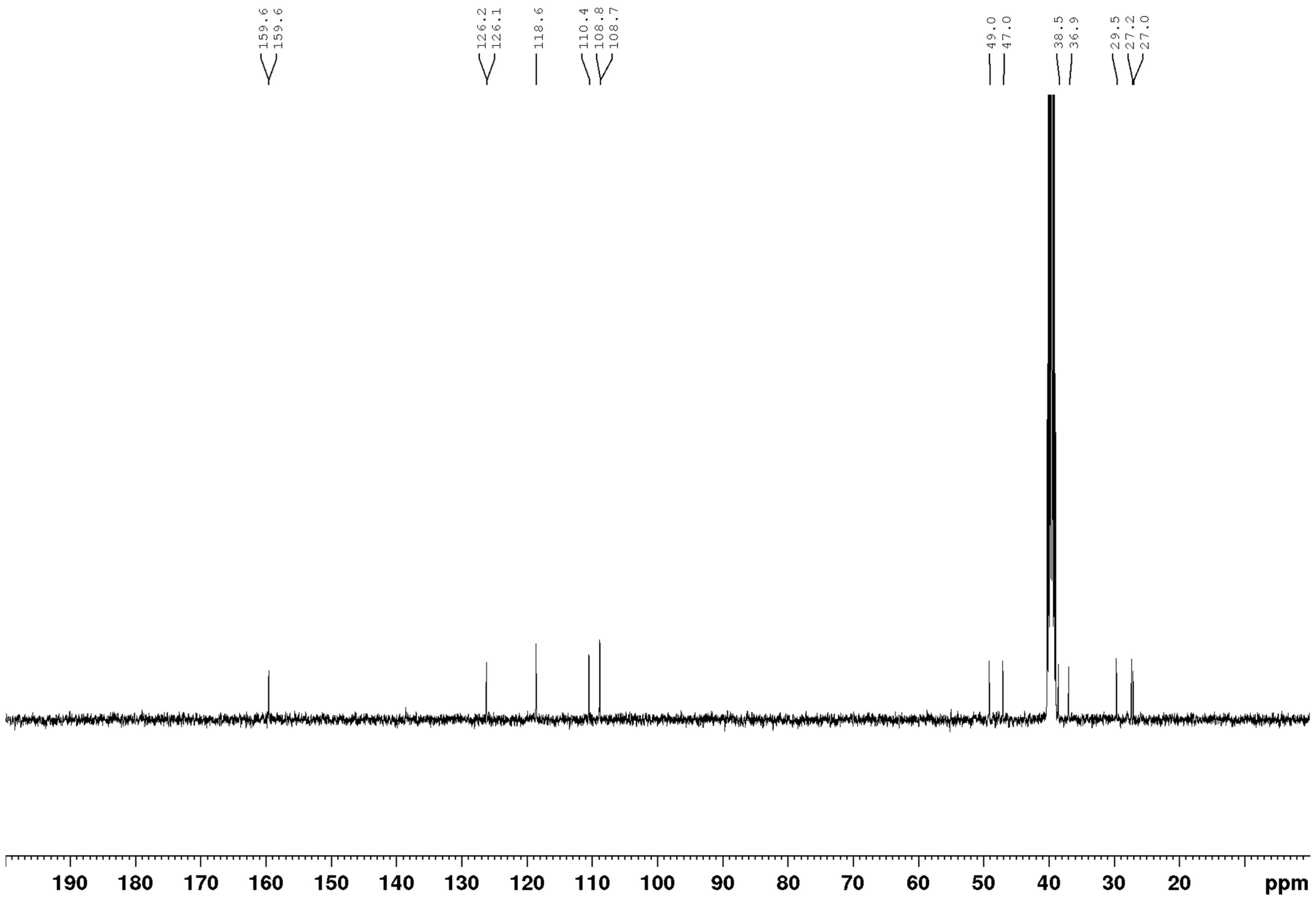

Figure x: ^{13}C NMR spectra of **17b** (100 MHz; DMSO-d_6).

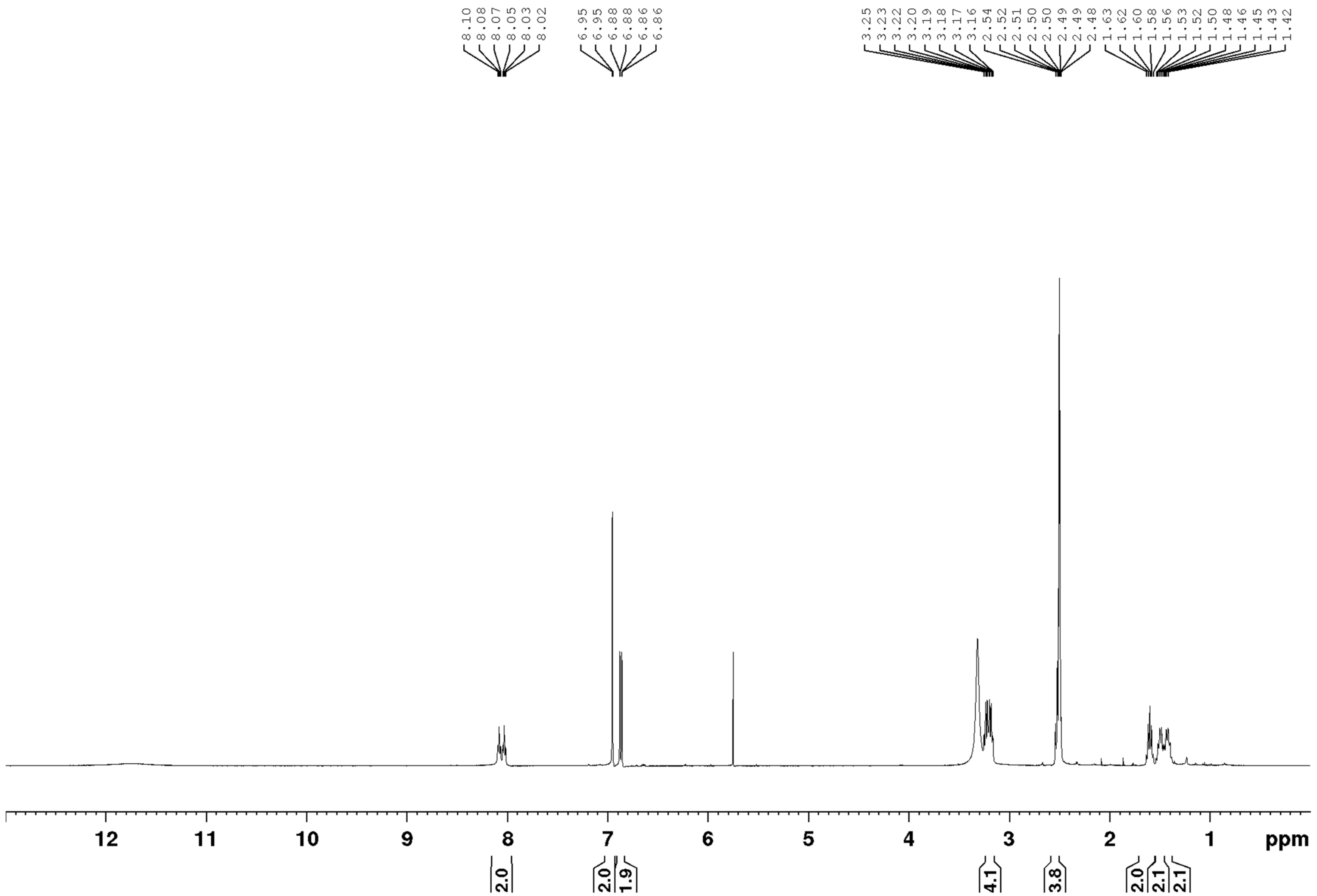

Figure x: ^{1}H NMR spectra of **17f** (400 MHz; DMSO-d_6).

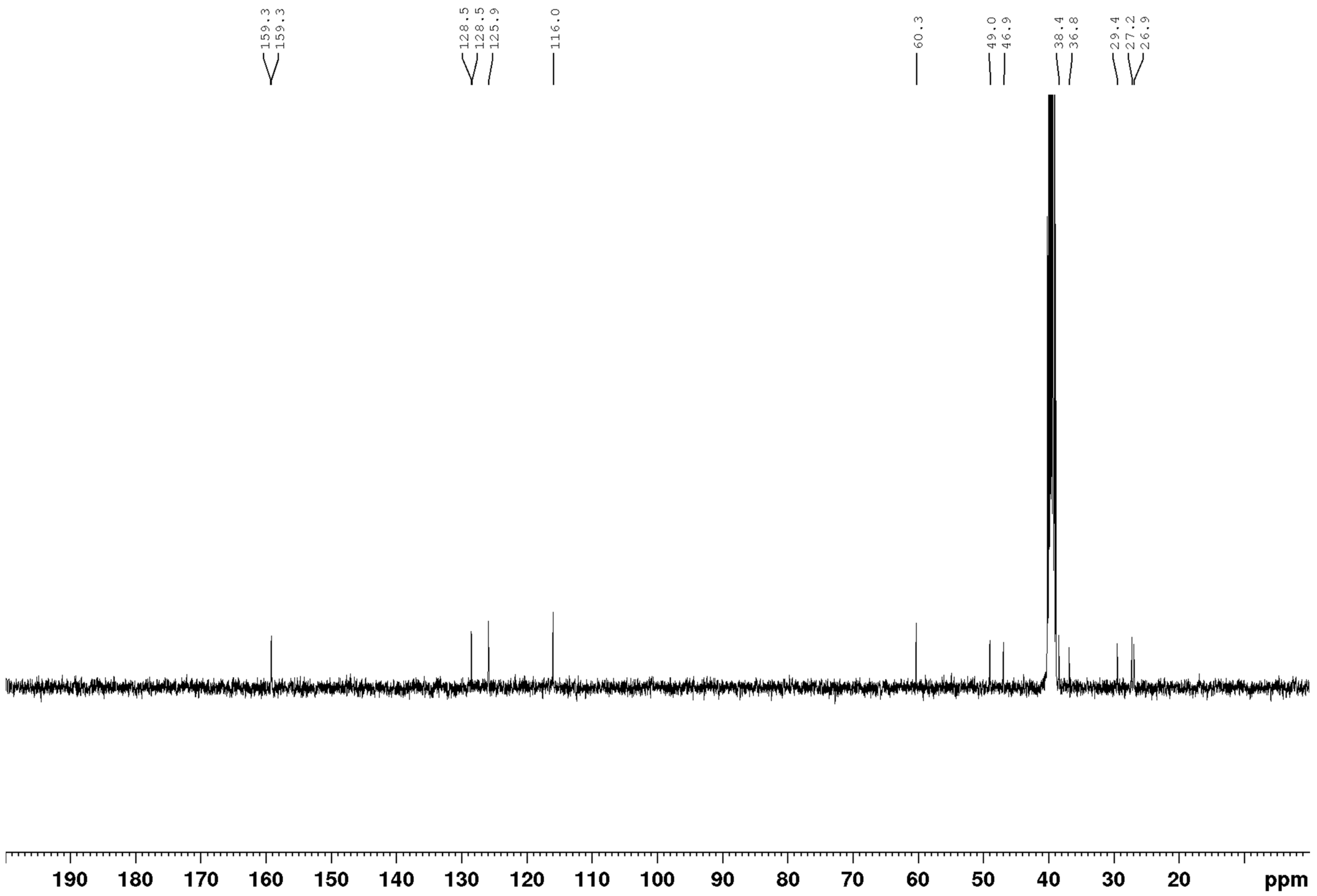

Figure x: ^{13}C NMR spectra of **17f** (100 MHz; DMSO-d_6).

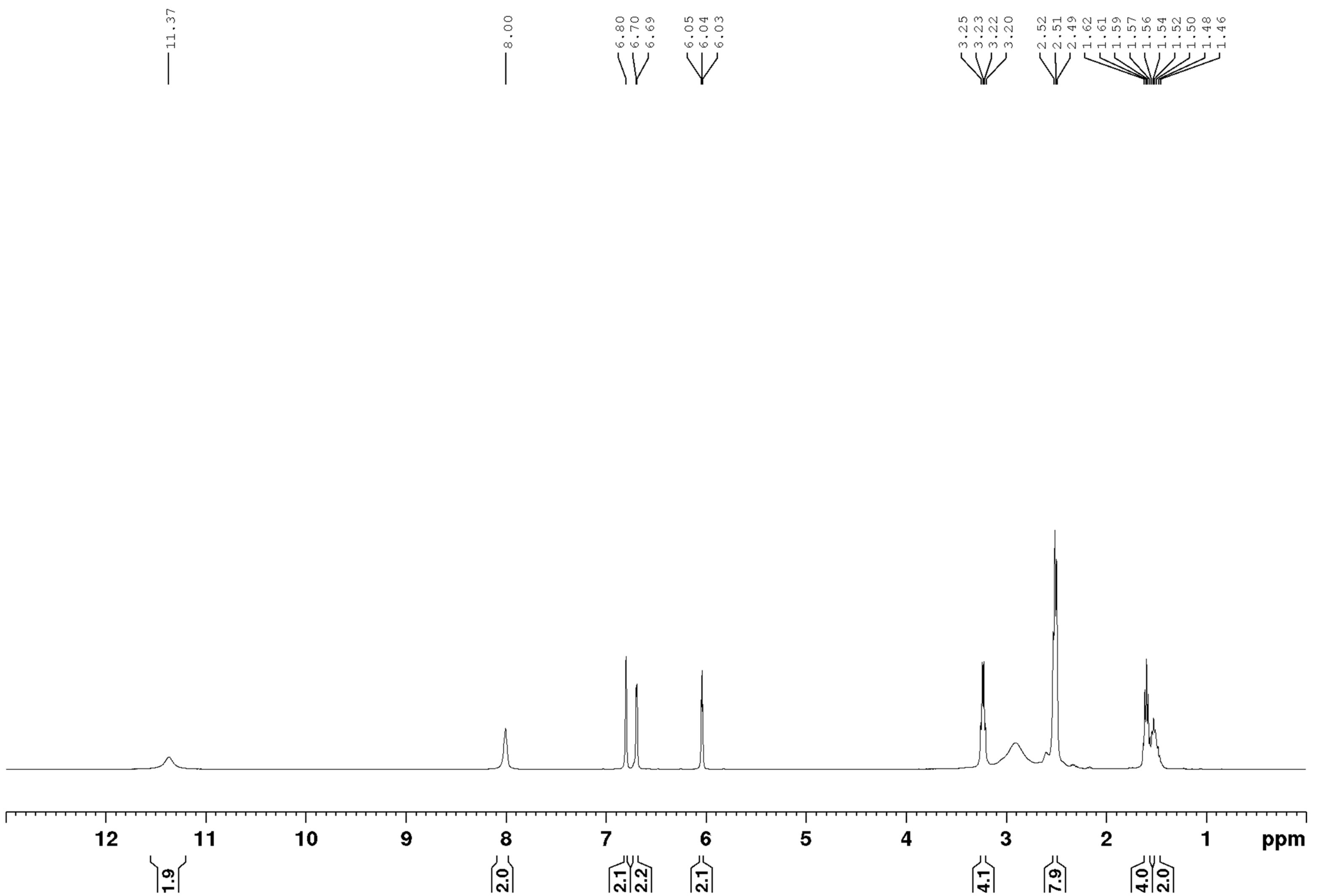

Figure x: ^{1}H NMR spectra of **18a** (400 MHz; DMSO-d_6).

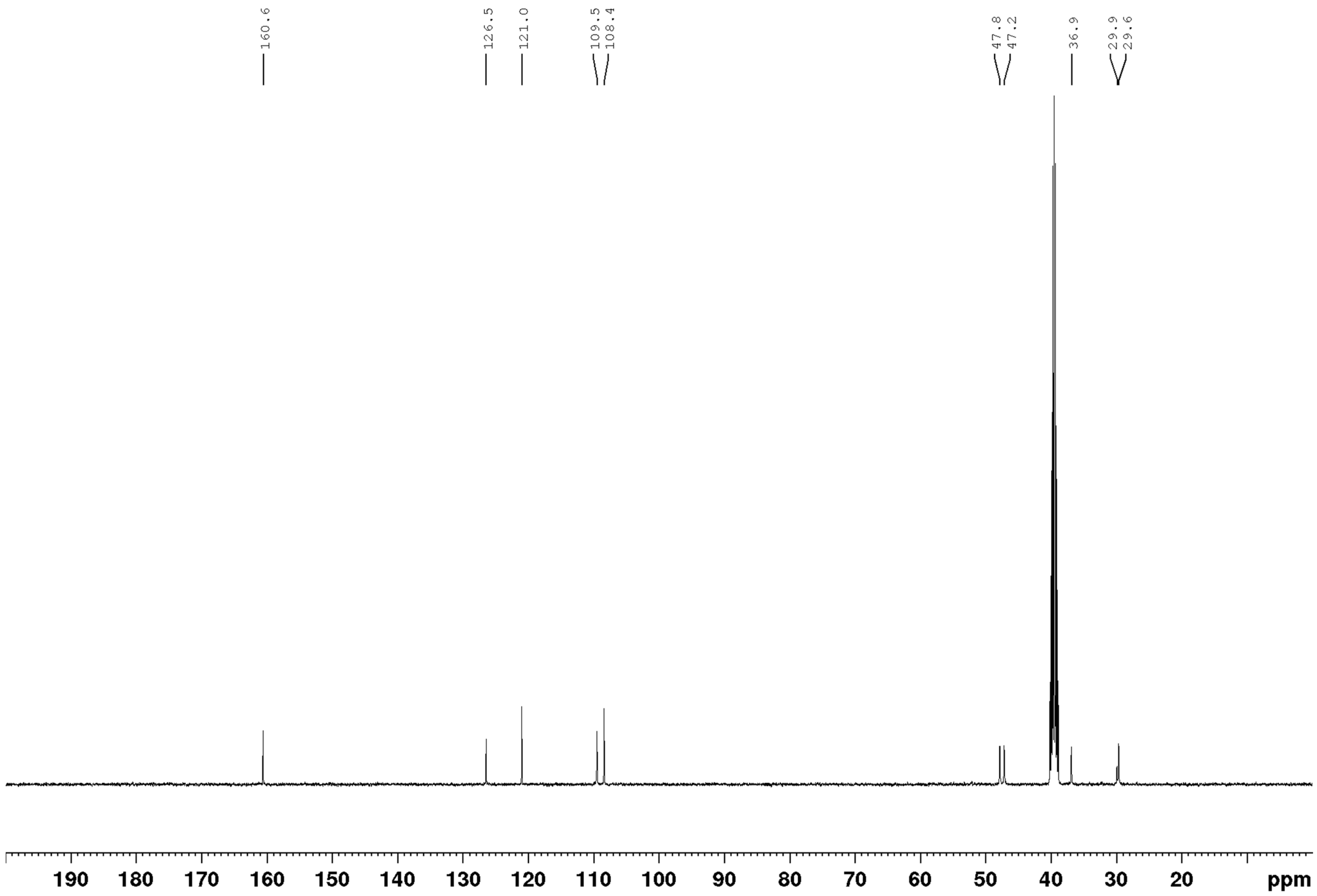

Figure x: ^{13}C NMR spectra of **18a** (100 MHz; DMSO-d_6).

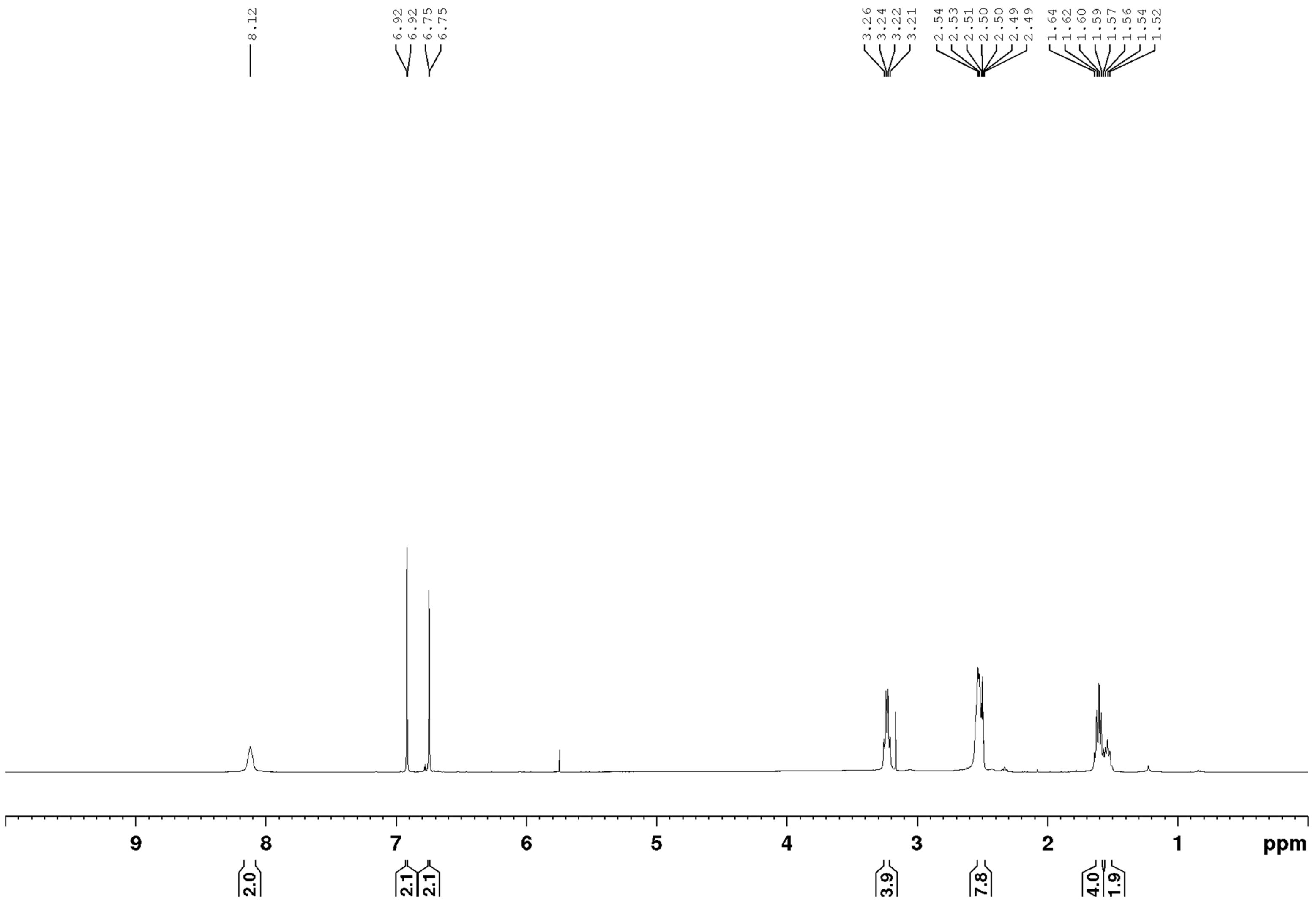

Figure x: ^{1}H NMR spectra of **18b** (400 MHz; DMSO-d_6).

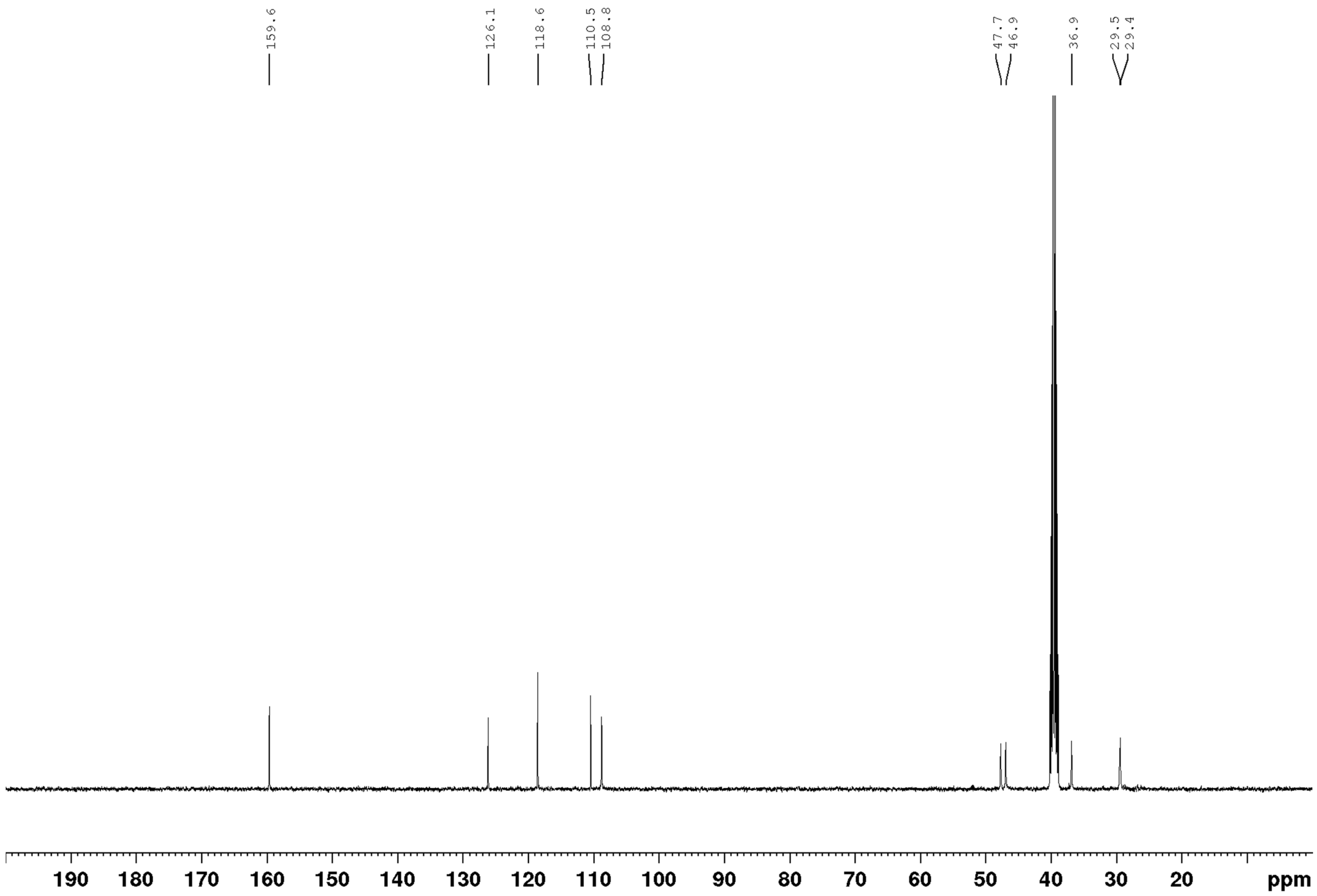

Figure x: ^{13}C NMR spectra of **18b** (100 MHz; DMSO-d_6).

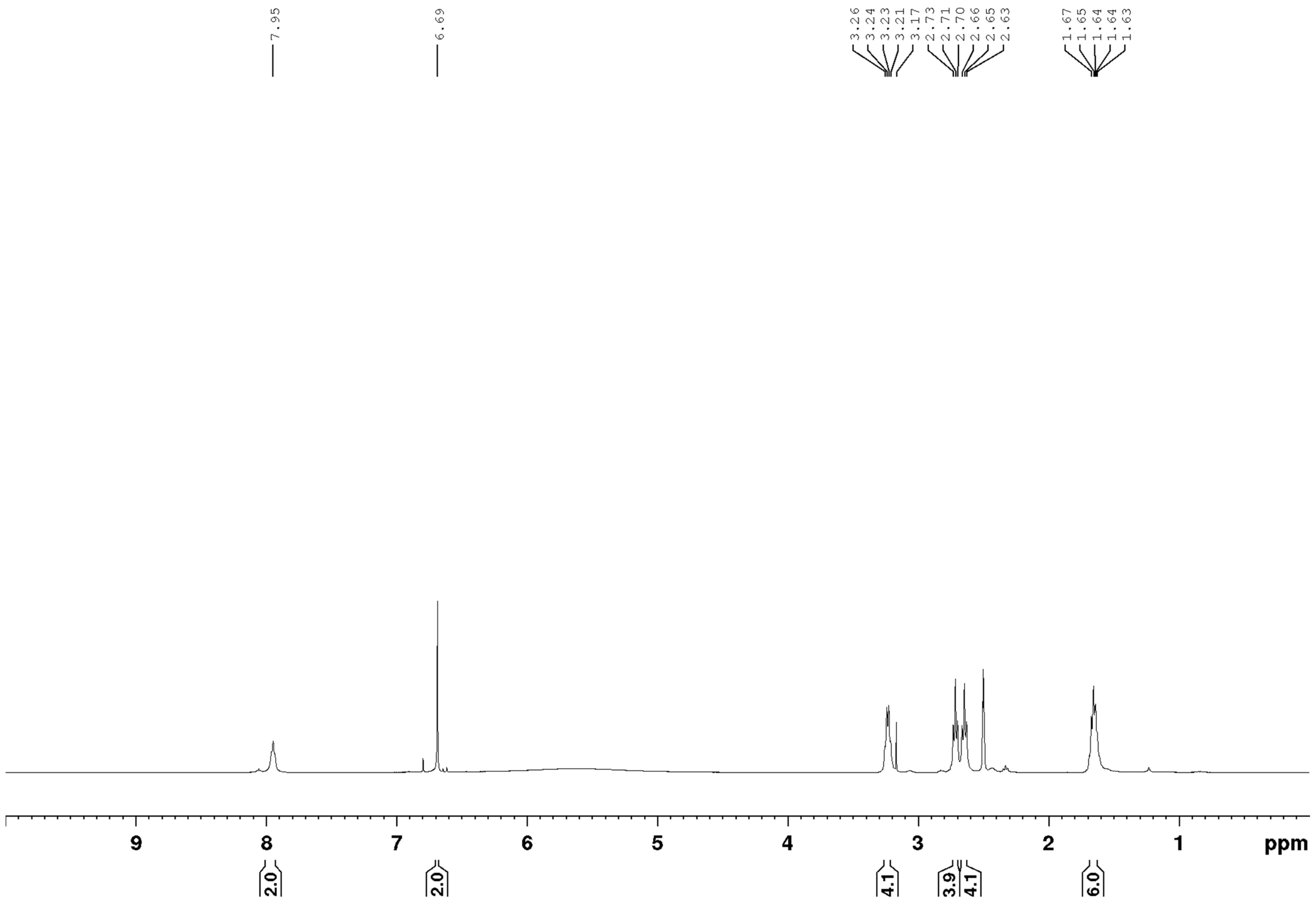

Figure x: ^{1}H NMR spectra of **18c** (400 MHz; DMSO-d_6).

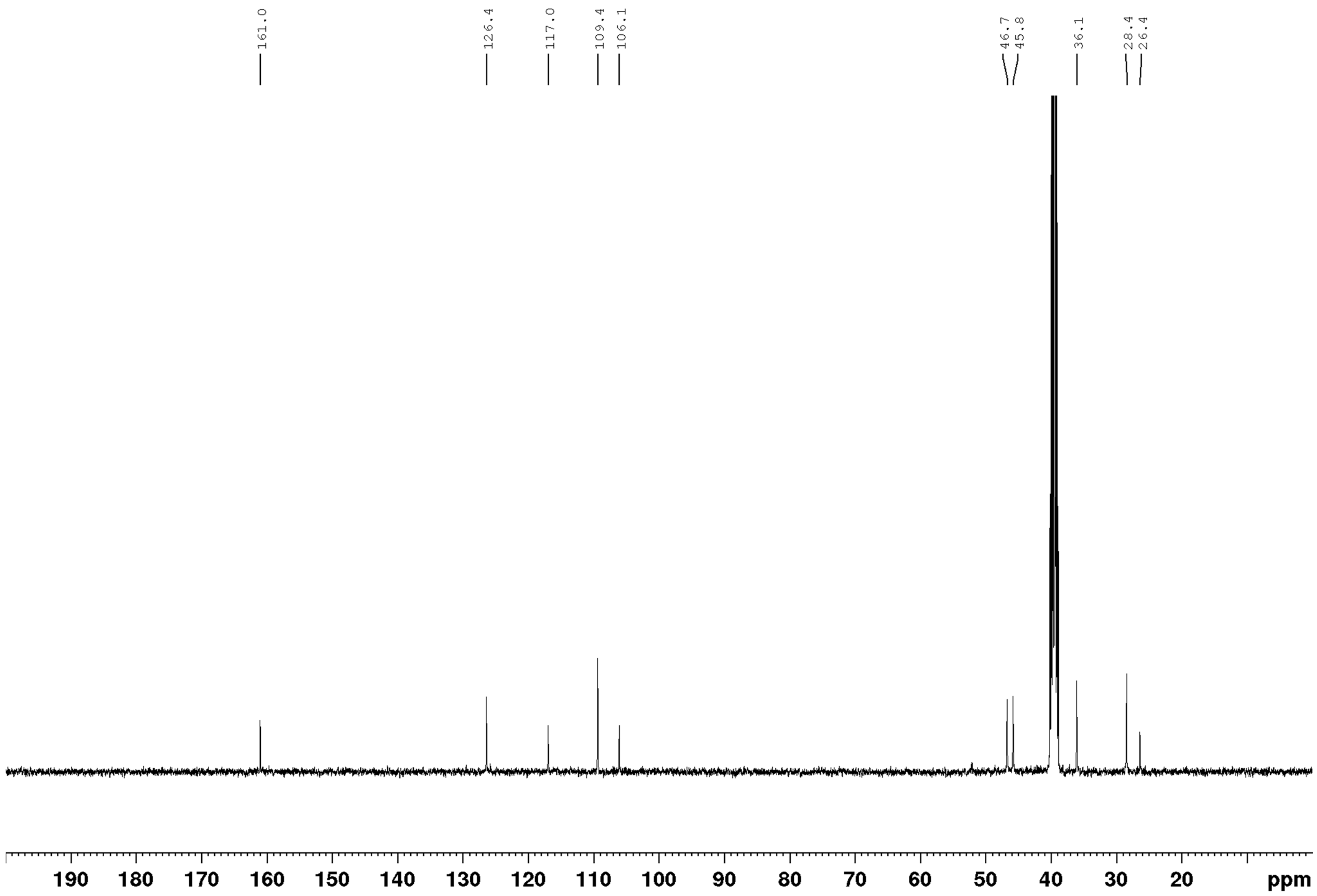

Figure x: ^{13}C NMR spectra of **18c** (100 MHz; DMSO-d_6).

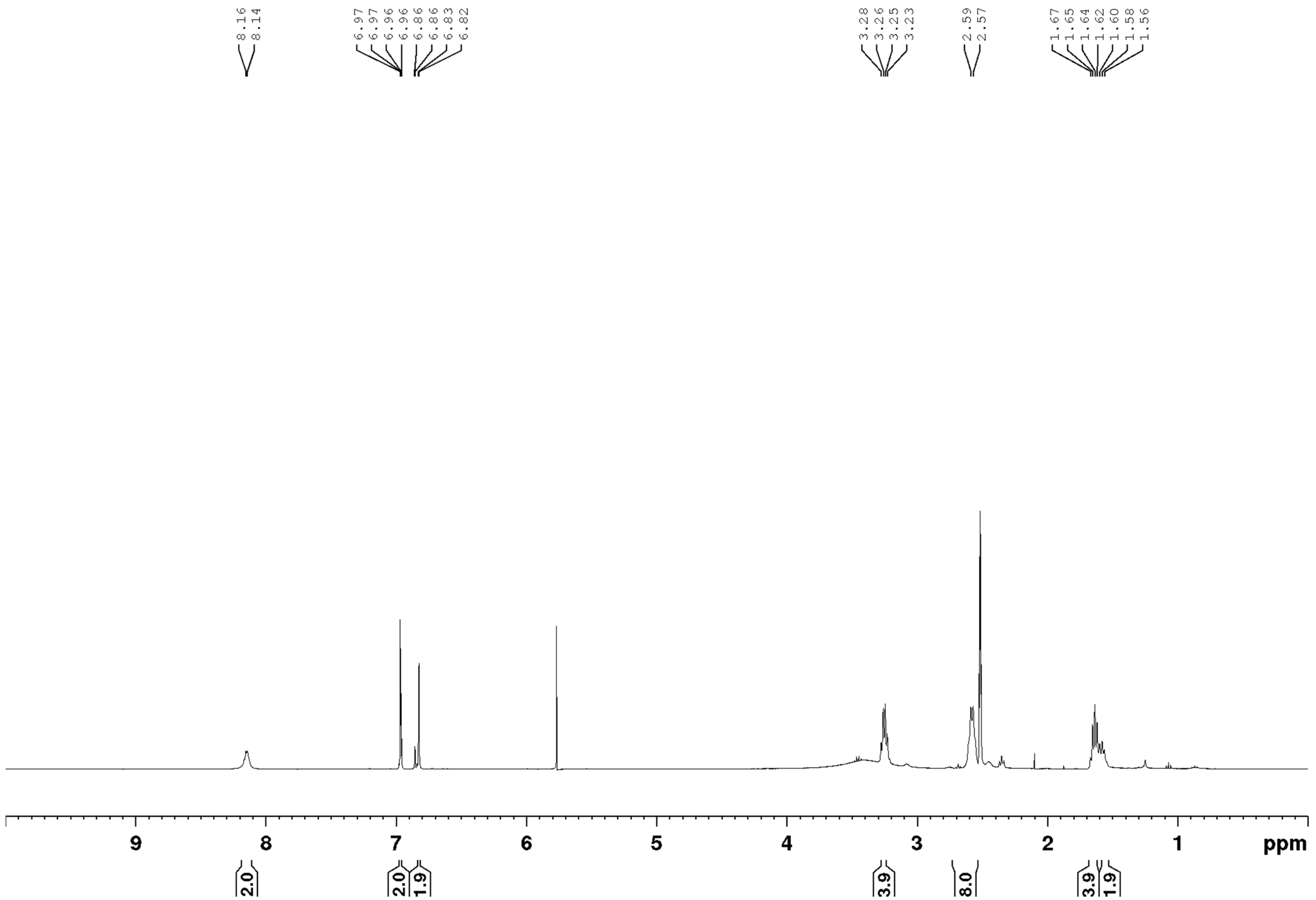

Figure x: ^{1}H NMR spectra of **18d** (400 MHz; DMSO-d_6).

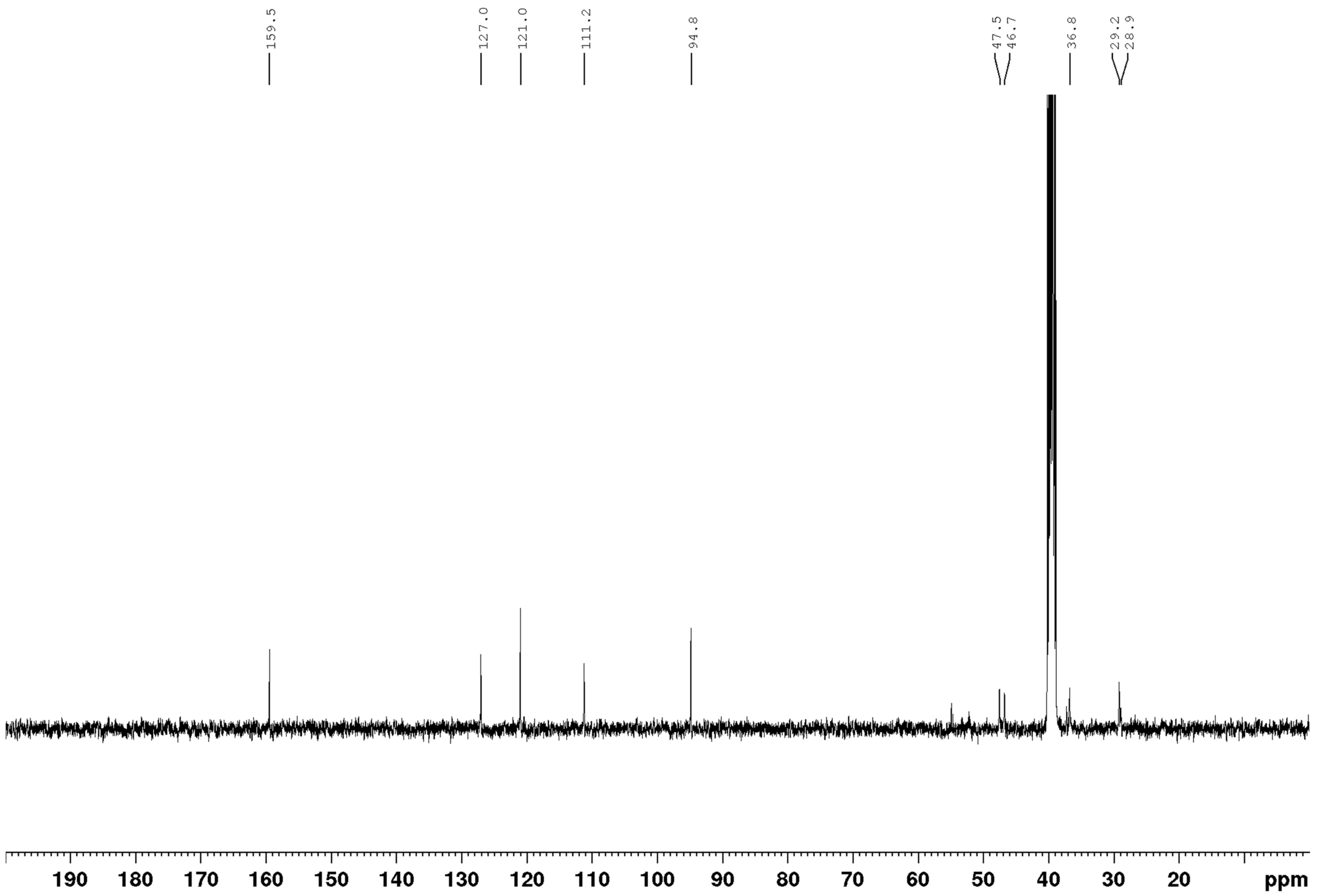

Figure x: ^{13}C NMR spectra of **18d** (100 MHz; DMSO-d_6).

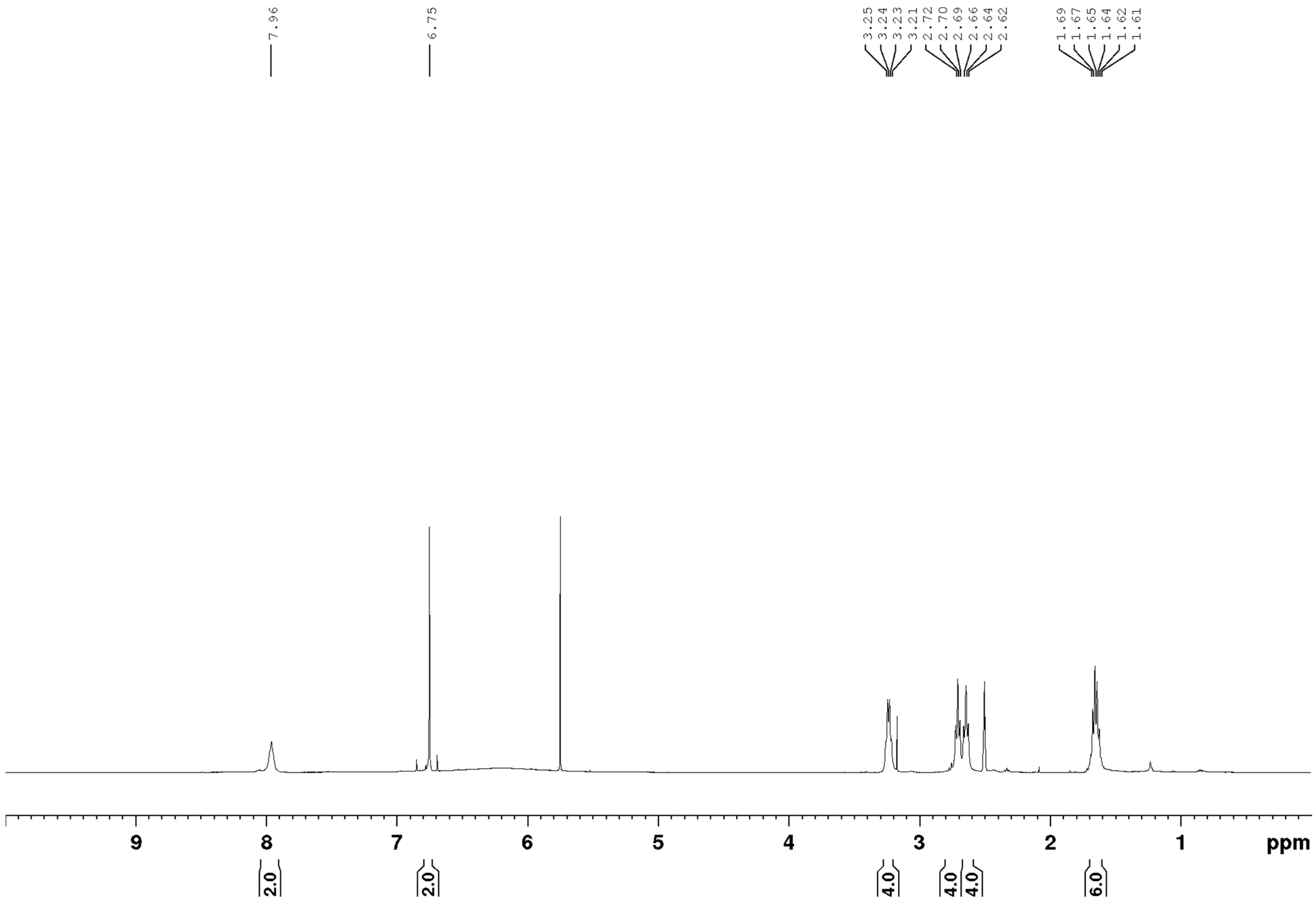

Figure x: ^{1}H NMR spectra of **18e** (400 MHz; DMSO-d_6).

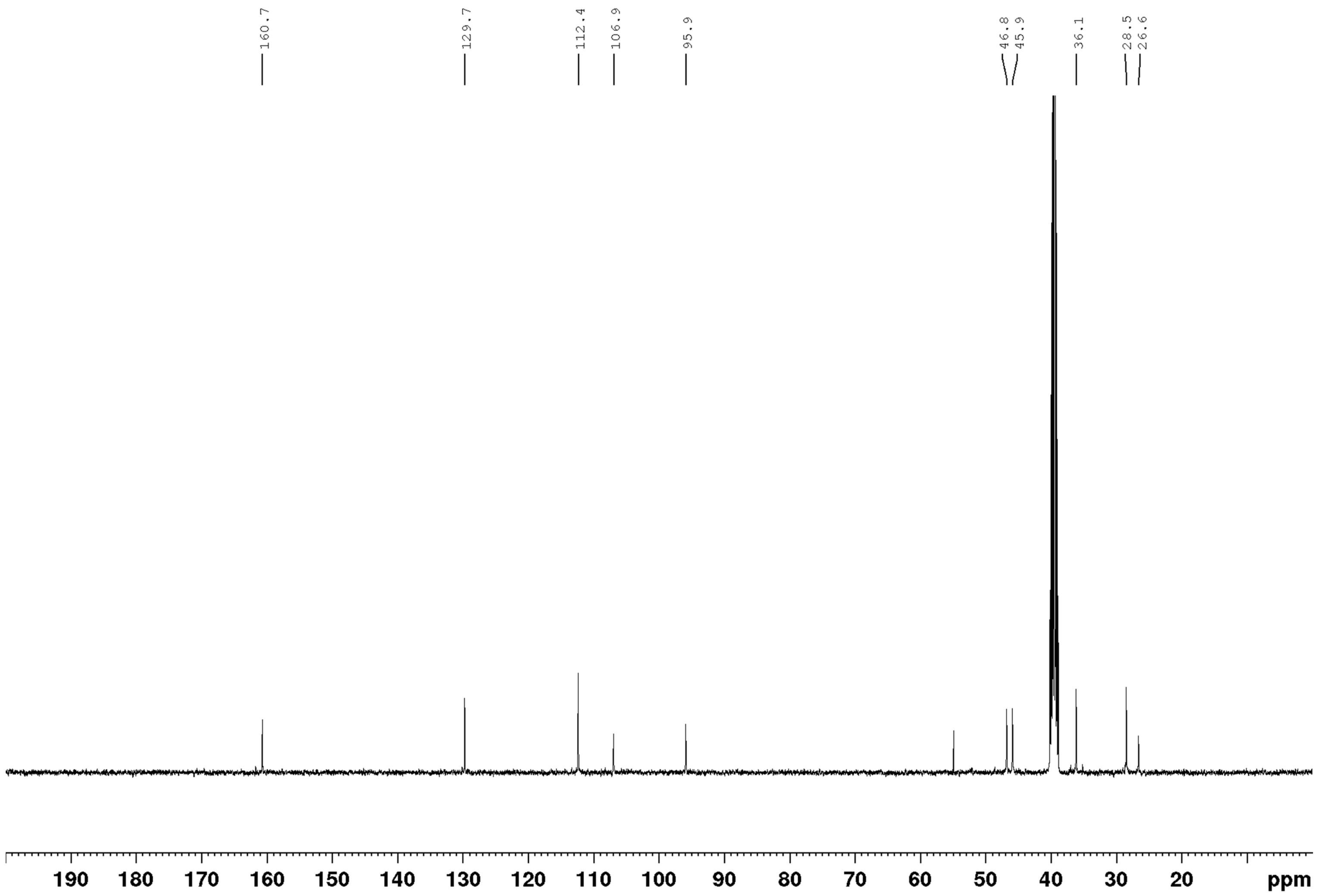

Figure x: ^{13}C NMR spectra of **18e** (100 MHz; DMSO-d_6).

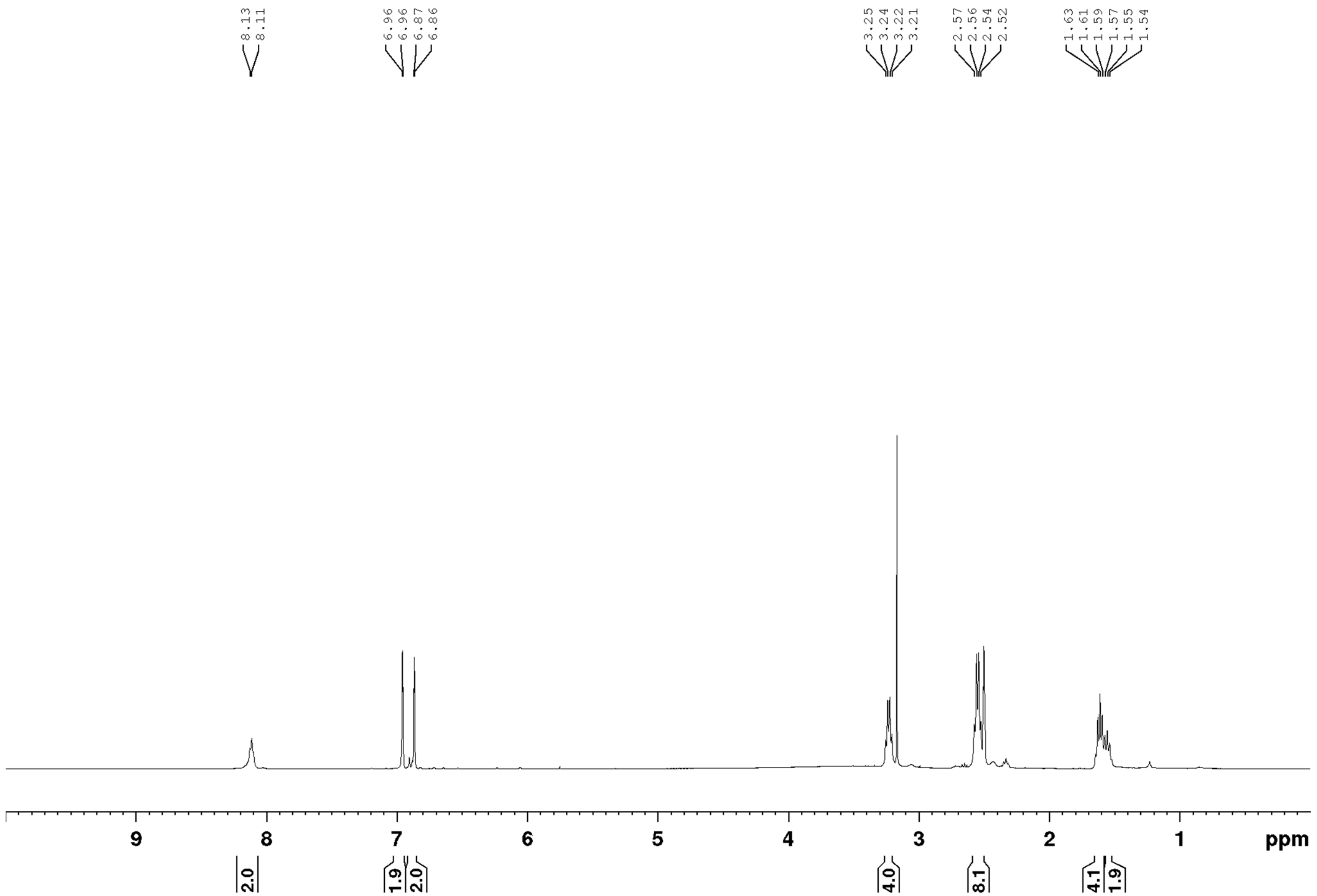

Figure x: ^{1}H NMR spectra of **18f** (400 MHz; DMSO-d_6).

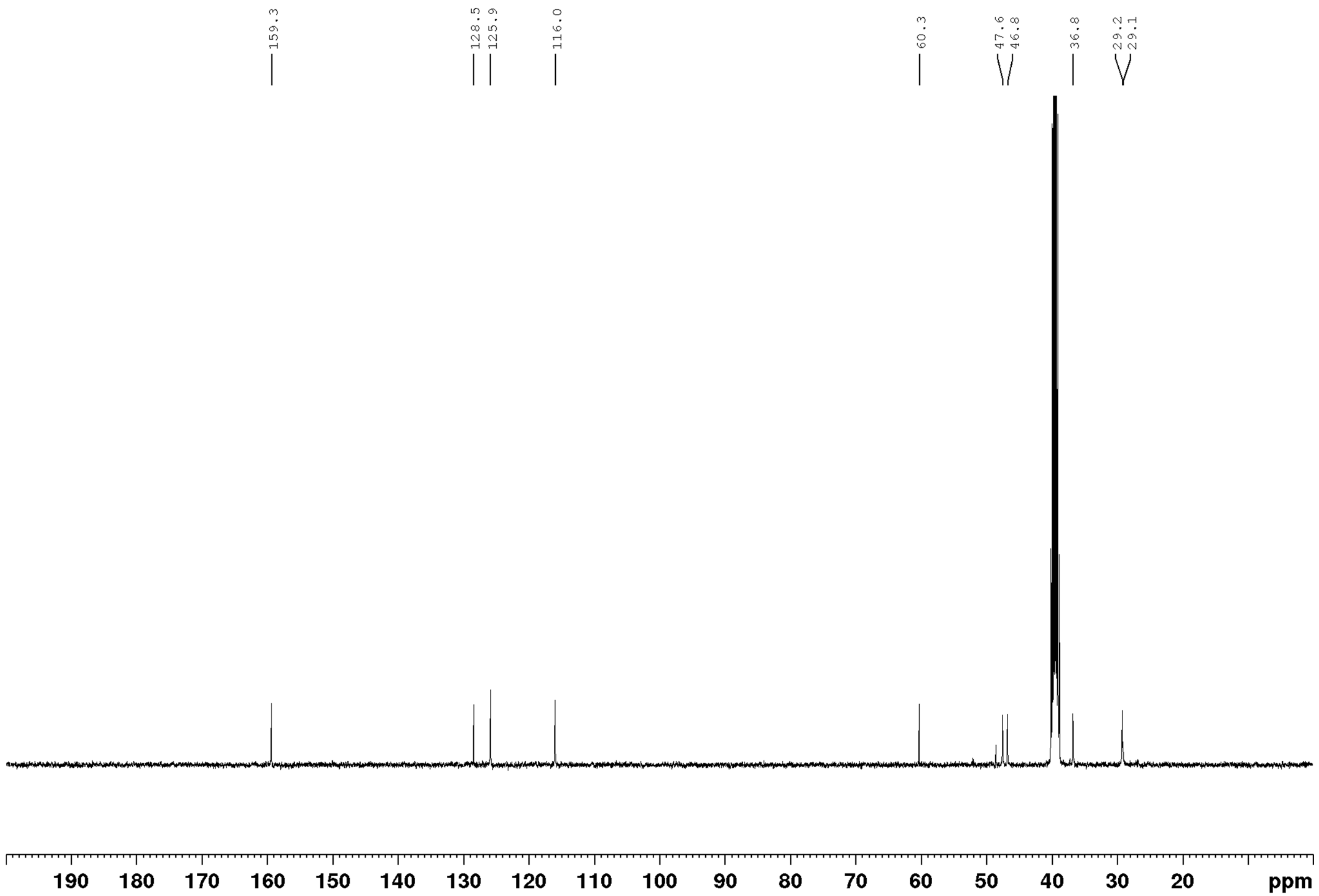

Figure x: ^{13}C NMR spectra of **18f** (100 MHz; DMSO-d_6).

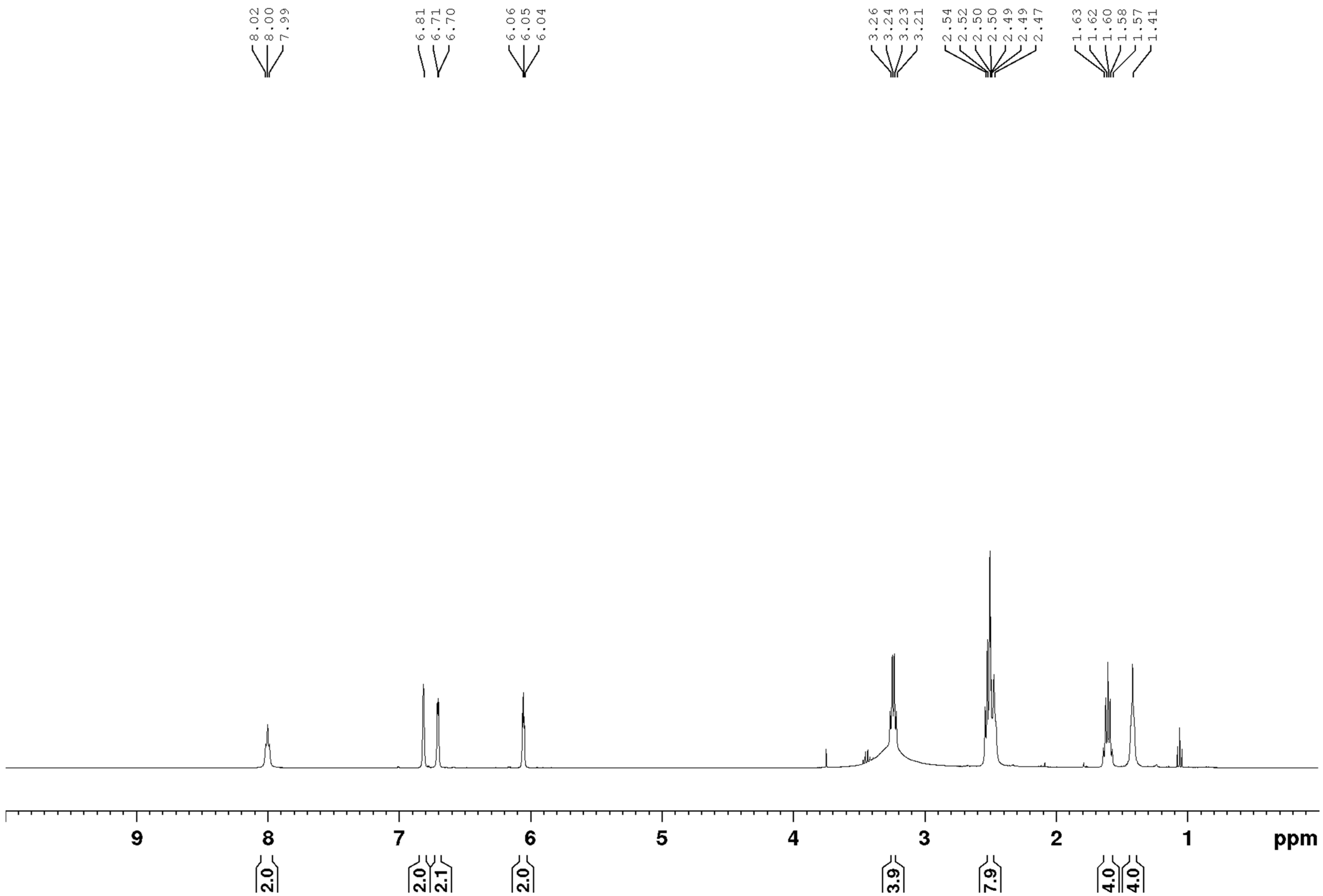

Figure x: ^{1}H NMR spectra of **19a** (400 MHz; DMSO-d_6).

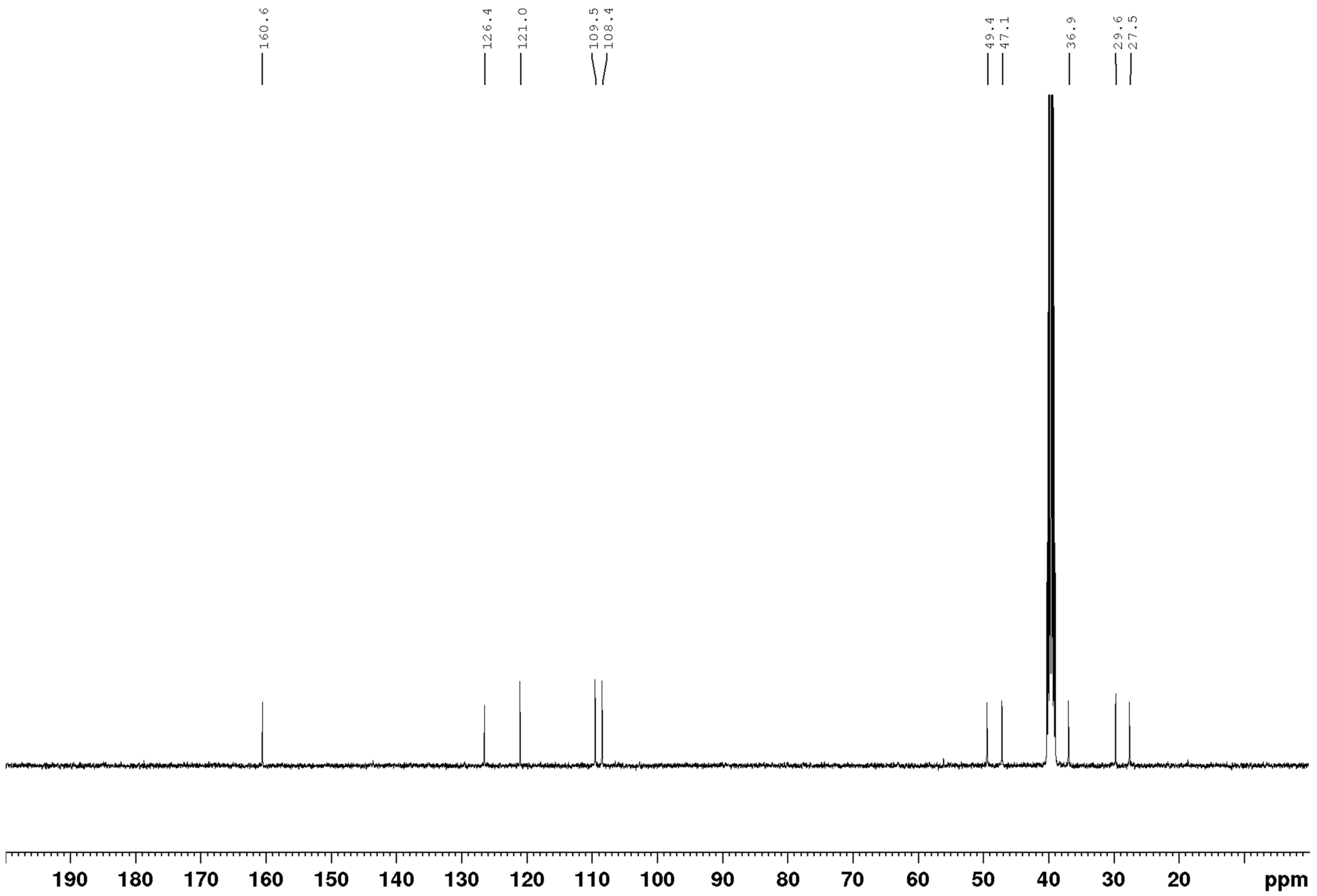

Figure x: ^{13}C NMR spectra of **19a** (100 MHz; DMSO-d_6).

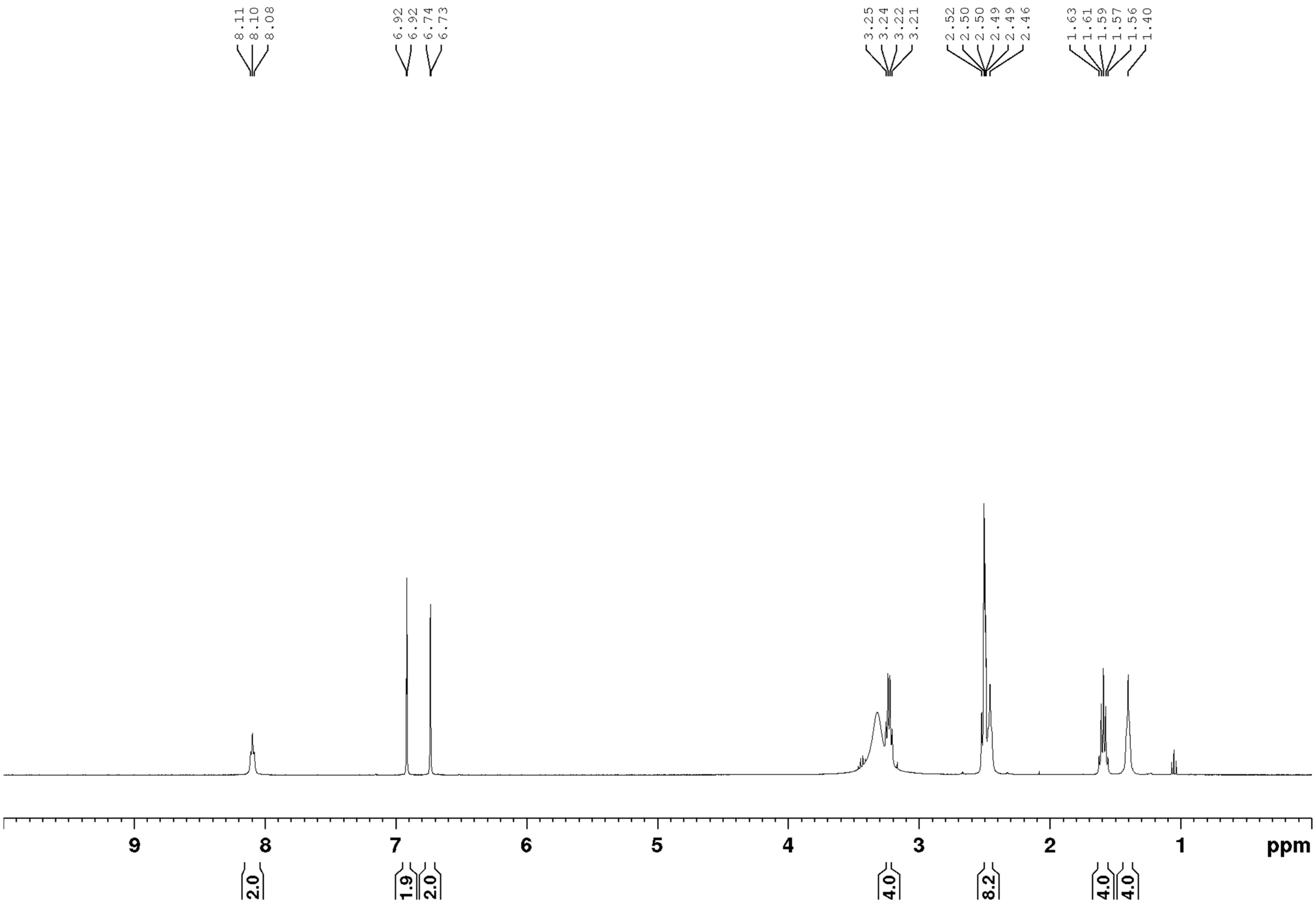

Figure x: ^{1}H NMR spectra of **19b** (400 MHz; DMSO-d_6).

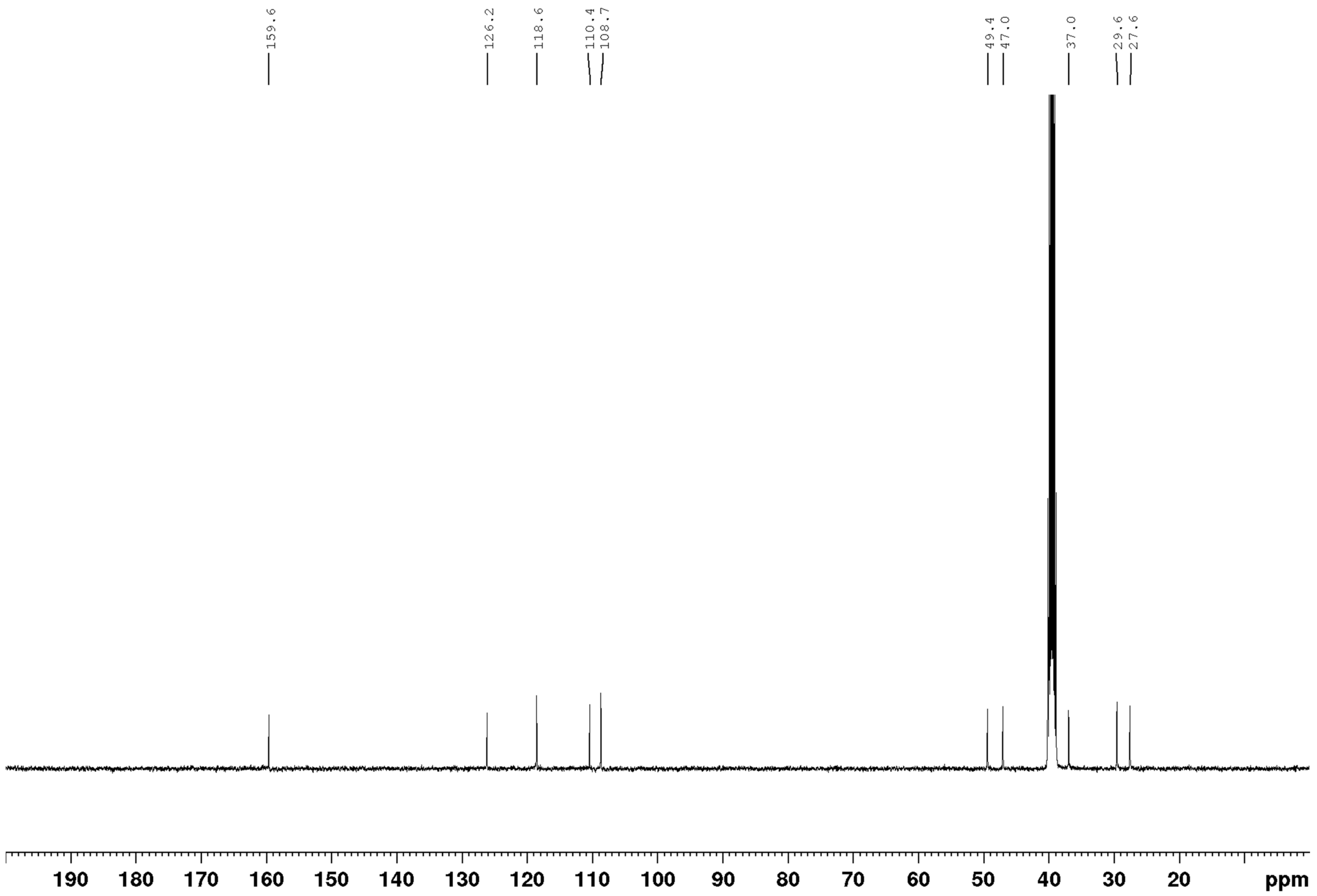

Figure x: ^{13}C NMR spectra of **19b** (100 MHz; DMSO-d_6).

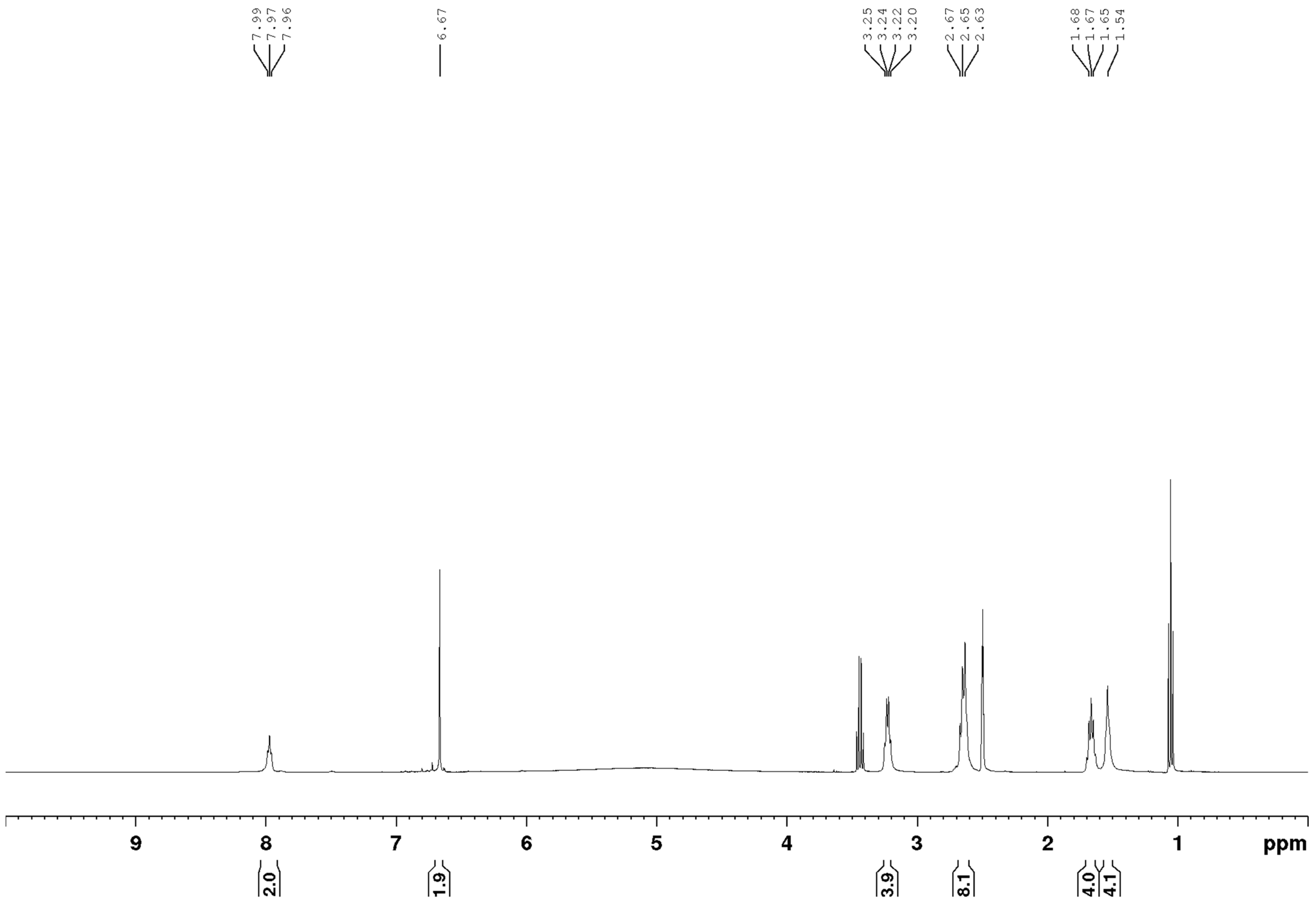

Figure x: ^{1}H NMR spectra of **19c** (400 MHz; DMSO-d_6).

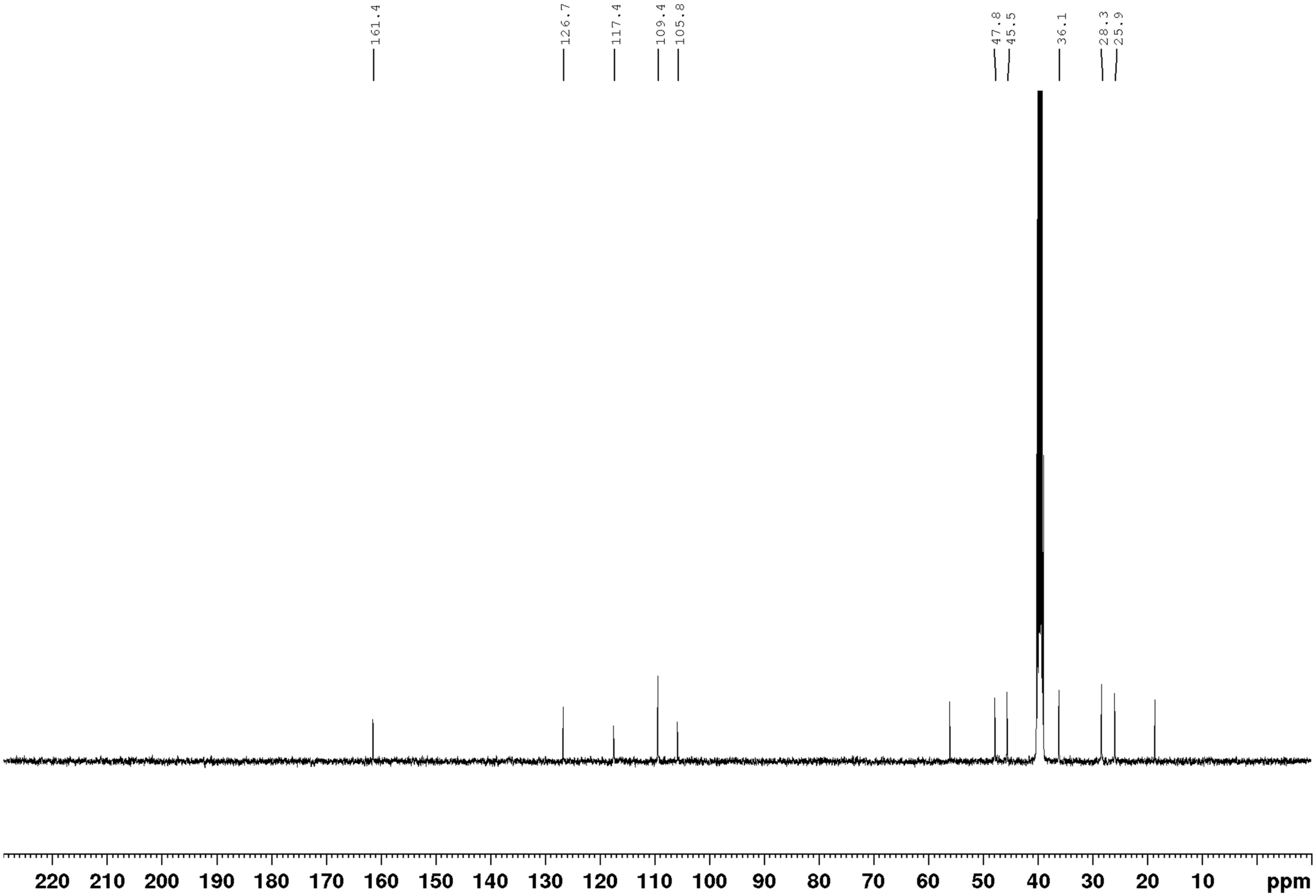

Figure x: ^{13}C NMR spectra of **19c** (100 MHz; DMSO-d_6).

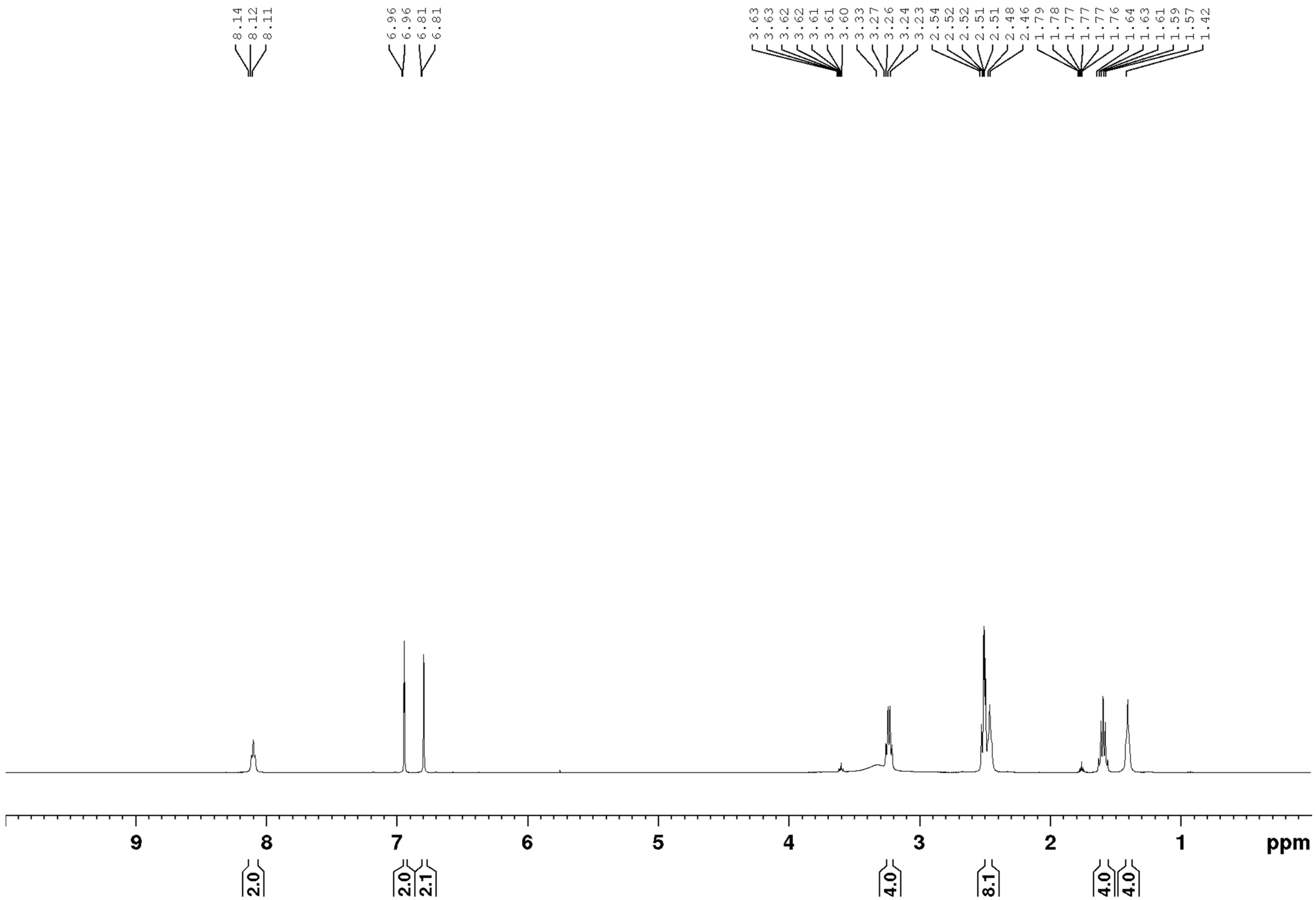

Figure x: ^{1}H NMR spectra of **19d** (400 MHz; DMSO-d_6).

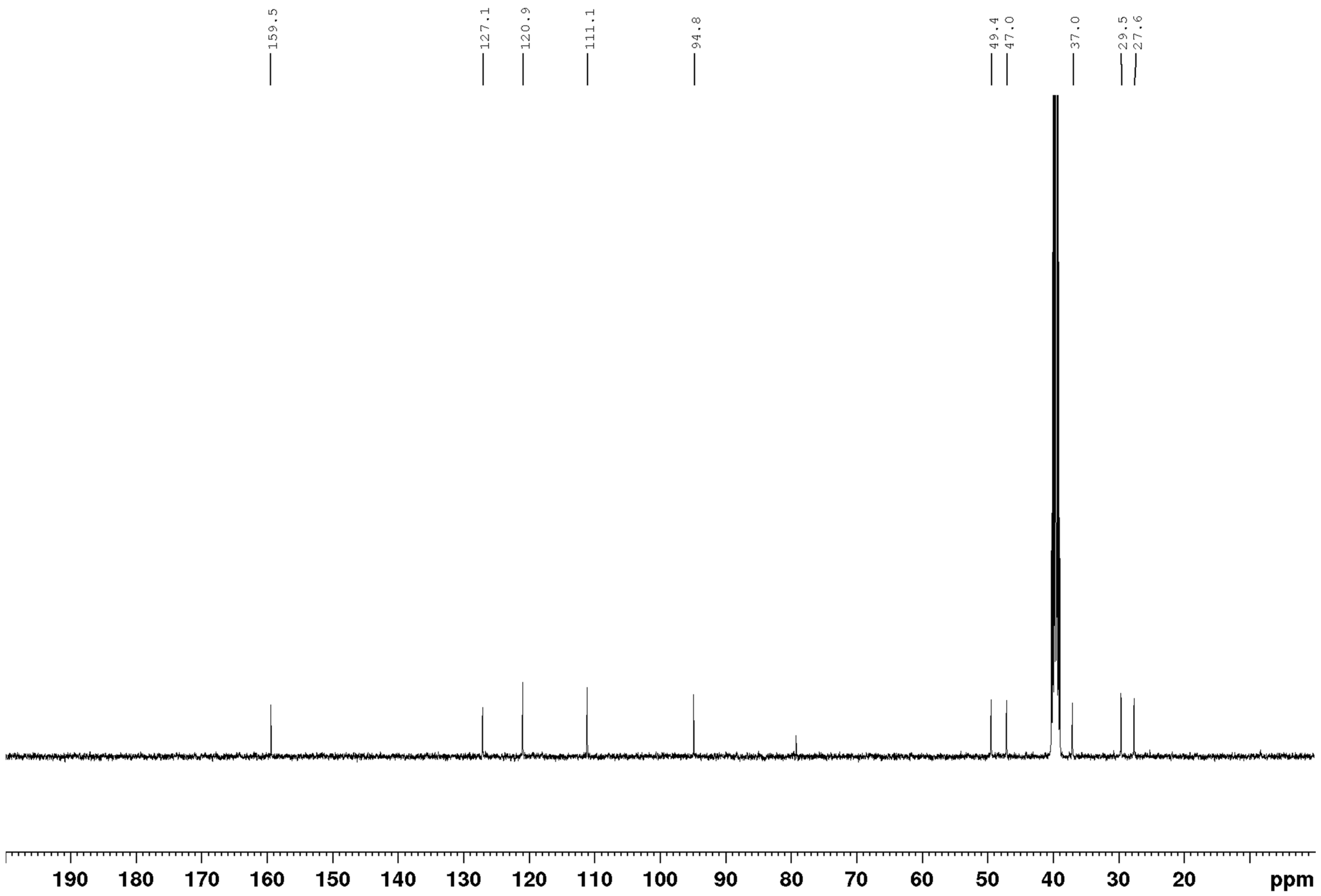

Figure x: ^{13}C NMR spectra of **19d** (100 MHz; DMSO-d_6).

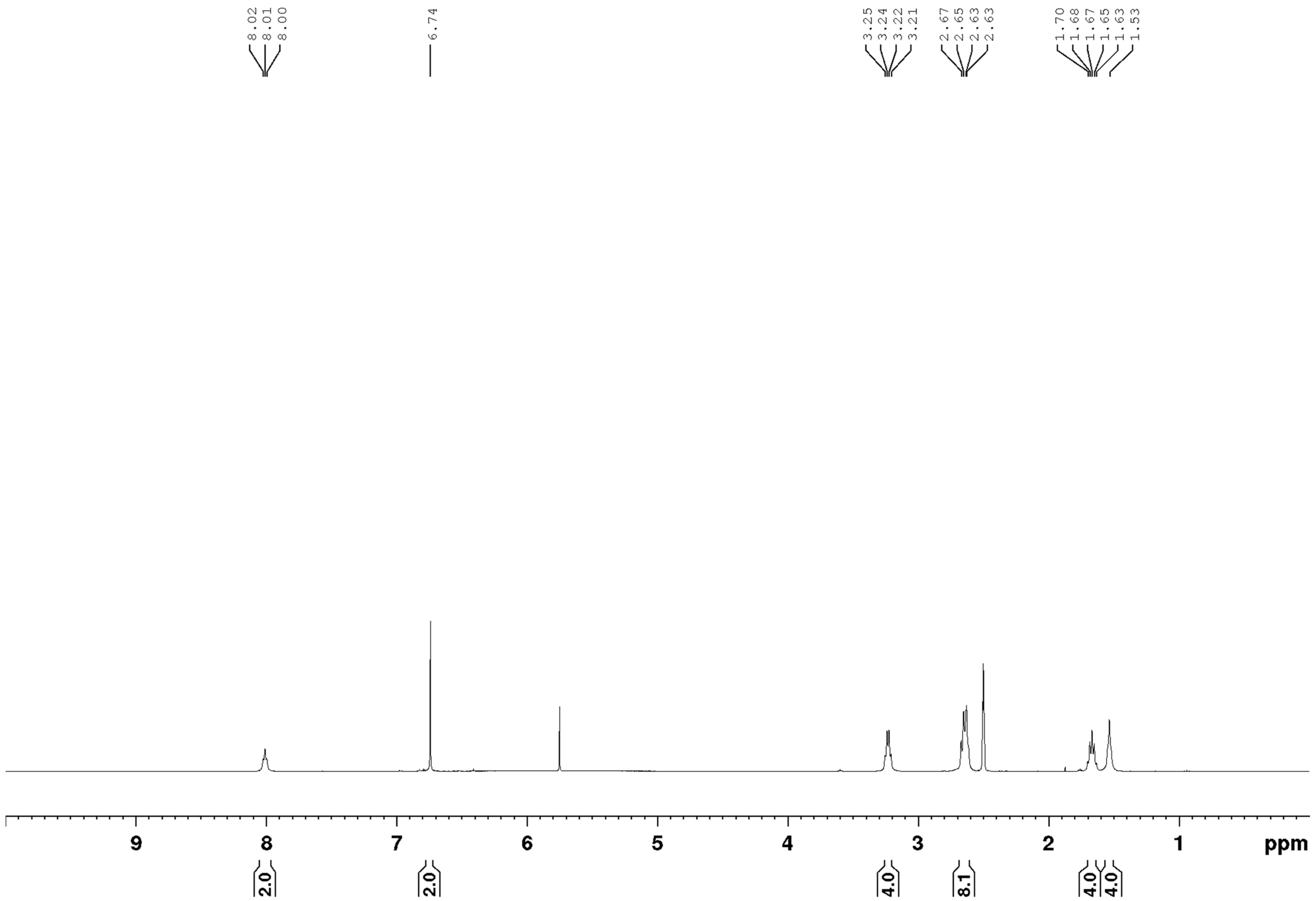

Figure x: ^{1}H NMR spectra of **19e** (400 MHz; DMSO-d_6).

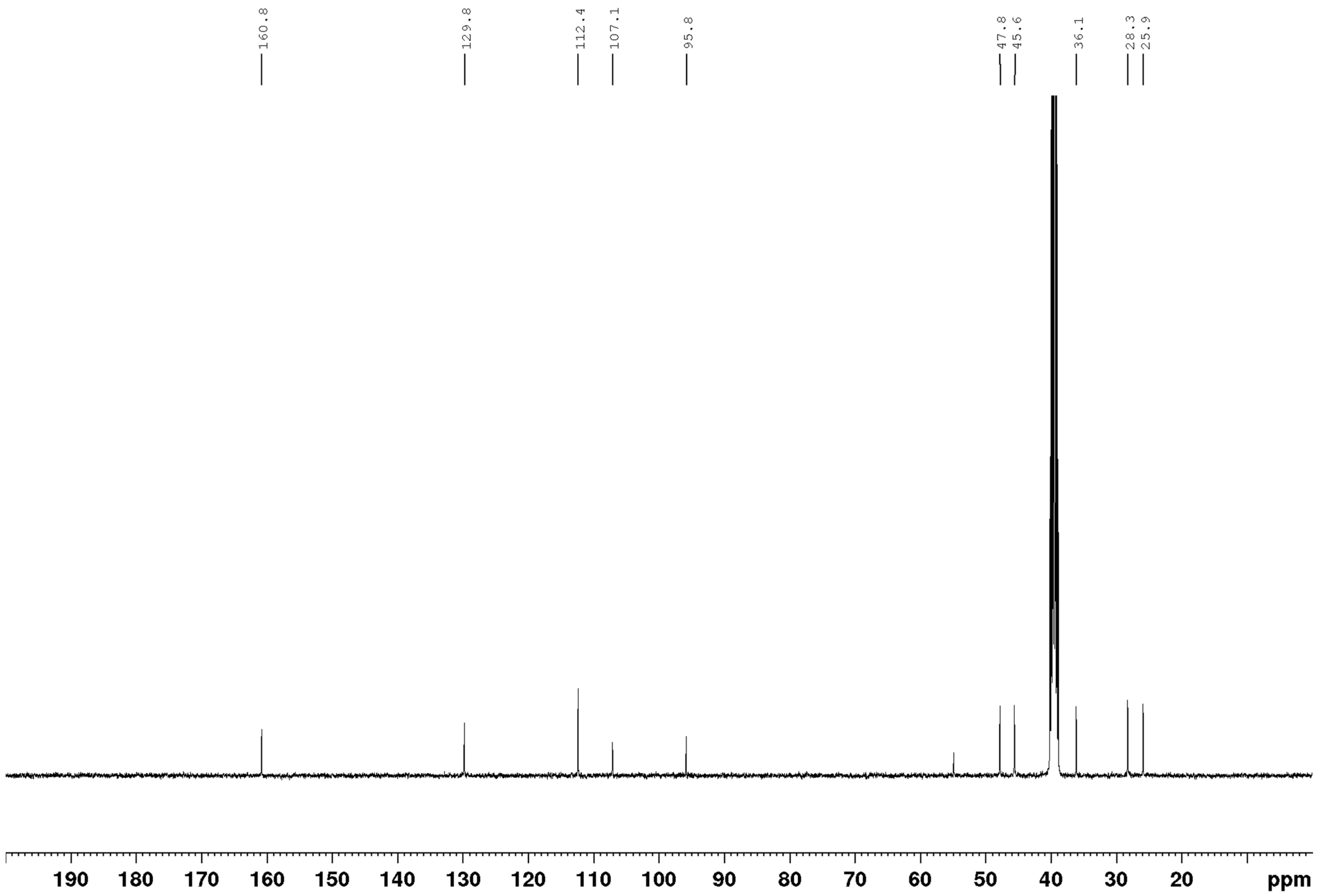

Figure x: ^{13}C NMR spectra of **19e** (100 MHz; DMSO-d_6).

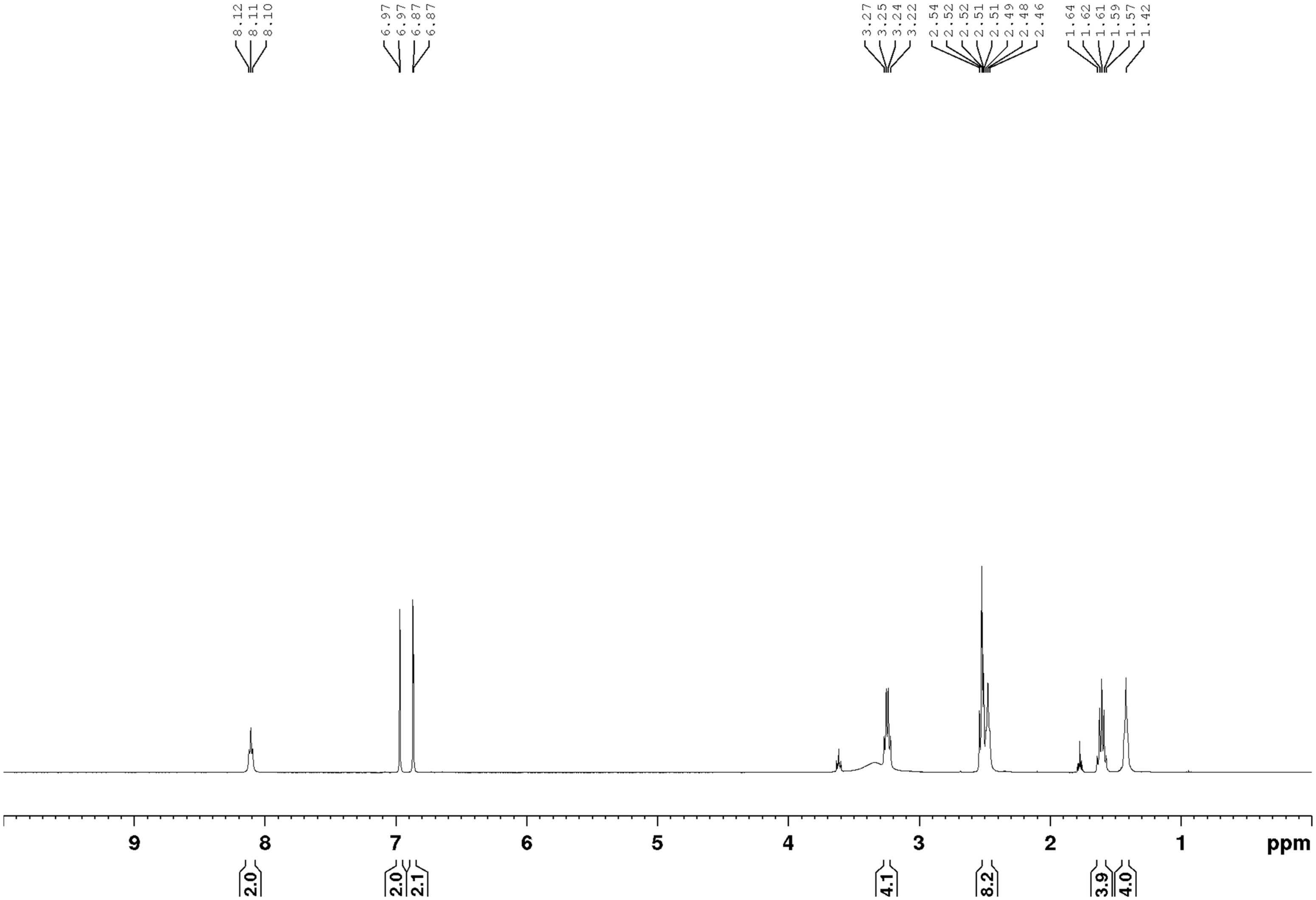

Figure x: ^{1}H NMR spectra of **19f** (400 MHz; DMSO-d_6).

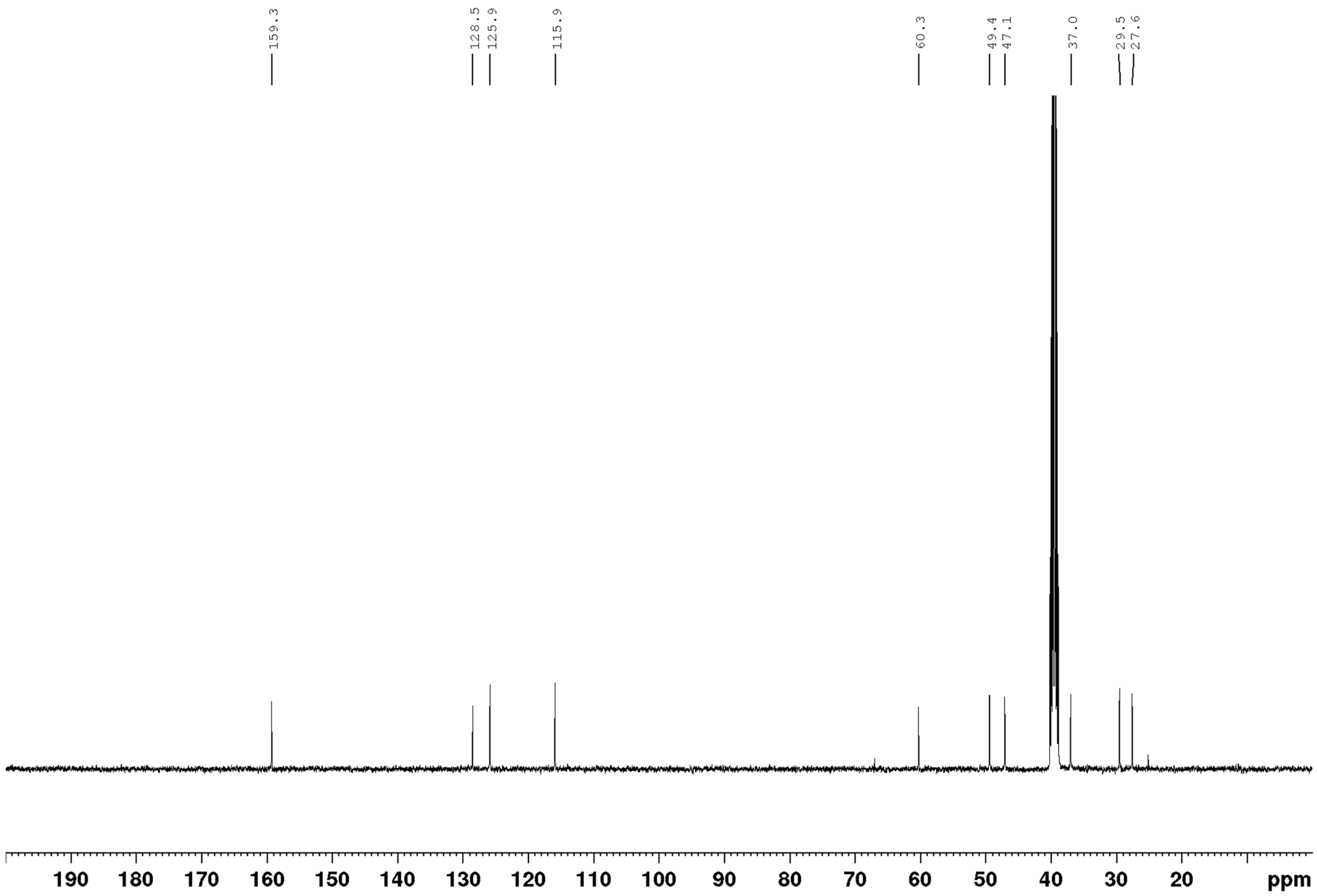

Figure x: ^{13}C NMR spectra of **19f** (100 MHz; DMSO-d_6).

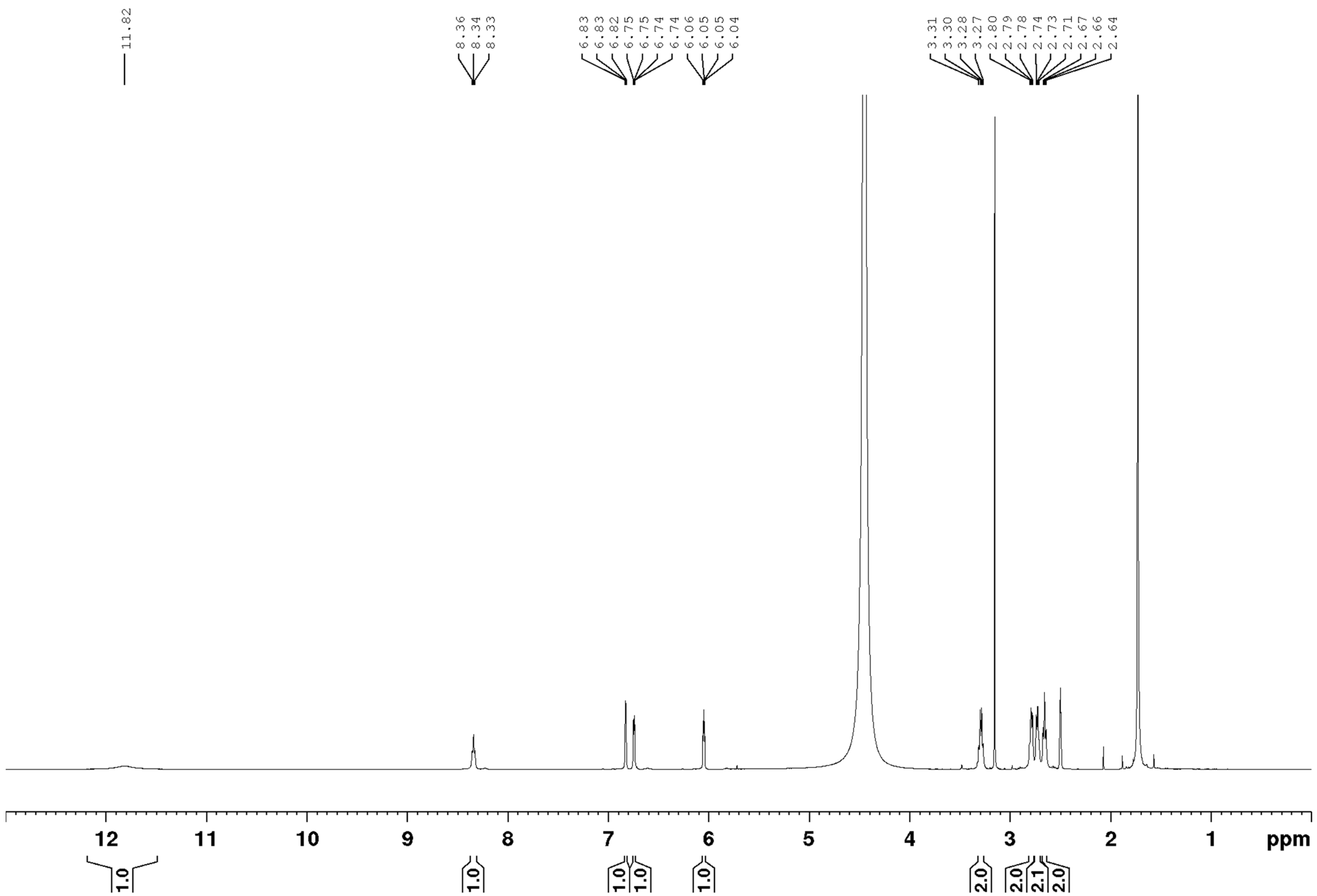

Figure x: ^{1}H NMR spectra of **20** (400 MHz; DMSO-d_6).

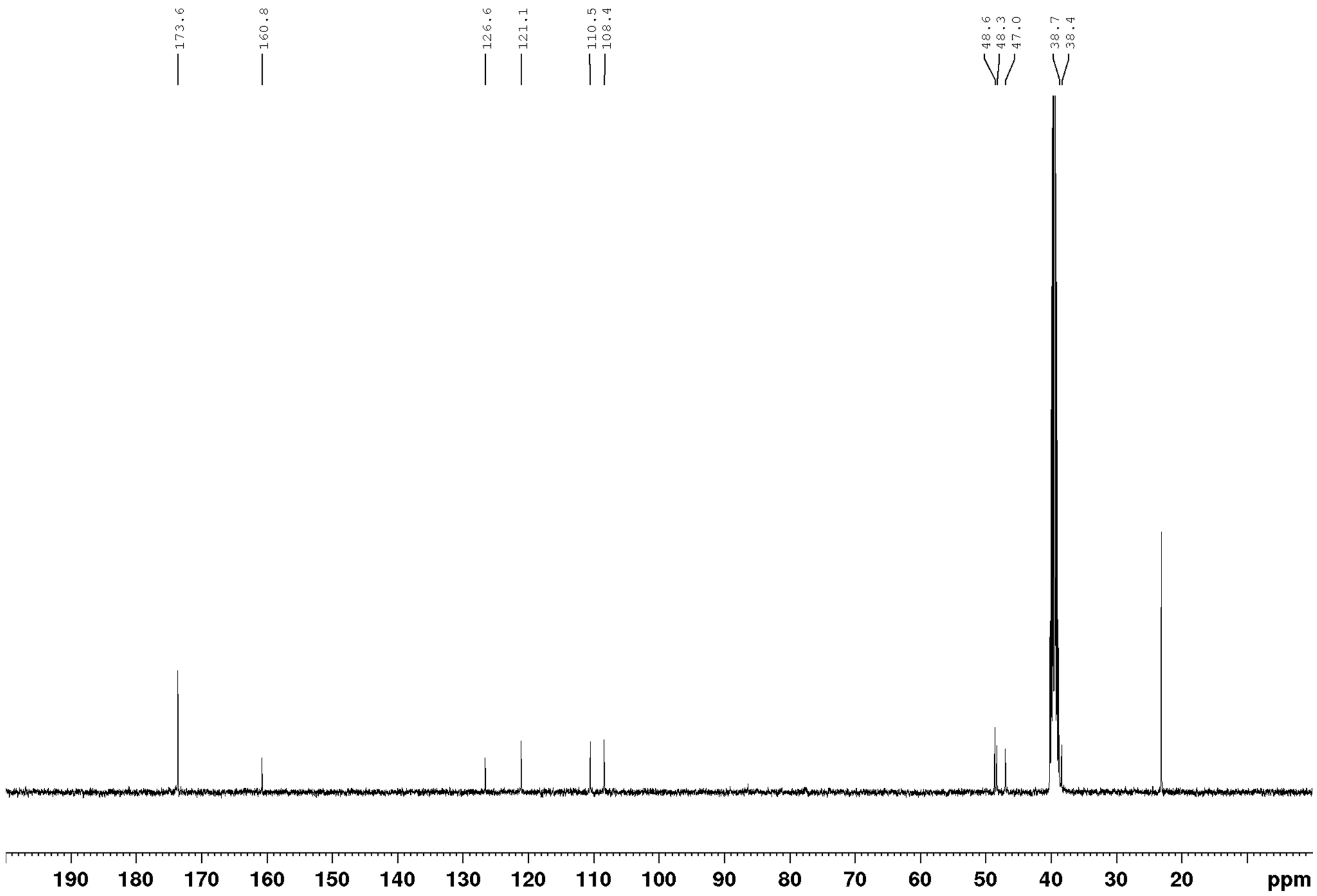

Figure x: ^{13}C NMR spectra of **20** (100 MHz; DMSO-d_6).

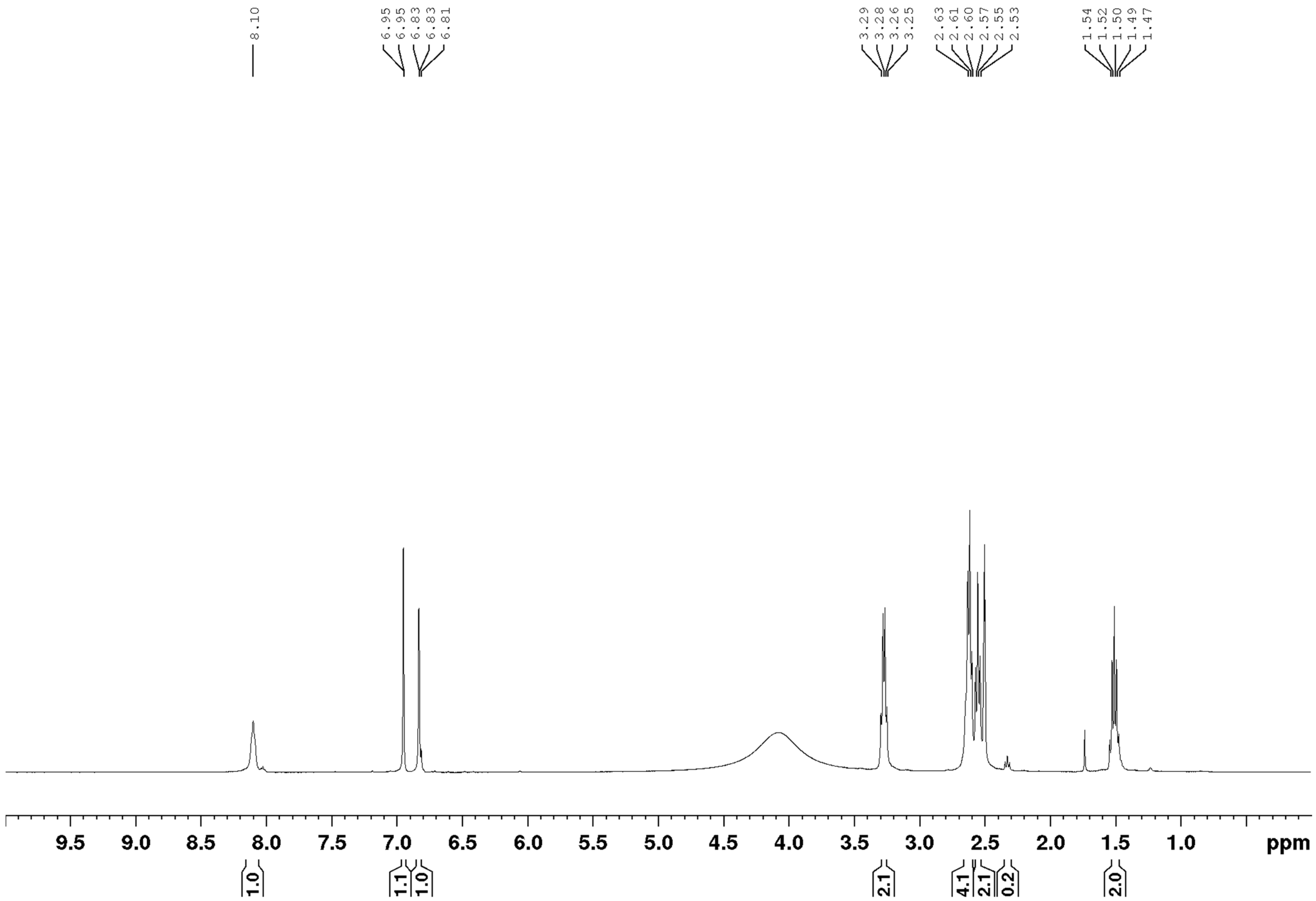

Figure x: ^{1}H NMR spectra of **21** (400 MHz; DMSO-d_6).

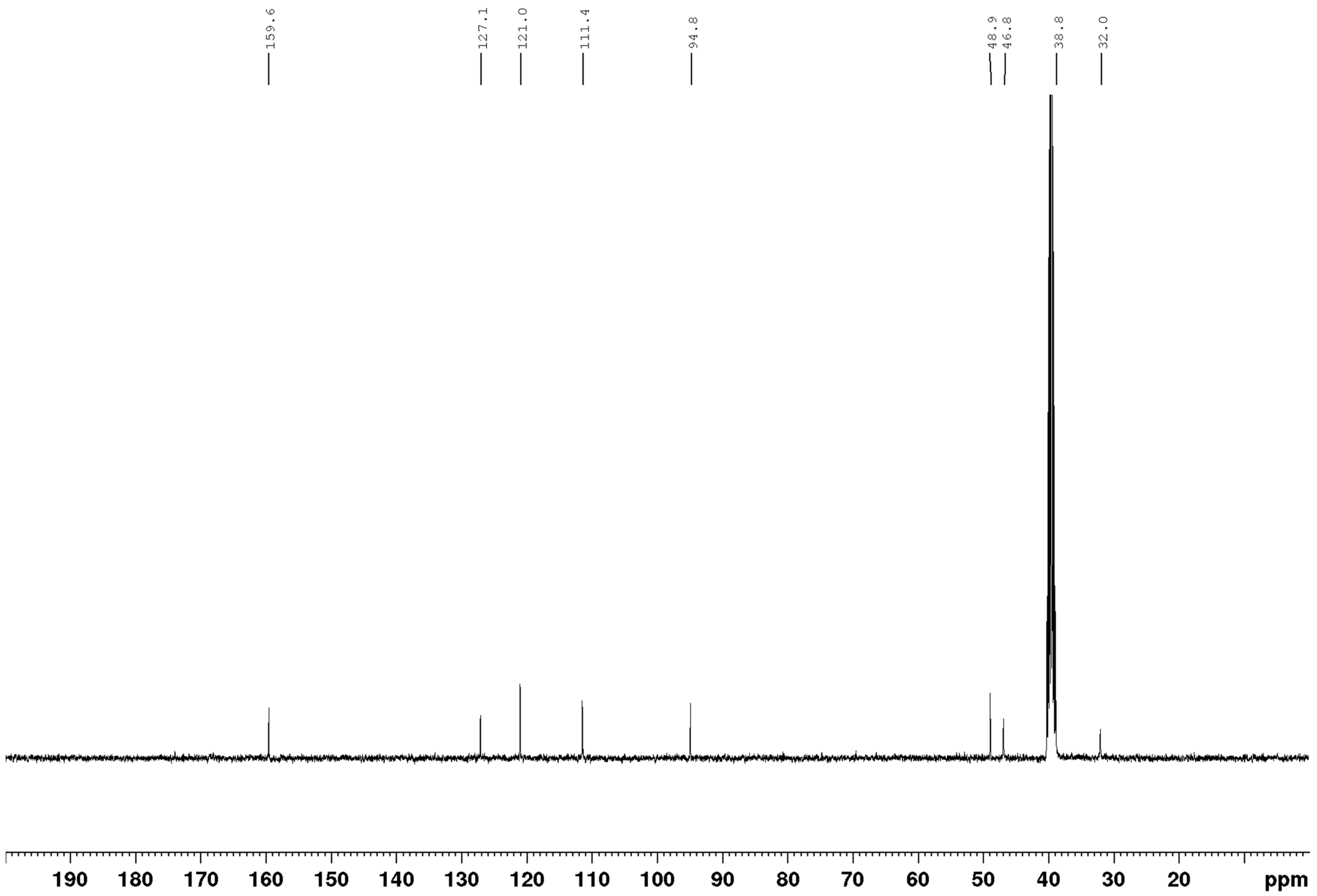

Figure x: ^{13}C NMR spectra of **21** (100 MHz; DMSO-d_6).

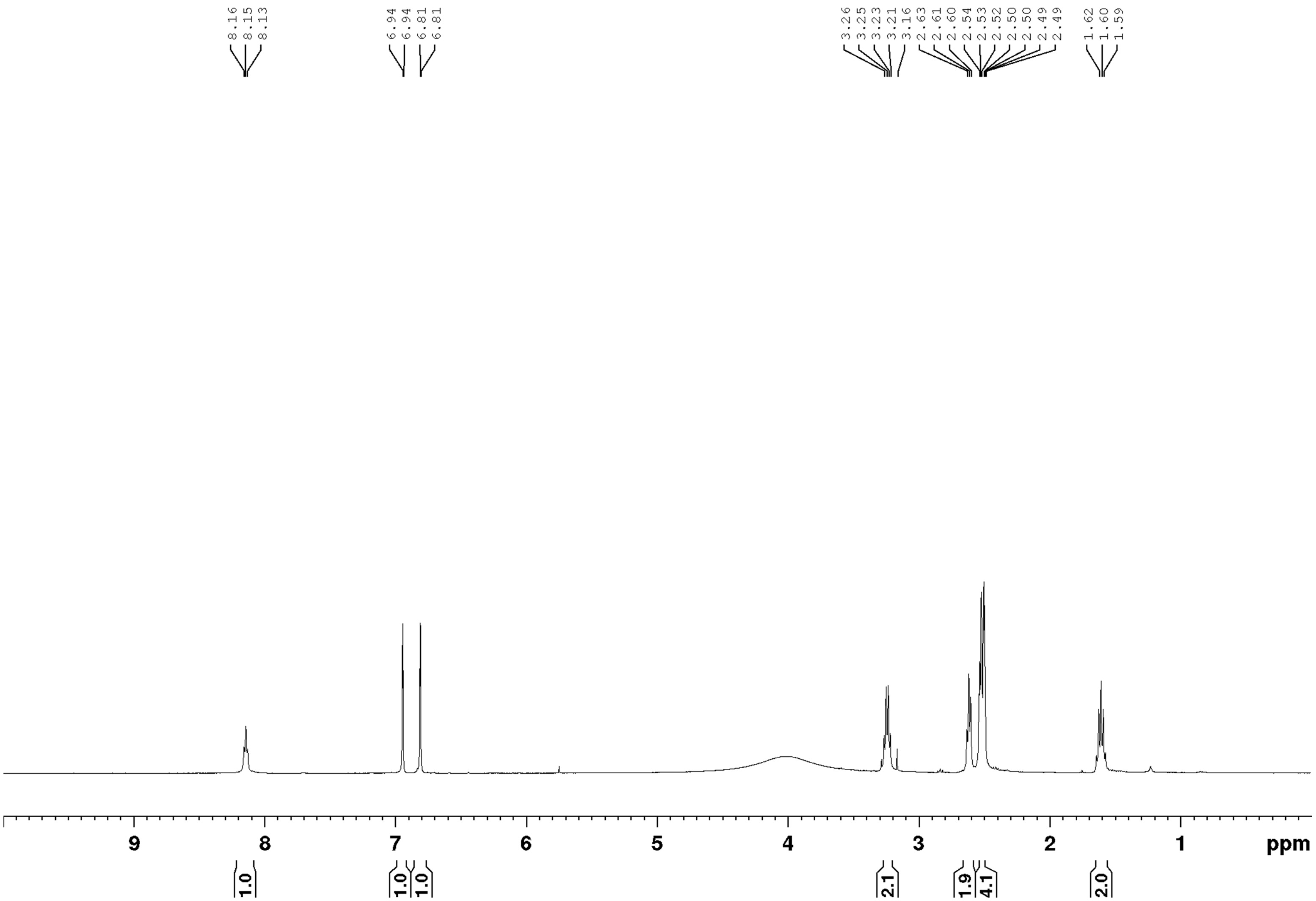

Figure x: ^{1}H NMR spectra of **22** (400 MHz; DMSO-d_6).

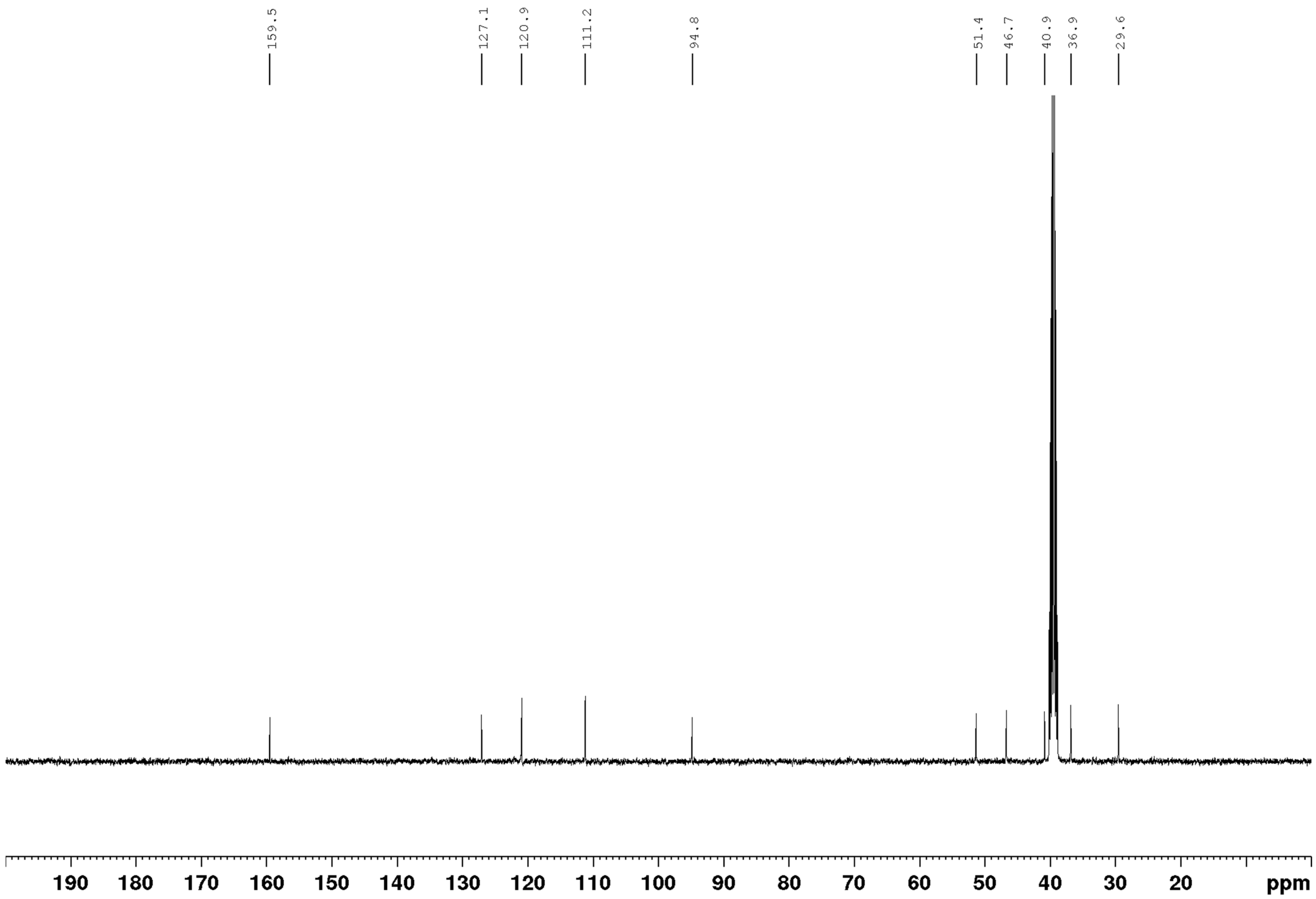

Figure x: ^{13}C NMR spectra of **22** (100 MHz; DMSO-d_6).

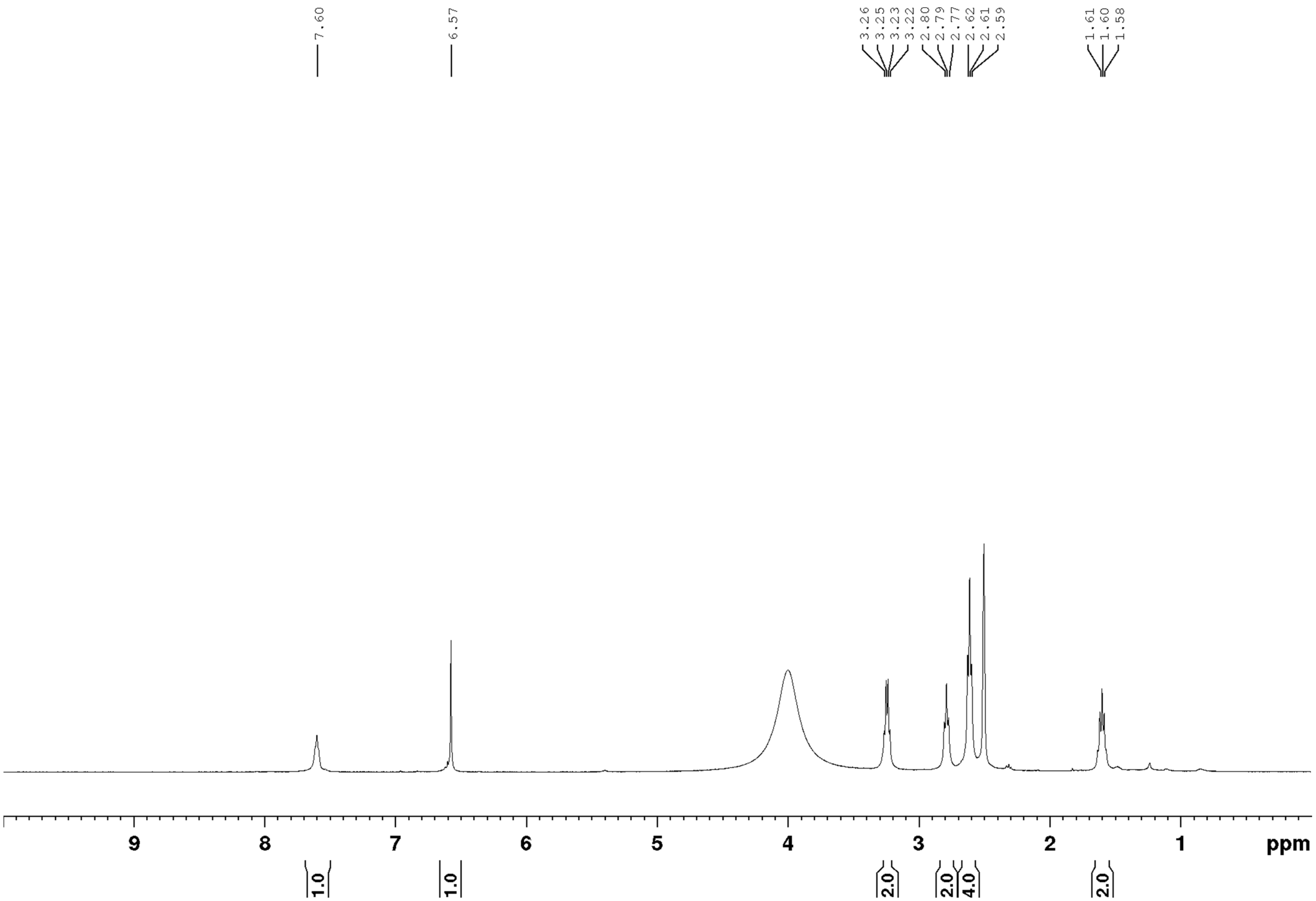

Figure x: ^{1}H NMR spectra of **23** (400 MHz; DMSO-d_6).

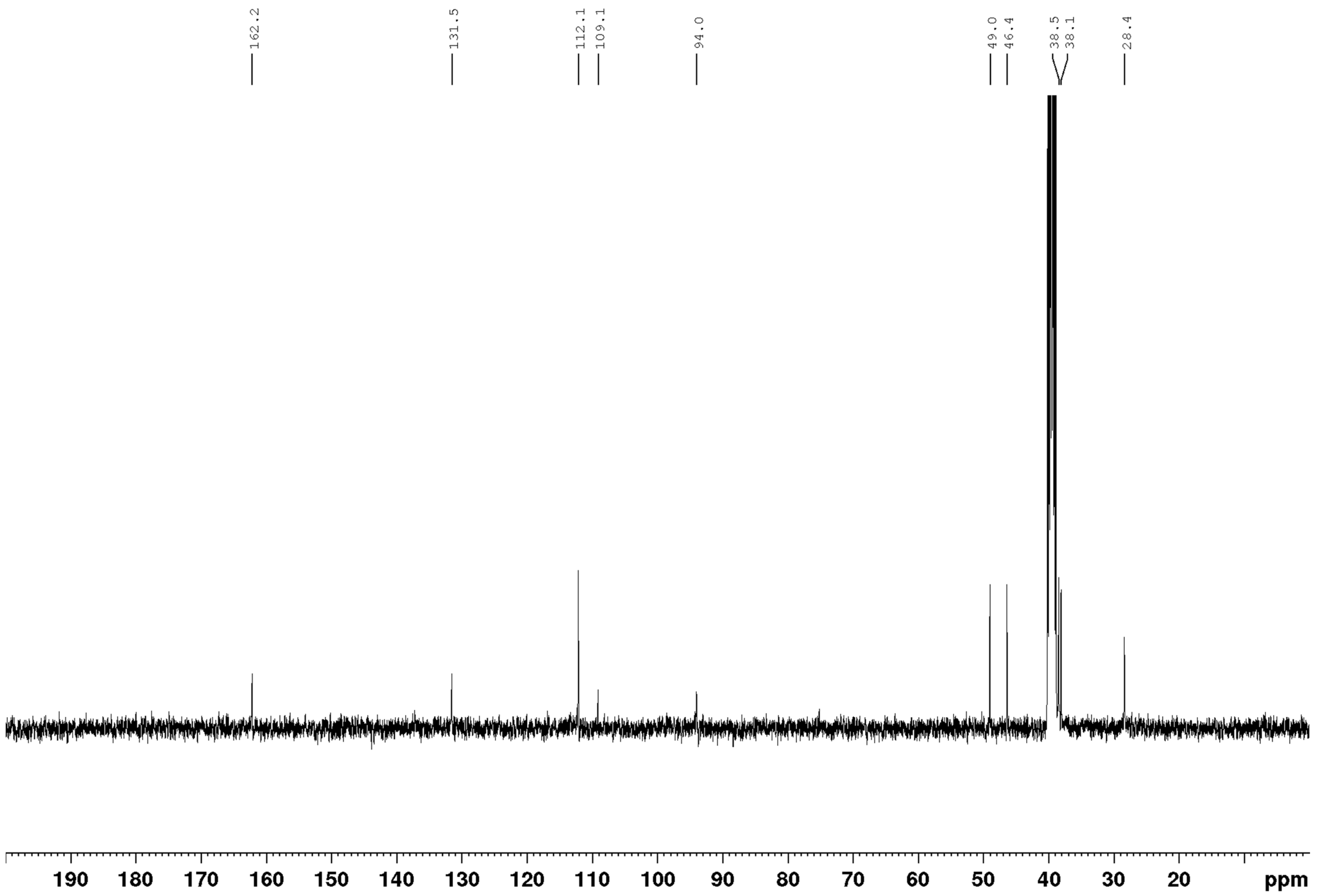

Figure x: ^{13}C NMR spectra of **23** (100 MHz; DMSO-d_6).

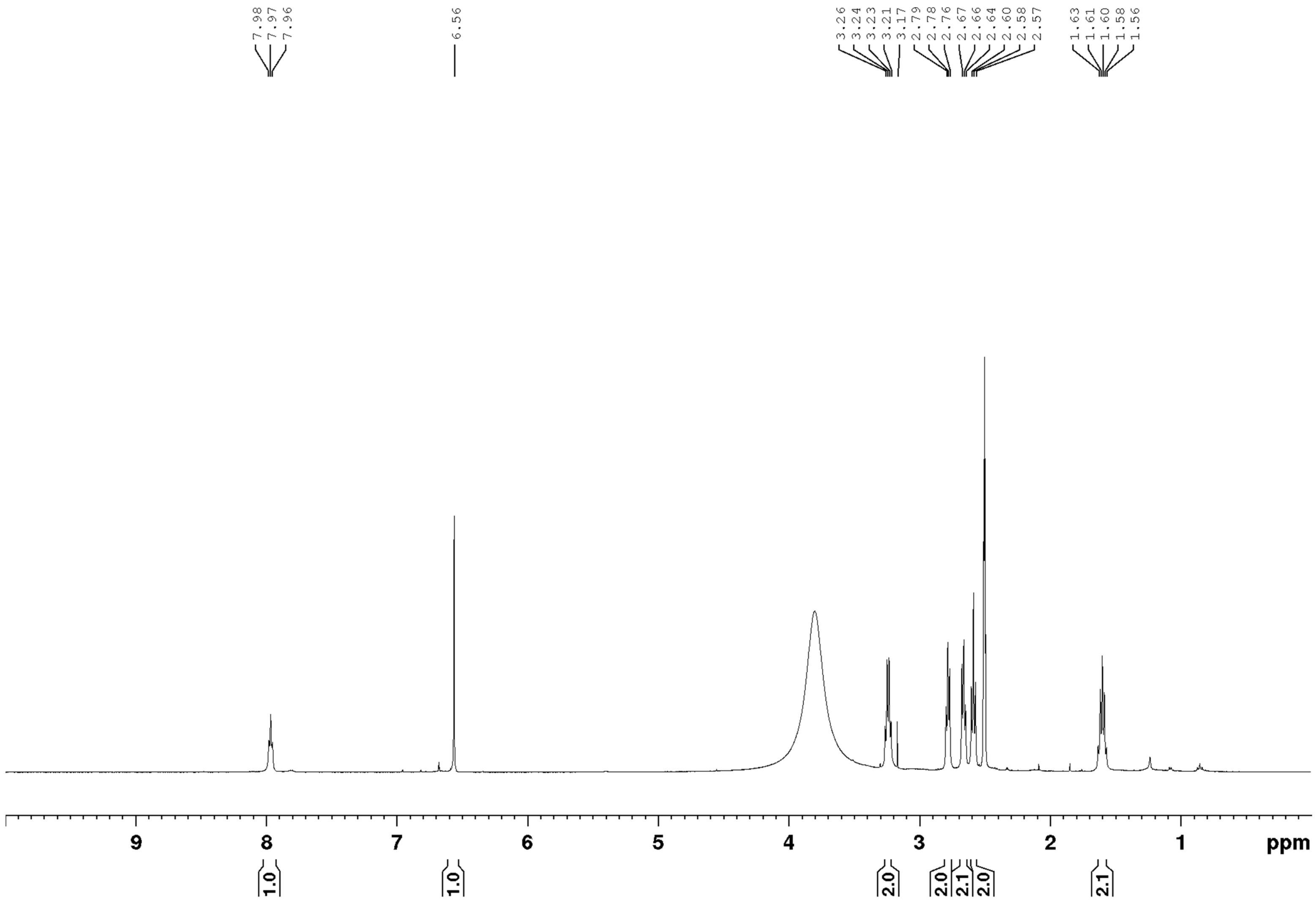

Figure x: ^{1}H NMR spectra of **24** (400 MHz; DMSO-d_6).

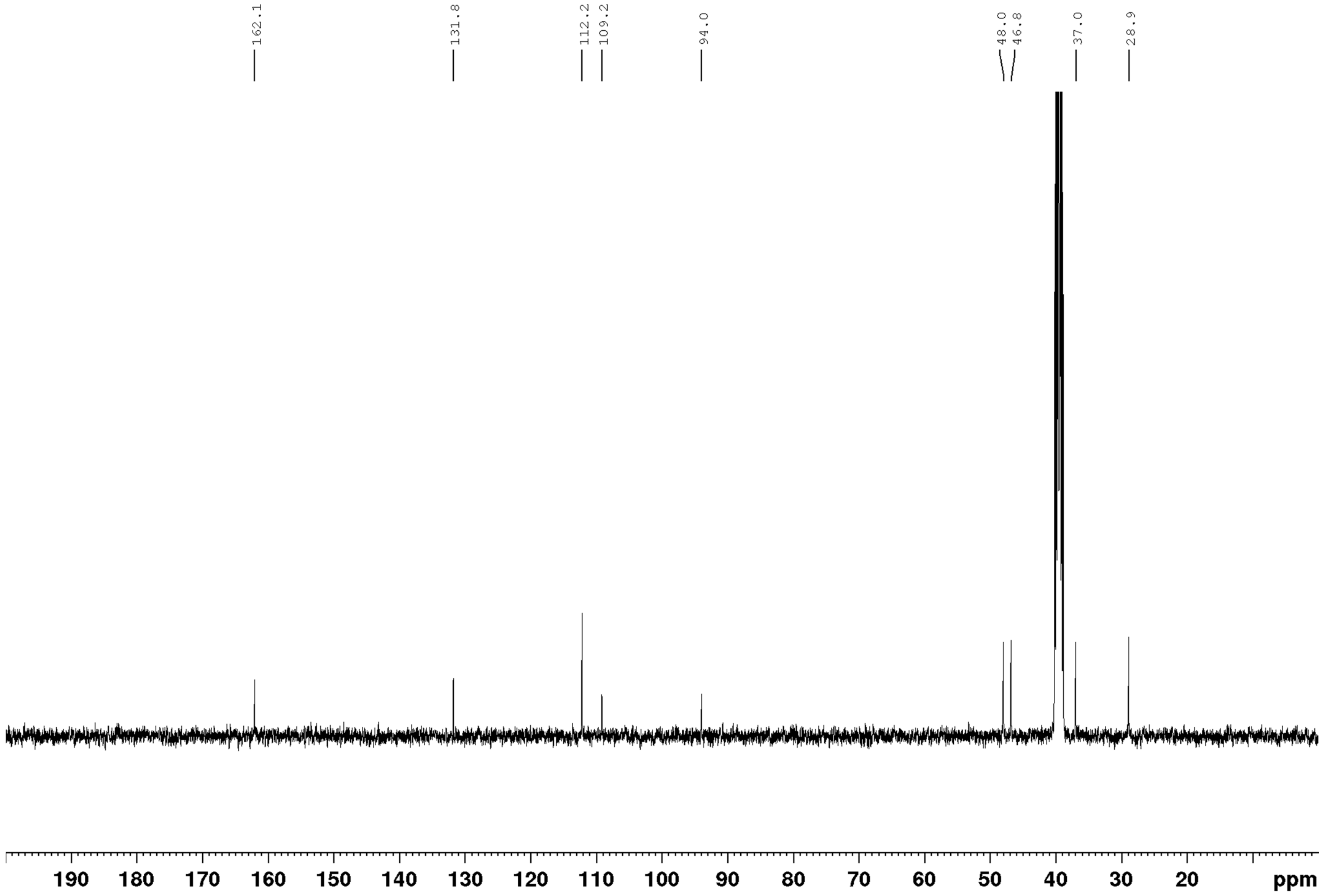

Figure x: ^{13}C NMR spectra of **24** (100 MHz; DMSO-d_6).

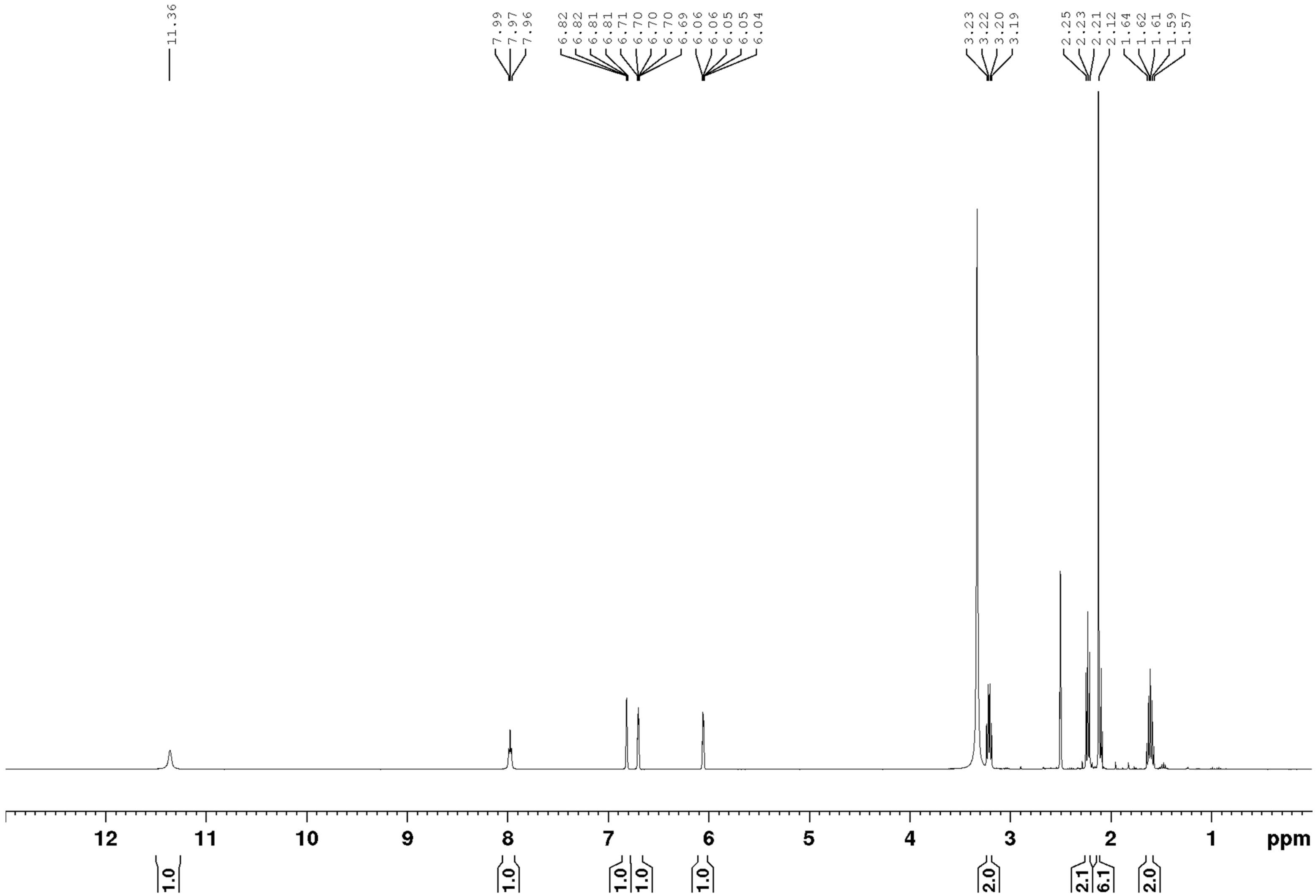

Figure x: ^{1}H NMR spectra of **27a** (400 MHz; DMSO-d_6).

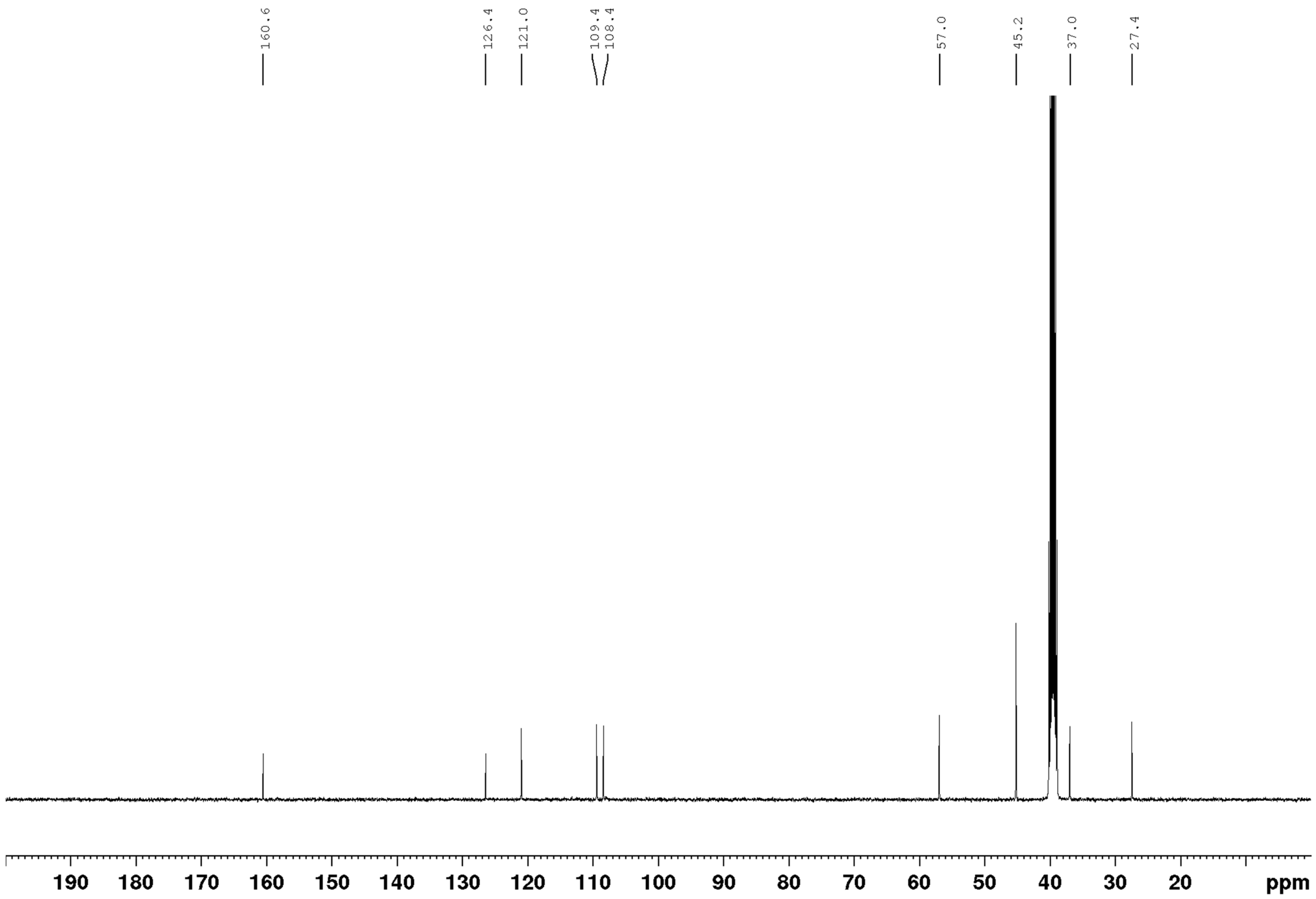

Figure x: ^{13}C NMR spectra of **27a** (100 MHz; DMSO-*d*$_6$).

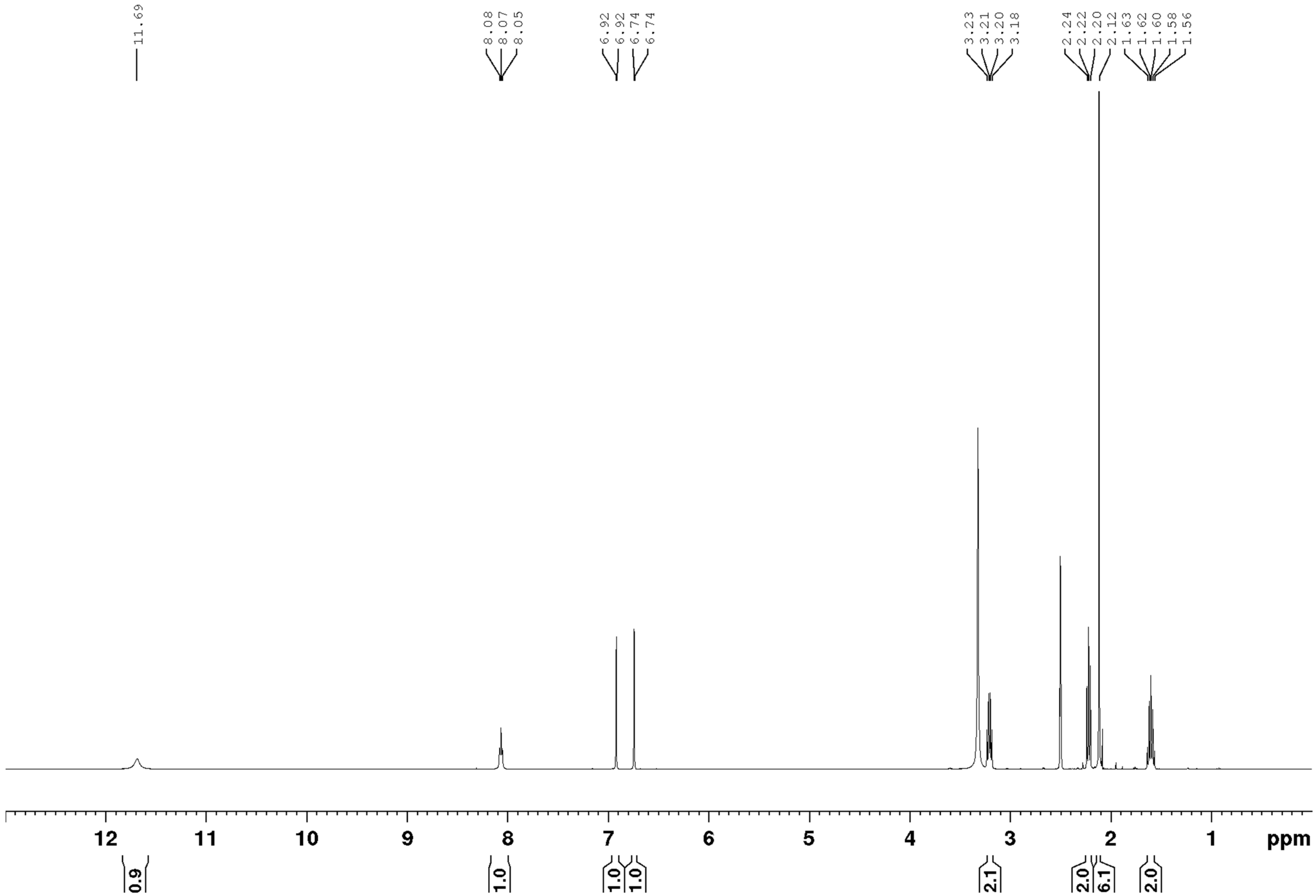

Figure x: ^{1}H NMR spectra of **27b** (400 MHz; DMSO-d_6).

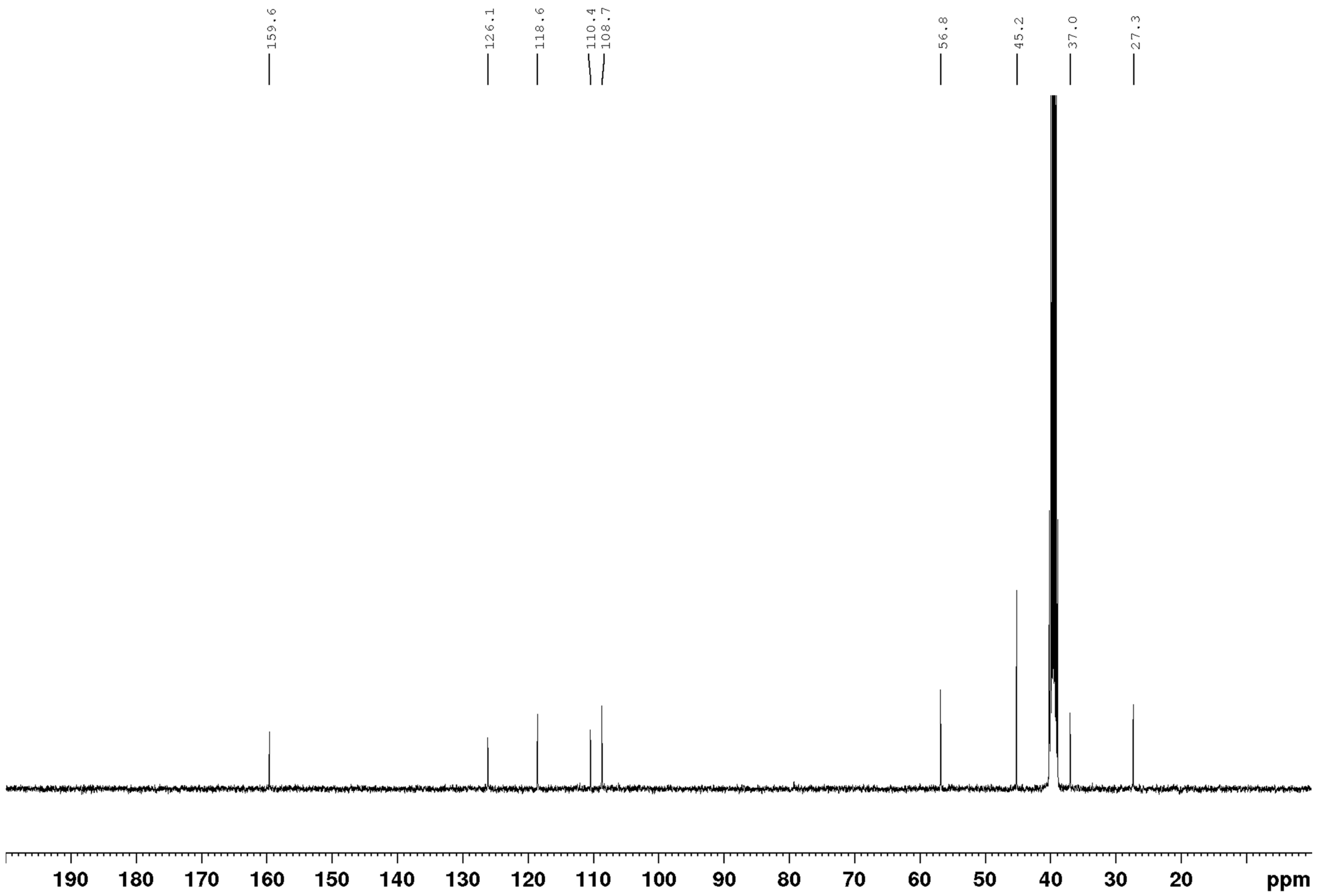

Figure x: ^{13}C NMR spectra of **27b** (100 MHz; DMSO-*d*$_6$).

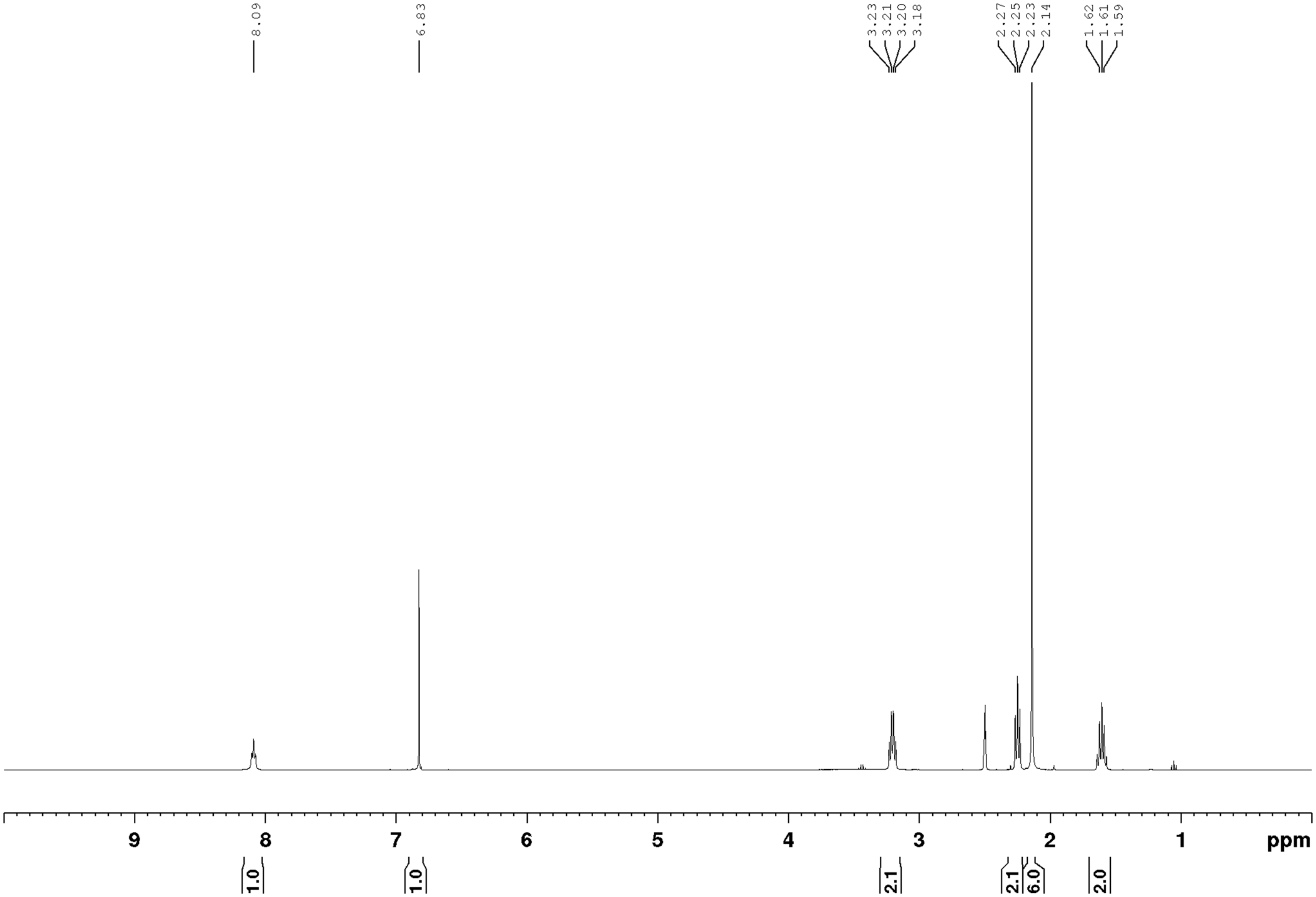

Figure x: ^{1}H NMR spectra of **27c** (400 MHz; DMSO-d_6).

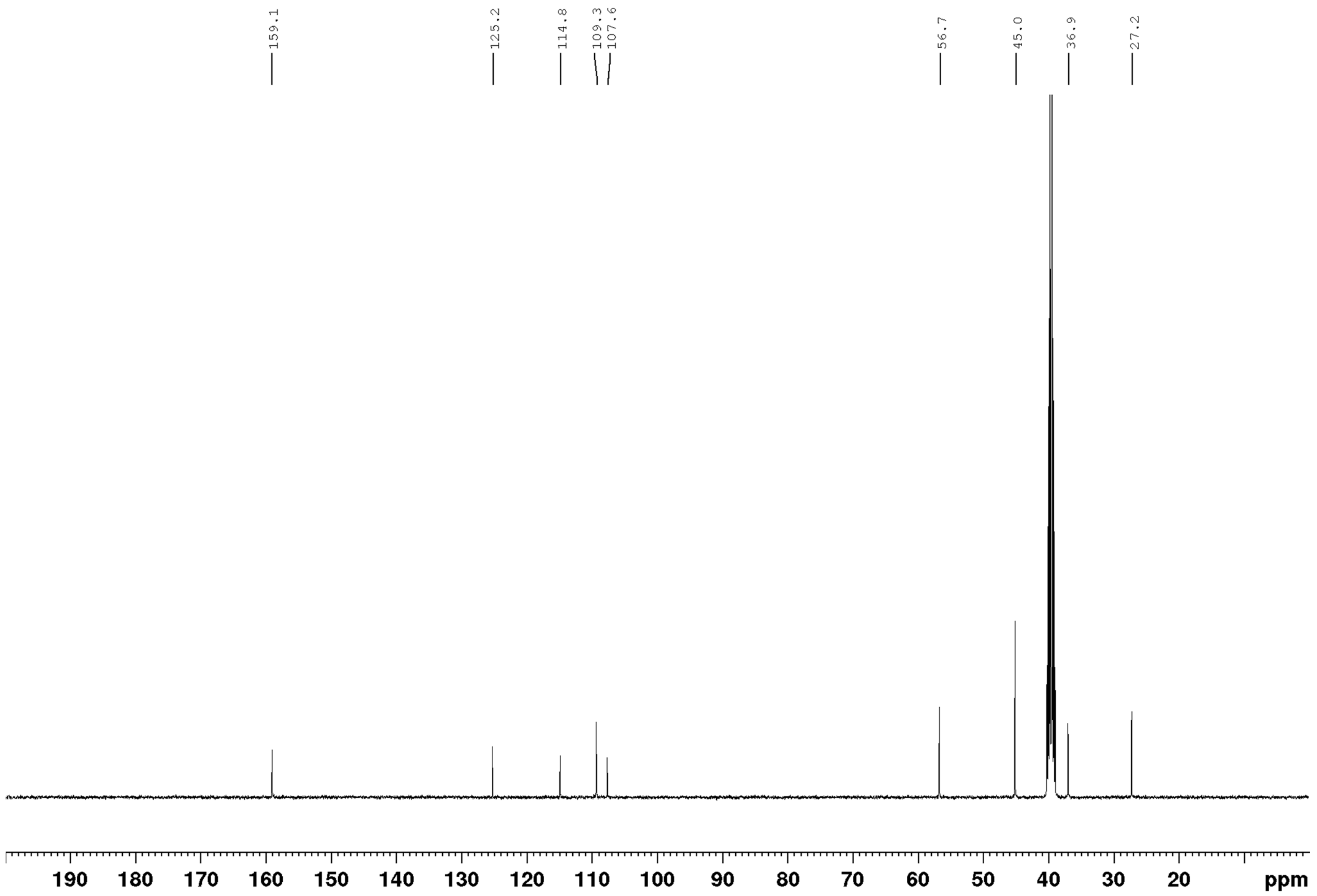

Figure x: ^{13}C NMR spectra of **27c** (100 MHz; DMSO-d_6).

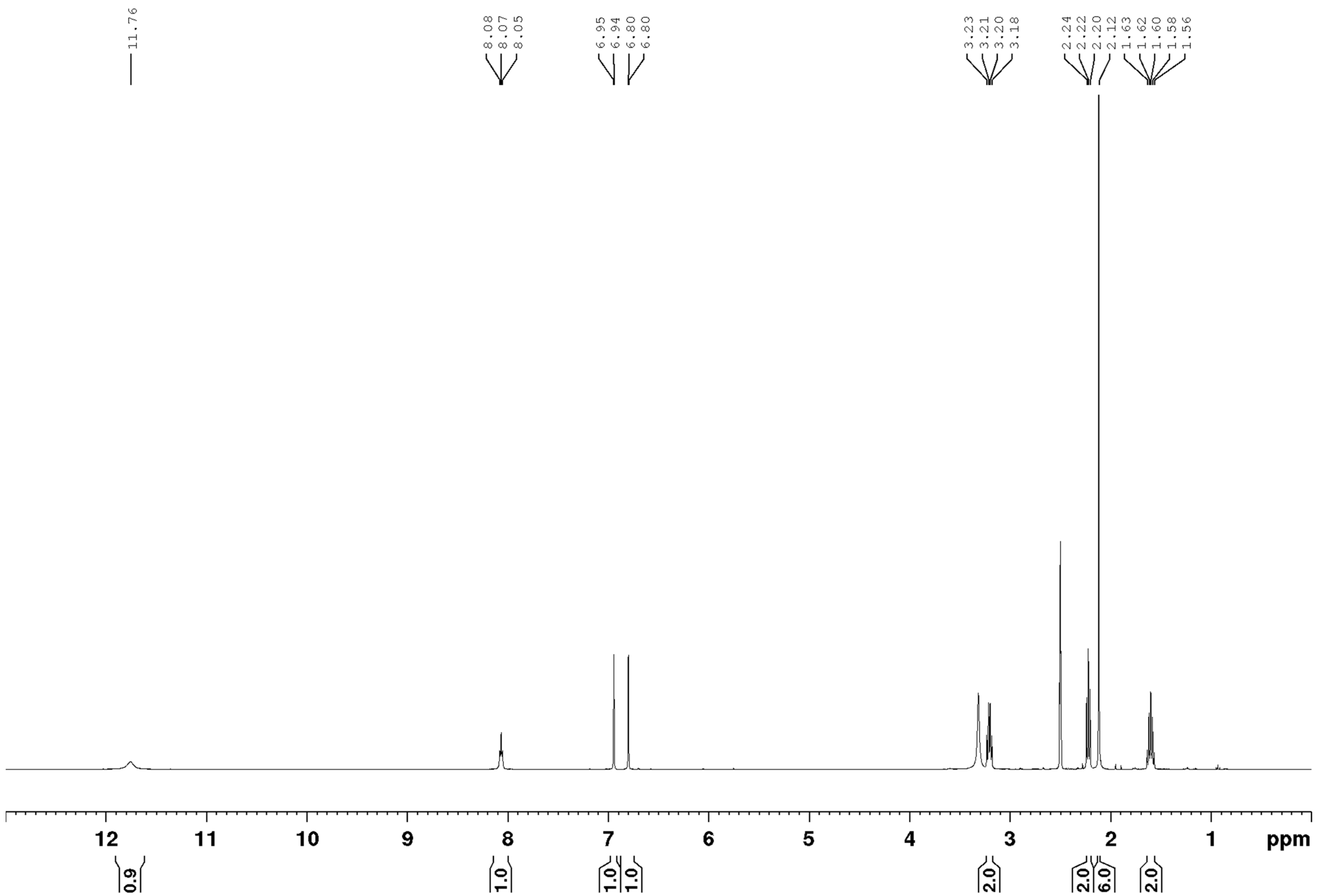

Figure x: ^{1}H NMR spectra of **27d** (400 MHz; DMSO-d_6).

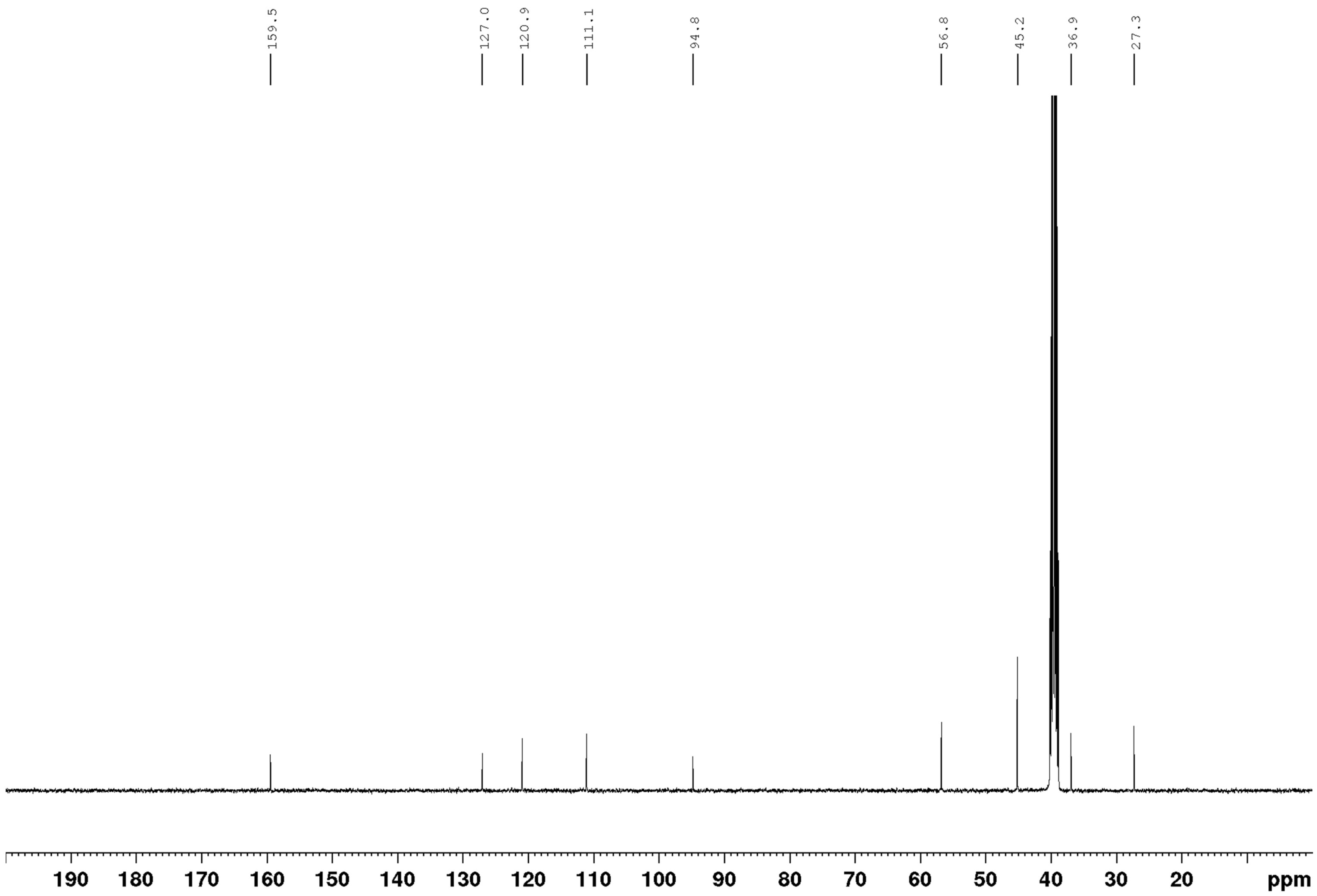

Figure x: ^{13}C NMR spectra of **27d** (100 MHz; DMSO-d_6).

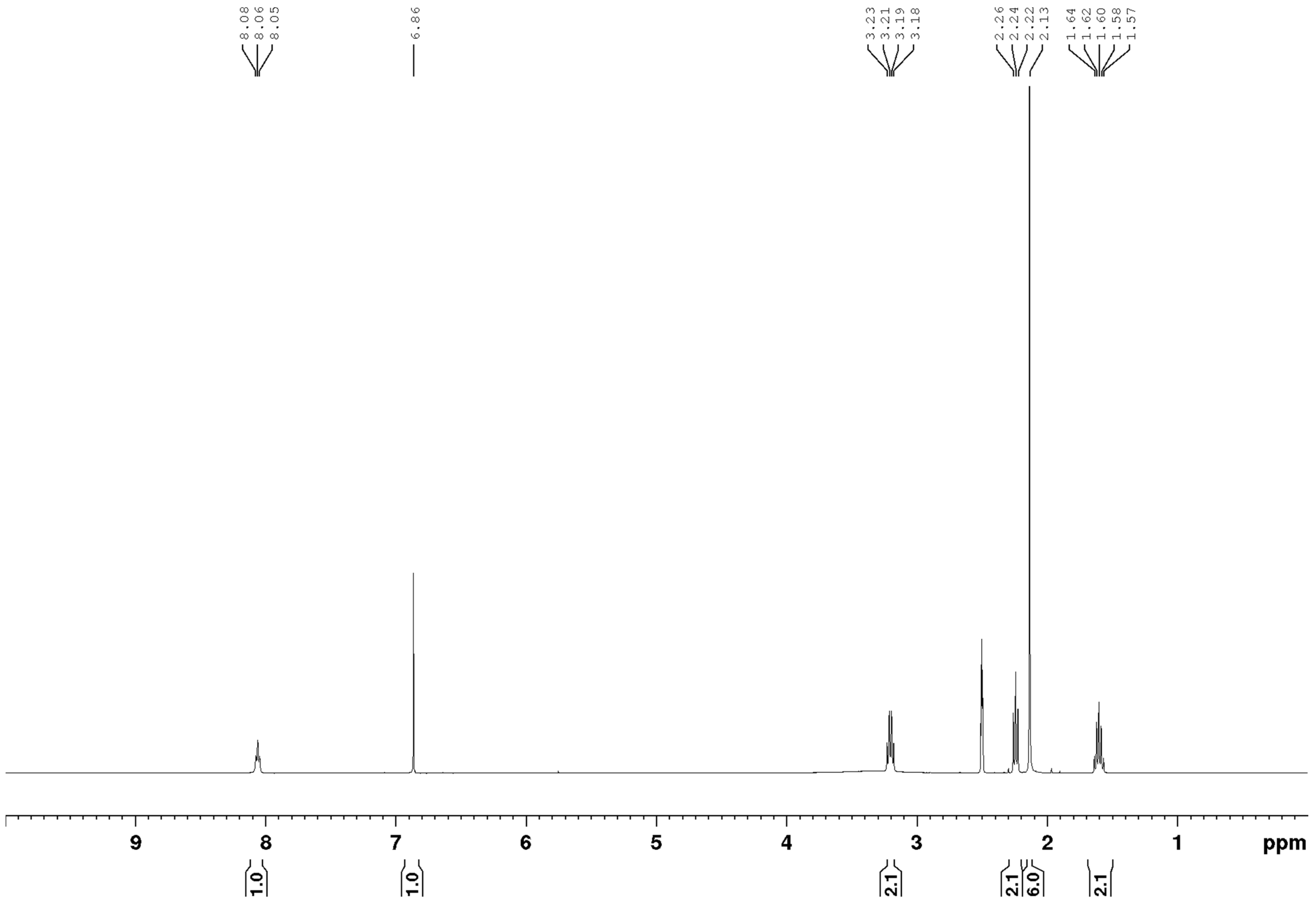

Figure x: ^{1}H NMR spectra of **27e** (400 MHz; DMSO-d_6).

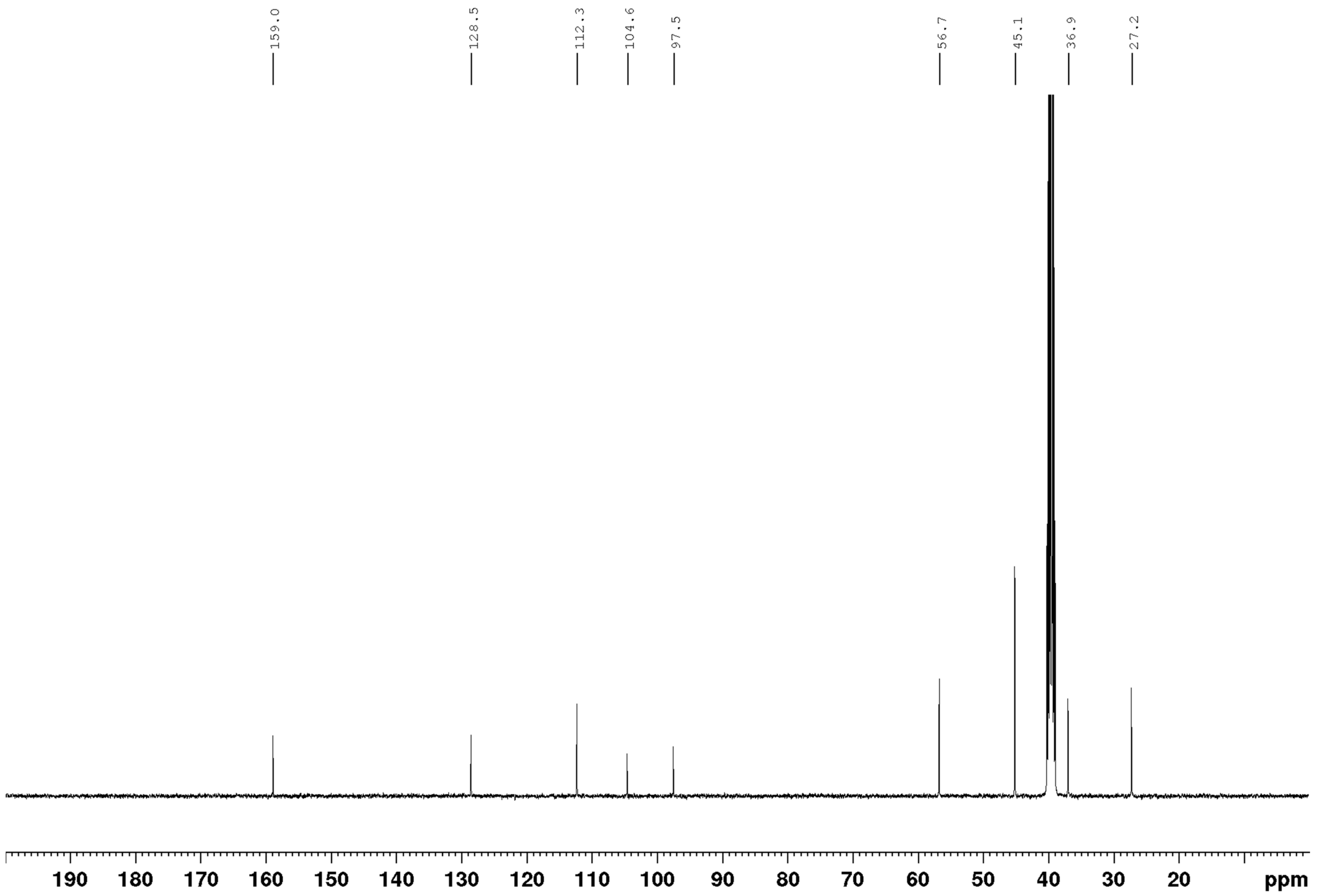

Figure x: ^{13}C NMR spectra of **27e** (100 MHz; DMSO-d_6).

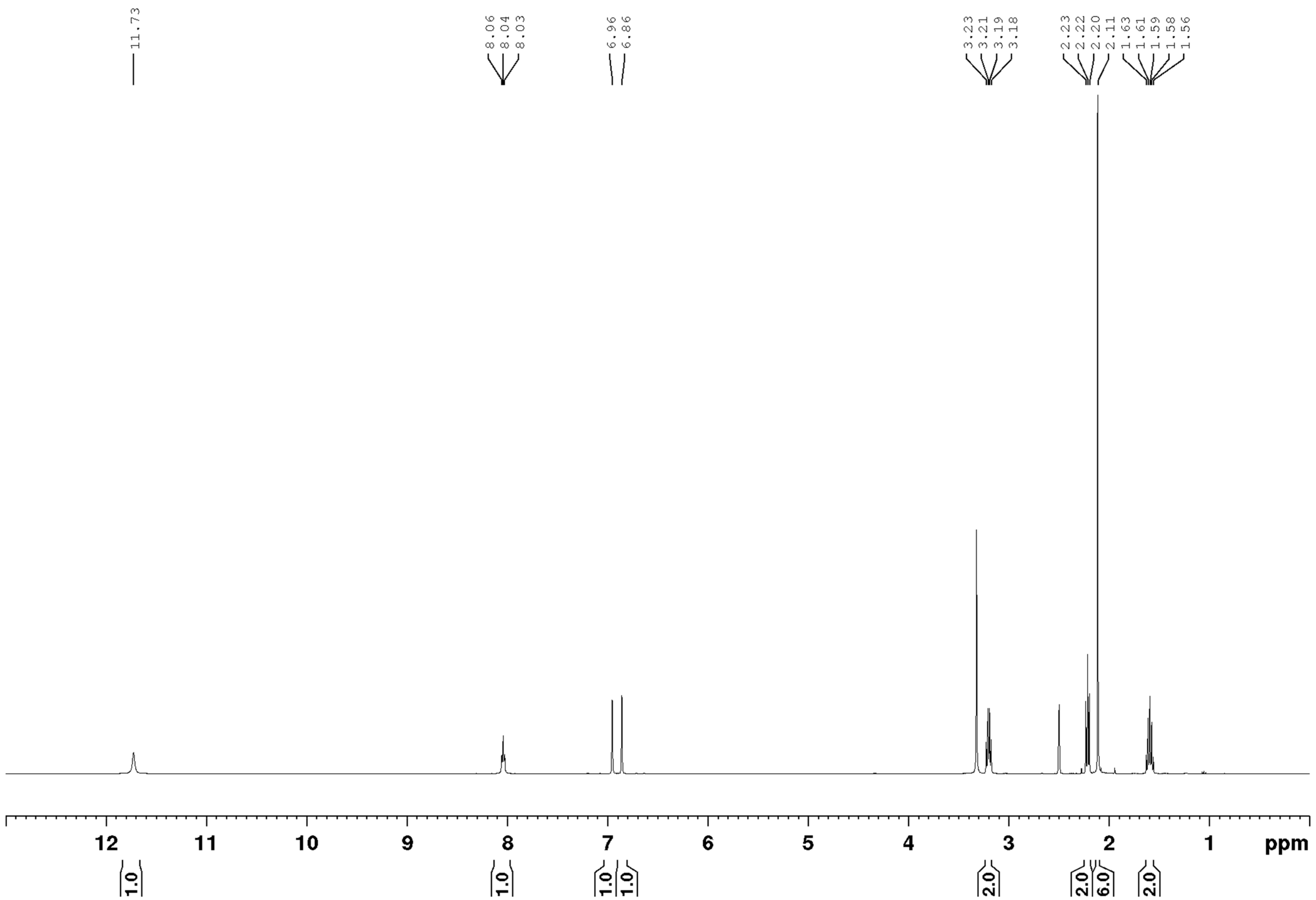

Figure x: ^{1}H NMR spectra of **27f** (400 MHz; DMSO-d_6).

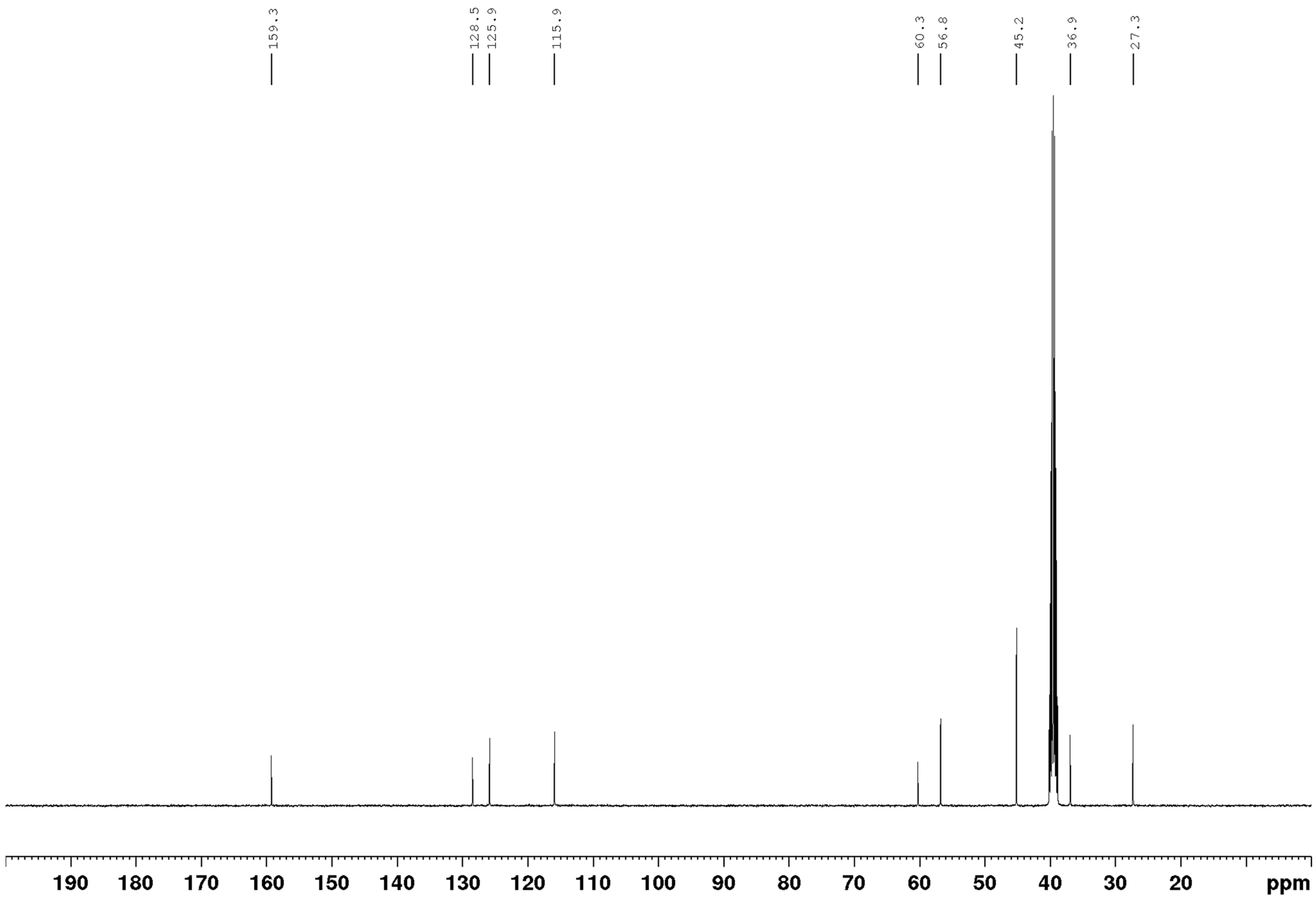

Figure x: ^{13}C NMR spectra of **27f** (100 MHz; DMSO-d_6).

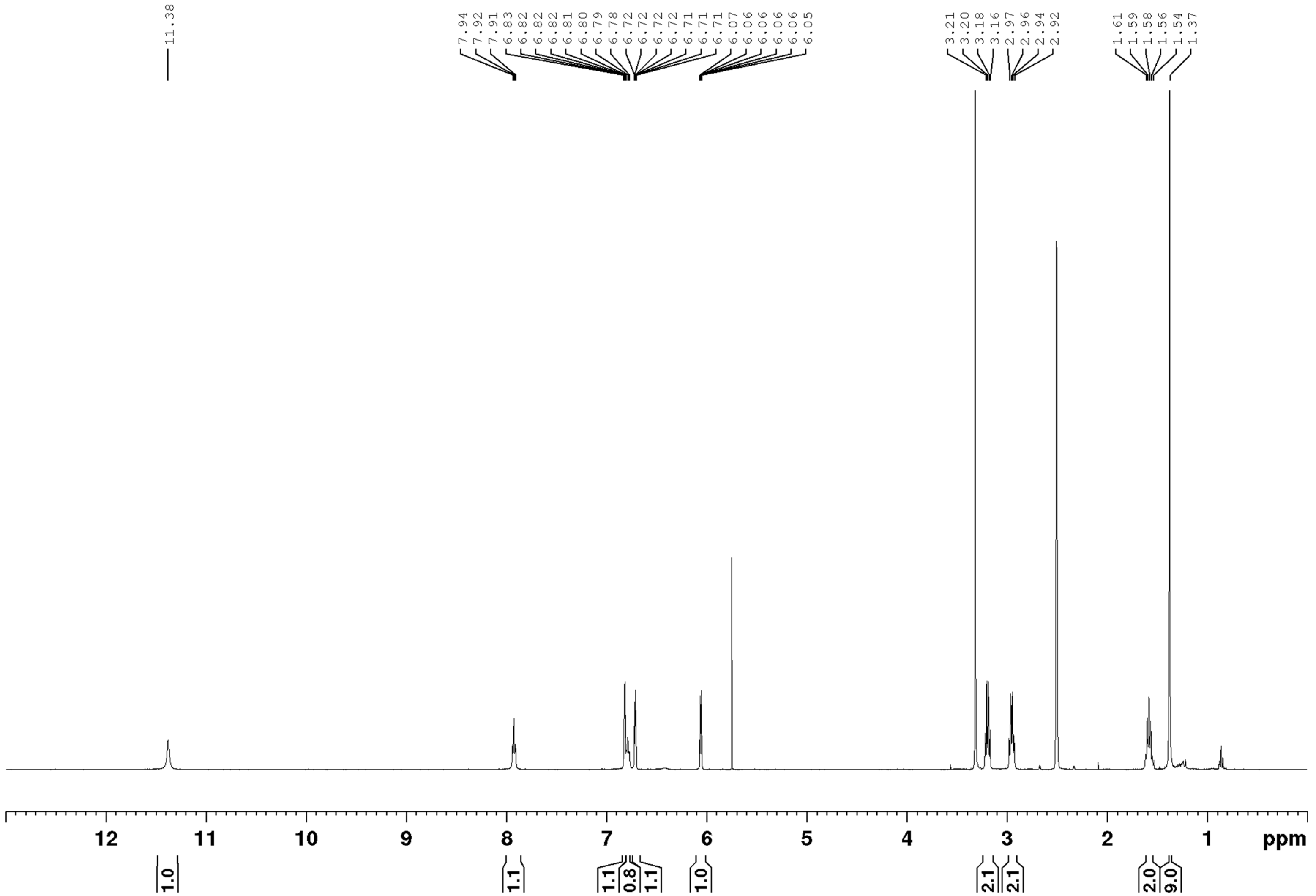

Figure x: ^{1}H NMR spectra of **28a** (400 MHz; DMSO-d_6).

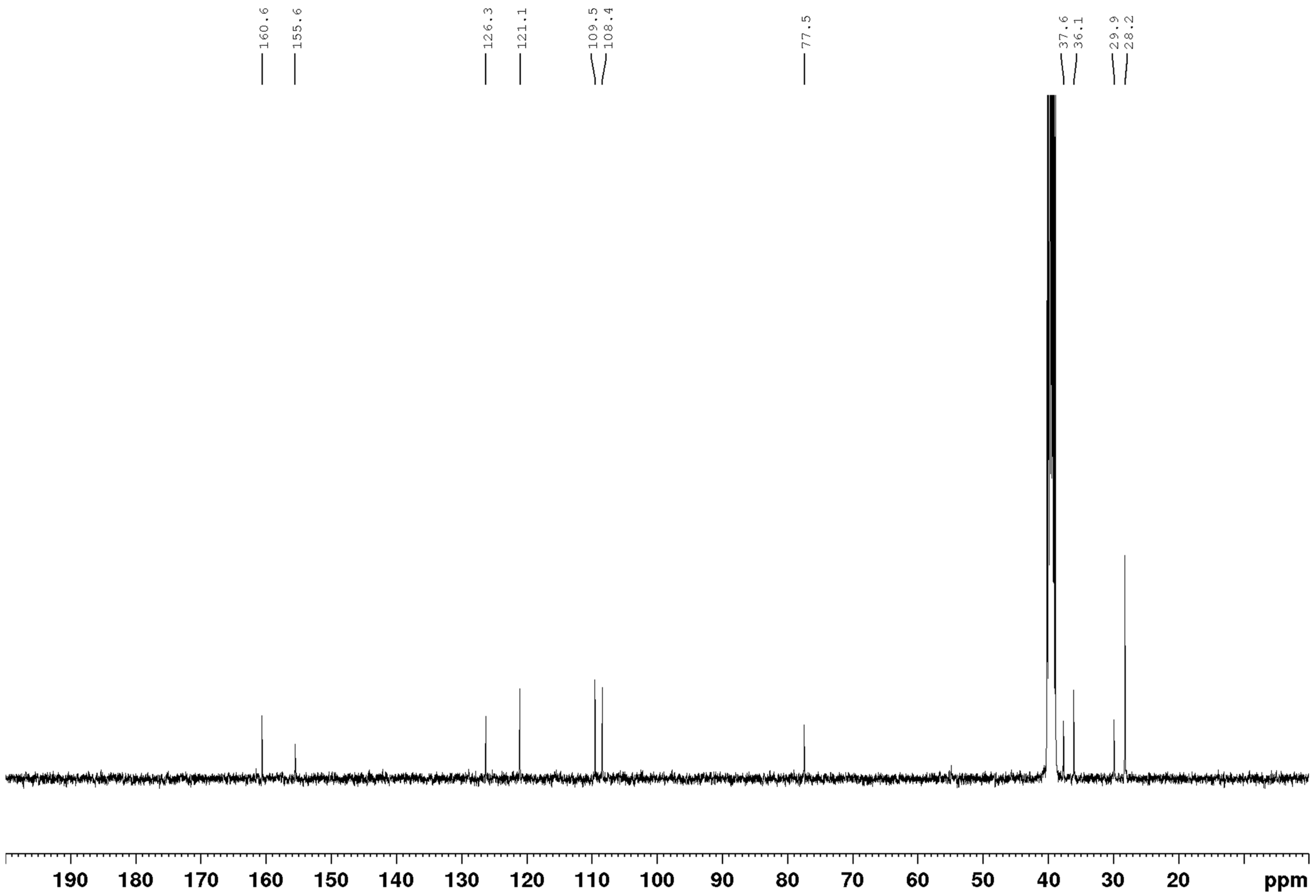

Figure x: ^{13}C NMR spectra of **28a** (100 MHz; DMSO-d_6).

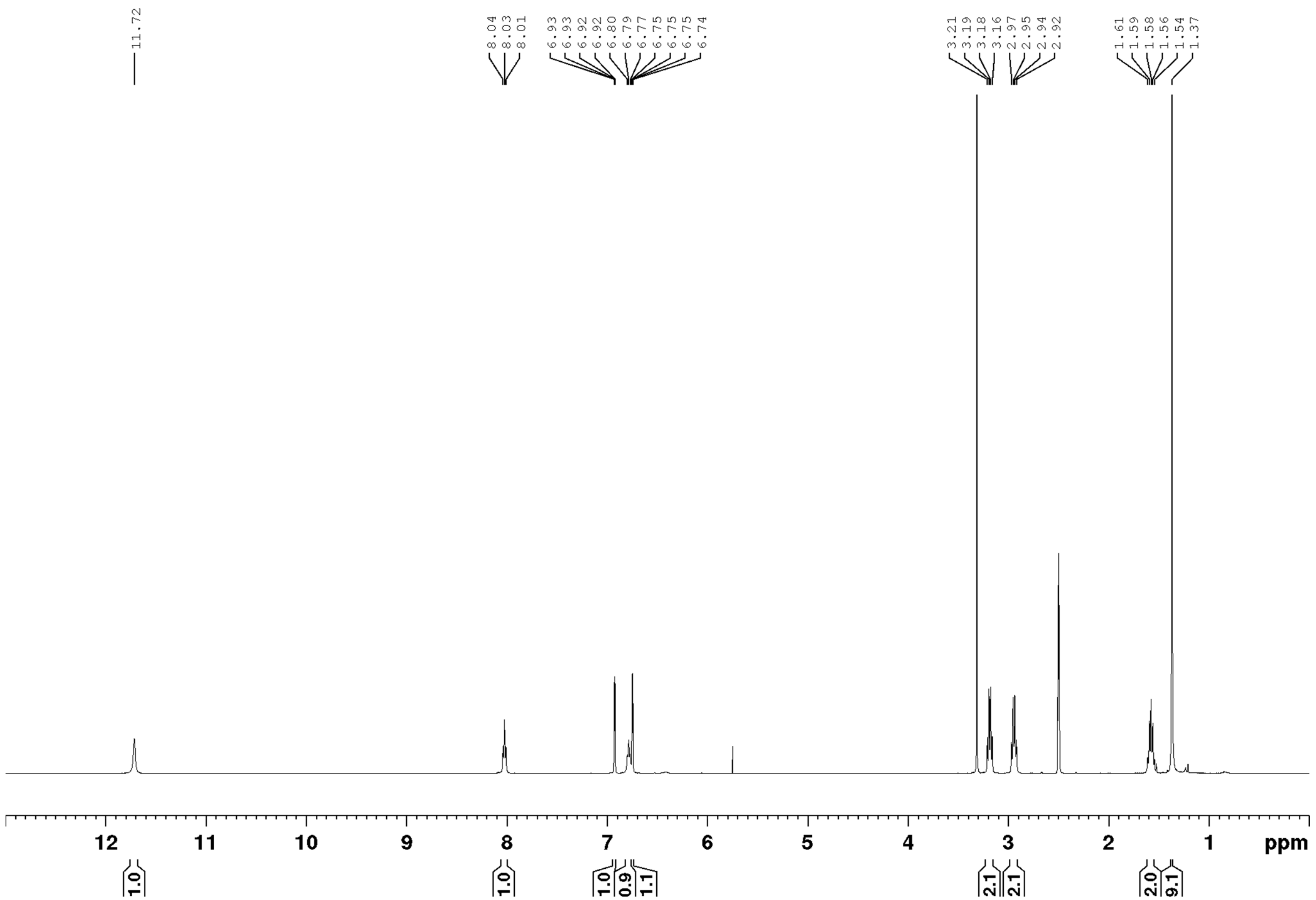

Figure x: ^{1}H NMR spectra of **28b** (400 MHz; DMSO-d_6).

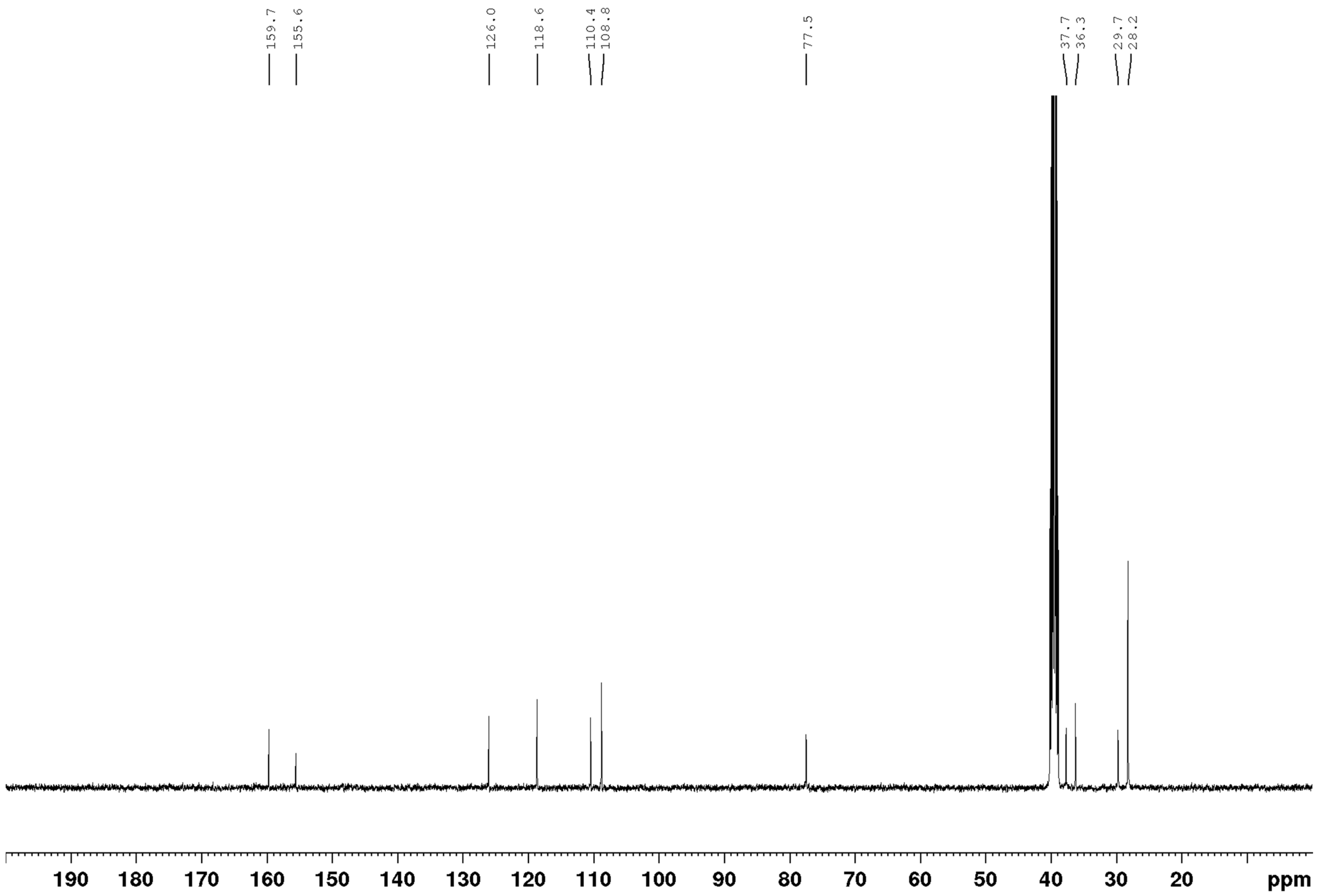

Figure x: ^{13}C NMR spectra of **28b** (100 MHz; DMSO-d_6).

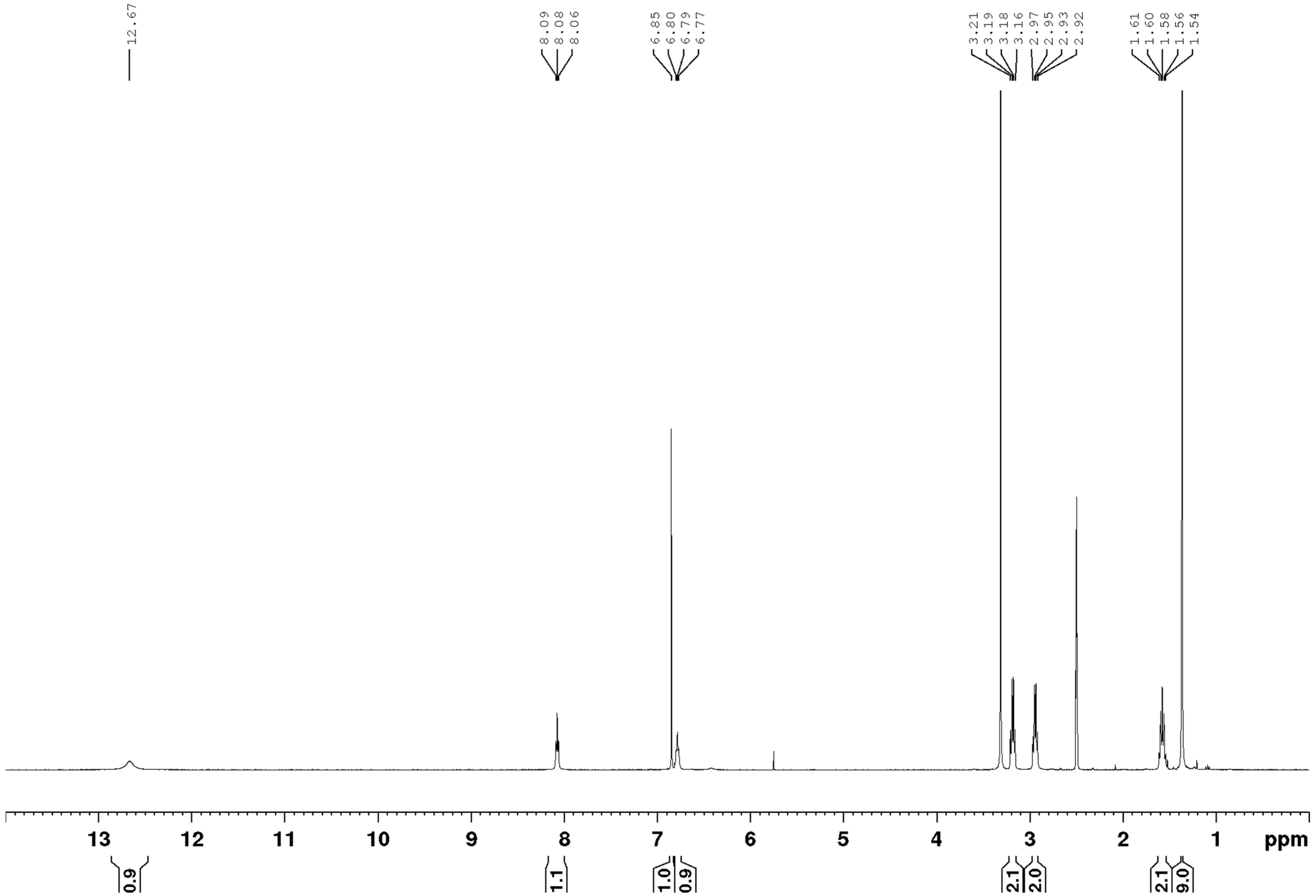

Figure x: ^{1}H NMR spectra of **28c** (400 MHz; DMSO-d_6).

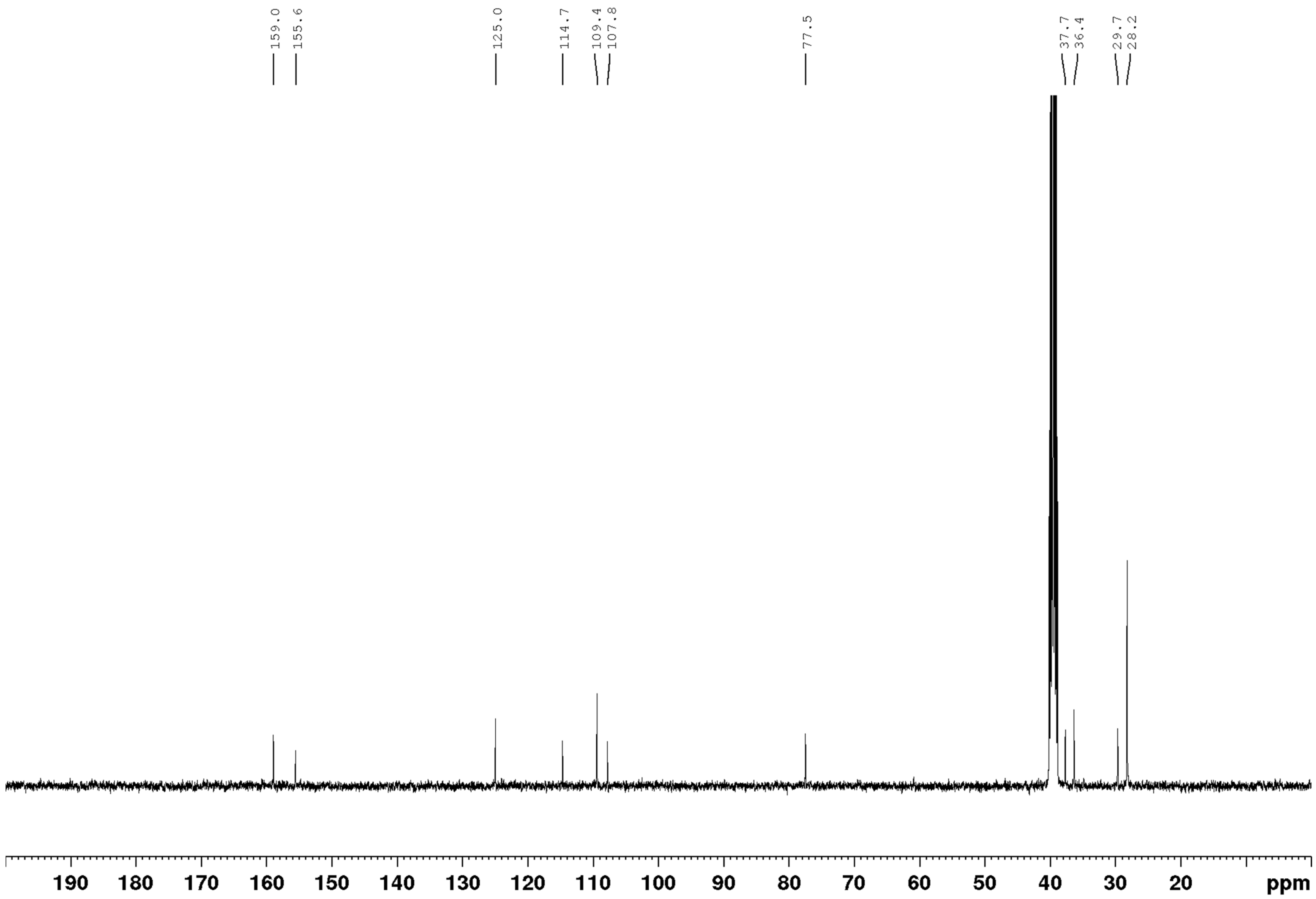

Figure x: ^{13}C NMR spectra of **28c** (100 MHz; DMSO-d_6).

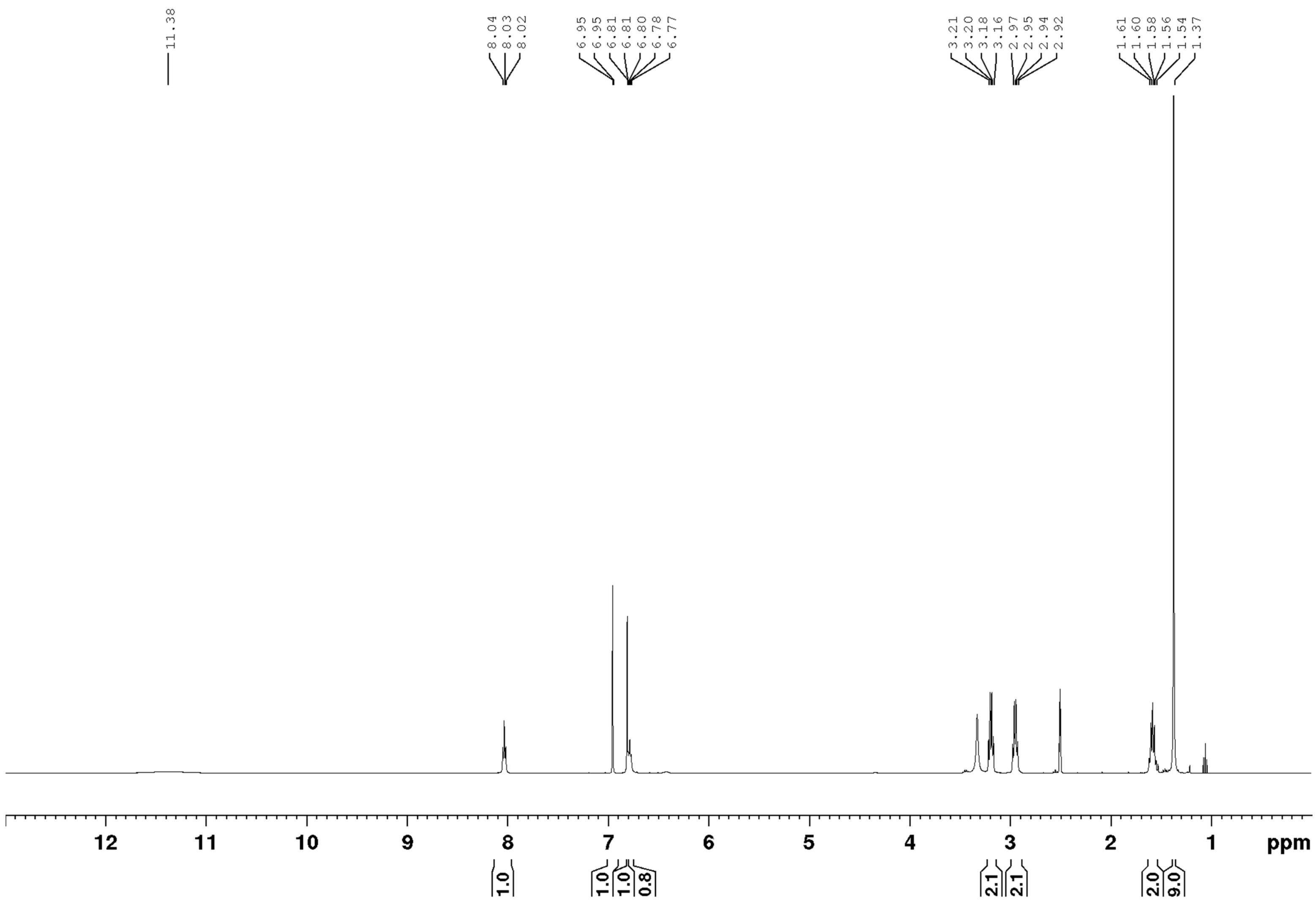

Figure x: ^{1}H NMR spectra of **28d** (400 MHz; DMSO-d_6).

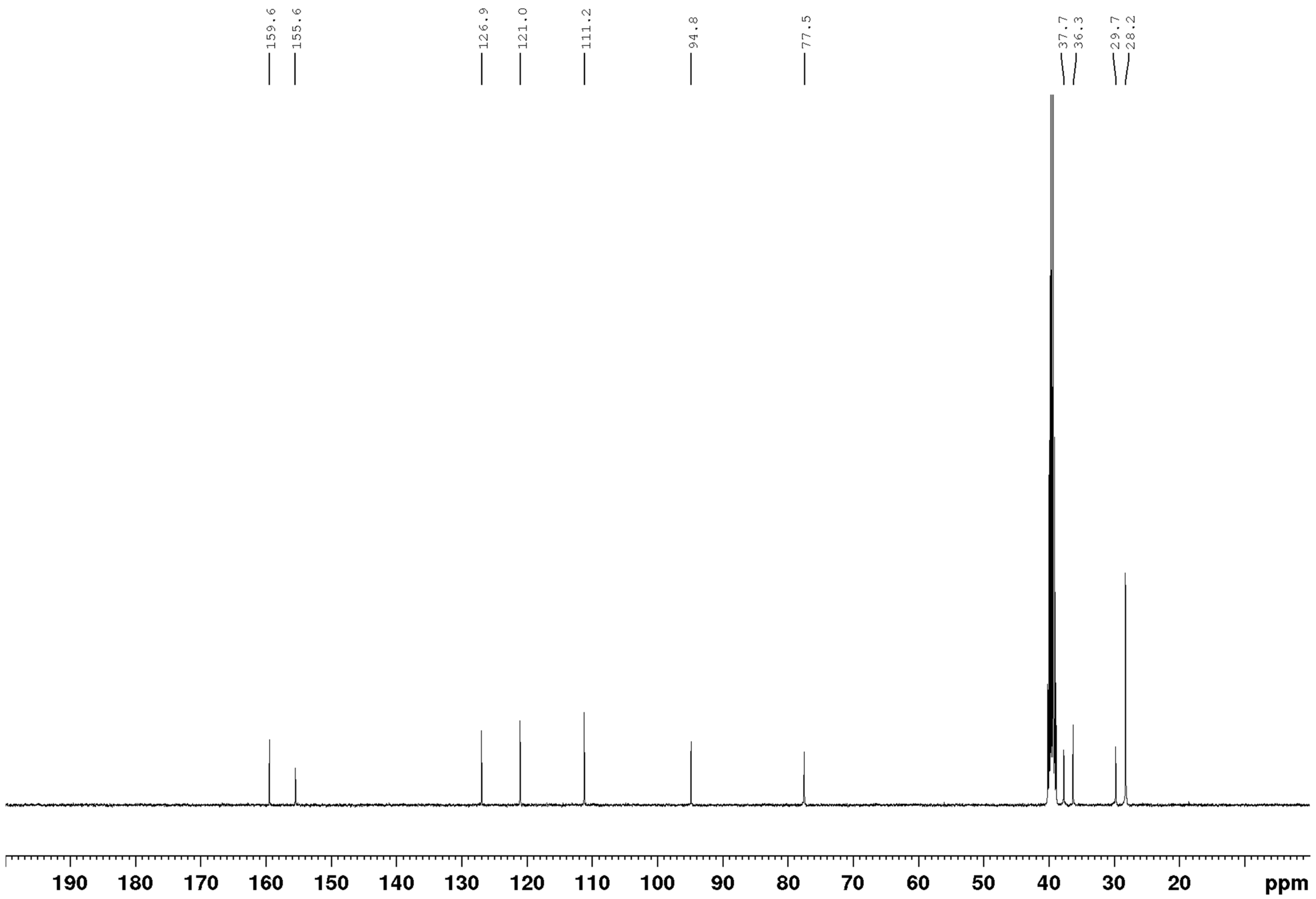

Figure x: ^{13}C NMR spectra of **28d** (100 MHz; DMSO-d_6).

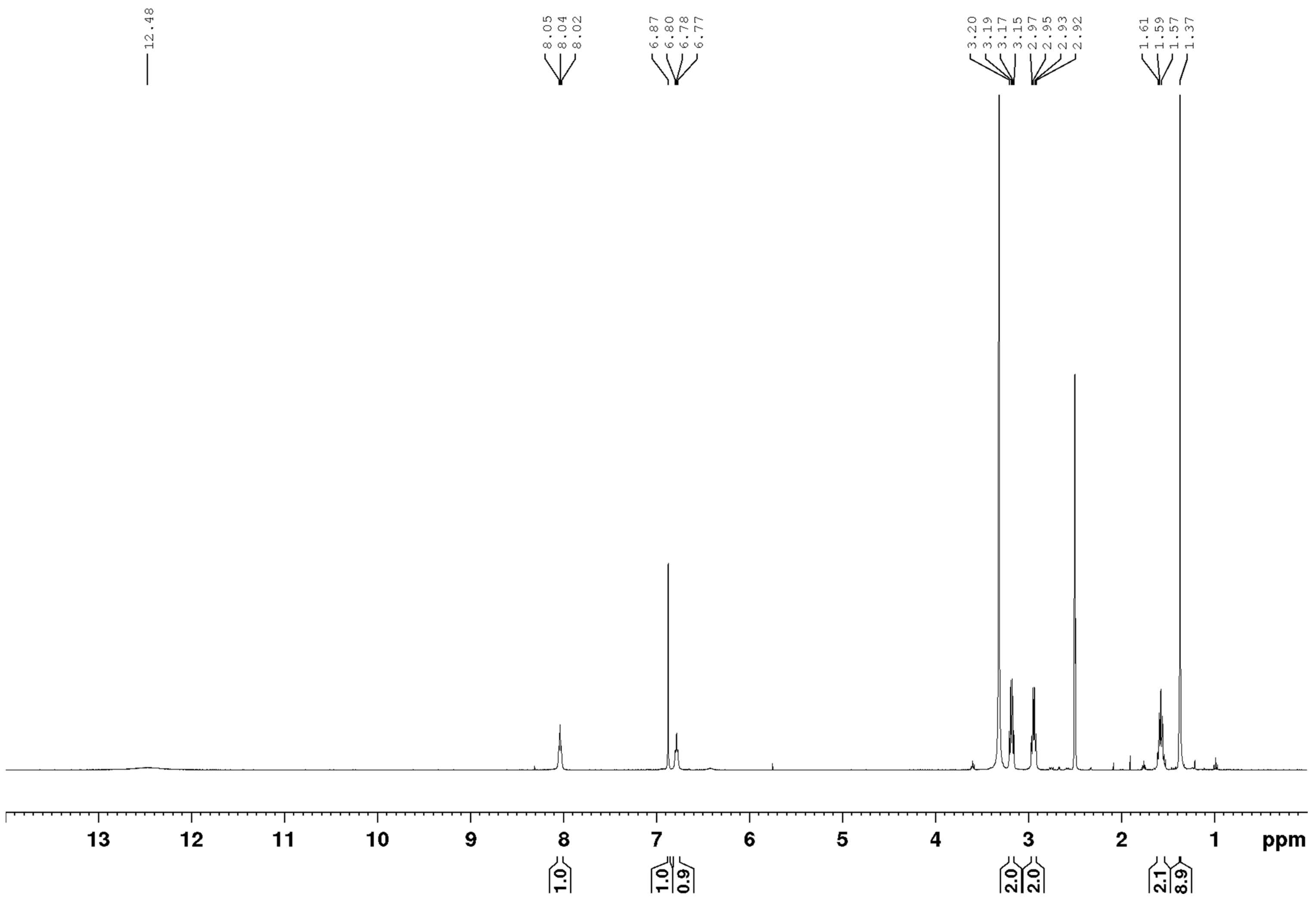

Figure x: ^{1}H NMR spectra of **28e** (400 MHz; DMSO-d_6).

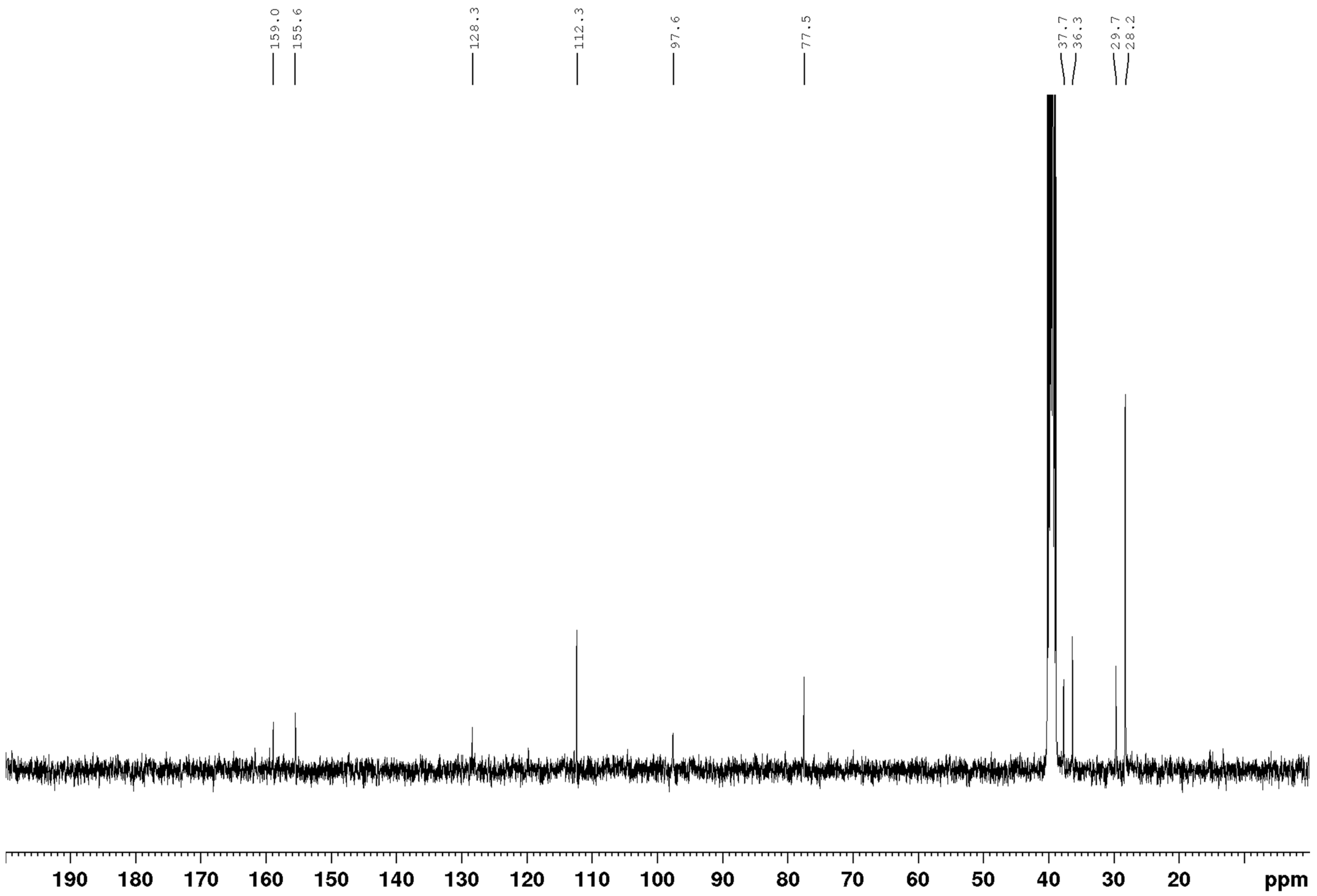

Figure x: ^{13}C NMR spectra of **28e** (100 MHz; DMSO-d_6).

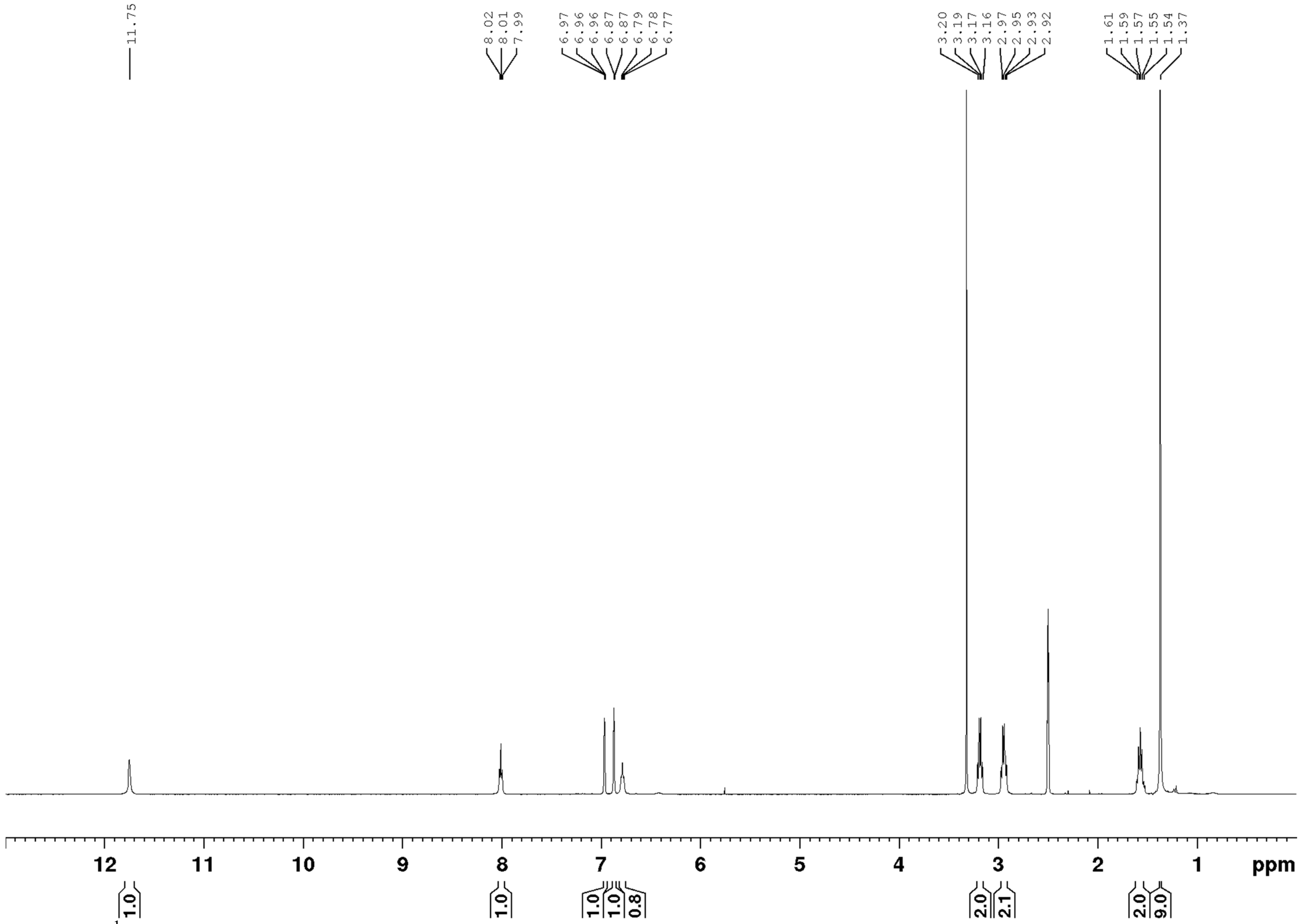

Figure x: ^{1}H NMR spectra of **28f** (400 MHz; DMSO-d_6).

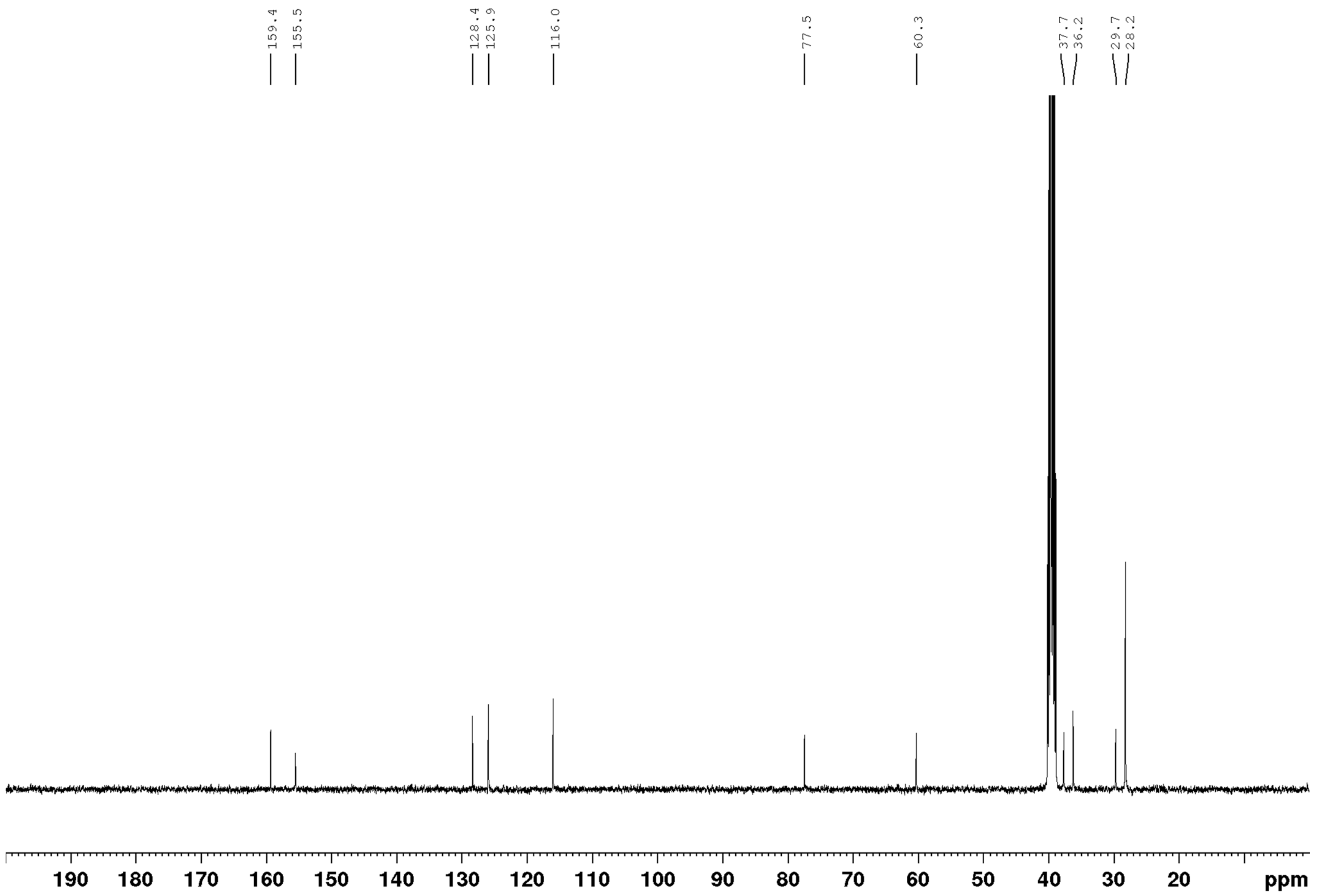

Figure x: ^{13}C NMR spectra of **28f** (100 MHz; DMSO-d_6).

References:

1. Gao, S.; Bethel, T. K.; Kakeshpour, T.; Hubbell, G. E.; Jackson, J. E.; Tepe, J. J. Substrate Controlled Regioselective Bromination of Acylated Pyrroles Using Tetrabutylammonium Tribromide ($TBABr_3$). *J. Org. Chem.* **2018**, *83* (16), 9250–9255.
2. Thach, O.; Mielczarek, M.; Ma, C.; Kutty, S. K.; Yang, X.; Black, D. StC.; Griffith, R.; Lewis, P. J.; Kumar, N. From Indole to Pyrrole, Furan, Thiophene and Pyridine: Search for Novel Small Molecule Inhibitors of Bacterial Transcription Initiation Complex Formation. *Bioorg. Med. Chem.* **2016**, *24* (6), 1171–1182.
3. Wang, M.; Zhang, Y.; Wang, T.; Wang, C.; Xue, D.; Xiao, J. Story of an Age-Old Reagent: An Electrophilic Chlorination of Arenes and Heterocycles by 1-Chloro-1,2-Benziodoxol-3-One. *Org. Lett.* **2016**, *18* (9), 1976–1979.
4. Rodriguez, R. A.; Pan, C.-M.; Yabe, Y.; Kawamata, Y.; Eastgate, M. D.; Baran, P. S. Palau'chlor: A Practical and Reactive Chlorinating Reagent. *J. Am. Chem. Soc.* **2014**, *136* (19), 6908–6911.
5. Essa, A. H.; Lerrick, R. I.; Tuna, F.; Harrington, R. W.; Clegg, W.; Hall, M. J. Reduction of 2,2,2-Trichloro-1-Arylethanones by RMgX: Mechanistic Investigation and the Synthesis of Substituted α,α-Dichloroketones. *Chem. Commun.* **2013**, *49* (27), 2756.
6. Fitzgerald, M. A.; Soltani, O.; Wei, C.; Skliar, D.; Zheng, B.; Li, J.; Albrecht, J.; Schmidt, M.; Mahoney, M.; Fox, R. J.; et al. Ni-Catalyzed C–H Functionalization in the Formation of a Complex Heterocycle: Synthesis of the Potent JAK2 Inhibitor BMS-911543. *J. Org. Chem.* **2015**, *80* (12), 6001–6011.
7. Parra, L. L. L.; Bertonha, A. F.; Severo, I. R. M.; Aguiar, A. C. C.; de Souza, G. E.; Oliva, G.; Guido, R. V. C.; Grazzia, N.; Costa, T. R.; Miguel, D. C.; et al. Isolation, Derivative Synthesis, and Structure–Activity Relationships of Antiparasitic

Bromopyrrole Alkaloids from the Marine Sponge *Tedania Brasiliensis*. *J. Nat. Prod.* **2018**, *81* (1), 188–202.

8. Banwell, M. G.; Hockless, D. C. R.; Flynn, B. L.; Longmore, R. W.; Rae, D. Assessment of Double-Barrelled Heck Cyclizations as a Means for Construction of the 14-Phenyl-8,9-Dihydro- 6H-[1]Benzopyrano[4′,3′:4,5]Pyrrolo[2,1-a]Isoquinolin- 6-One Core Associated with Certain Members of the Lamellarin Class of Marine Natural Product. *Aust. J. Chem.* **1999**, *52* (8), 755–766.
9. Behrens, C.; Christoffersen, M. W.; Gram, L.; Nielsen, P. H. A Convenient Synthesis of Pseudoceratidine and Three Analogs for Biological Evaluation. *Bioorg. Med. Chem. Lett.* **1997**, *7* (3), 321–326.
10. Tsukamoto, S.; Kato, H.; Hirota, H.; Fusetani, N. Pseudoceratidine: A New Antifouling Spermidine Derivative from the Marine Sponge Pseudoceratina Purpurea. *Tetrahedron Lett.* **1996**, *37* (9), 1439–1440.
11. Ponasik, J. A.; Conova, S.; Kinghorn, D.; Kinney, W. A.; Rittschof, D.; Ganem, B. Pseudoceratidine, a Marine Natural Product with Antifouling Activity: Synthetic and Biological Studies. *Tetrahedron* **1998**, *54* (25), 6977–6986.
